# Identification of T2W hypointense ring as a novel noninvasive indicator for glioma grade and IDH genotype

**DOI:** 10.1186/s40644-024-00726-3

**Published:** 2024-06-28

**Authors:** Yawen Lu, Ningfang Du, Xuhao Fang, Weiquan Shu, Wei Liu, Xinxin Xu, Yao Ye, Li Xiao, Renling Mao, Kefeng Li, Guangwu Lin, Shihong Li

**Affiliations:** 1 Department of Radiology, Huadong Hospital, Fudan University, No.220 West YanAn Road, Shanghai, 200040 China; 2 Department of Radiology, The Second Affiliated Hospital of Soochow University, Suzhou, China; 3 Department of Neurosurgery, Huadong Hospital, Fudan University, Shanghai, China; 4 Clinical Research Center for Gerontology, Huadong Hospital, Fudan University, Shanghai, China; 5 Department of Pathology, Huadong Hospital, Fudan University, Shanghai, China; 6 Center for AI-driven Drug Discovery, Faculty of Applied Sciences, Macao Polytechnic University, Macao, SAR China

**Keywords:** Glioma, Magnetic resonance imaging, Isocitrate dehydrogenase, T2W hypointense ring, T2-FLAIR mismatch

## Abstract

**Background:**

This study aimed to evaluate the T2W hypointense ring and T2-FLAIR mismatch signs in gliomas and use these signs to construct prediction models for glioma grading and isocitrate dehydrogenase (IDH) mutation status.

**Methods:**

Two independent radiologists retrospectively evaluated 207 glioma patients to assess the presence of T2W hypointense ring and T2-FLAIR mismatch signs. The inter-rater reliability was calculated using the Cohen’s kappa statistic. Two logistic regression models were constructed to differentiate glioma grade and predict IDH genotype noninvasively, respectively. Receiver operating characteristic (ROC) analysis was used to evaluate the developed models.

**Results:**

Of the 207 patients enrolled (119 males and 88 females, mean age 51.6 ± 14.8 years), 45 cases were low-grade gliomas (LGGs), 162 were high-grade gliomas (HGGs), 55 patients had IDH mutations, and 116 were IDH wild-type. The number of T2W hypointense ring signs was higher in HGGs compared to LGGs (*p* < 0.001) and higher in the IDH wild-type group than in the IDH mutant group (*p* < 0.001). There were also significant differences in T2-FLAIR mismatch signs between HGGs and LGGs, as well as between IDH mutant and wild-type groups (*p* < 0.001). Two predictive models incorporating T2W hypointense ring, absence of T2-FLAIR mismatch, and age were constructed. The area under the ROC curve (AUROC) was 0.940 for predicting HGGs (95% CI = 0.907–0.972) and 0.830 for differentiating IDH wild-type (95% CI = 0.757–0.904).

**Conclusions:**

The combination of T2W hypointense ring, absence of T2-FLAIR mismatch, and age demonstrate good predictive capability for HGGs and IDH wild-type. These findings suggest that MRI can be used noninvasively to predict glioma grading and IDH mutation status, which may have important implications for patient management and treatment planning.

**Supplementary Information:**

The online version contains supplementary material available at 10.1186/s40644-024-00726-3.

## Background

Glioma is the most common malignant primary brain tumor in adults [1]. Even though therapeutic advances have been made in treating temozolomide since its advent, coupled with the aggressive treatment of surgery and radiotherapy, the prognosis of patients with high-grade glioma (HGG) remains poor [2, 3]. Both the 2016 and 2021 World Health Organization (WHO) classifications of diffuse glioma have emphasized the value of molecular test results, including isocitrate dehydrogenase (IDH) genotypes [4–6]. Accurate preoperative glioma grading and prediction of IDH mutation status are crucial for individualized preoperative treatment planning and better prognosis prediction [4, 6].

Currently, the grading of glioma and IDH genotype mainly relies on surgery or biopsy, but elderly and frail patients with the poor conditions cannot tolerate these invasive methods. Secondly, due to the heterogeneity of the internal structure of gliomas, sampling bias may lead to errors in the grading and diagnosis [7]. Furthermore, limited availability of medical resources and the high cost of genetic testing can often limit a comprehensive diagnosis in such patients. Therefore, there is an urgent need for an imaging-based, non-invasive method to predict the molecular structure of these tumors.

Magnetic resonance imaging (MRI) has been widely used in the early diagnosis of glioma and preoperative WHO classification due to its high image contrast [8, 9]. In recent years, as the clinical significance of the IDH gene has become clearer, a few studies have been conducted to noninvasively predict IDH gene status using multi-parameter MRI, quantitative magnetic resonance imaging (qMRI), MRI-based radiomics, and deep learning [10–14]. However, there is still further potential for exploring the value of conventional MRI in cancer grading and IDH gene mutation prediction. “T2-FLAIR mismatch” sign has been described as a possible imaging marker for IDHmut [15, 16]. This sign can be used to diagnose IDHmut astrocytoma (IDH mutation, no 1p/19q coding deletion) before surgery, and its positive predictive value can be as high as 100% [17]. A previous study had found that gliomas with circular enhancement on gadolinium-enhanced T1 weighted MR imaging sequences might have hypointense rings on T2 weighted sequences [18]. However, circular enhancement and hypointense rings were not completely consistent [18]. During our clinical work, we also found that hypointense rings on glioma T2W MRI images were generally located between the tumor parenchyma and peritumoral edema or at the edge of the tumor. Whether it can be used as a useful marker to predict glioma grade or IDH genotype remains unclear.

The purpose of this study was to evaluate the T2W hypointense ring and T2-FLAIR mismatch signs in order to predict glioma grading and IDH mutation status.

## Methods

This retrospective study was approved by the Institutional Review Board (IRB) of Huadong Hospital, affiliated with Fudan University (2022K155). The requirement for informed consent was waived.

### Study population

A total of 419 patients with glioma diagnosed pathologically from May 2013 to July 2021 were collected. Inclusive criteria included: (1) No treatment before MRI examination; (2) All patients underwent MRI with or without contrast enhancement scan, with complete T2W image information; (3) All patients underwent surgical treatment and obtained postoperative pathological results. Exclusion criteria included: (1) Lacking complete image data; (2) No pathological result; (3) Complicated with other neurological diseases, such as cerebral infarction and cerebral hemorrhage; (4) Tumors located in the brain stem, ventricle, and sellar region; (5) Pilocytic astrocytoma (WHO Grade I).

### MRI parameters

The MR images were acquired at 3T MR scanners for all patients (MAGNETOM Trio, Vero, Skyra, Prisma, Vida; Siemes Healthcare in Erlangen, Germany). The MRI protocols included T1-weighted images both before (T1), and after (cT1) administration of gadolinium contrast agent (Gd-DTPA, 0.1mmol/kg, 3-5 ml/s), T2-weighted images (T2WI), fluid-attenuated inversion recovery sequence images (FLAIR), and diffusion-weighted imaging (DWI, including b = 0/1000 s/mm² and apparent diffusion coefficient (ADC) maps). All patients signed the informed consent forms before the enhanced MRI examination. The parameters of each sequence are shown in Supplementary Material.

### Histopathologic grading and analysis

All tissue specimens were fixed in paraffin blocks and then analyzed in the pathology department of our hospital. The tumors were classified as grades I, II, III, and IV according to the 2016 World Health Organization (WHO) Classification of Tumors of the Central Nervous System (Glioma-Related Classification and Grading). The specimens were further analyzed for IDH mutation status using immunohistochemistry and monoclonal antibodies that detect point mutations in the R132H gene of IDH1 in gliomas. Positive IDH1 expression was defined as IDHmut, while negative IDH1 expression was defined as IDHwt. A total of 171 patients were tested for IDH mutation status.

### Imaging analysis

MRI images were independently analyzed by two radiologists for the evaluation of T2W hypointense ring and T2-FLAIR mismatch signs in gliomas. Both radiologists were blinded to pathological diagnosis, age, and other conditions affecting the judgment of outcome.

The T2 hypointense ring was defined as being located between the tumor parenchyma and edema or at the edge of the glioma on T2W images, with lower signal intensity than the tumor parenchyma and edema. It might appear as a complete ring or arc (superior or inferior arc); a homogeneous or heterogeneous thin or thick ring. The T2-FLAIR mismatch sign was identified with the following criteria: (1) the presence of homogenous or near-homogenous hyperintense signal intensity on T2W images, (2) the presence of a relatively hypointense signal on FLAIR, except for a hyperintense thin peripheral rim, (3) necrotic cavities and small cysts were excluded, (4) by little or no contrast enhancement, (5) the degree of FLAIR signal suppression was inhomogeneous within the tumor [17, 19]. If there was any disagreement at the end of the evaluation, a second independent evaluation of the images was performed separately. If the disagreement persisted, a third experienced radiologist made the final decision. Although the focus of the study is on the existence of the T2W hypointense ring sign, some additional data was collected. We measured the ADC values of the T2W hypointense ring on the ADC maps. The region of interest (ROI) was drawn on the obvious T2W hypointense ring area using the brush tool of the software Miele-LXIV DICOM Workstation and Image Viewer (version 12.6, Alex Bettarini).

### Statistical analysis

Statistical analysis was performed using the SPSS v.26.0 software. Cohen’s kappa statistics (κ) were used to evaluate inter-rater agreement between two evaluators for assessing T2W hypointense ring and T2-FLAIR mismatch signs. κ values ≤ 0.2 are slight agreement, 0.21–0.4 are fair agreement, 0.41–0.6 are moderate agreement, and > 0.6 are significant agreement.

The Shapiro-Wilk test was conducted to test the normality of continuous variables and continuous variables with non-normal distribution were compared by nonparametric testing (Mann-Whitney test). Chi-square test was performed to evaluate the difference in WHO classification and IDH mutation status between gliomas with or without T2W hypointense ring and T2-FLAIR mismatch signs. Subsequently, logistic regression was conducted to evaluate the diagnostic accuracy of gender, age, T2W hypointense ring sign, no-T2-FLAIR mismatch sign, and ADC values of T2W hypointense ring for HGG or IDHwt status. Finally, characteristics with substantial consistency were used as predictor variables to predict HGGs or IDHwt gliomas, and receiver-operating characteristic (ROC) curves were performed. The performance of the models was evaluated by calculating the sensitivity, specificity, as well as the area under the curve (AUC). *p* < 0.05 was considered statistically significant for all tests.

## Results

### Cohort characteristics

**Fig. 1:**
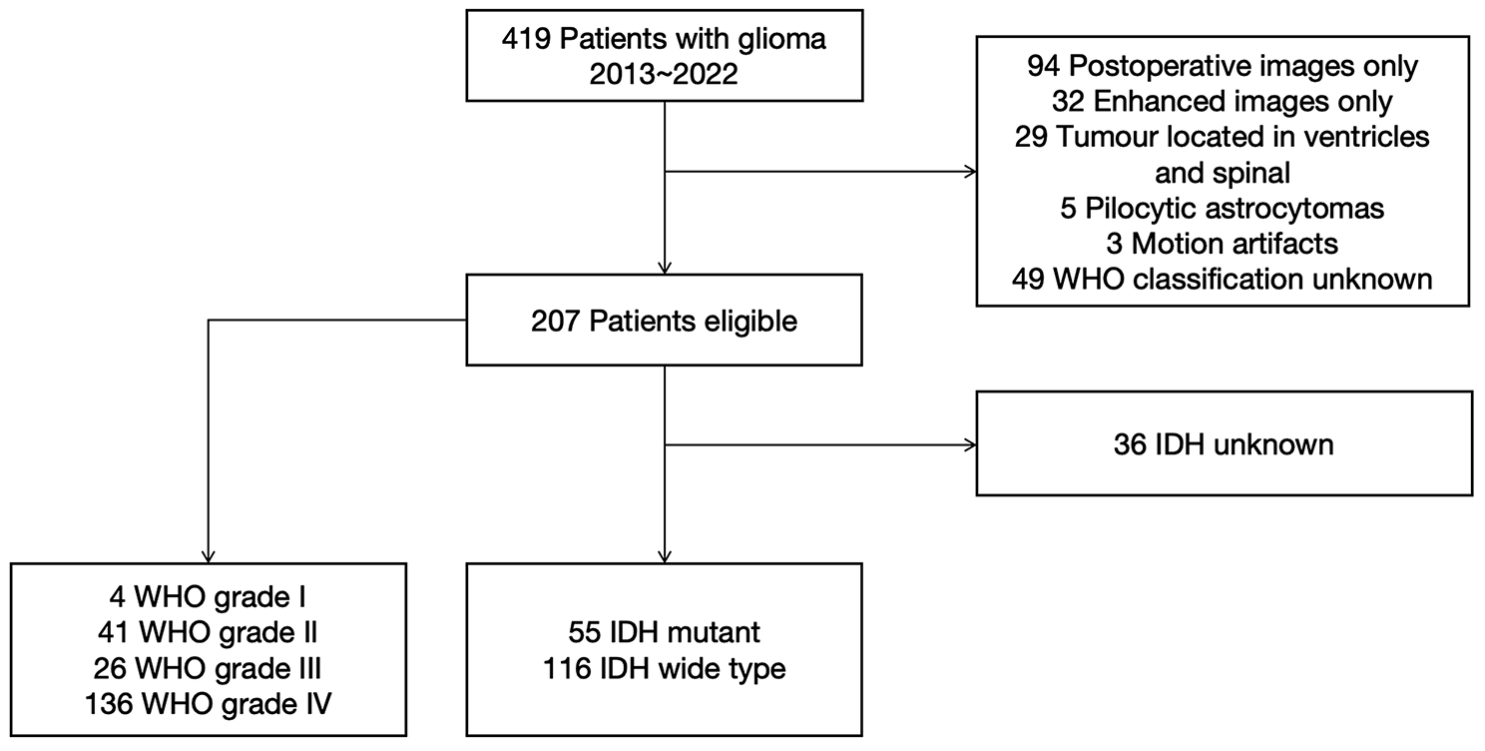
Flow diagram of the patient selection process

At the beginning of the study, there were 419 patients with gliomas. One hundred sixty-three (163) patients with unusable images (including only postoperative MRI images, only preoperative enhanced images, poor image quality) and 49 patients with unusable clinical data (including lesions located in ventricles, sellar region, or missing clinical data) were removed. 207 patients (age 51.6 ± 14.8 years; 119 males and 88 females) were included in the final analysis. The flowchart of case selection is shown in Fig. 1. Forty-five (45) cases were LGGs (4 cases of WHO Grade I, 41 cases of WHO Grade II), and 162 cases were HGGs (26 cases of WHO Grade III, 136 cases of WHO Grade IV). Of these, IDH mutation status was not available in 36 patients. Among the remaining 171 patients, 55 patients were IDHmut (32.16%), and 116 patients were IDHwt (67.84%) (Table 1).

**Table 1:** Comparison between gliomas with and without T2W hypointense ring sign and T2-FLAIR mismatch sign

	All (*n* = 207)		Z/χ^2^	*p* value	All (*n* = 184)^*b*^		Z/χ^2^	*p* value
	T2W hypointense ring (*n* = 143)	No T2W hypointense ring (*n* = 64)			T2-FLAIR mismatch (*n* = 29)	No-T2-FLAIR mismatch (*n* = 155)		
***Gender***			0.297	0.586			2.739	0.098
Male	84 (58.74%)	35 (54.69%)			12 (41.38%)	93 (60.00%)		
Female	59 (41.26%)	29 (45.31%)			17 (58.62%)	62 (40.00%)		
***Age at diagnosis (y)***	56 (47, 65)	41.5 (33.25, 48.75)	-6.200	**< 0.001**	42 (33.5, 48)	55 (44, 65)	-4.503	**< 0.001**
***WHO***			/	**< 0.001** ^***c***^			43.700	**< 0.001**
LGGs	4 (2.80%)	41 (64.06%)			20 (68.97%)	19 (12.26%)		
HGGs	139 (97.20%)	23 (35.94%)			9 (31.03%)	136 (87.74%)		
***Molecular subtype*** ^*a*^			36.000	**< 0.001**			/	**< 0.001** ^***c***^
IDHmut	21 (17.80%)	34 (64.15%)			19 (90.47%)	30 (22.73%)		
IDHwt	97 (82.20%)	19 (35.85%)			2 (9.52%)	102 (77.27%)		
Unknown	25	11			8	23		

^*a*^ Molecular subtype was available for a total of 171 cases

^*b*^ There were 184 cases with T2-FLAIR images, 153 cases of which had gene testing

^*c*^ Fisher’s exact test

LGGs: Low-grade gliomas; HGGs: High-grade gliomas; IDH: Isocitrate dehydrogenase; T2W: T2-weighted; FLAIR: Fluid-attenuated inversion recovery

### Assessment of T2W hypointense ring sign

For the independent assessment of the T2W hypointense ring sign, the results of the two evaluators were 137 cases (66.18%) and 145 cases (70.05%), respectively. Eight cases (3.86%) had discordant judgment. The Cohen’s κ coefficient was 0.85. After reassessment, the still disagreed images were reviewed by a third radiologist. Finally, T2W hypointense ring sign was considered positive in 143 gliomas (69.08%), while 64 gliomas (30.92%) lacked an obvious T2 hypointense ring sign. Table 1 shows the correlation assessment of the T2W hypointense ring sign.

**Fig. 2:**
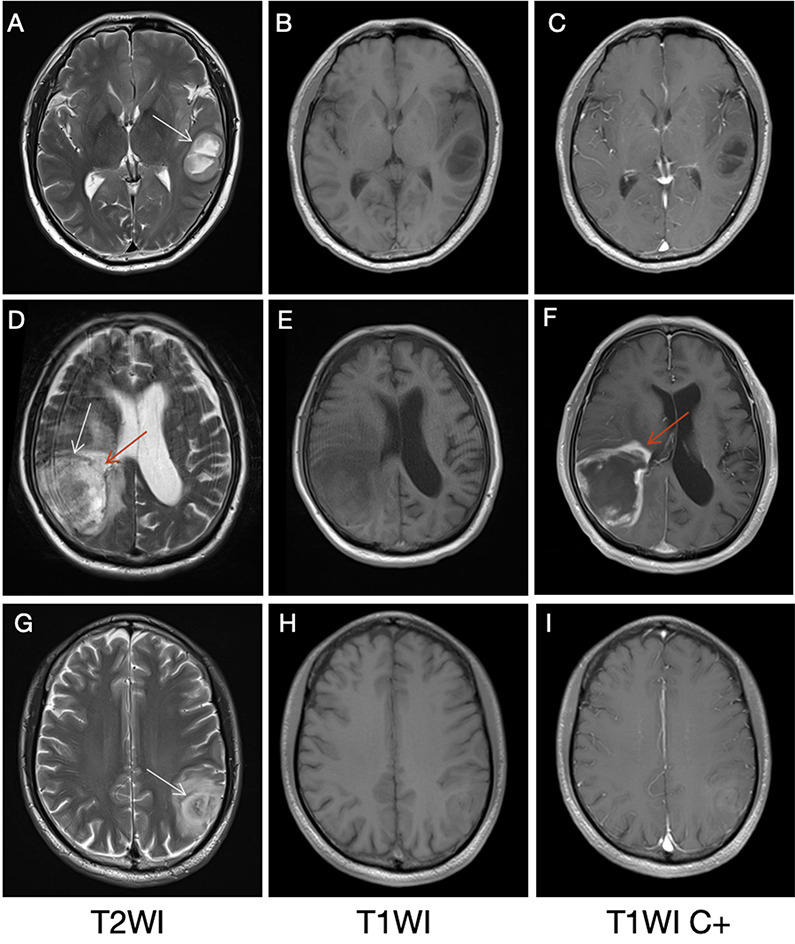
T2W hypointense ring sign in three cases (white arrows). From left to right are T2WI, T1WI, and contrast-enhanced T1WI, respectively. Images **(A-C)** showed a patient (male, 51 years) with glioblastoma (IDHwt) in the left temporal lobe, no obvious edema around the tumor parenchyma on T2W, and no enhancement on the contrast-enhanced T1W image. **(D-F)** showed a patient (male, 59 years) with glioblastoma (IDHwt) in the right temporoparietal lobe. The T2W hypointense ring on T2WI was not completely consistent with the enhanced morphology, as indicated by the medial red arrow, where the T2W hypointense ring broke, which might suggest glioma invasion (red arrows). **(G-I)** A patient (male, 35 years) with an oligodendroglioma, IDHmut, showed a clear low signal arc on T2WI, and the tumor parenchyma was mildly enhanced

T2W hypointense ring signs were present in 85.80% (139/162) HGGs (WHO III, IV) and only in 8.89% (4/45) LGGs (WHO I, II). A significant difference existed between the two groups (*p* < 0.001). In all patients with IDH genetic detection results, T2W hypointense ring signs were shown in 83.62% (97/116) IDHwt gliomas and 38.18% (21/55) IDHmut cases. There was a significant difference between IDHwt and IDHmut gliomas (*p* < 0.001). The age of the patients without T2W hypointense ring was significantly lower than that of patients with the signs (*p* < 0.001). There was no significant difference in gender between tumors with and without the T2W hypointense ring (*p* = 0.586). Figure 2 shows three patients with positive hypointense ring signs.

### Assessment of T2-FLAIR mismatch sign

The FLAIR images were missing in 23 patients, and the rest of 184 patients were evaluated for T2-FLAIR mismatch signs. The presence of T2-FLAIR mismatch sign was assessed independently by two radiologists. The Cohen’s κ coefficient of consistency between two radiologists was 0.84. After resolving the discordant cases, the T2-FLAIR mismatch sign was present in 29 cases (15.76%) and absent in 155 cases (84.24%). Table 1 shows the assessment of the T2-FLAIR mismatch sign.

**Fig. 3:**
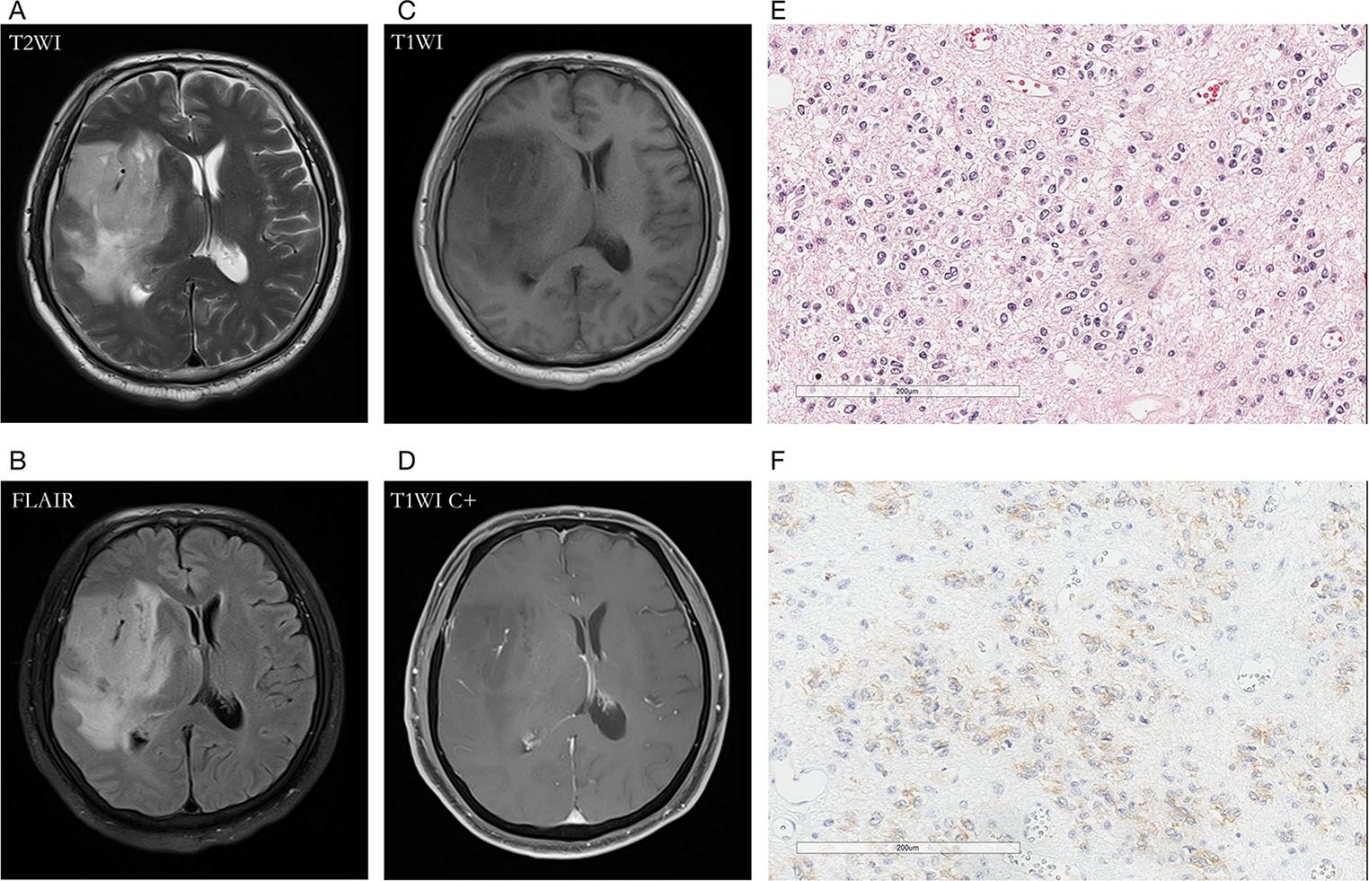
A 60-year-old male patient with oligodendroglioma (WHO Grade III, IDHmut) in the right temporal lobe. **(A, B)** axial T2WI showed T2W hyperintense lesions in tumor parenchyma, absence of T2W hypointense ring, and T2-FLAIR mismatch signs; **(C, D)** contrast-enhanced T1W image showed no obvious enhancement; **(E)** HE staining showed moderate to a severe increase in cell density, accompanied by the atypical nucleus, and perinuclear halo (×200); **(F)** IDH1 mutation positive expression

**Fig. 4:**
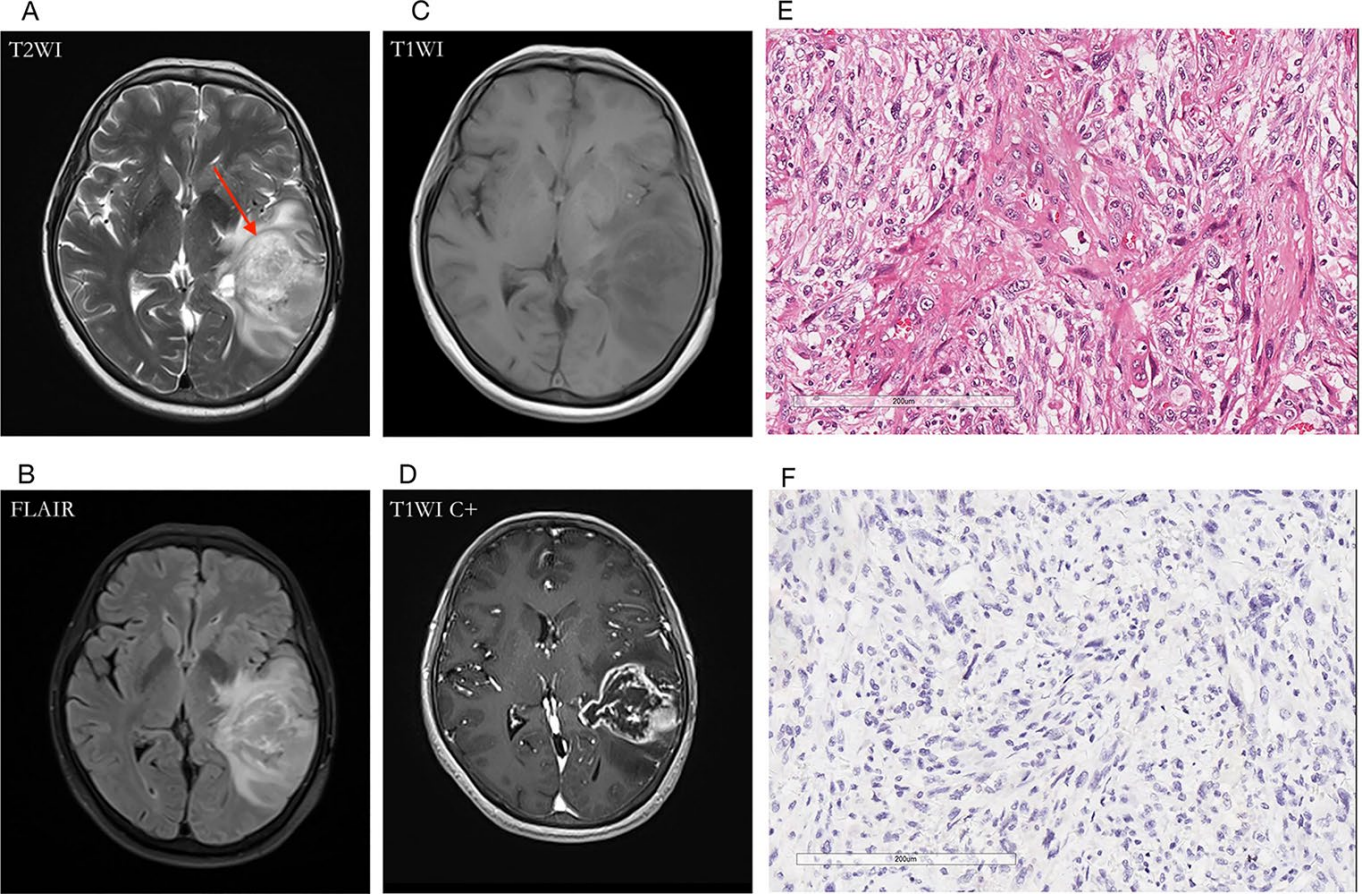
A 64-year-old female with glioblastoma (WHO Grade IV, IDHwt) in the left temporal lobe. **(A)** T2WI showed a hyperintense tumor, and the internal signal is inhomogeneous. The hypointense ring was located between the tumor parenchyma and edema as the red arrow indicated; **(B)** T2-FLAIR mismatch sign was absent; **(C, D)** Contrast-enhanced T1W image showed garland-like contrast enhancement; **(E)** HE staining showed dense cells, obvious atypia, and vascular endothelial hyperplasia (×200); **(F)** IDH1 mutation negative expression (×200)

**Fig. 5:**
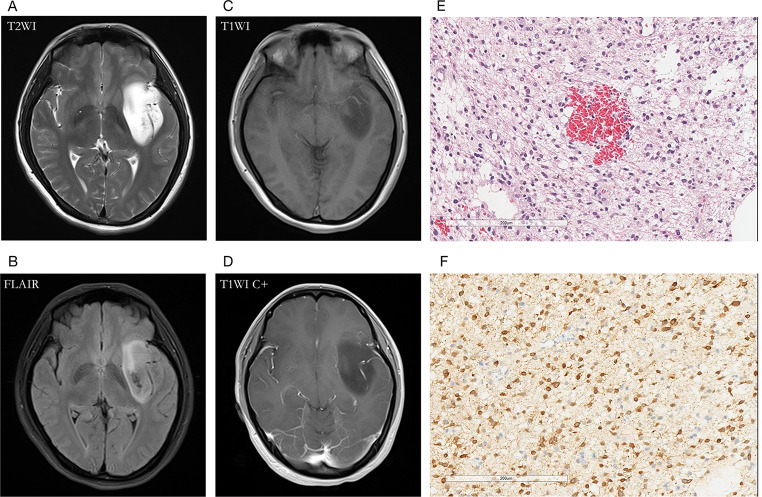
A 30-year-old female with diffuse astrocytoma (WHO Grade II, IDHmut) in the left temporal lobe. **(A)** A hyperintense tumor showed in T2W MRI, without hypointense ring sign; **(B)** T2-FLAIR mismatch sign was positive; **(C, D)** Contrast-enhanced T1W image showed no obvious enhancement; **(E)** HE staining showed moderate increase in cell density with atypical nuclei (×200); (F) IDH1 mutation positive expression (×200)

**Fig. 6:**
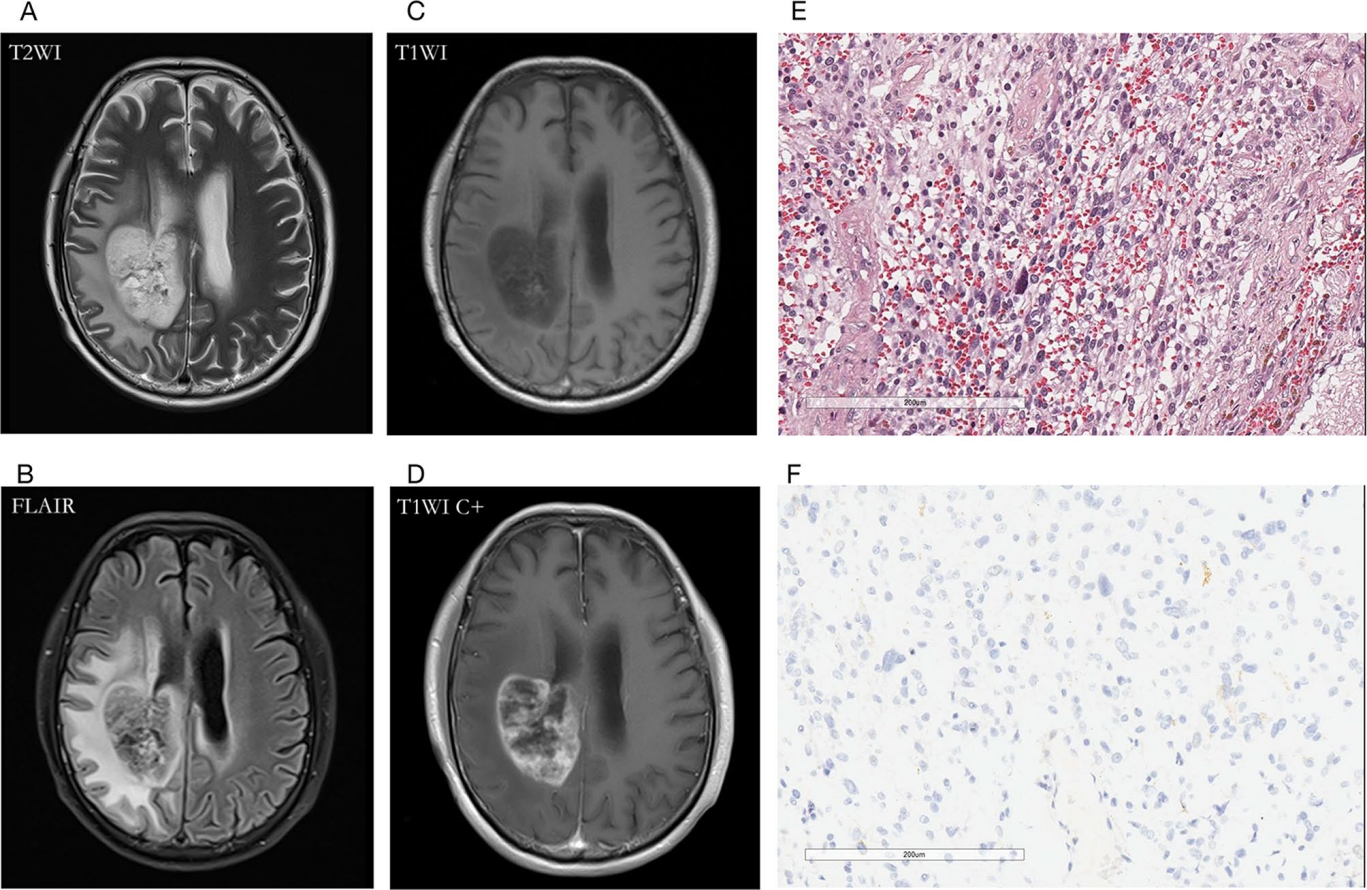
A 62-year-old male with glioblastoma (WHO Grade IV, IDH-wt) in the right temporoparietal lobe. **(A)** The T2WI demonstrated a hypointense ring between the tumor parenchyma and edema; **(B)** T2-FLAIR mismatch sign was negative for the necrotic cavity; **(C, D)** Contrast-enhanced T1W image showed garland-like contrast enhancement; **(E)** HE staining with moderate to high cell proliferation (×200); **(F)** IDH1 mutation negative expression (×200)

Compared with the LGGs group, HGGs showed more no-T2-FLAIR mismatch signs (87.74%, *p* < 0.001). There were 154 cases with IDH genotype detections and T2-FLAIR images concurrently. Compared with the IDHmut group, IDHwt gliomas were more likely to show no-T2-FLAIR mismatch sign (77.27%, *p* < 0.001). There was no difference in gender between tumors with versus without the T2-FLAIR mismatch sign. Figures 3, 4, 5 and 6 showed the typical MRI and pathological features of four gliomas with T2W hypointense ring and T2-FLAIR mismatch signs. In addition, we performed a secondary analysis of the T2W hypointense ring and T2-FLAIR mismatch signs, see Supplementary Material for details.

### Prediction of LGGs and HGGs groups by logistic regression analysis

**Fig. 7:**
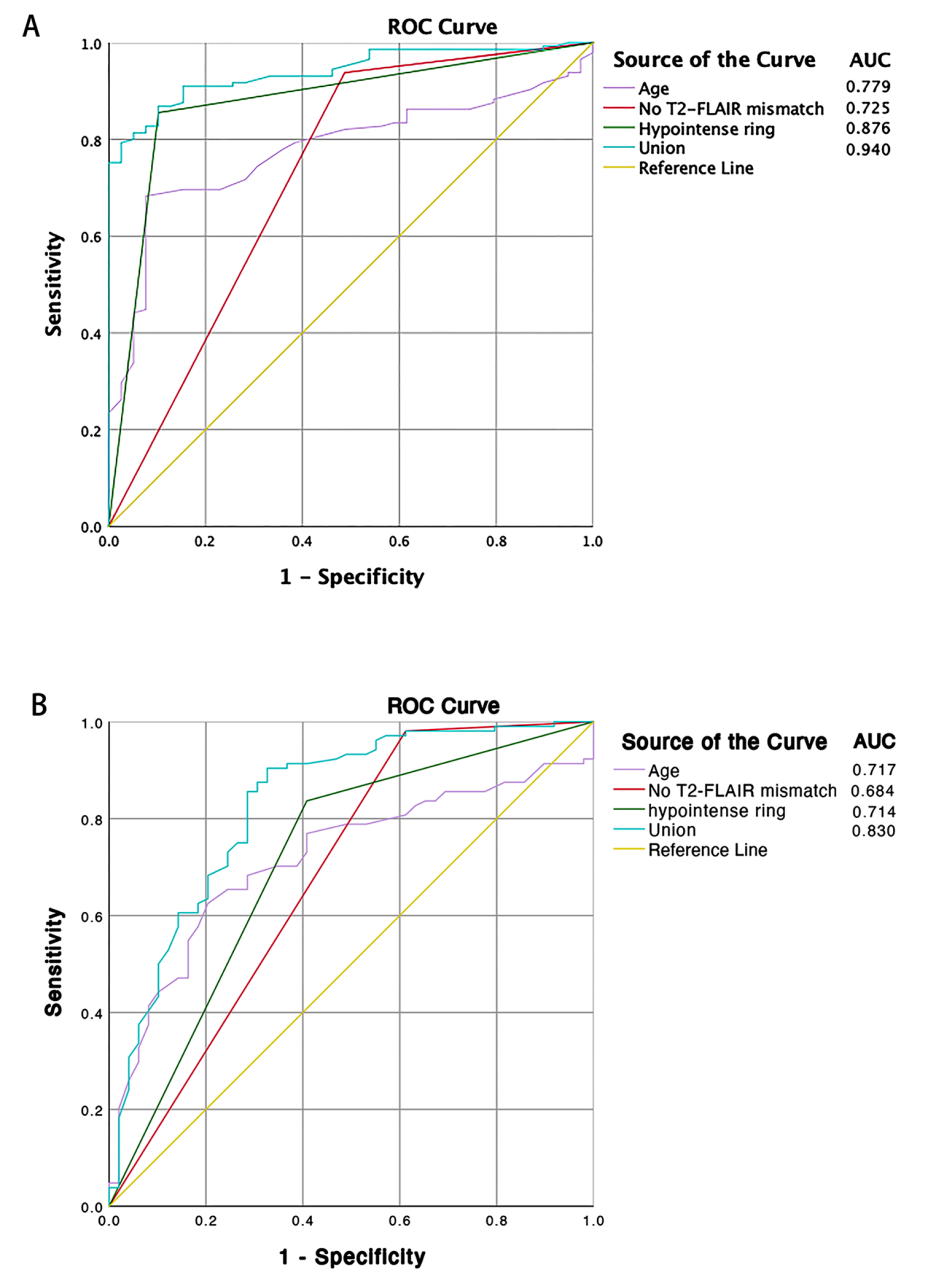
The ROC curves using age、no-T2-FLAIR mismatch、hypointense ring, and union of these factors to predict HGGs **(A)** and IDHwt **(B)**

The enrolled cases were divided into LGGs and HGGs group according to the 2016 WHO grades, and the logistic regression analysis was performed. We found that age, T2W hypointense ring sign, and no-T2-FLAIR mismatch were risk factors for predicting HGGs (Table 2). Then we tested the multivariate logistic regression model in predicting glioma grading by combining all the above significant factors. Compared with a single index, the accuracy of the multivariable model was improved with an AUROC of 0.940 (*p* < 0.001, 95% CI = 0.907–0.972), a sensitivity of 79.3%, specificity of 97.4%, and Youden index of 0.767 in differentiating HGGs from LGGs (Table 2; Fig. 7A).

**Table 2:** Univariable and multivariable logistic regression analysis to predict HGGs

Variables	Univariate Analysis		Multifactor Analysis	
	OR (95%CI)	*p* value	OR (95%CI)	*p* value
Gender (male/female)	0.641 (0.330–1.245)	0.189		
Age	1.080 (1.050–1.112)	**< 0.001**	1.031 (0.989–1.075)	0.147
T2W hypointense ring sign (yes/no)	61.946 (20.263-189.373)	**< 0.001**	30.913 (9.174-104.162)	**< 0.001**
T2-FLAIR mismatch (no-mismatch/ mismatch)^*a*^	15.906 (6.329–39.977)	**< 0.001**	7.599 (2.170-26.616)	**0.002**
ADC of T2W hypointense ring^a^	0.247 (0.001–45.828)	0.600		

^*a*^There were 184 cases with T2W and FLAIR images and 126 cases with ADC images

T2W: T2-weighted; FLAIR: Fluid-attenuated inversion recovery; ADC: apparent diffusion coefficient; OR: Odds ratio; CI: Confidence interval

### Prediction of IDH mutation state by logistic regression analysis

The univariable logistic regression to predict IDHwt results was summarized in Table 3. Several features were statistically significant predictors, including age, T2W hypointense ring, and T2-FLAIR mismatch signs (*p* < 0.001). Gender demonstrated no associations with IDH mutation status. Then three statistically significant factors were included in the multivariate logistic analysis, and the results are listed in Table 3. It showed that the combined prediction efficiency was higher than the single prediction of the three factors, with the AUROC of 0.830 (*p* < 0.001, 95% CI = 0.757–0.904) (Fig. 7B; Table 3), a sensitivity of 90.4%, specificity of 67.3%, and Youden index of 0.577 in differentiating IDHwt from IDHmut.

**Table 3:** Univariable and multivariable logistic regression analysis to predict IDHwt

Variables	Univariate Analysis		Multifactor Analysis	
	OR (95%CI)	*p* value	OR (95%CI)	*p* value
Gender (male/female)	0.547 (0.286–1.049)	0.069		
Age	1.049 (1.024–1.075)	**< 0.001**	1.020 (0.990–1.051)	0.192
T2 hypointense ring (yes/no)	8.266 (3.970-17.208)	**< 0.001**	4.187 (1.703–10.294)	**0.002**
T2-FLAIR mismatch (no-mismatch/mismatch) ^*a*^	32.300 (7.115-146.626)	**< 0.001**	18.213 (3.766–88.066)	**< 0.001**
ADC of Hypointense ring	0.313 (0.020–4.998)	0.411		

^*a*^There were 153 cases with IDH genotype detections and T2-FLAIR images concurrently and 105 cases with IDH genotype detections and ADC images

FLAIR: Fluid-attenuated inversion recovery; ADC: Apparent diffusion coefficient; OR: Odds ratio; CI: Confidence interval

## Discussion

In this study, we primarily evaluated two imaging signs on MRI, T2W hypointense ring and T2-FLAIR mismatch, which have significant value in distinguish LGGs and HGGs, together with IDH mutation status. The assessment of these two signs demonstrated a high consistency among readers (κ = 0.85 and 0.84, respectively). In addition, we constructed multivariate models to predict HGGs and IDHwt using T2W hypointense ring, no-T2-FLAIR mismatch and age. Both models, based on the combination of multiple parameters, displayed excellent prediction ability and outperformed models using a single parameter.

The appearance of the T2W hypointense ring of glioma might vary between different patients, appearing as a thin or thick ring, intact or incomplete loops, just like a superior or inferior arc. Schwartz et al. reported that 69% of gliomas had hypointense borders, 26% of which were rims, and 74% were arcs [18]. Our findings of T2W hypointense ring in gliomas were consistent with their results (69.08%). But the exact causes of this sign with various shapes should be further investigated pathologically. As highly heterogeneous tumors, the extensive genetic variations and microenvironmental biochemistry of gliomas are the underlying causes of the different MRI presentations. It is generally recognized in WHO Grade II and III glioma subtypes (astrocytoma and oligodendroglioma) and secondary glioblastoma, while IDH mutation is not found in any pilocytic astrocytomas of WHO grade I. This indicates that these tumors occur through different mechanisms, and pilocytic astrocytoma rarely undergoes malignant transformation [20, 21]. In this study, in order to eliminate its influence, pilocytic astrocytomas (WHO Grade I) were not included.

When Schwartz et al. compared the position of the enhancing ring on T1WI with the hypointense borders (rims or arcs) on T2WI, they found that the T2 border of most gliomas only partially corresponded to the hyperintense ring [18]. The reason may be that in all intracranial lesions, the circular enhancement represents blood-brain barrier (BBB) destruction to varying degrees, while the T2W hypointense ring may originate from the tumor itself, compressed white matter, granulation tissue, and paramagnetic free radicals produced by macrophages [22]. During clinical work, some patients cannot undergo enhanced MRI examination because of the contraindications, such as severe renal insufficiency, allergic constitution, asthma, etc. In this instance, if we can preliminarily judge the grade of glioma and IDH mutation status based on routine MRI images without contrast agents, it will be an excellent direction for patient treatment and prognosis. In this study, some HGGs showed no enhancement after injection of contrast agent and some gliomas with rosette enhancement were not completely consistent with the T2W hypointense ring, indicating that the T2W hypointense ring of gliomas might be an imaging indicator independent of the enhanced state, with clinical application value.

To our knowledge, there were few studies on evaluating the T2W hypointense ring sign in MRI of glioma to date. This sign is generally located between the parenchyma of the tumor and the edema. We speculate that it may be an indicator of tumor glial hyperplasia and invasion into the peripheral normal brain tissues. At present, the radiological definition of the tumor margins in clinical practice is usually determined by comparing the boundary of enhanced lesions on post-gadolinium T1W images [23, 24]. However, previous studies had found that tumor cells usually existed in peritumoral edema of glioblastoma multiforme, and the actual tumor edge could extend several centimeters beyond the edge of tumor parenchyma detected by microscope image analysis [25]. This study couldn’t directly and intuitively judge the boundary of glioma invasion, but if the T2W hypointense ring was confirmed as tumor glial hyperplasia, it might be possible to use this sign to predict whether glioma was invading around and the direction of invasion.

Another possibility is that the deposition of hemosiderin accounted for the formation of the T2W hypointense ring. A previous study revealed that hypointense rings in necrotic glioblastoma were incomplete, irregular, and commonly found at the inner aspect of the contrast-enhanced border [26]. This study speculated the hypointense rings in glioblastoma might resulted from the random accumulation of hemorrhage products at the edge of the necrotic cavity [26]. Schwartz et al. also reported that T2W hypointense ring sign might correspond to paramagnetic free radicals produced by macrophages, hemosiderin-containing macrophages, or fiber edges [18]. Glioblastoma is a highly invasive tumor with blood vessels rapidly generated to support tumor cells growth. Compared to normal capillaries, these neovasculatures have immature vessel walls and wider endothelial spaces, which determine that the intravascular fluid is more likely to leak outside the vessels, leading to peritumoral edema and hemorrhage [27]. In our study, most gliomas with T2W hypointense ring signs were highly invasive HGGs that could easily invade and cause rupture and bleeding of abundant neovasculatures, subsequently leading to hemosiderin deposition.

The ADC value is negatively correlated with cell proliferation indices [28]. Previous studies confirmed that compared with LGGs, HGGs had a lower ADC value [10, 29]. In this study, we also attempted to analyze the ADC values of the T2W hypointense ring. The results showed that compared with LGGs, HGGs had lower ADC values of the T2W hypointense ring. However, this finding did not reach a significant statistical difference, which might be owing to the small sample size of LGGs with T2W hypointense rings (*n* = 4). Moreover, if hemosiderin deposition existed within the T2W hypointense ring, this might lead to a large deviation of ADC values due to magnetic susceptibility. On the other hand, most of the T2W hypointense rings in our cases were thin and some rings showed uneven signals, which made it a great challenge to accurately draw ROIs for ADC measurement.

Recently, studies had reported on the signs of T2-FLAIR mismatch [15–17, 19, 30]. Patel et al. investigated the T2-FLAIR mismatch marker in two groups of LGG data sets and found this sign could identify IDHmut-Noncodel glioma in both sets with a PPV of 100%, but its NPV was only 54% and 76% [17]. This was further confirmed by the research of Broen et al. [16]. Another meta-analysis, including 12 studies and 1053 patients, had a combination of a specificity of 100%, but a sensitivity of only 42% [30]. At present, the pathophysiological basis of the T2-FLAIR mismatch sign remains unclear. Patel et al. also showed that in IDHmut-Noncodel gliomas with T2-FLAIR mismatch, the protein level in the mTOR pathway was significantly increased [17]. Another possible explanation was that the T2-FLAIR mismatch sign might reflect tumor cell structure [30]. Unlike previous studies, we focused on the “no-T2-FLAIR mismatch” sign to predict HGGs and IDHwt gliomas, and the results were statistically significant.

The following are the limitations of this study. This was a single-center retrospective study and the sample size of patients with LGGs was relatively small. To further validate our findings, a multicenter prospective study should be conducted in the future. Additionally, the nature of the T2W hypointense ring in glioma should be clarified in the future.

## Conclusions

This study found that the T2W hypointense ring of the gliomas could noninvasively predict high-grade and IDHwt gliomas, and the multivariate logistic regression model constructed by combining T2W hypointense ring, no-T2-FLAIR mismatch and age was found to be superior to models based on a single factor. Overall, our study demonstrated the utility of routine MR imaging features to distinguish glioma grade and IDH genotypes, and if validated, our approach would be particularly beneficial for patients who cannot tolerate invasive biopsy, and contrast enhancement or those who cannot afford the gene tests.

### Electronic supplementary material

Below is the link to the electronic supplementary material.

Supplementary Material 1

Supplementary Material 2

Supplementary Material 3

Supplementary Material 4

Supplementary Material 5

Supplementary Material 6

## Data Availability

The dataset analysed during the current study are available from the corresponding author on reasonable request.

## Abbreviations

IDH: Isocitrate dehydrogenase
LGGs: Low-grade gliomas
HGGs: High-grade gliomas
ROI: Region of interest
AUC: Area under the curve
PPV: Positive predictive value
NPV: Negative predictive value
BBB: Blood-brain barrier
T2WI: T2-weighted images
FLAIR: Fluid-attenuated inversion recovery
DWI: Diffusion-weighted imaging
ADC: Apparent diffusion coefficient
