# Supplementary material for: Identification of T2W hypointense ring as a novel noninvasive indicator for glioma grade and IDH genotype
Source: Cancer Imaging. 2024 Jun 28;24:80. doi: 10.1186/s40644-024-00726-3 (PMC11212435; doi:10.1186/s40644-024-00726-3)
Supplement: Supplementary file 2 — Supplementary Material 2 [file 40644_2024_726_MOESM2_ESM.pdf]

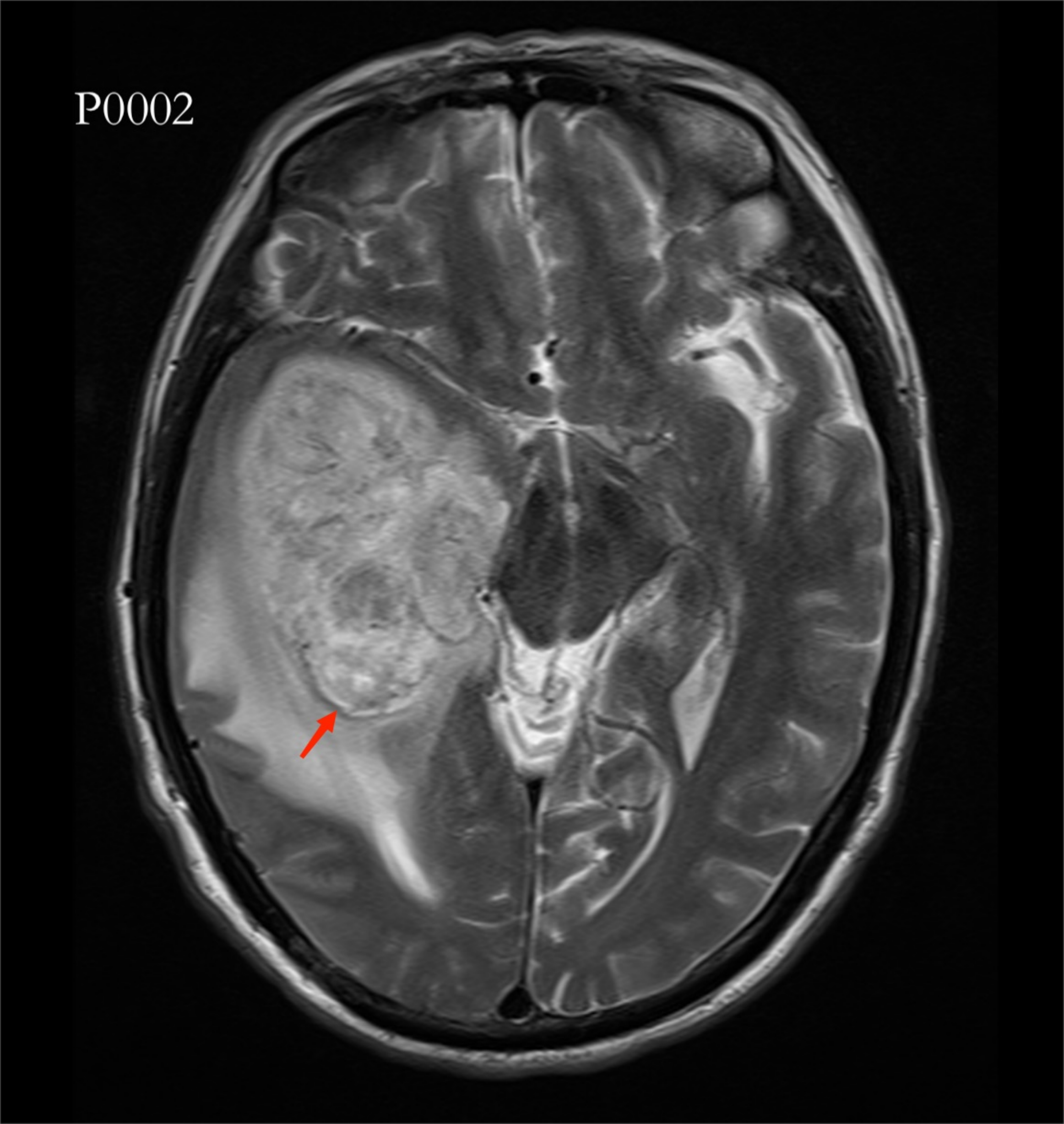
P0002

P0003

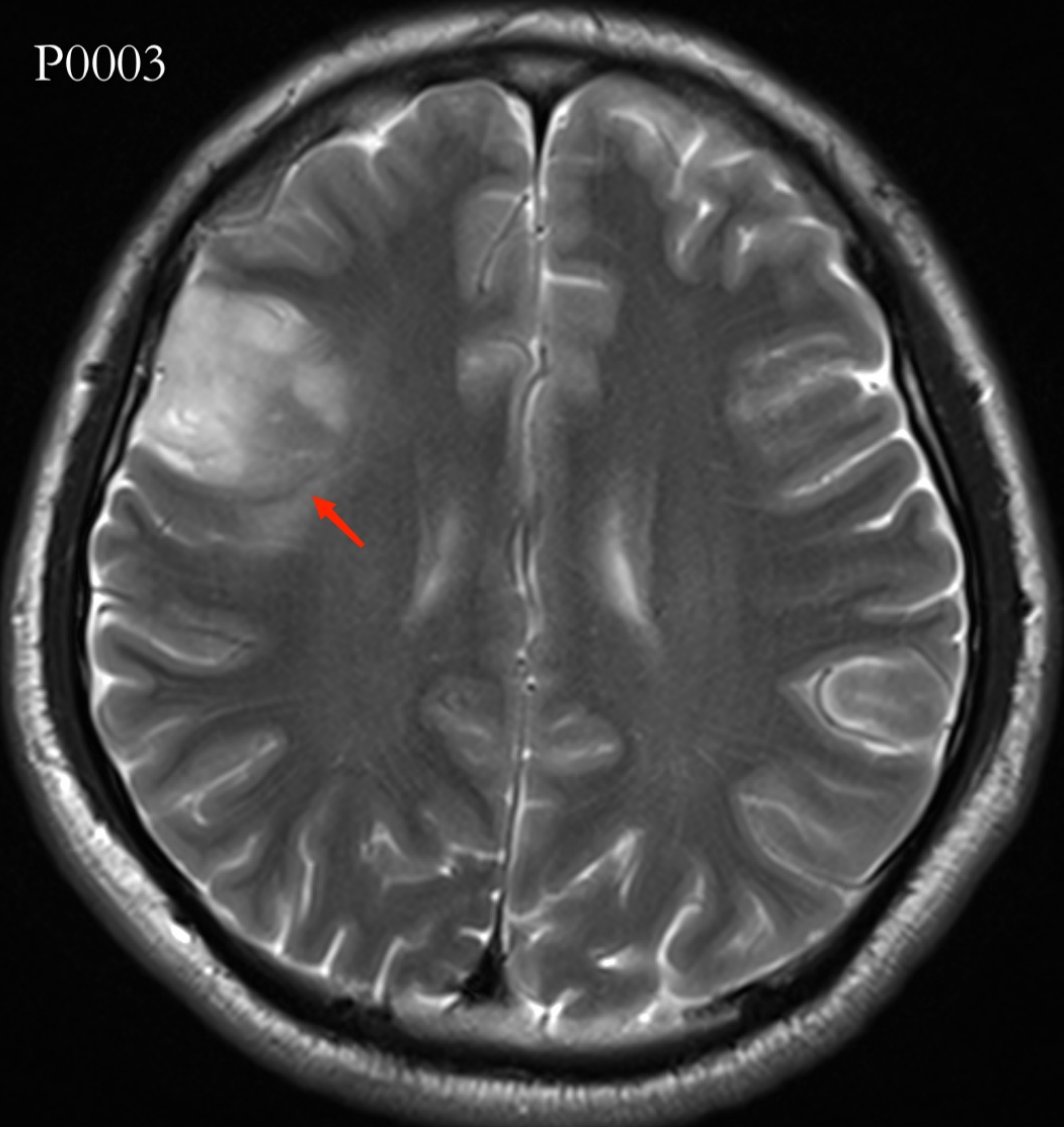

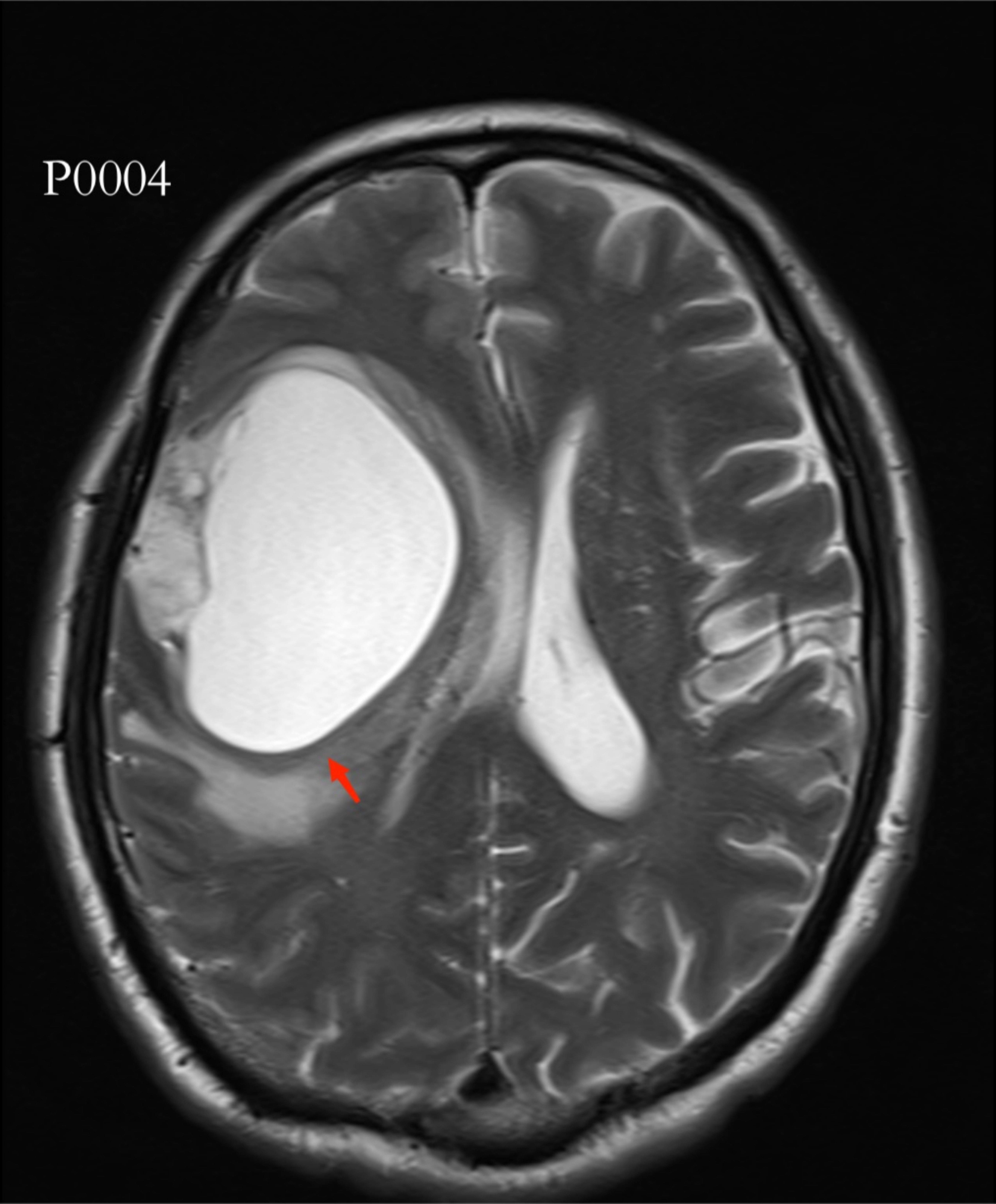
P0004

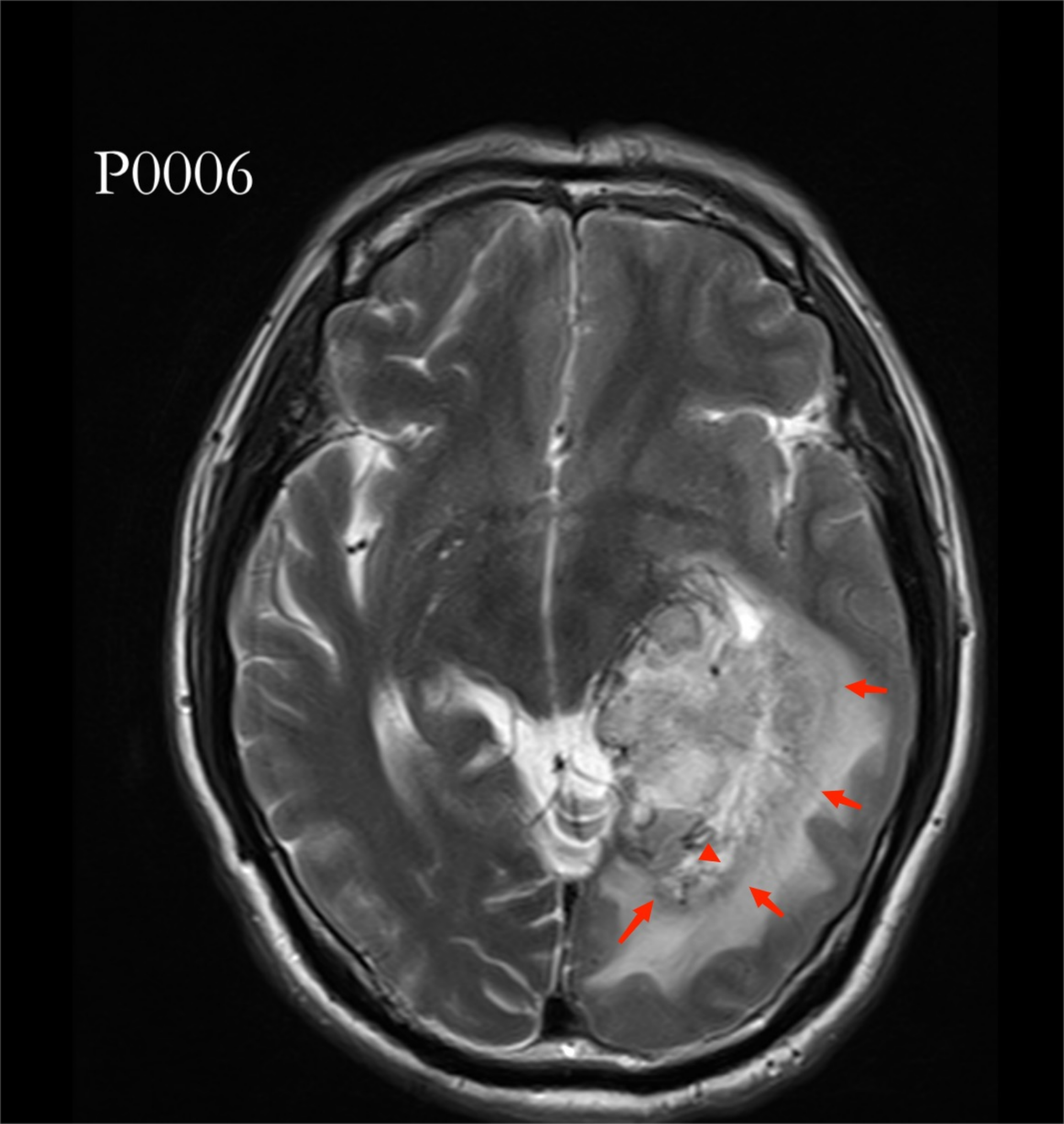
P0006

P0007

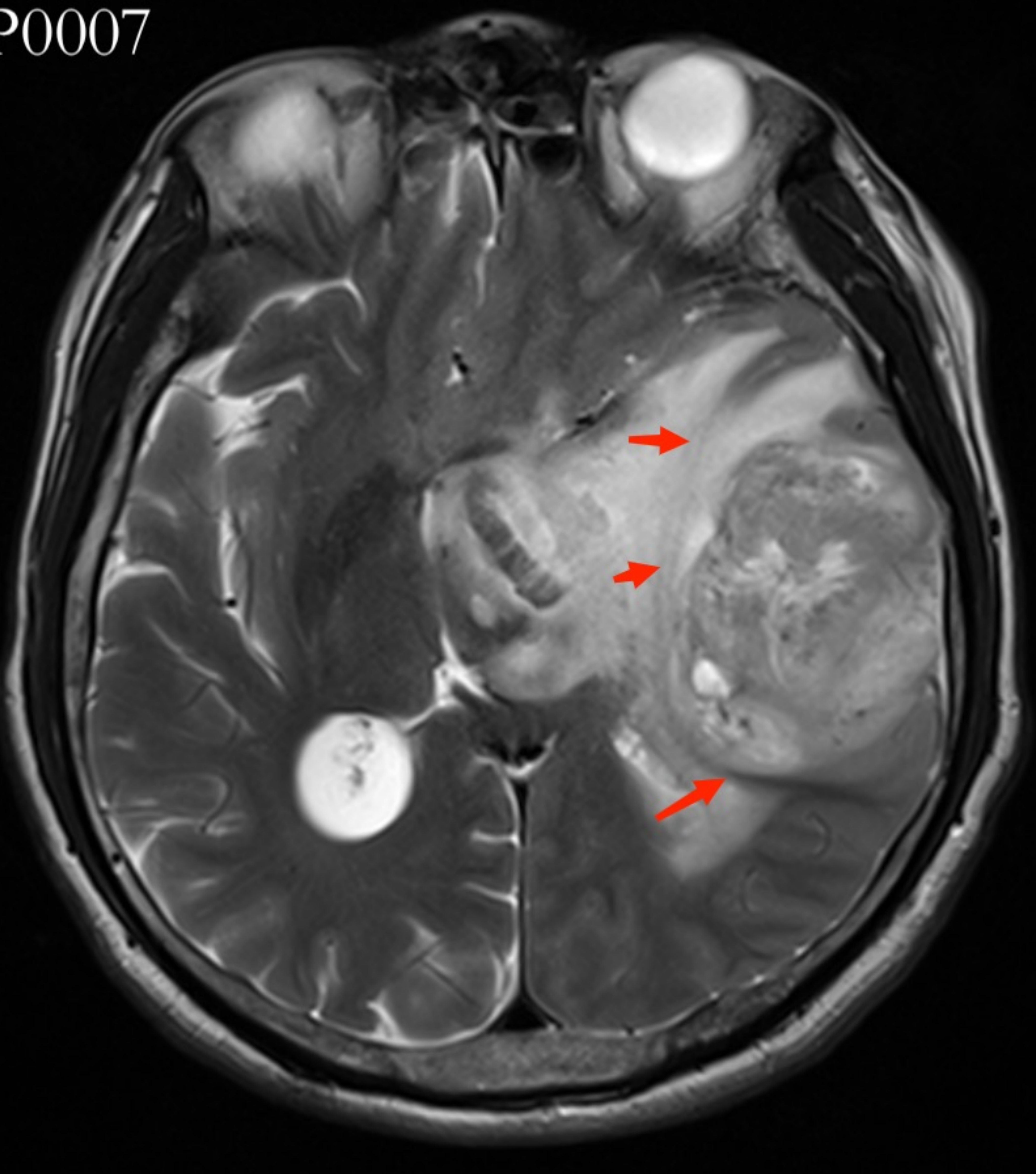

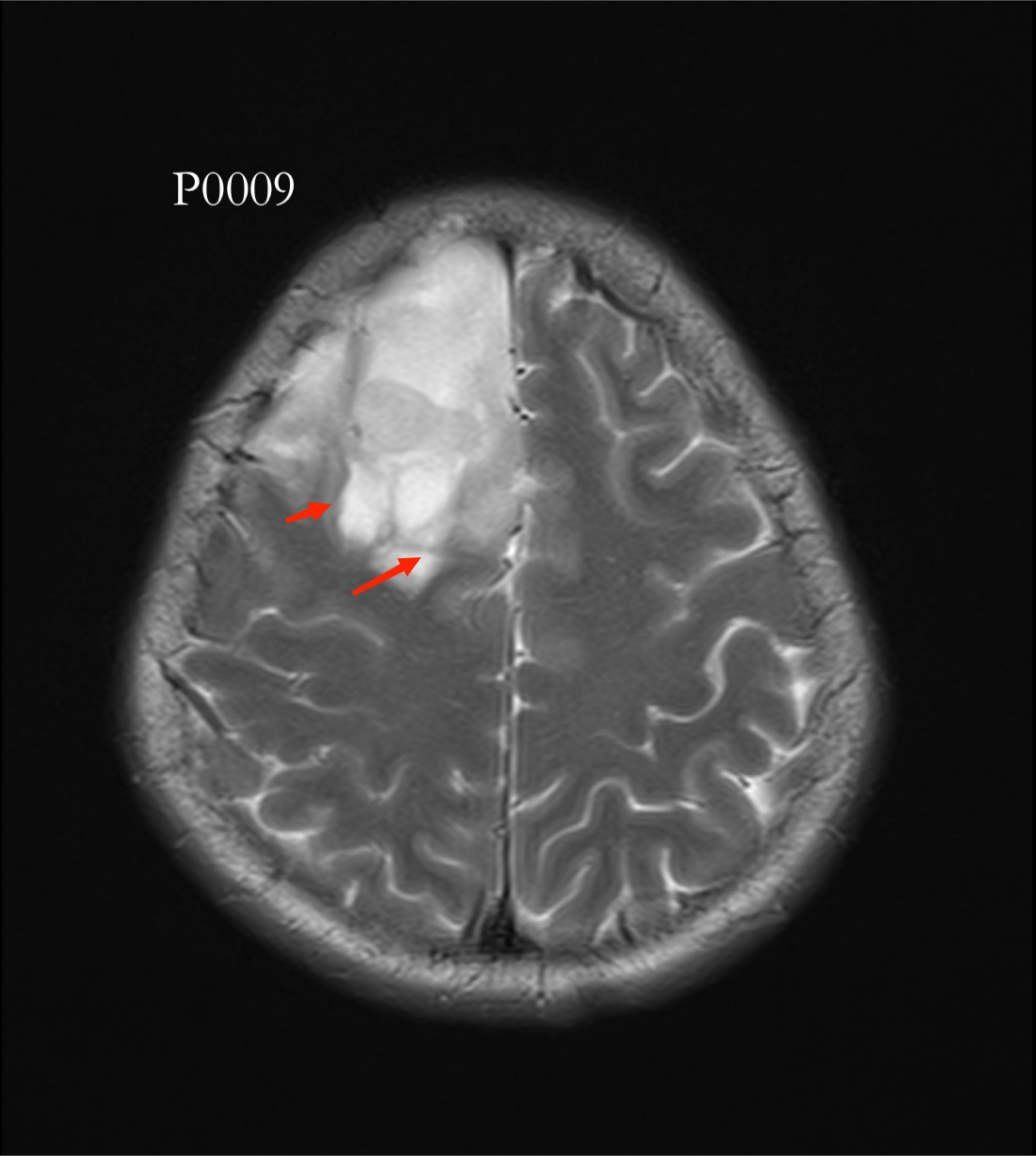
P0009

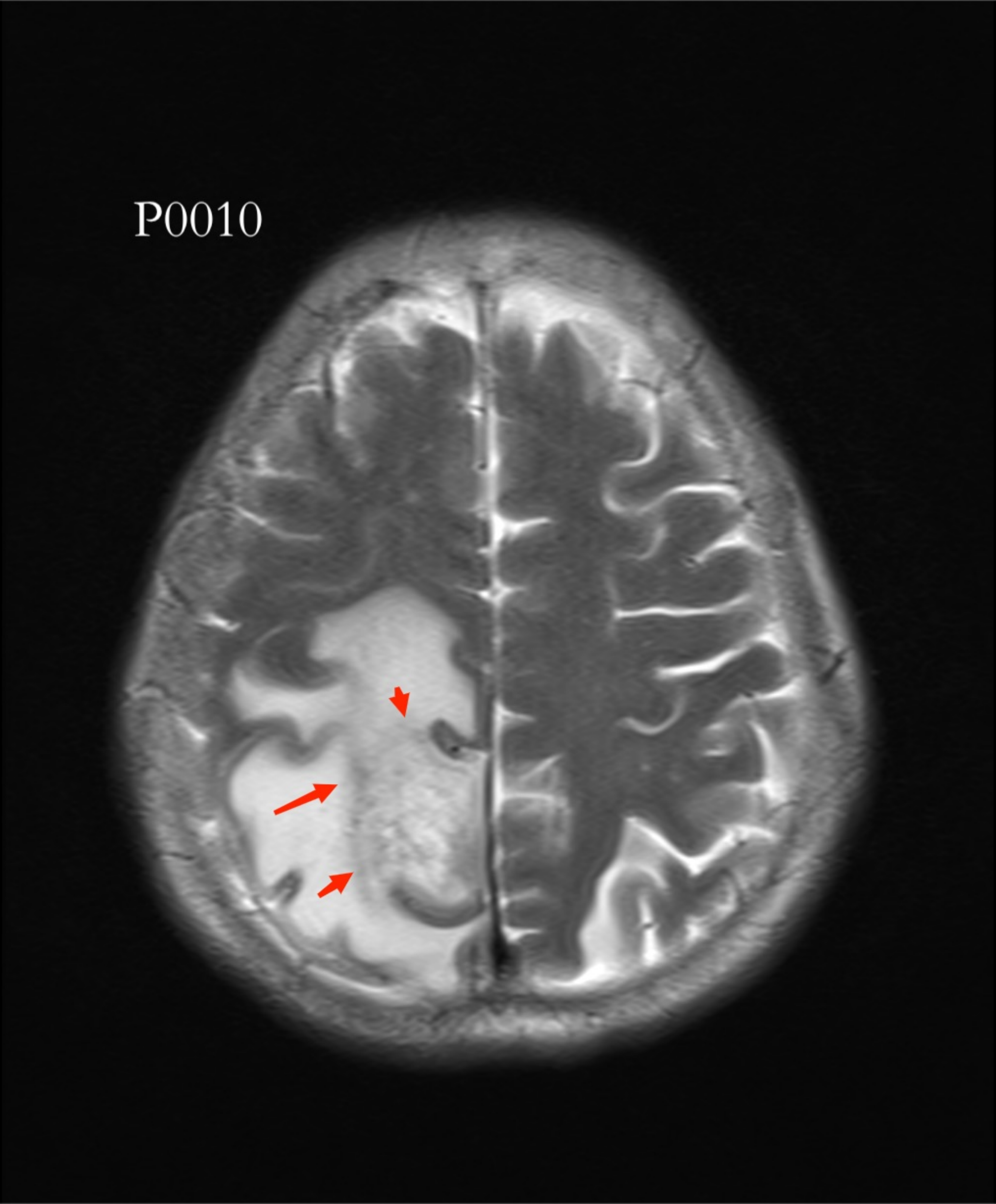
P0010

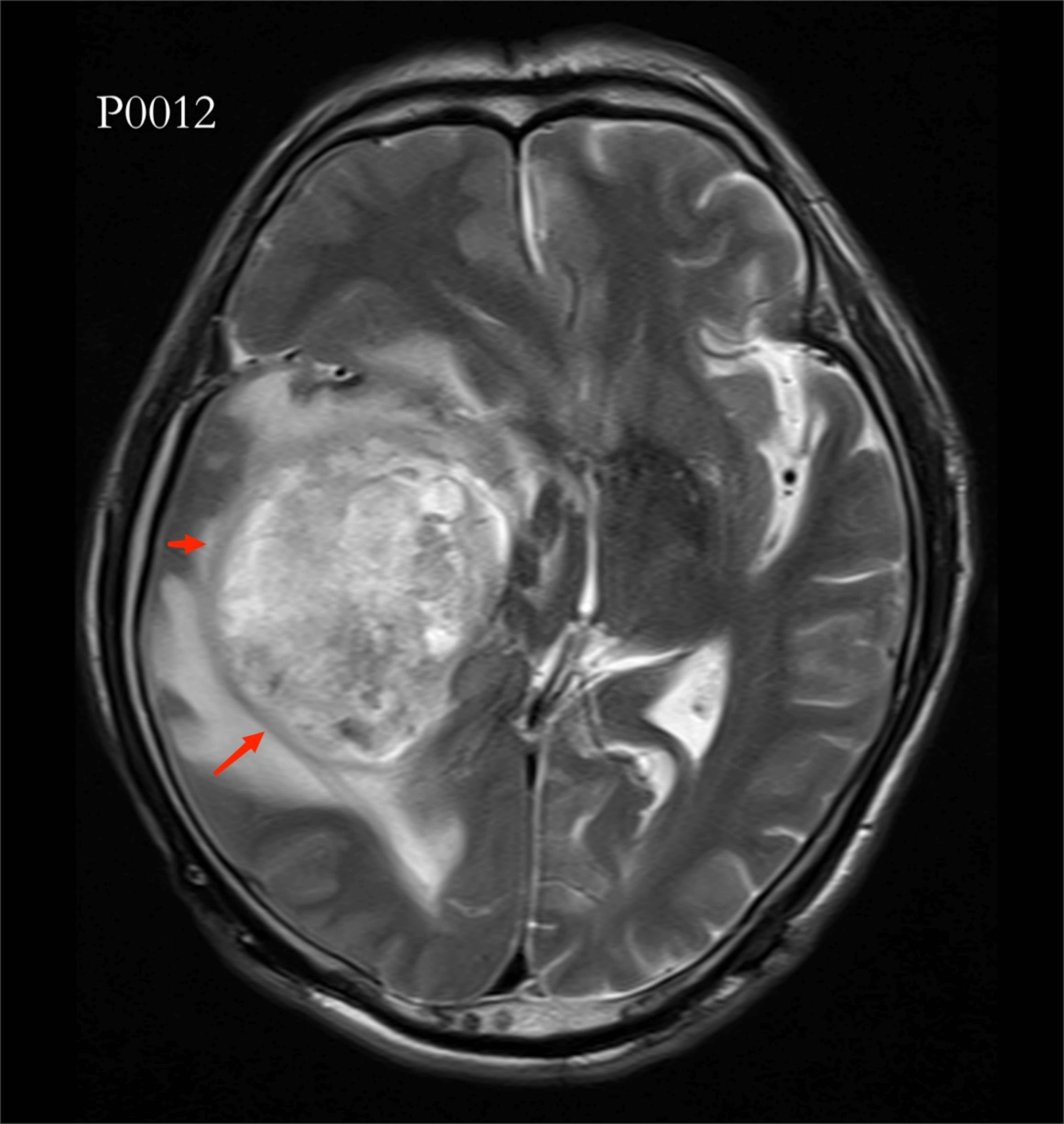
P0012

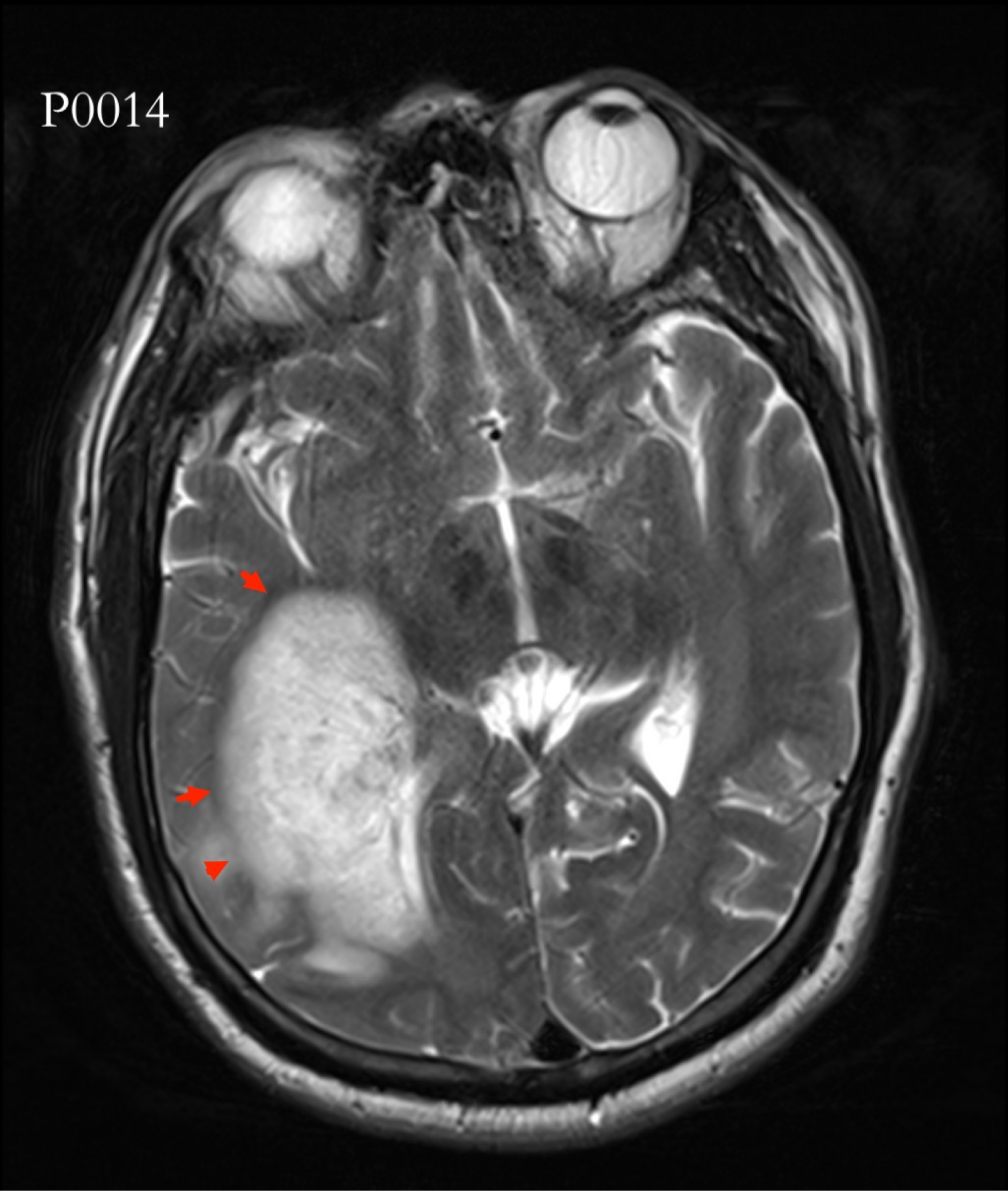
P0014

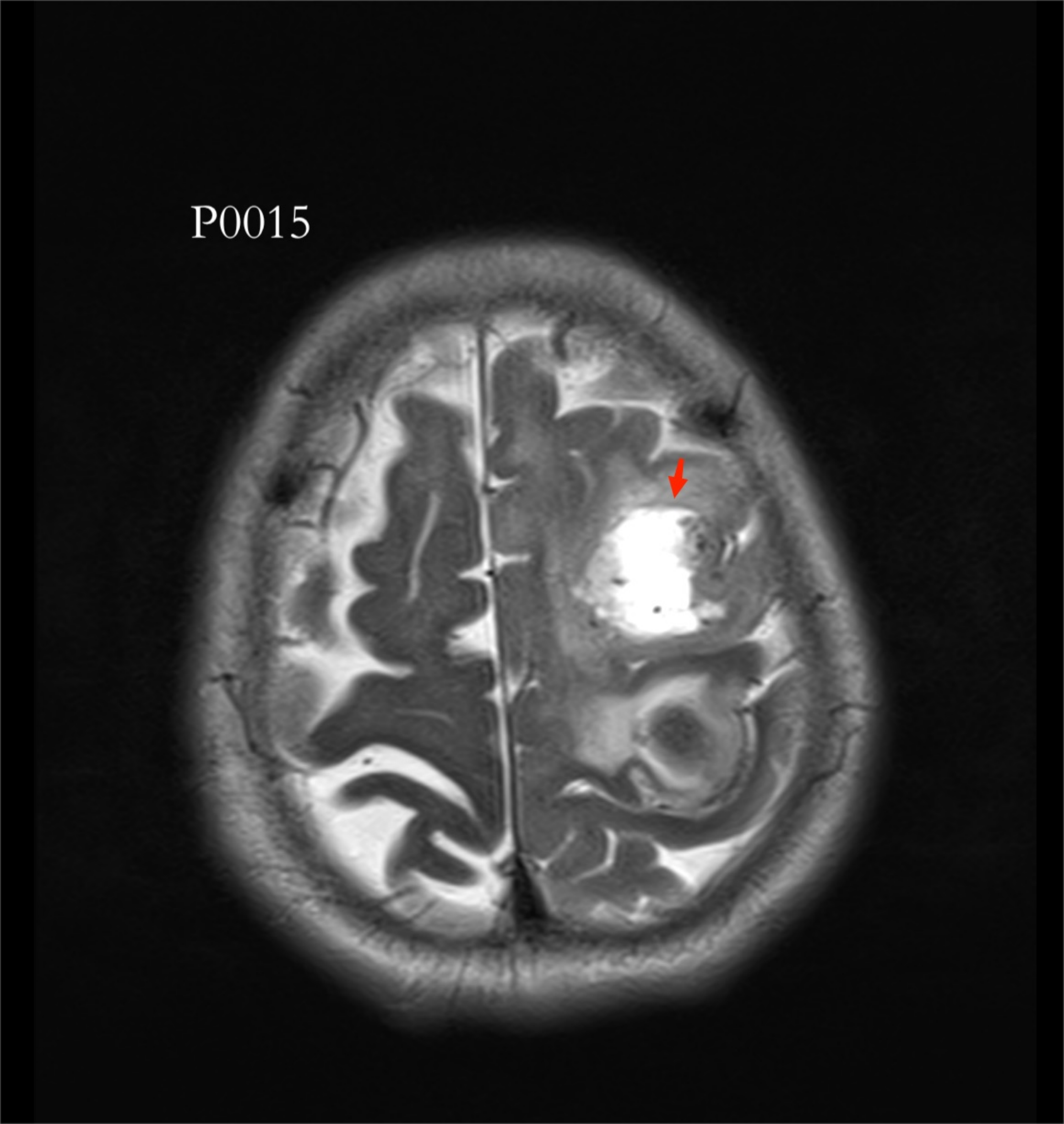
P0015

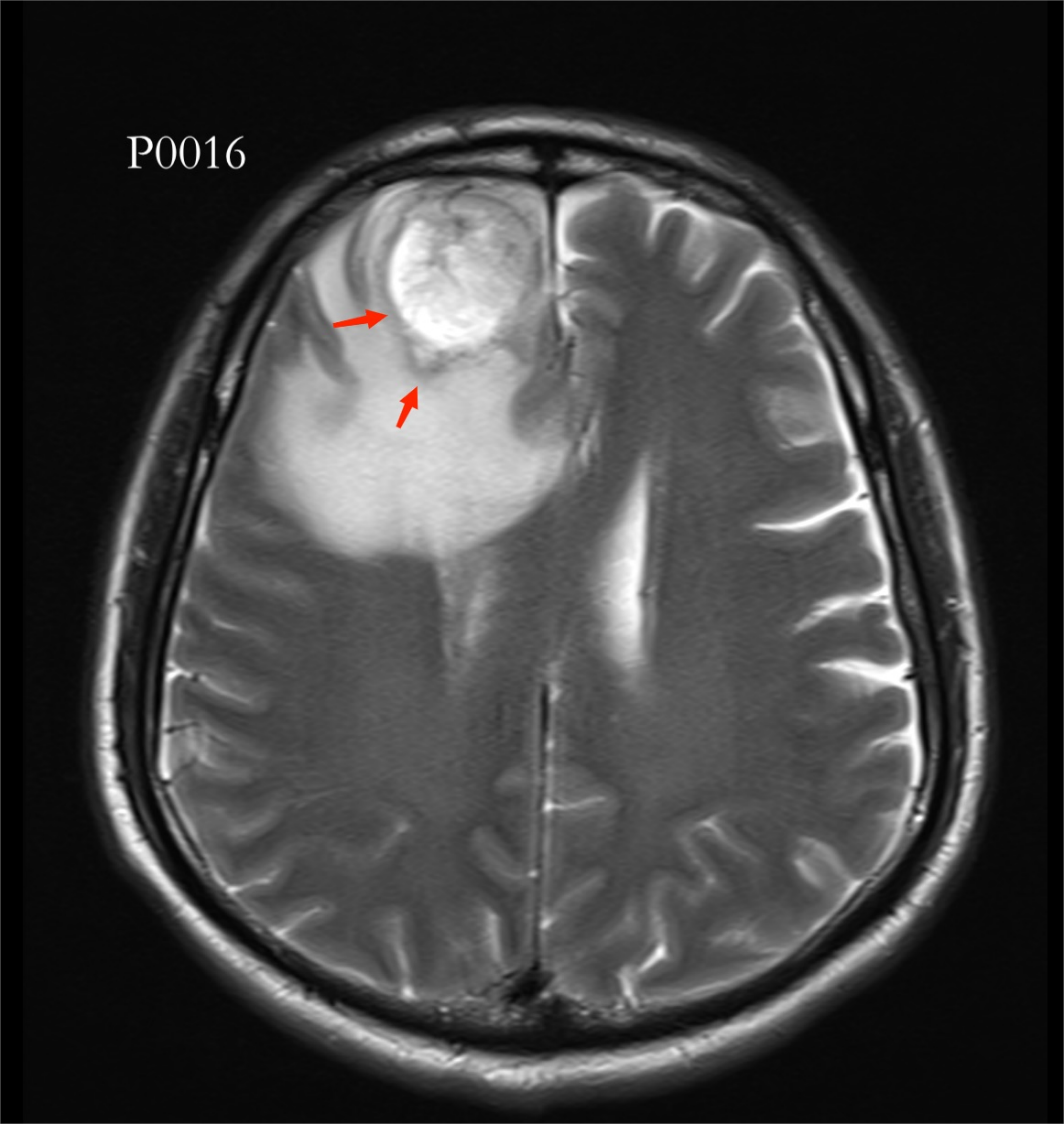
P0016

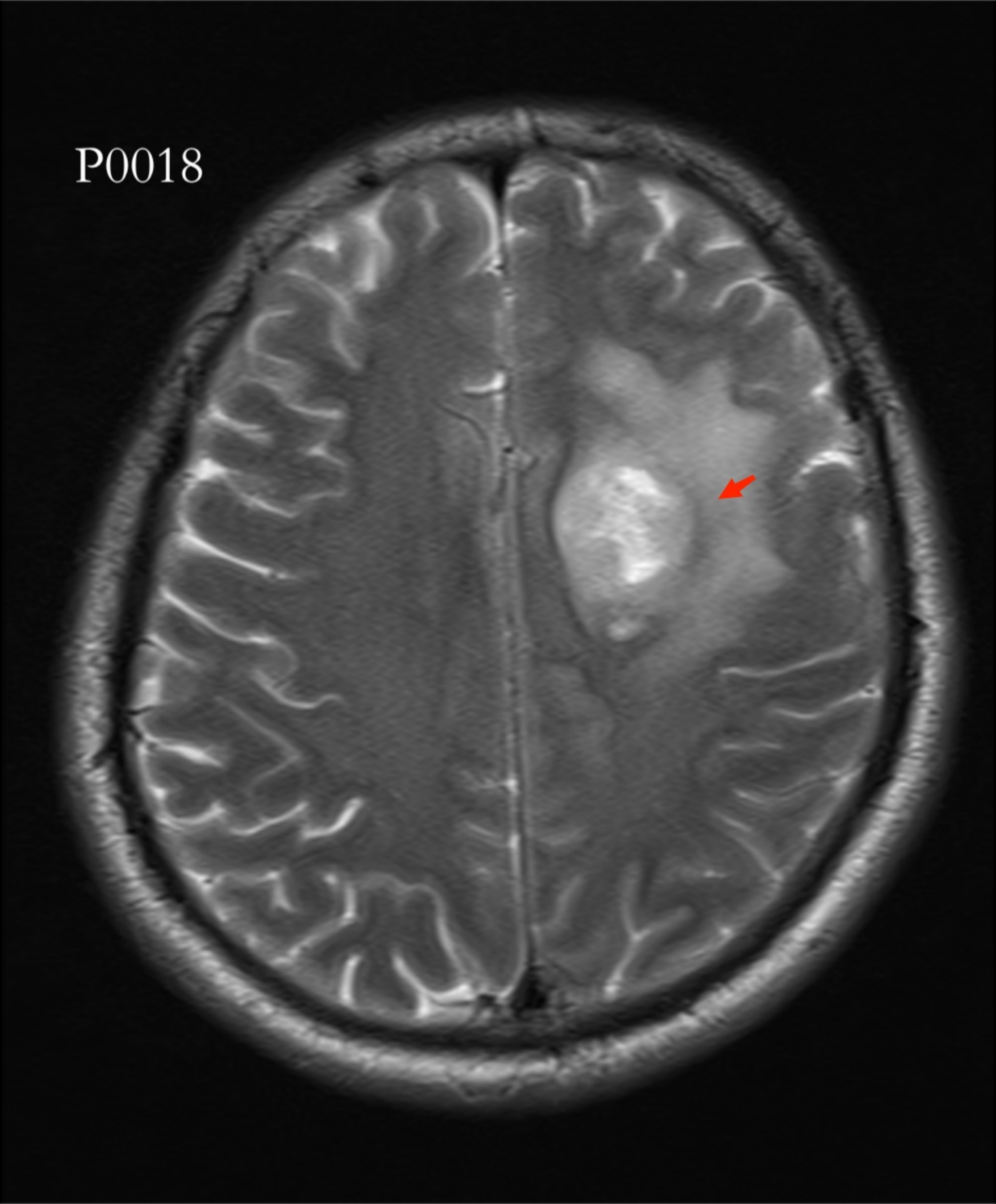
P0018

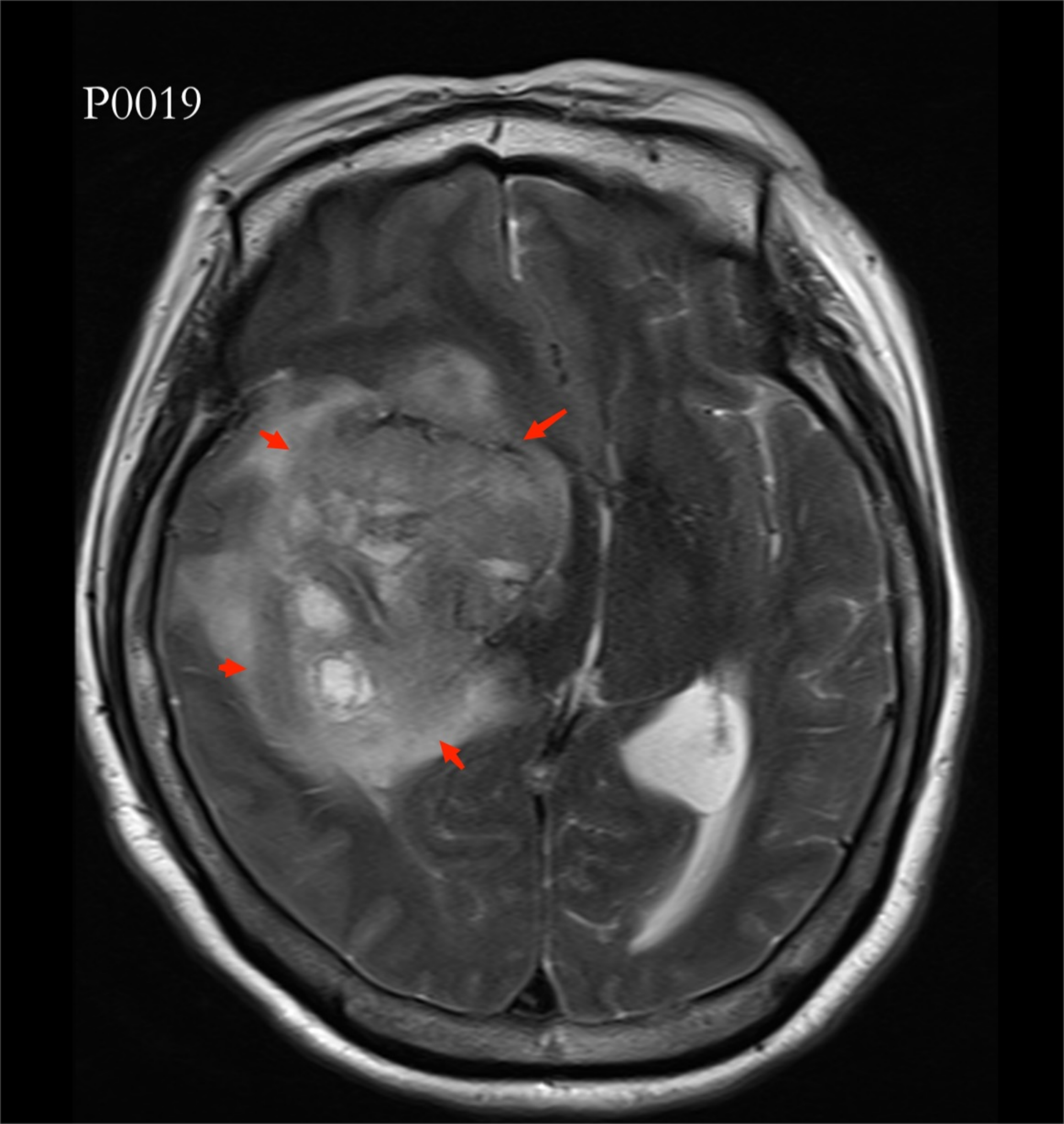
P0019

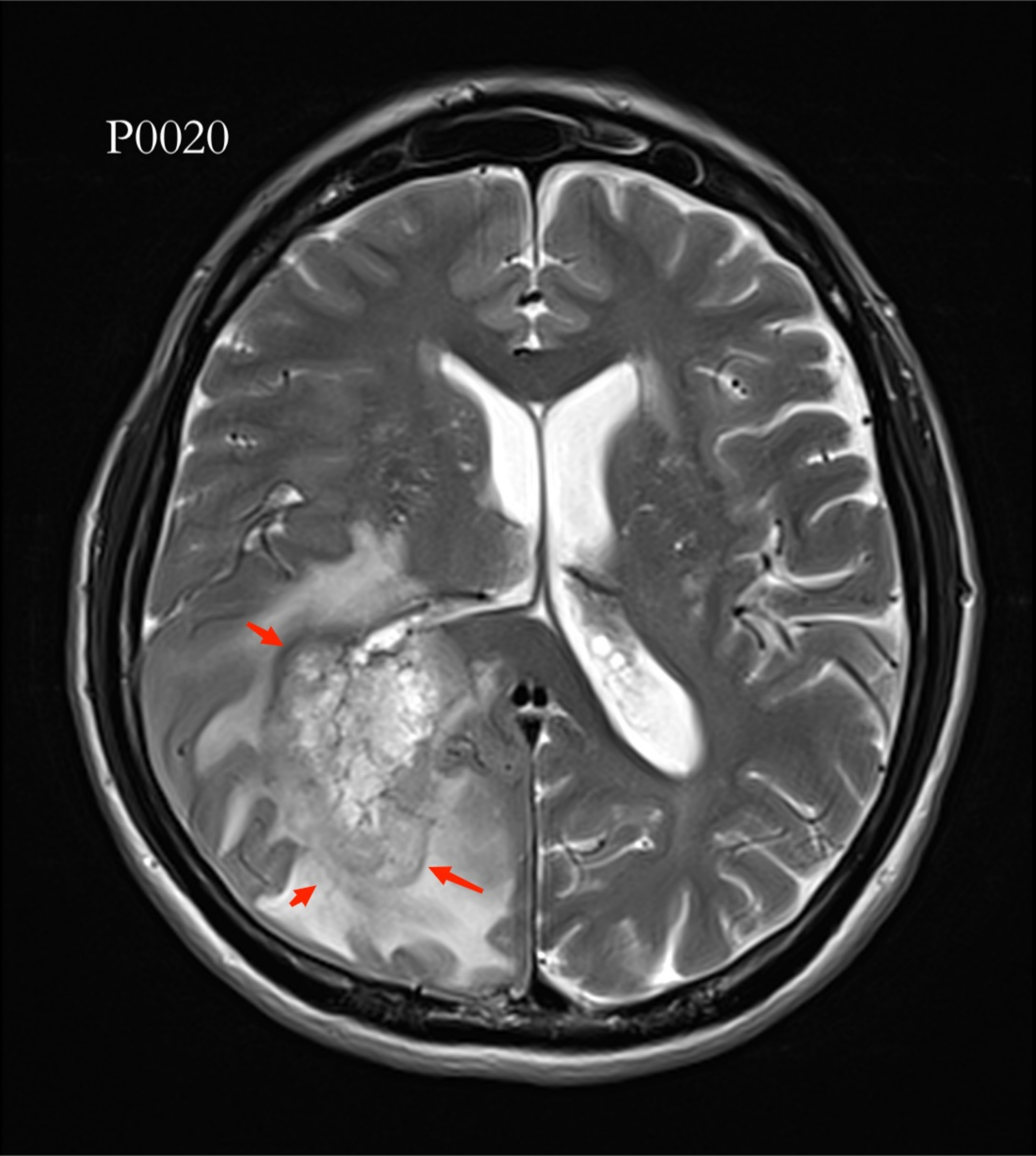
P0020

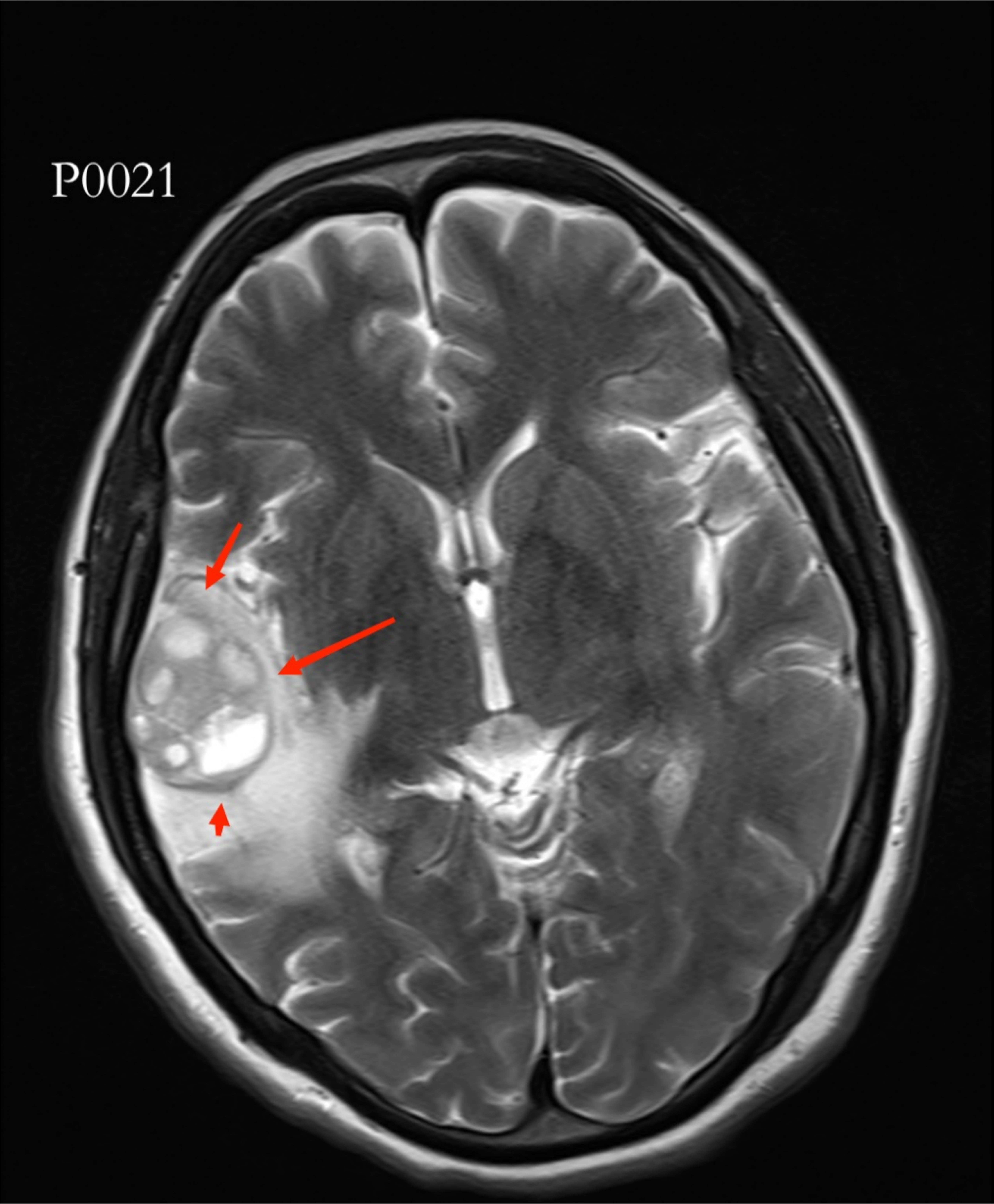
P0021

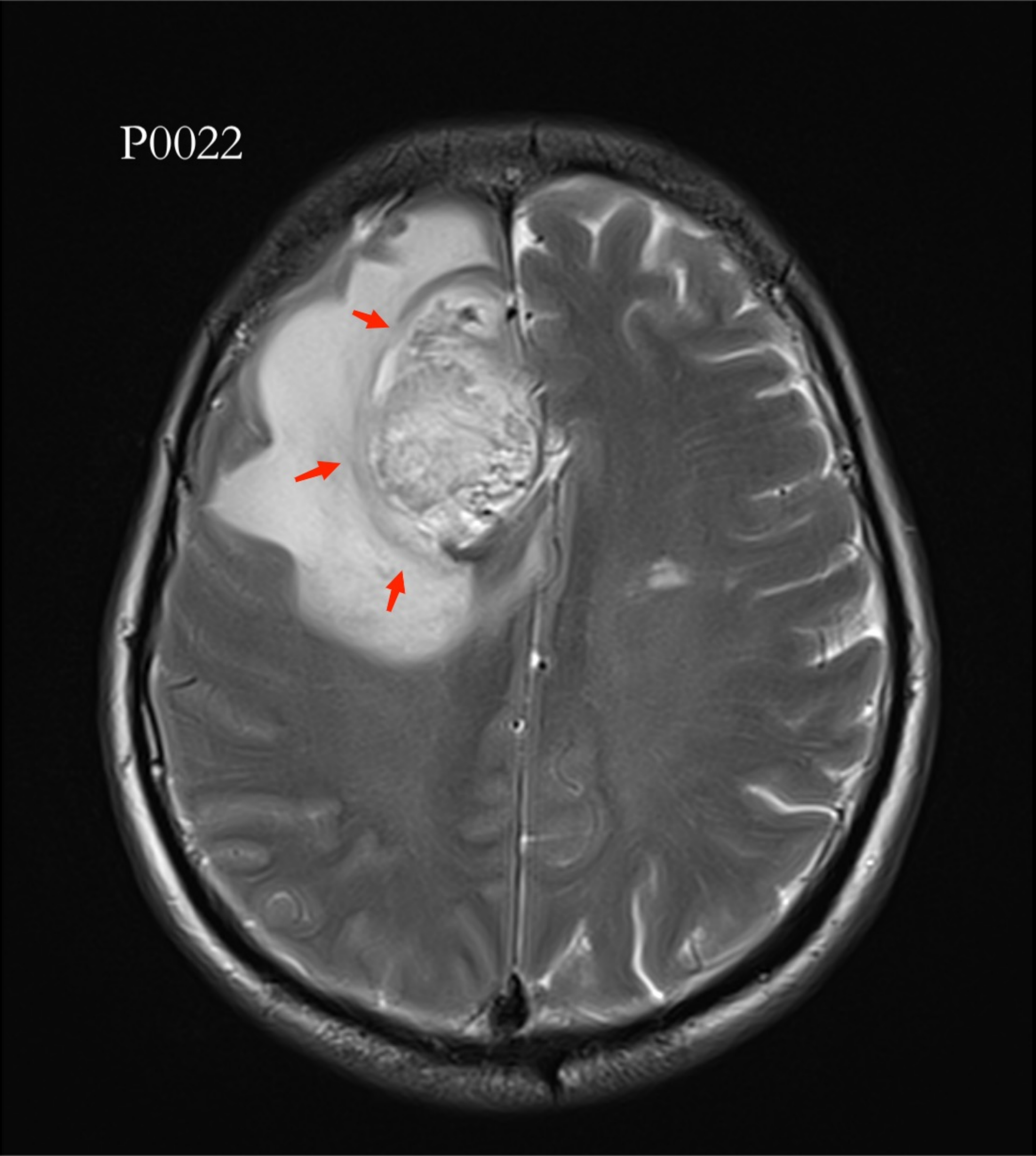
P0022

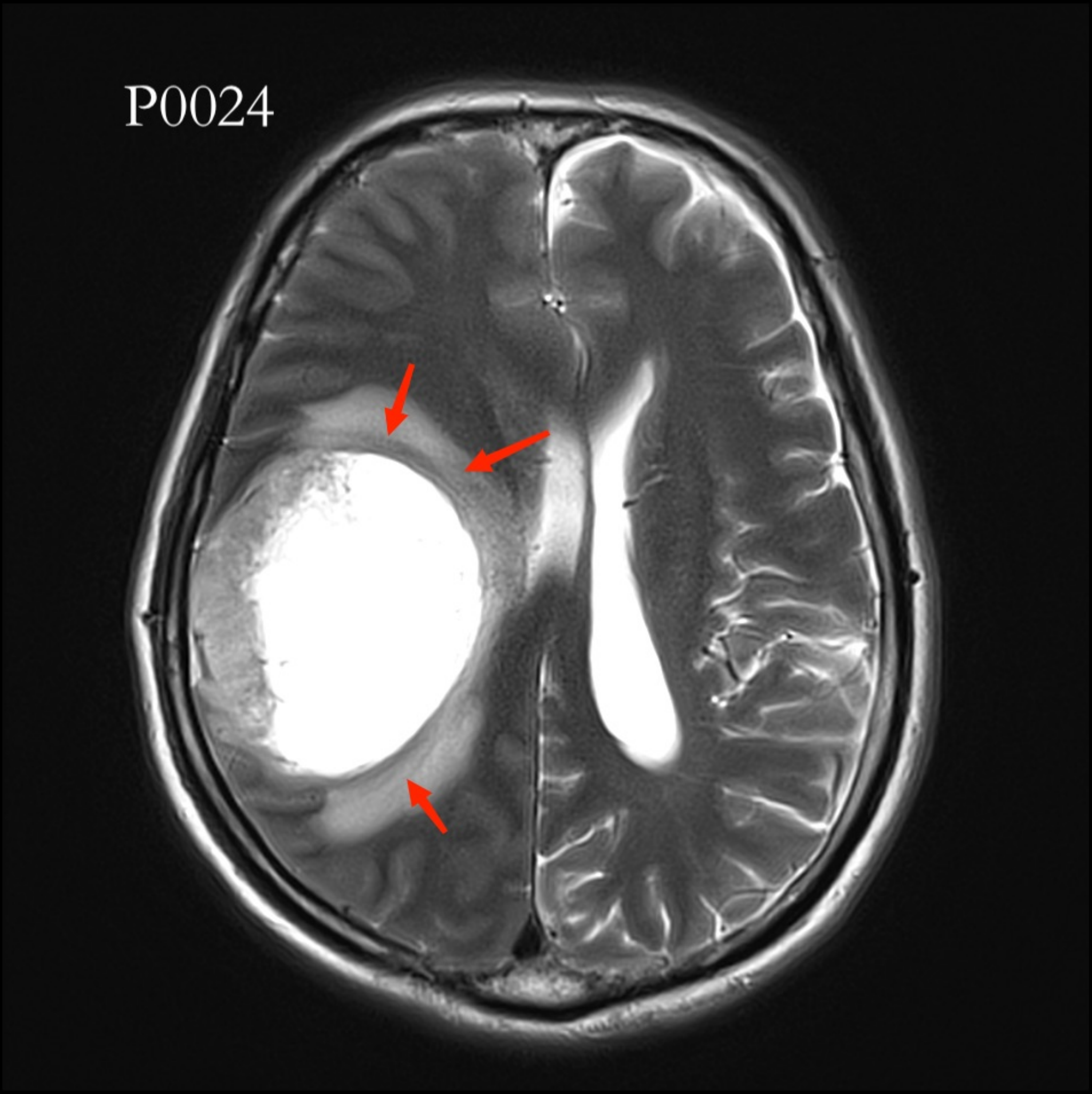
P0024

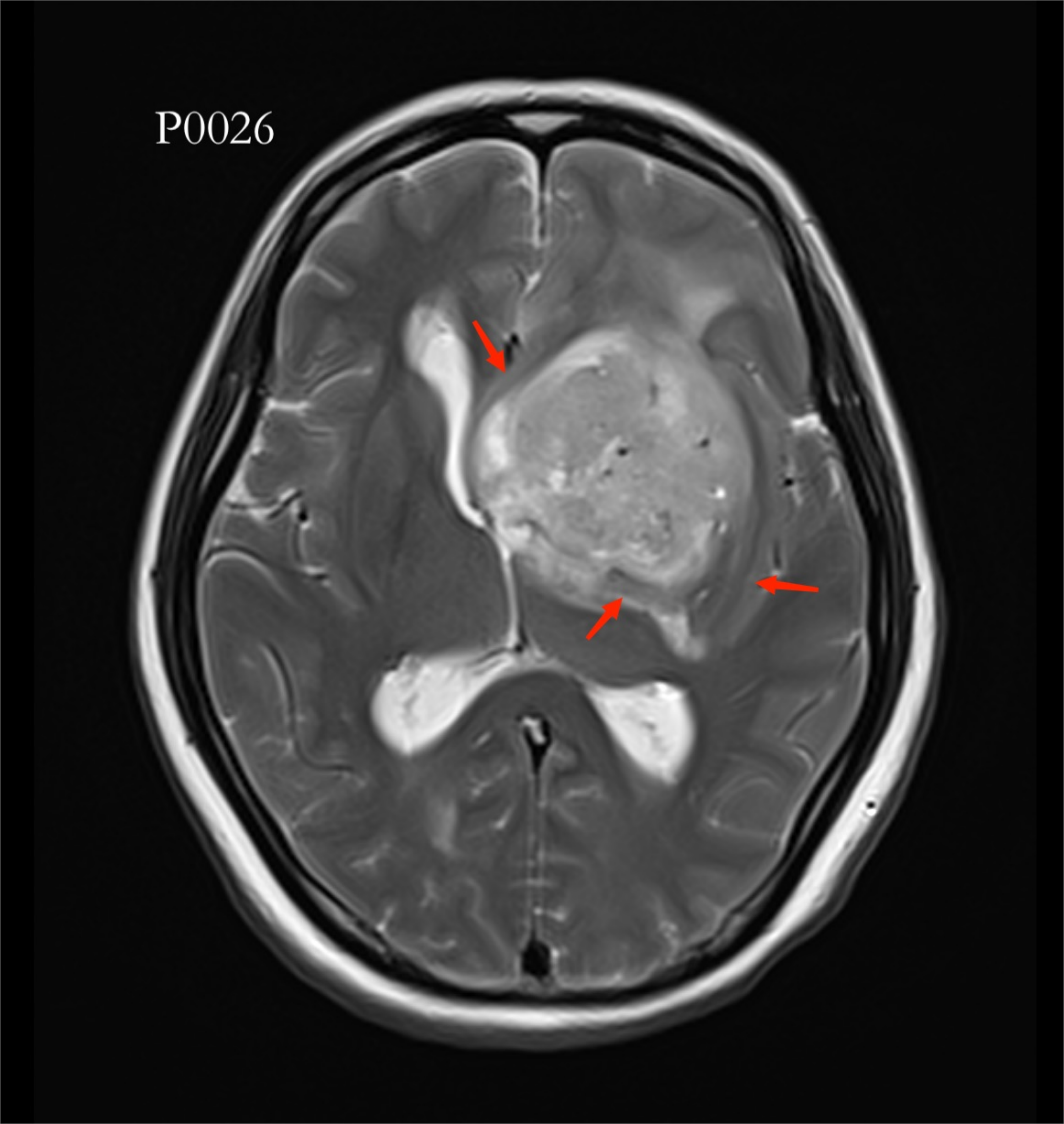
P0026

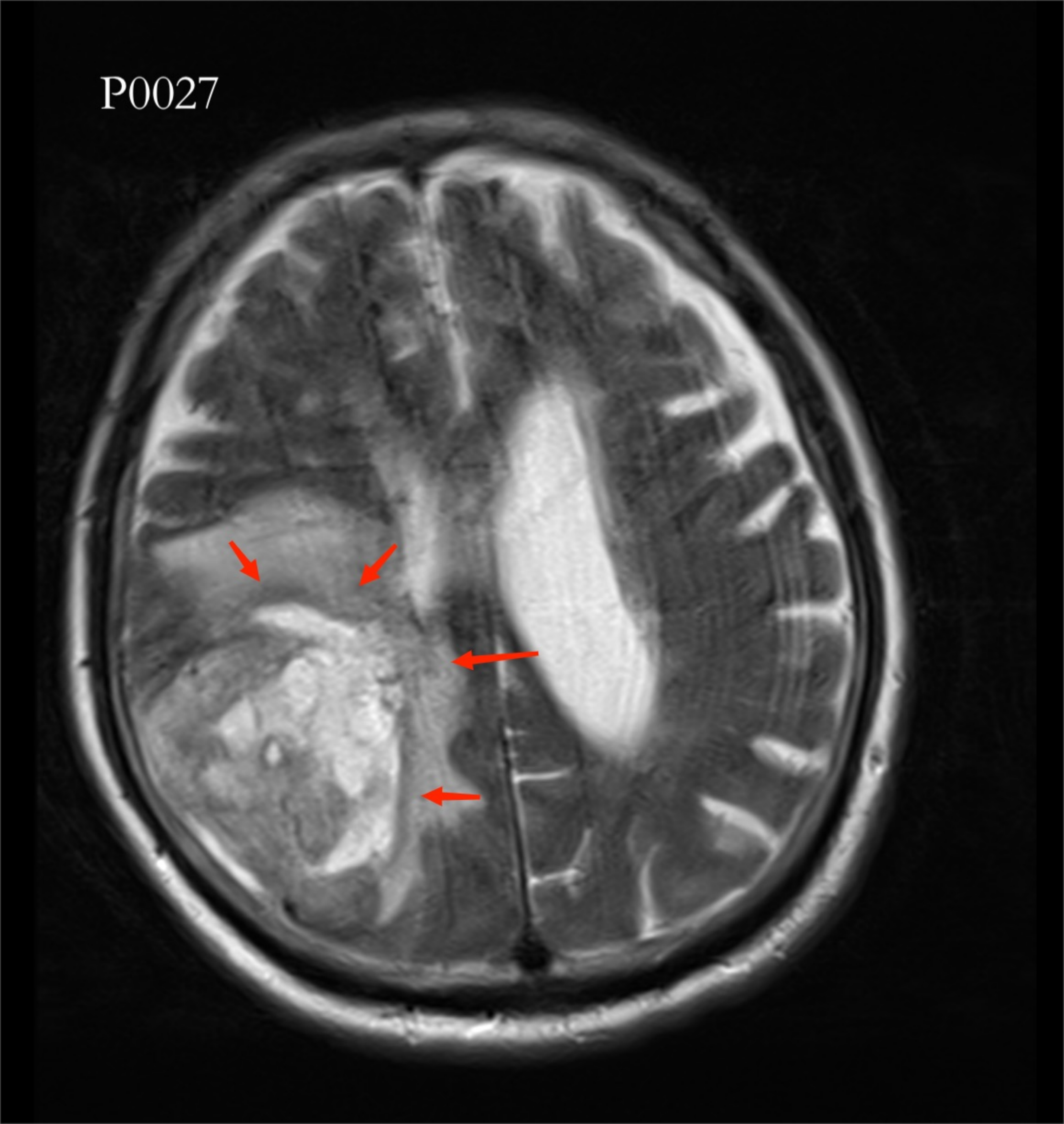
P0027

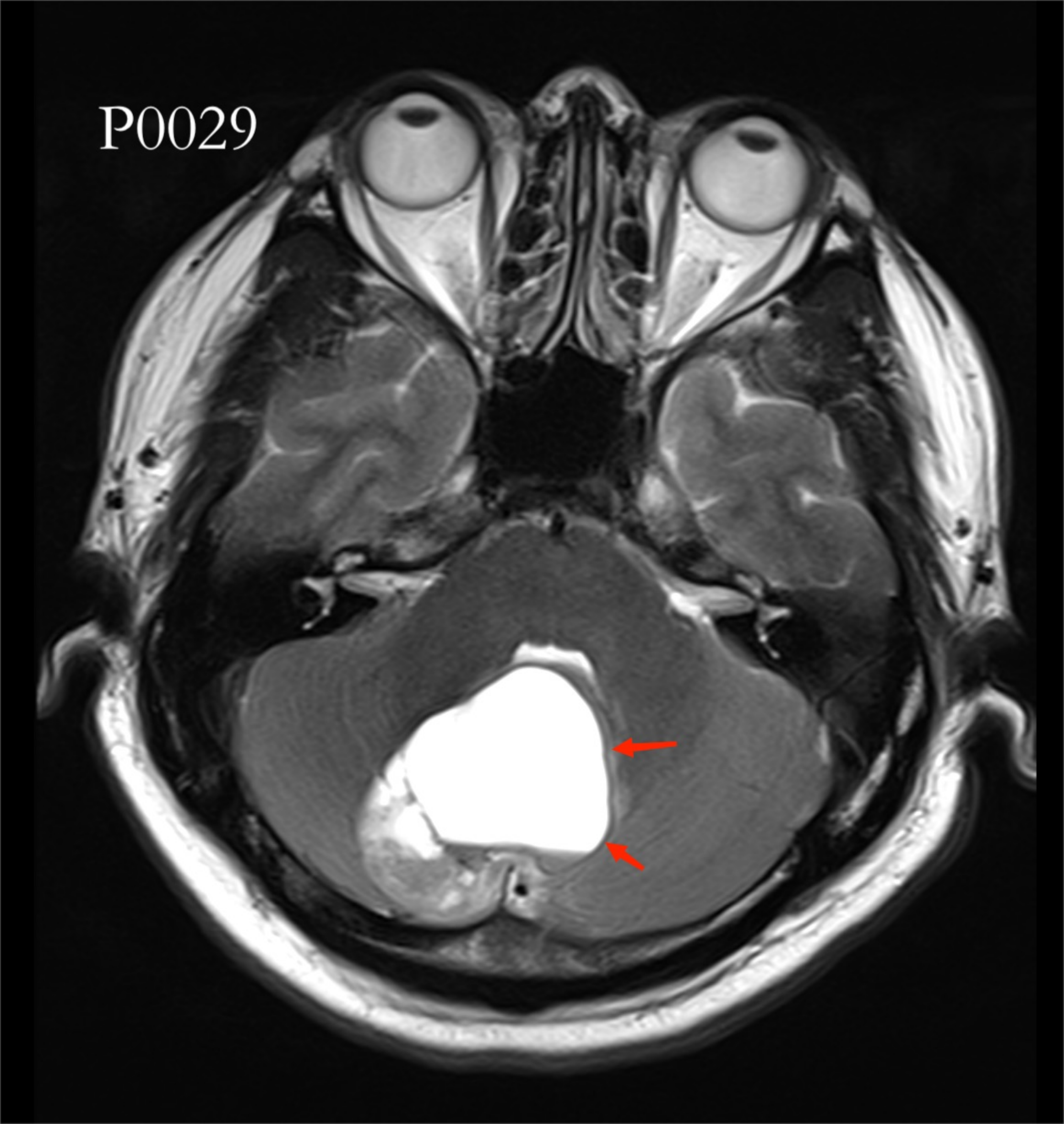
P0029

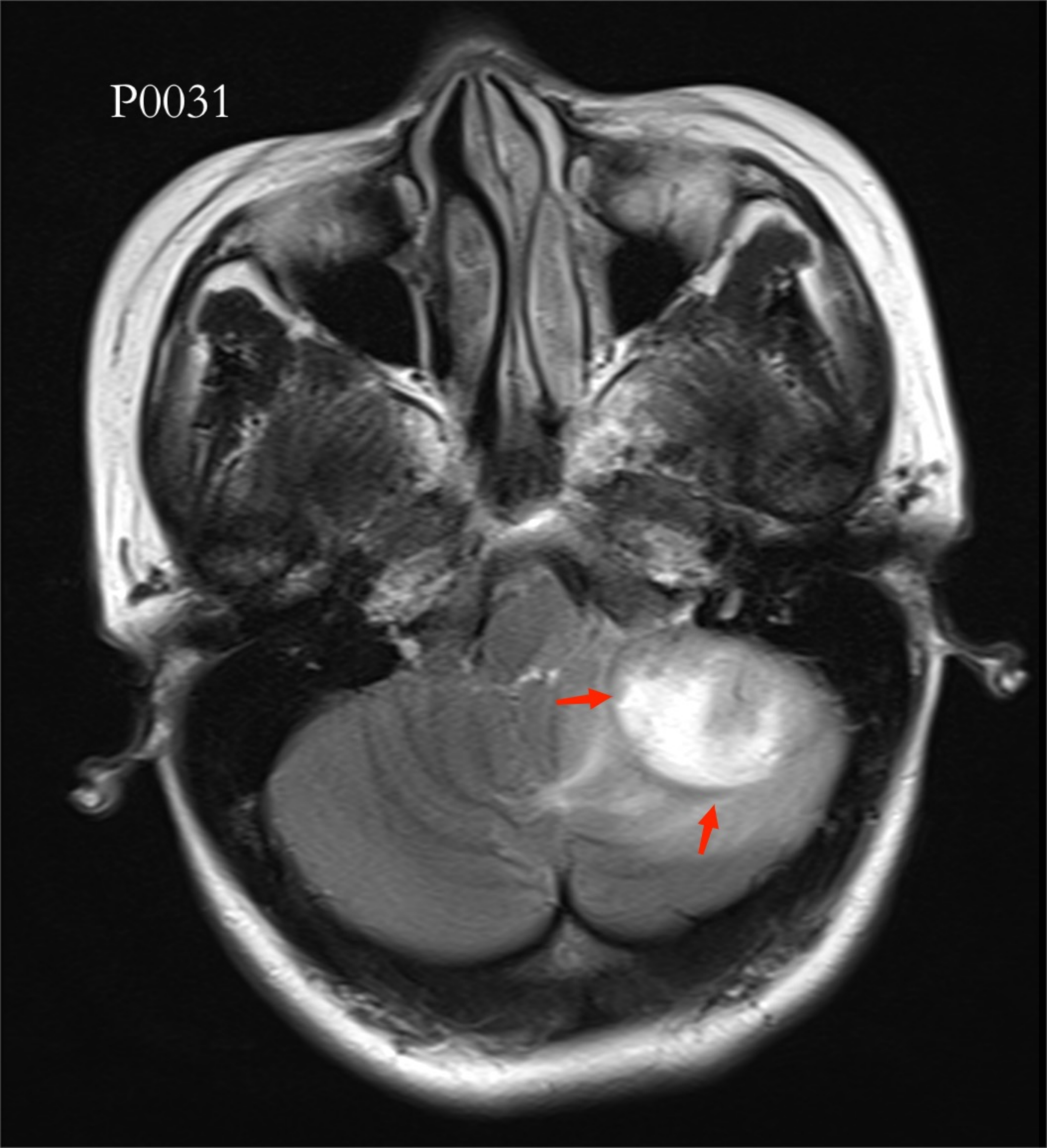
P0031

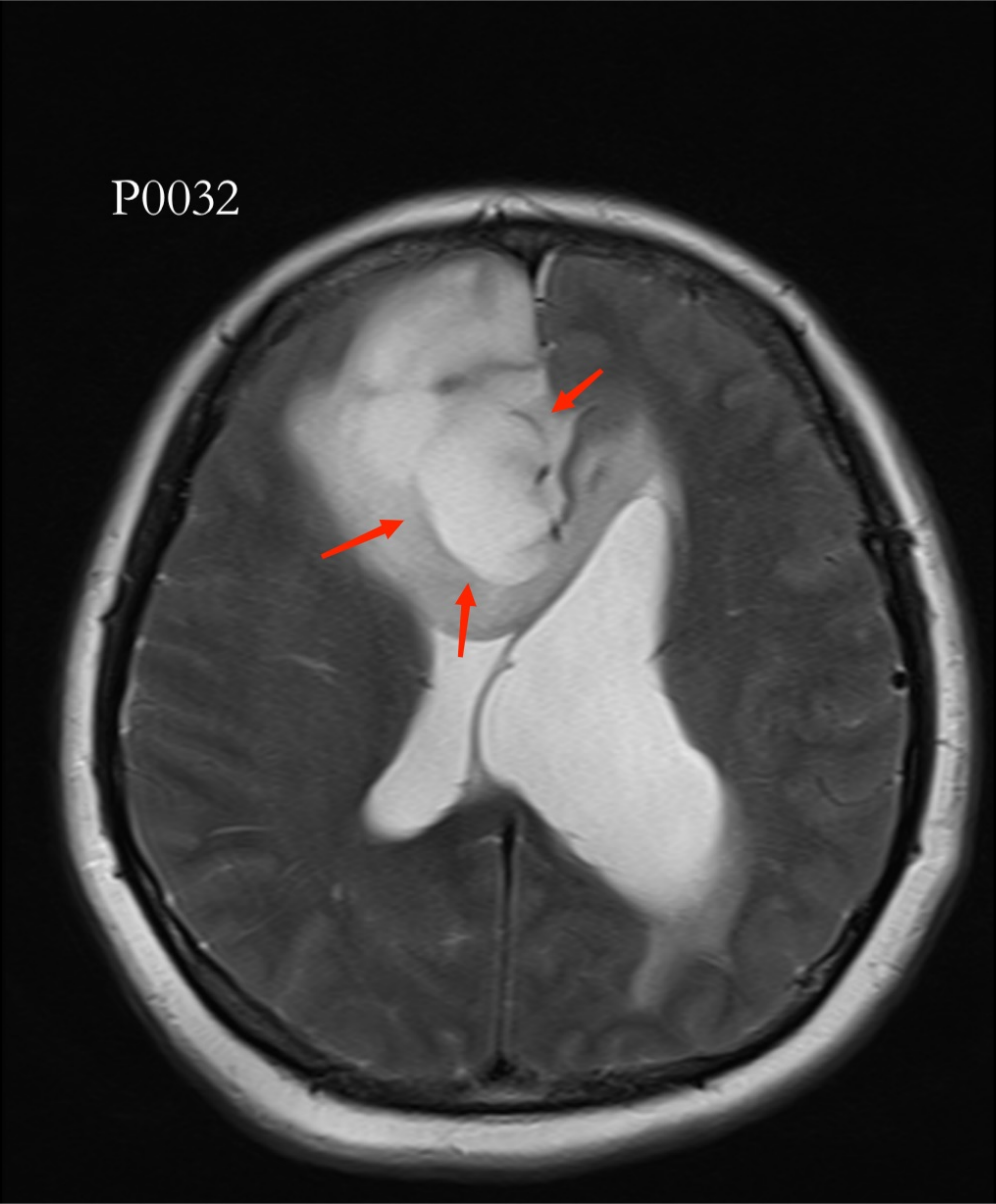
P0032

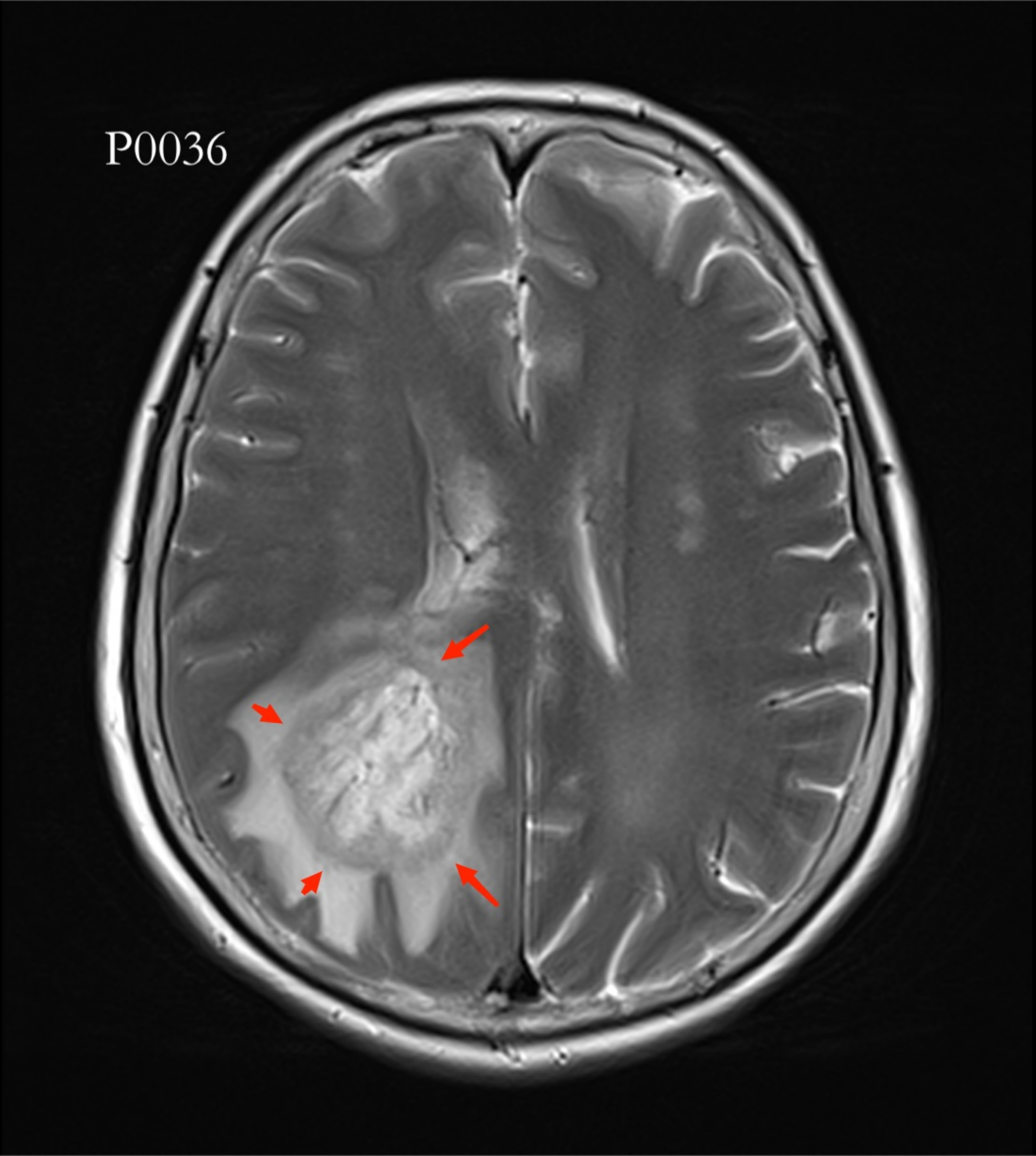
P0036

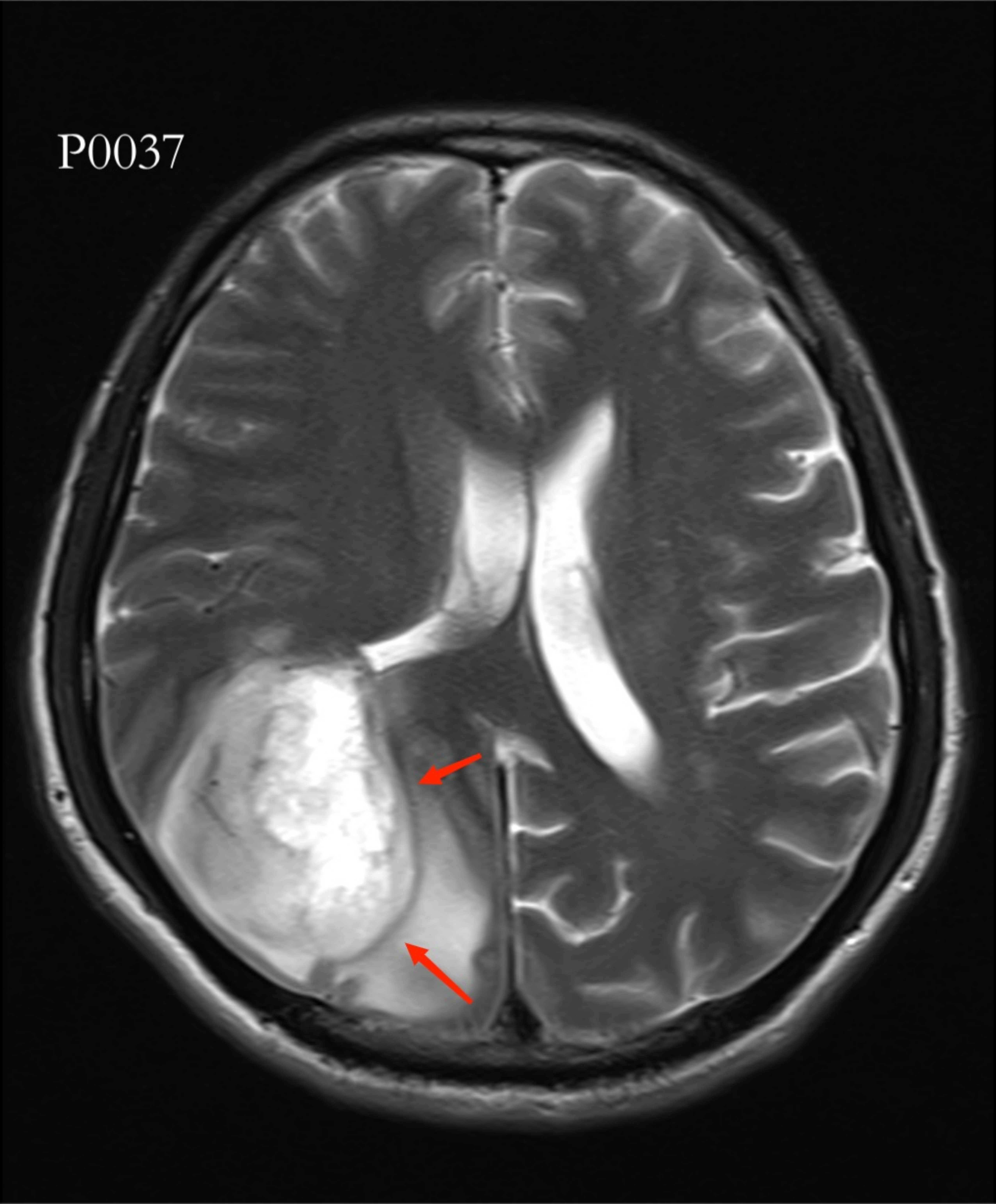
P0037

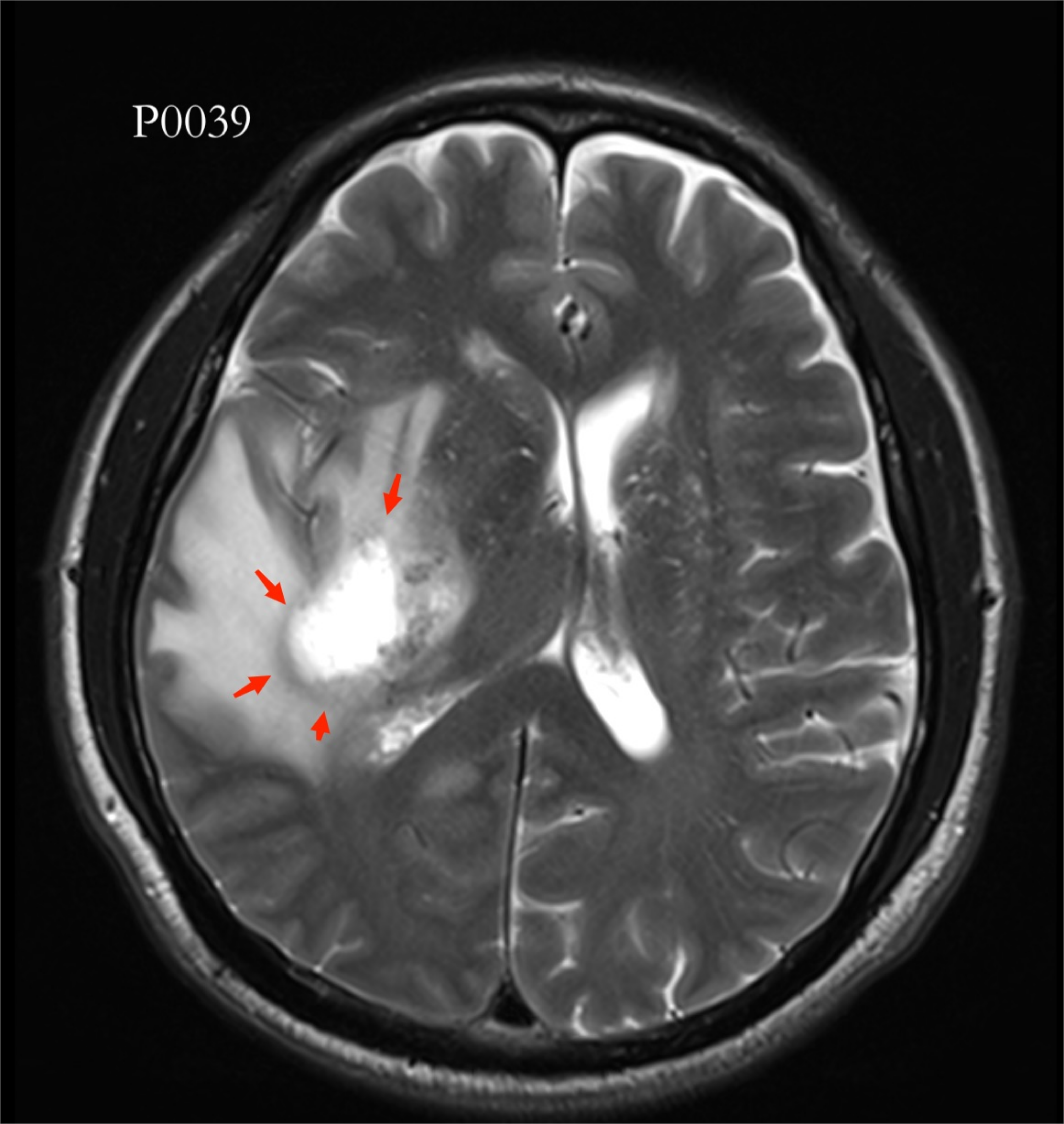
P0039

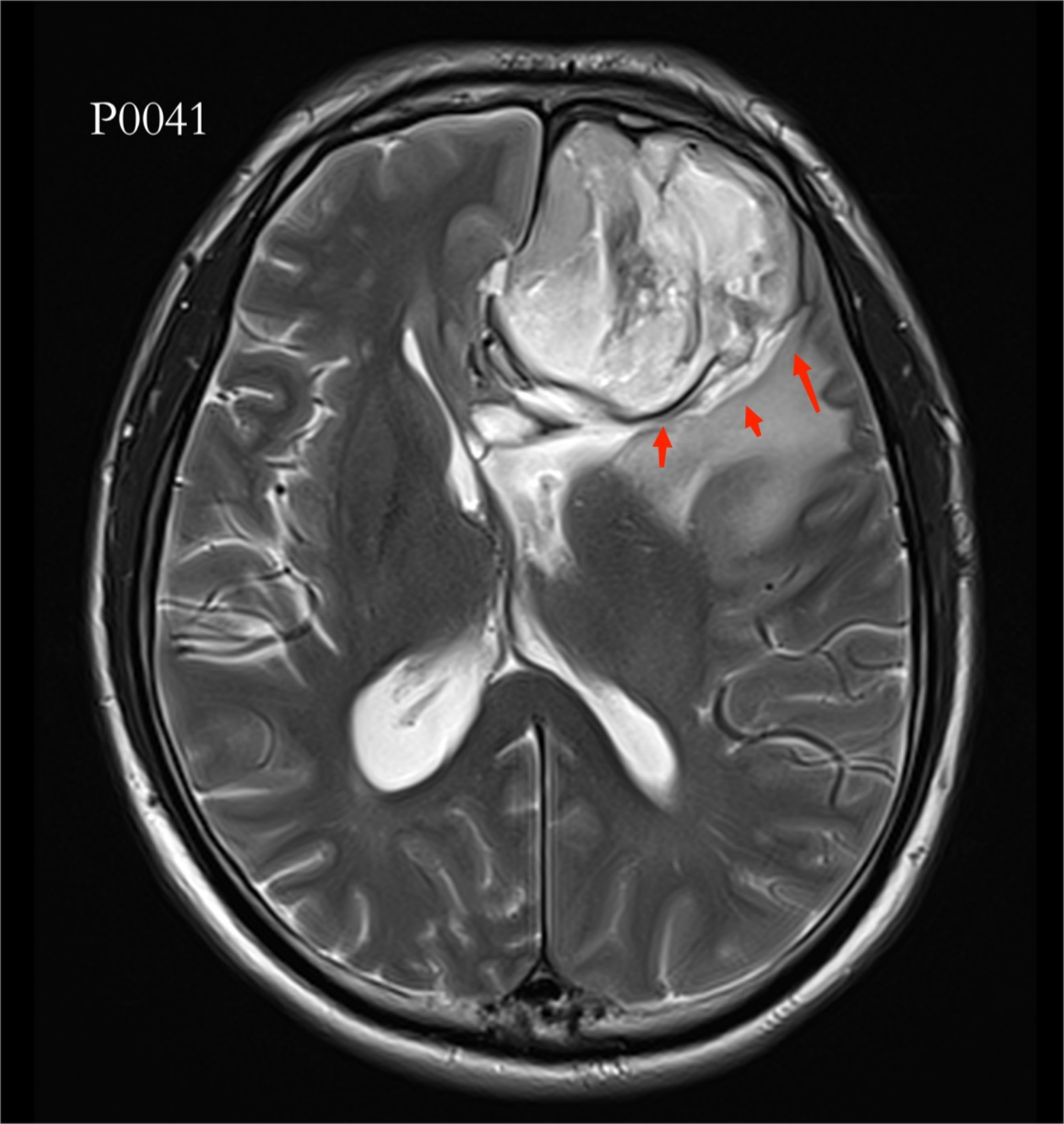
P0041

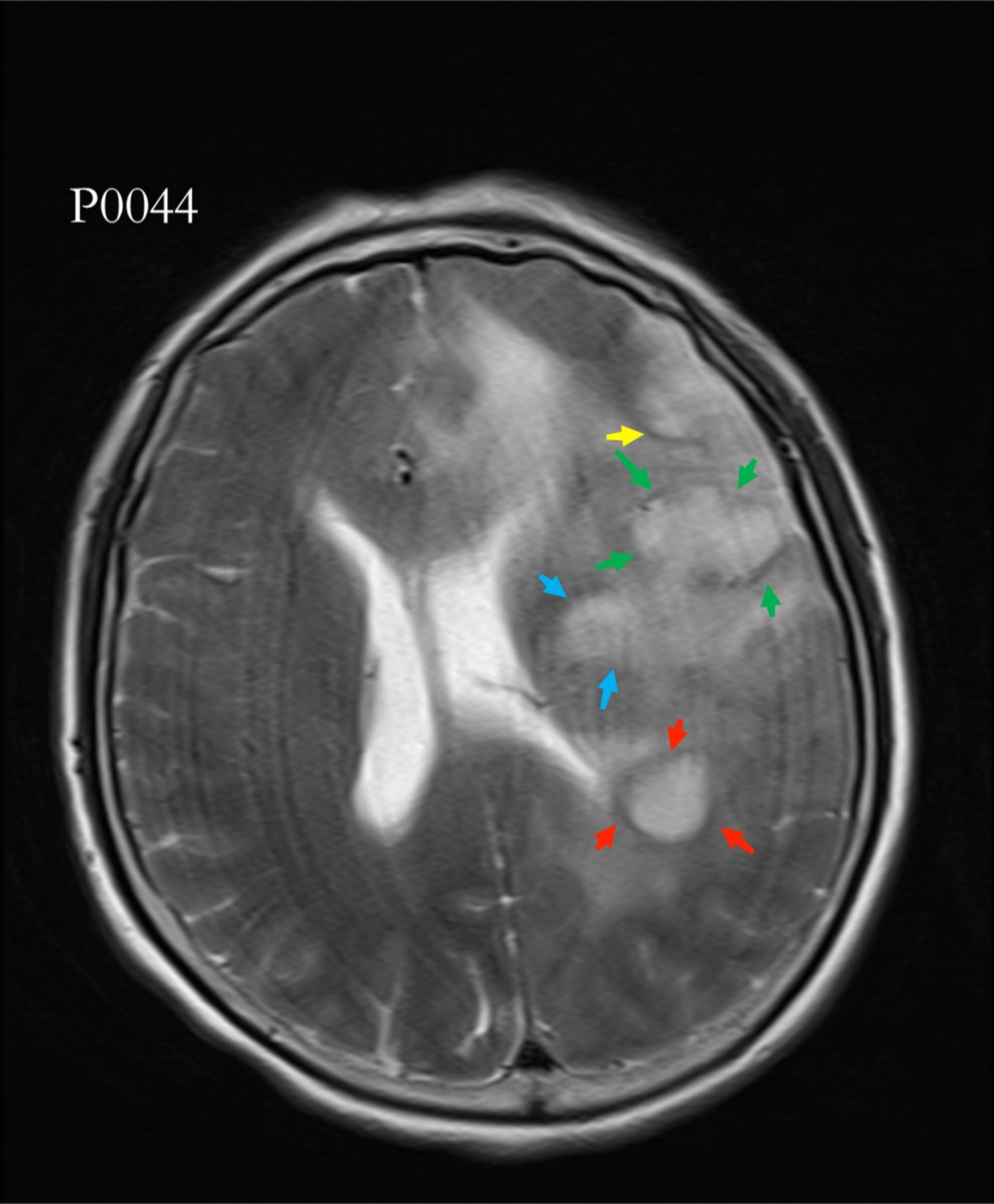
P0044

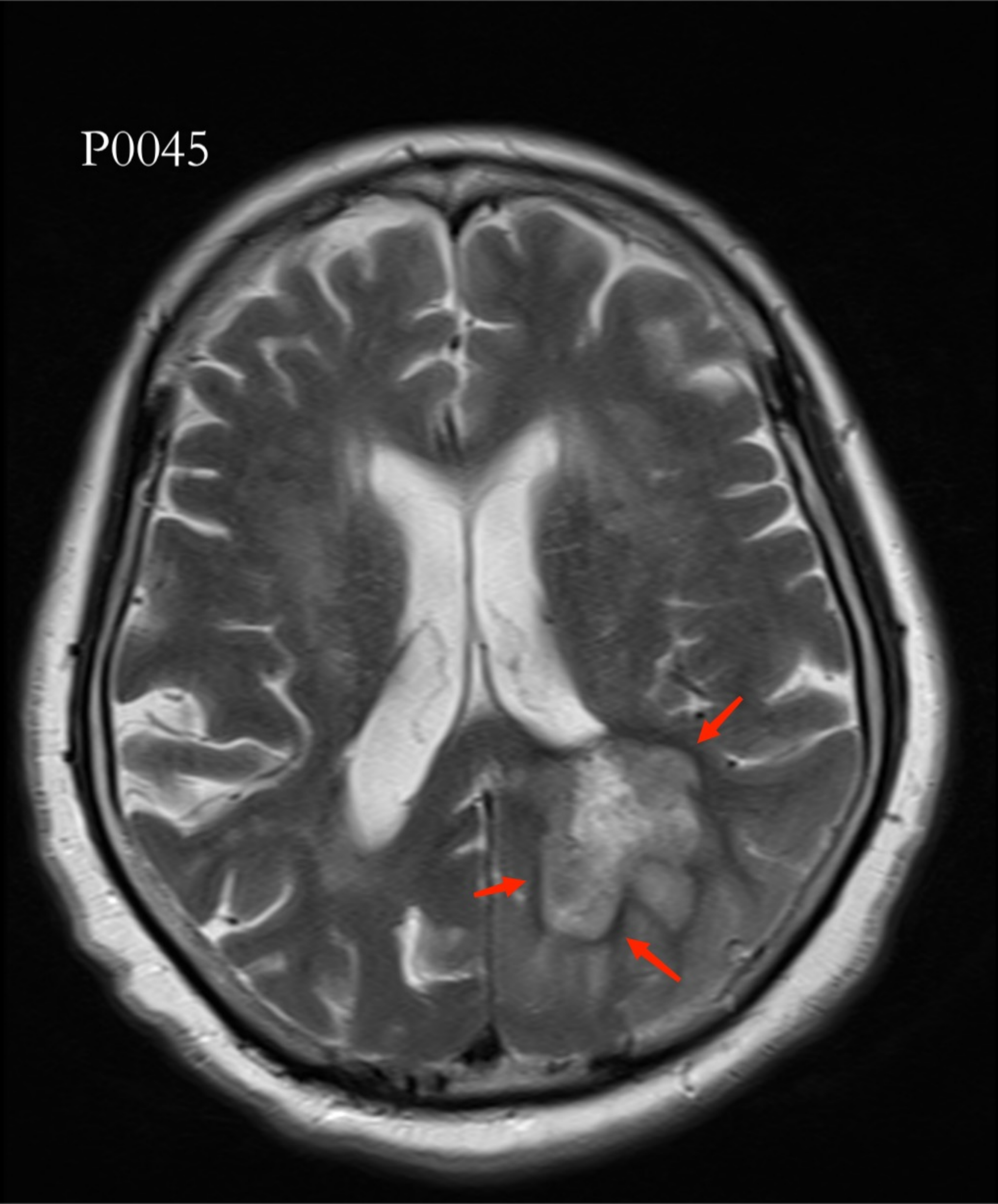
P0045

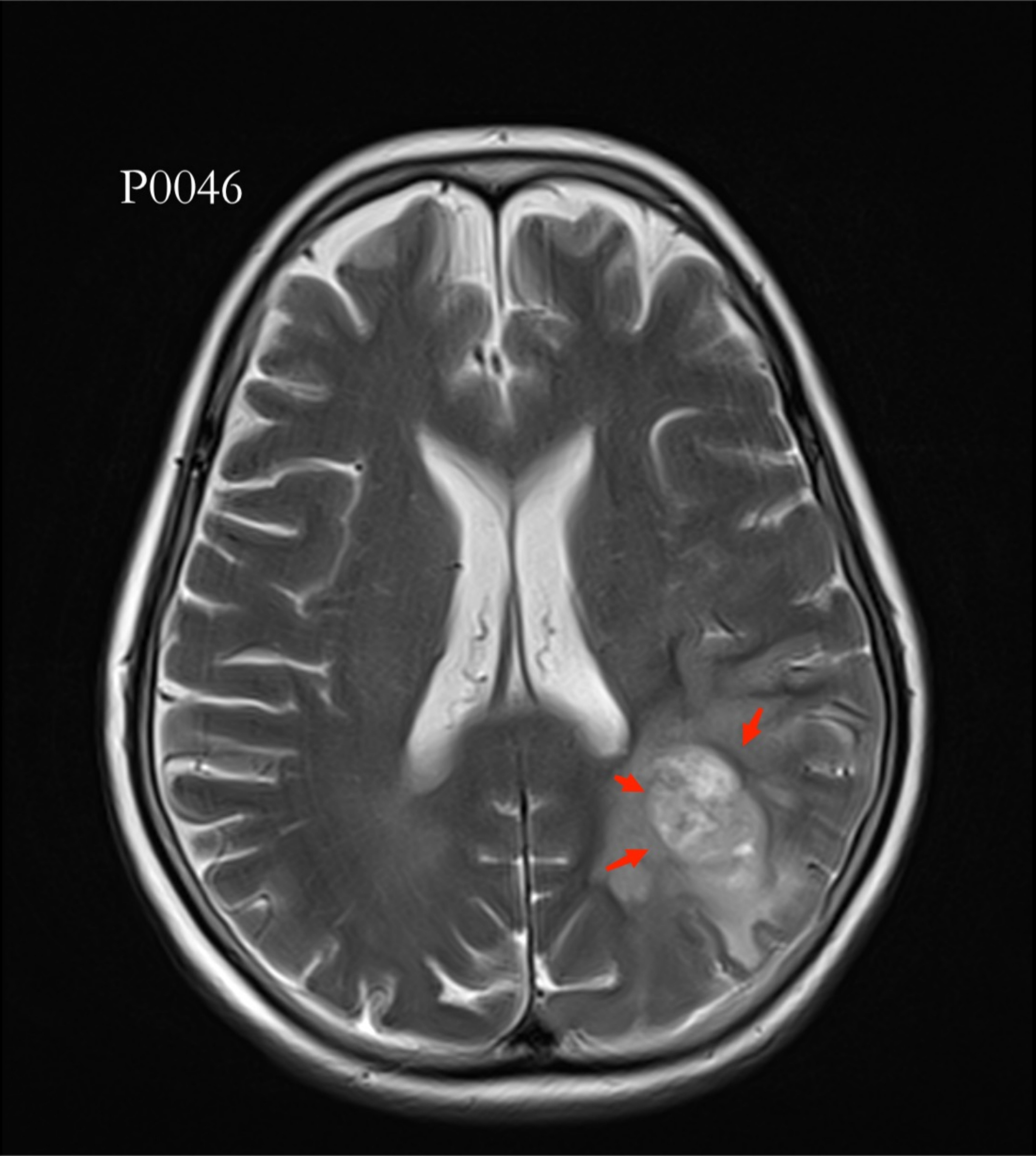
P0046

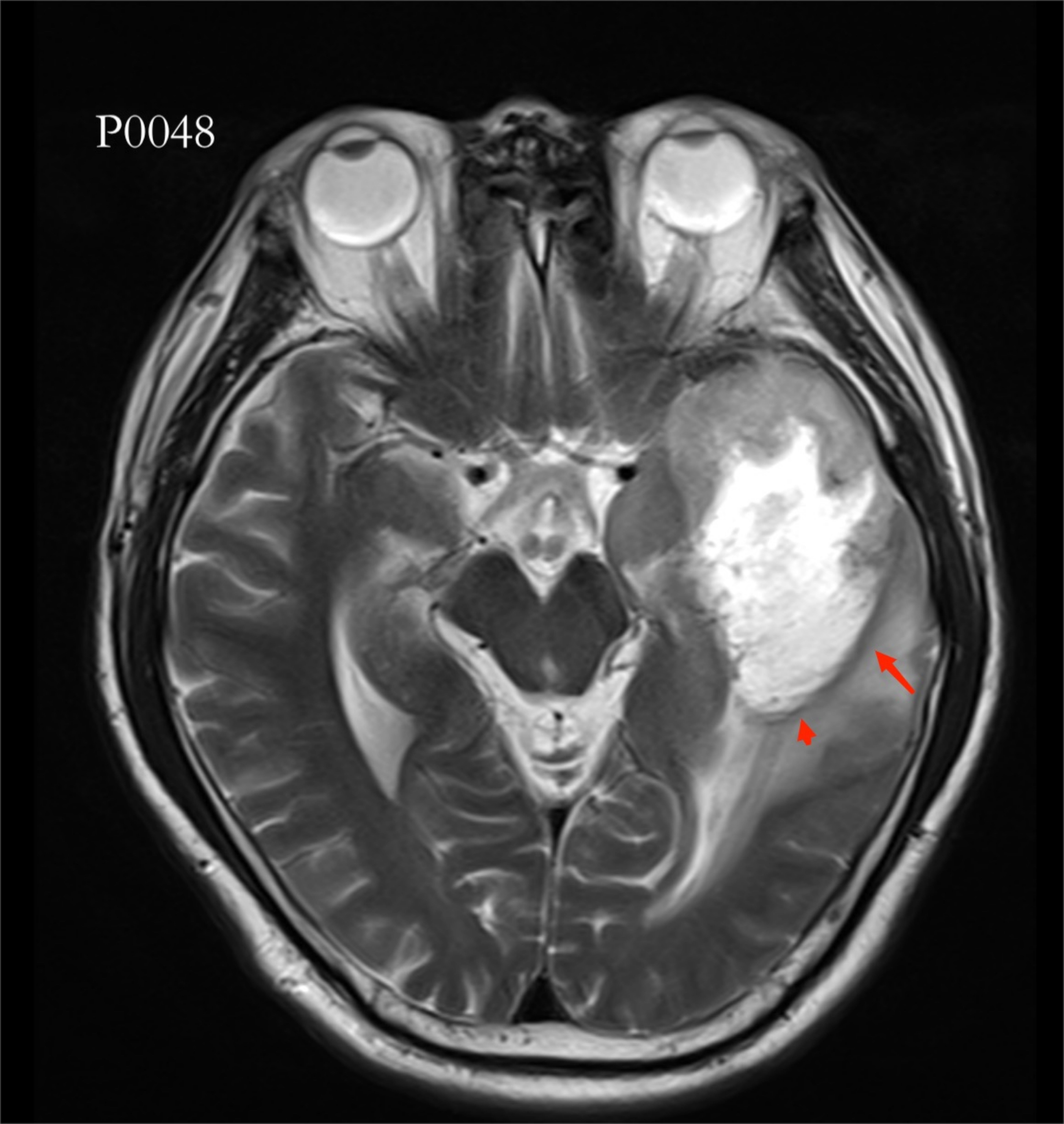
P0048

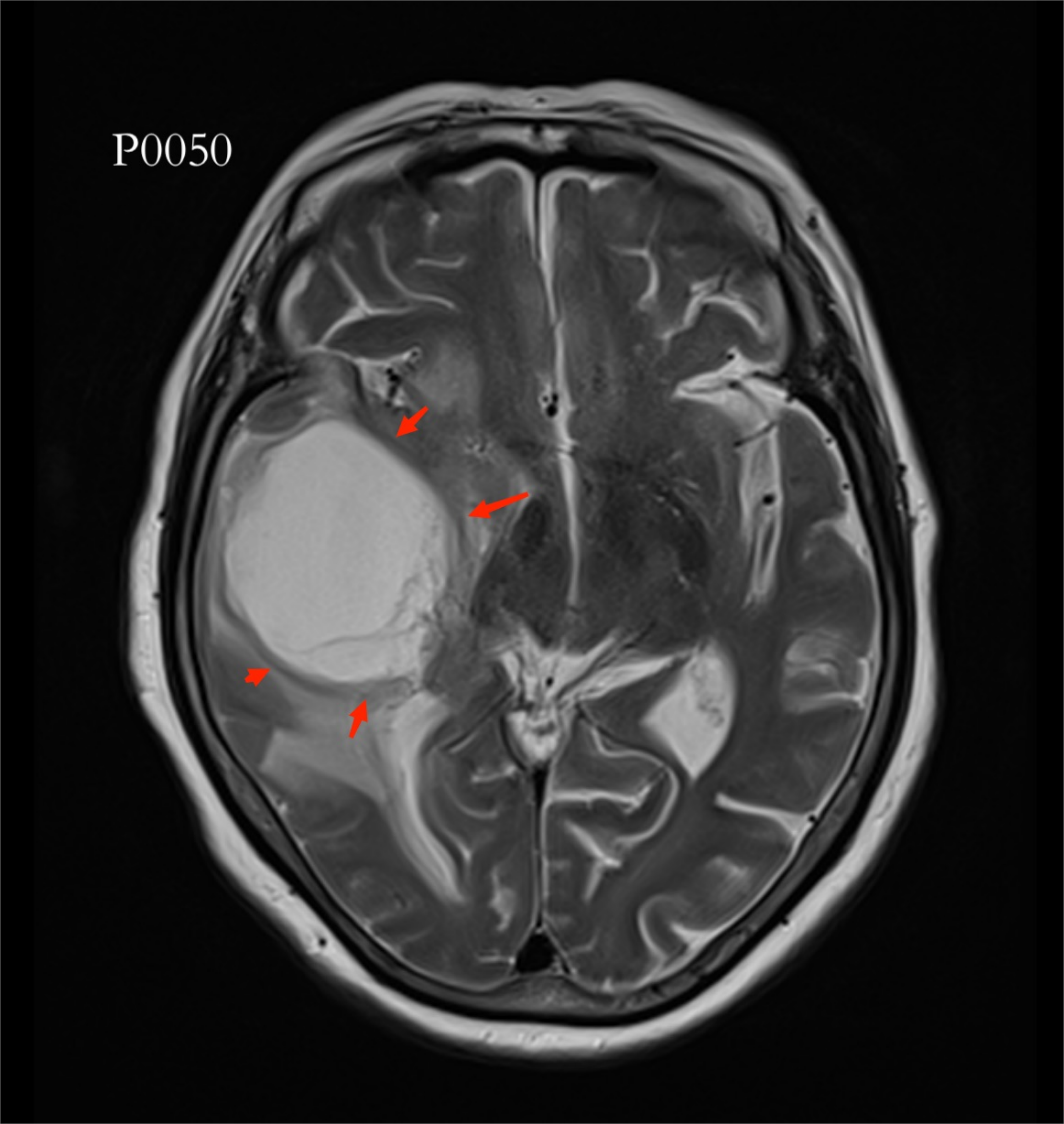
P0050

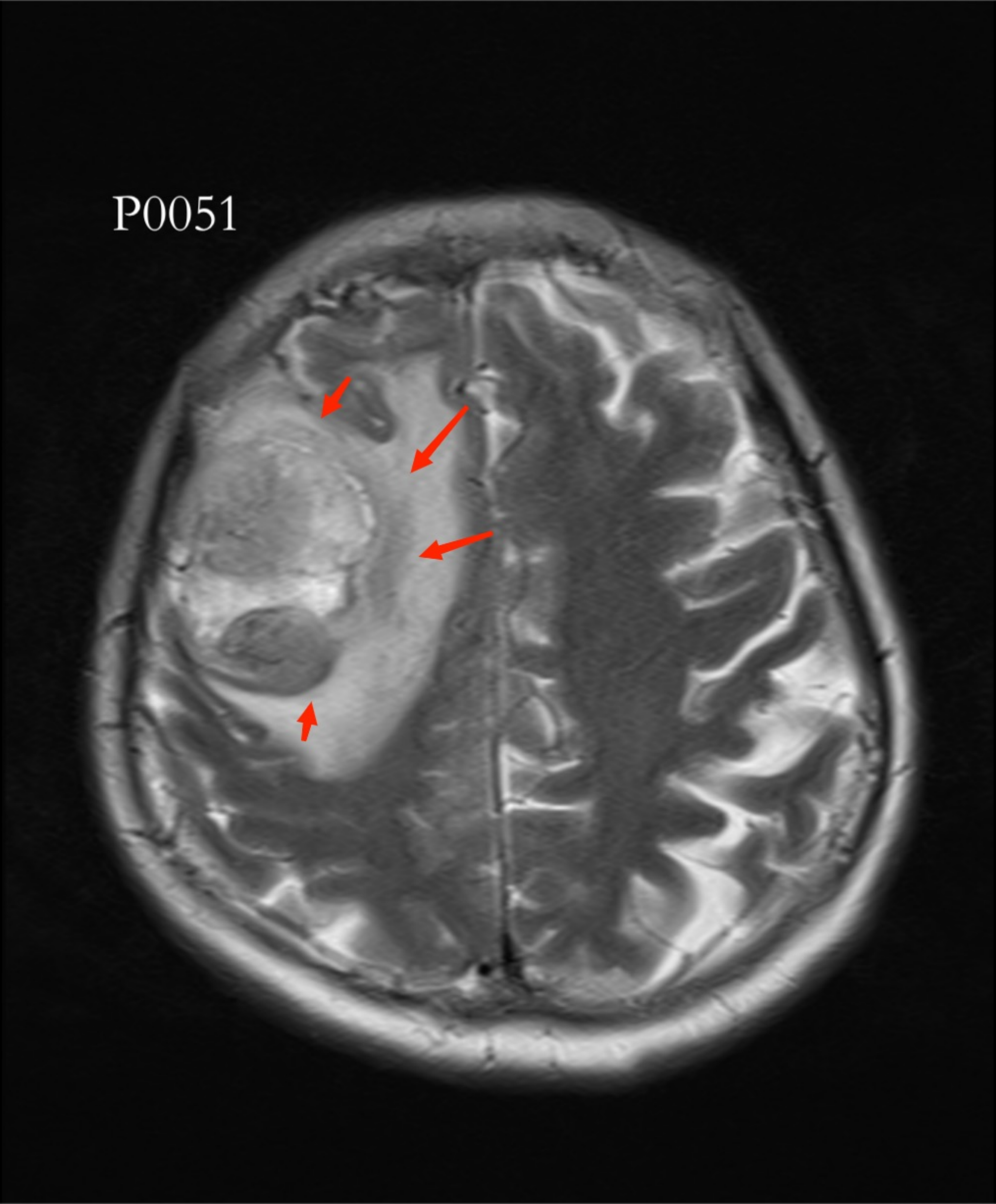
P0051

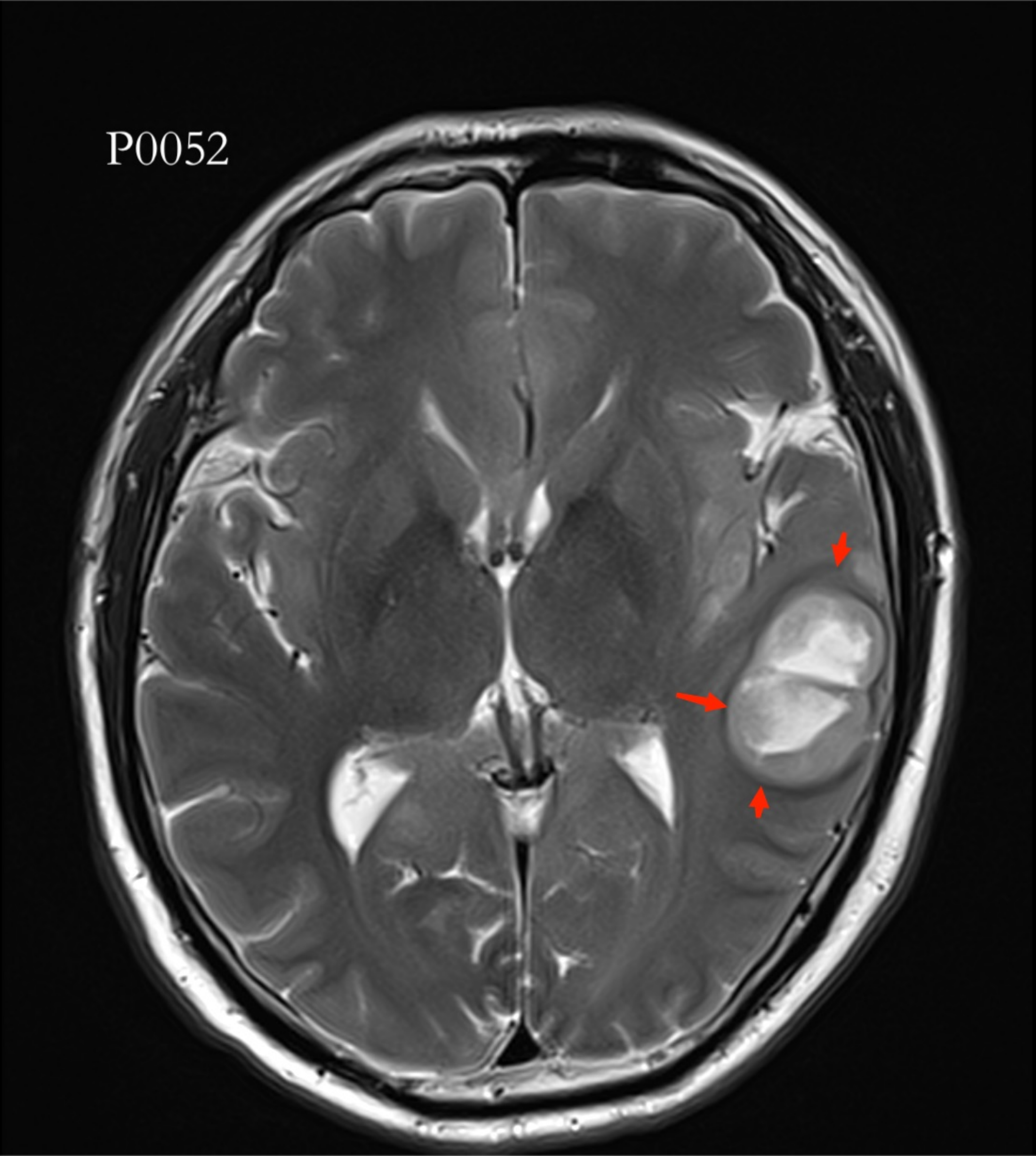
P0052

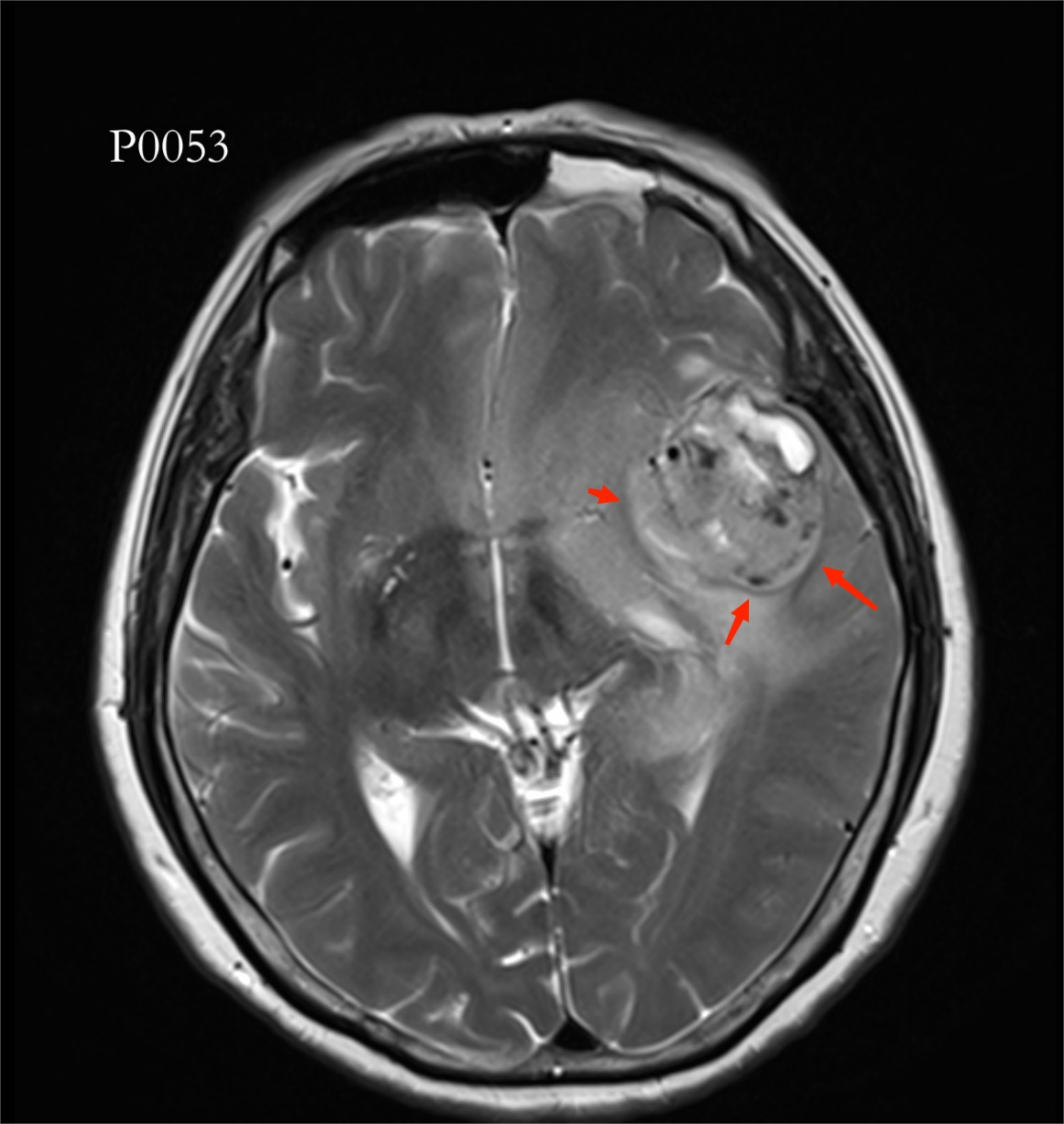
P0053

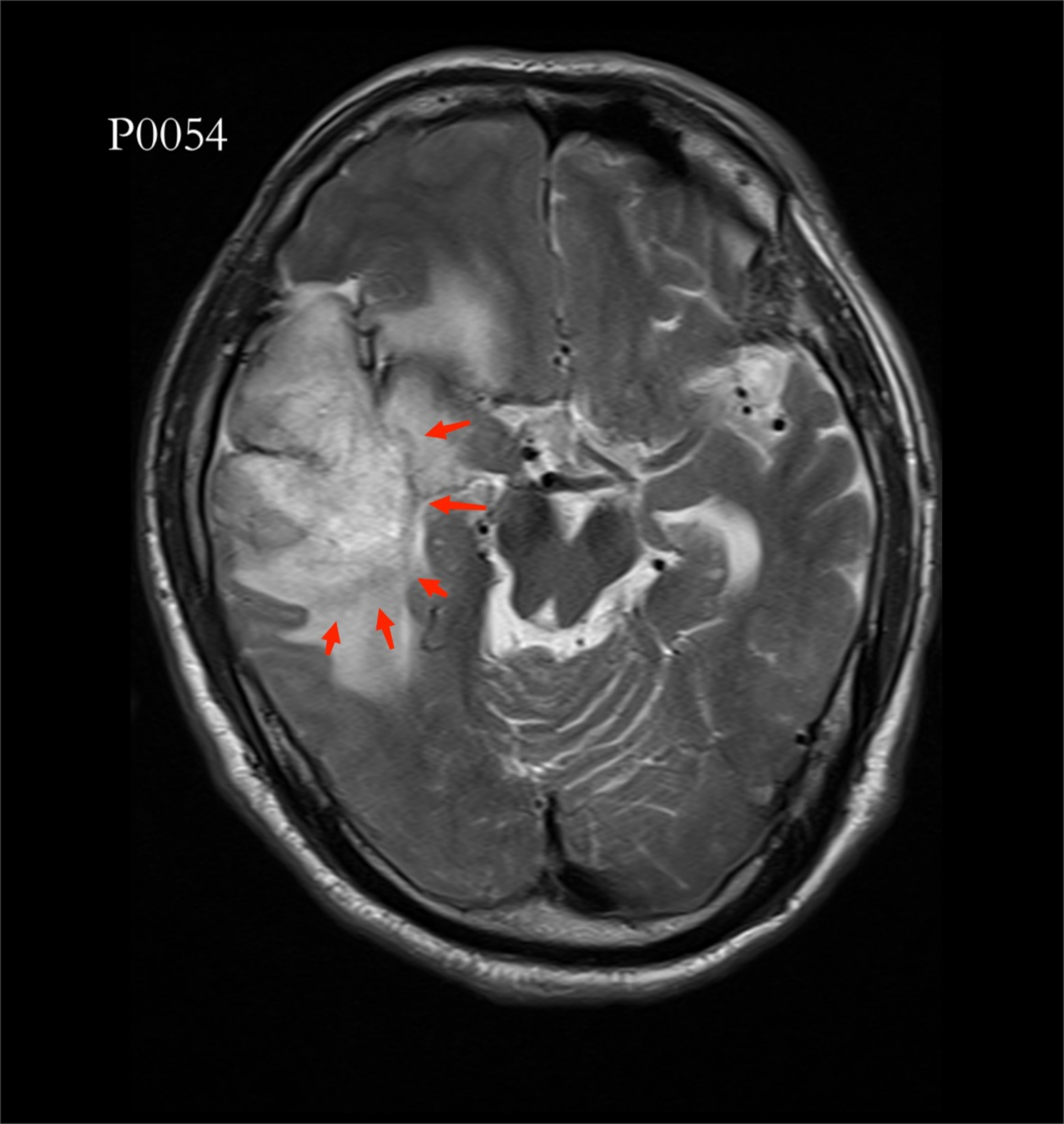
P0054

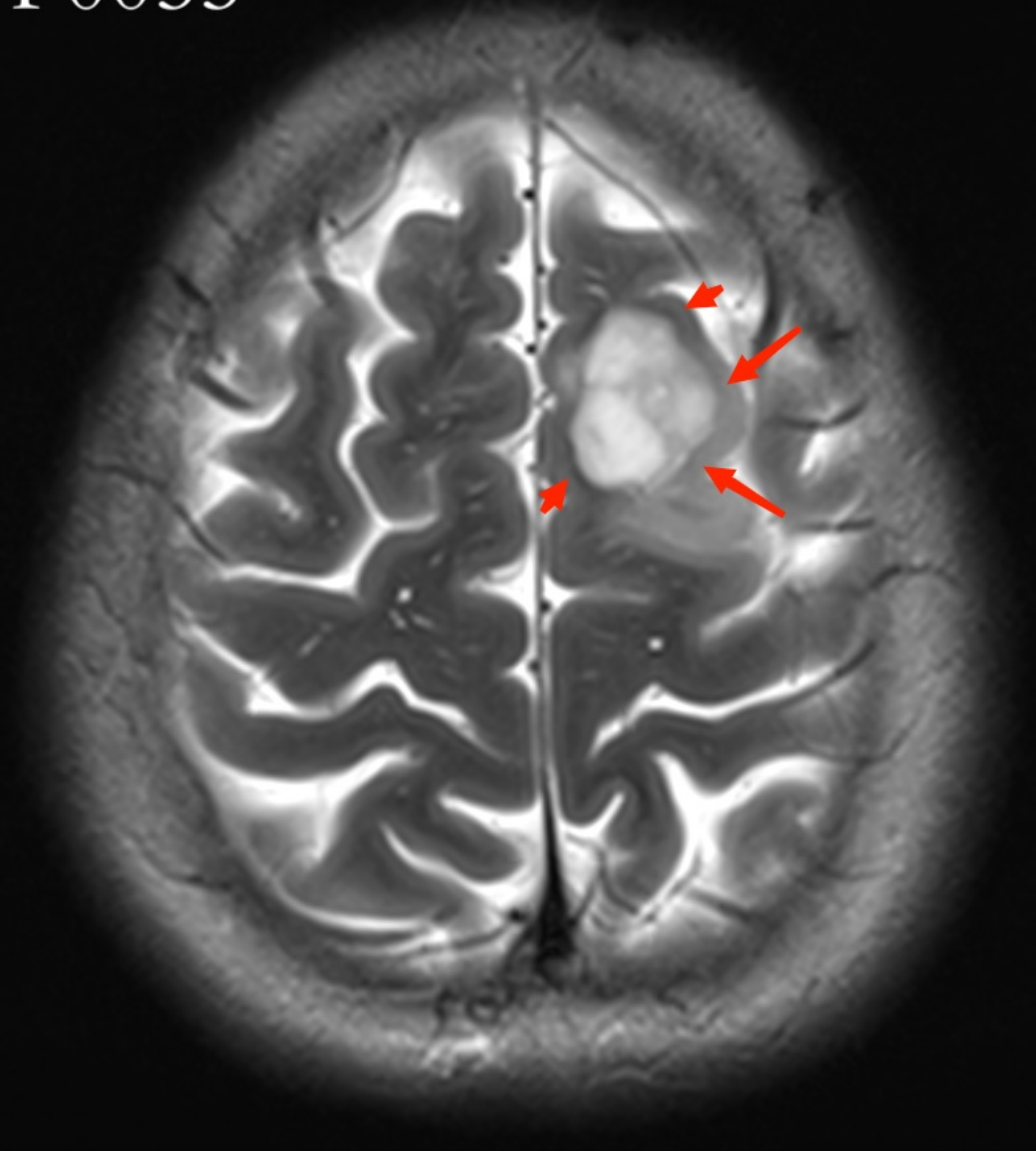
P0055

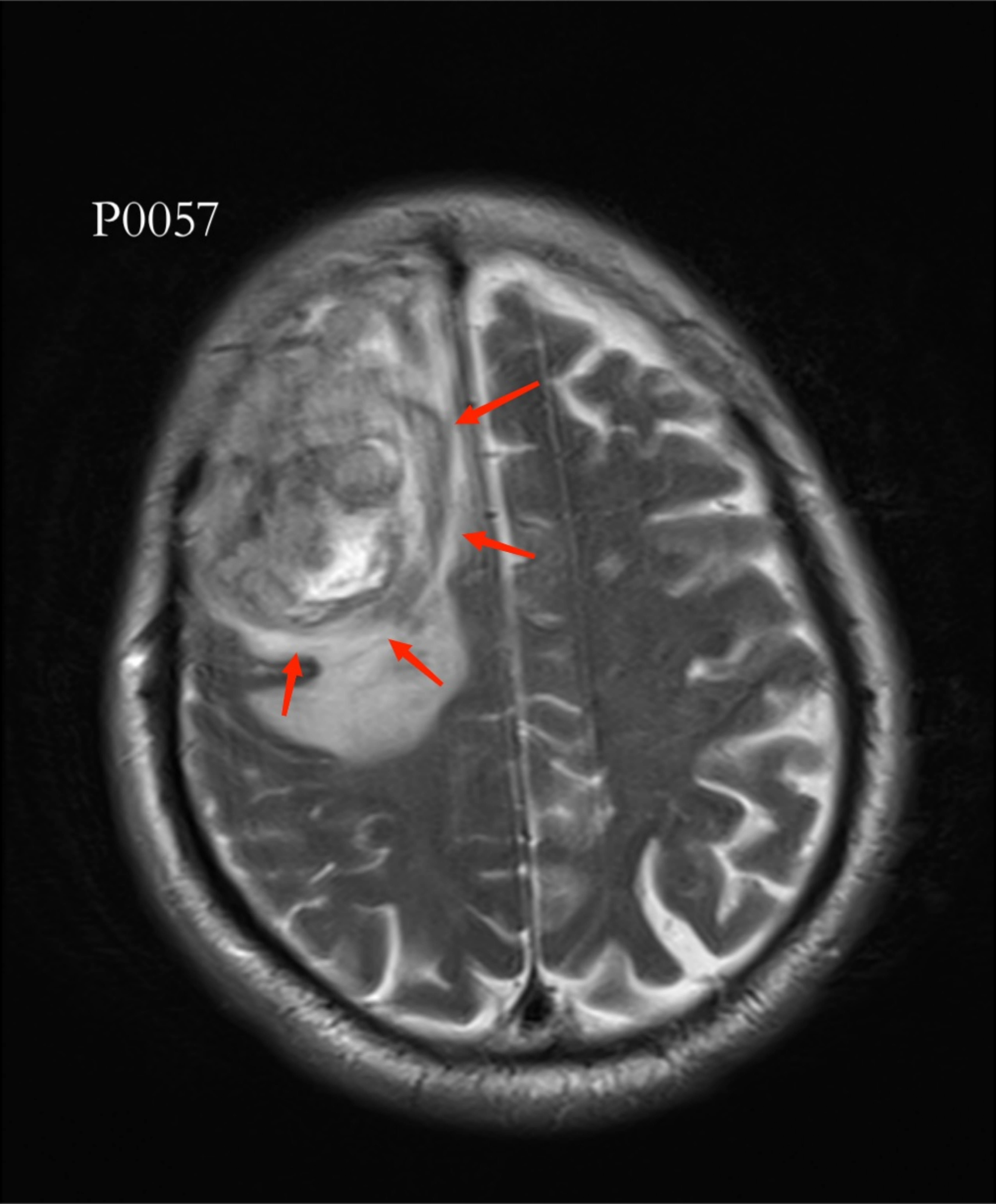
P0057

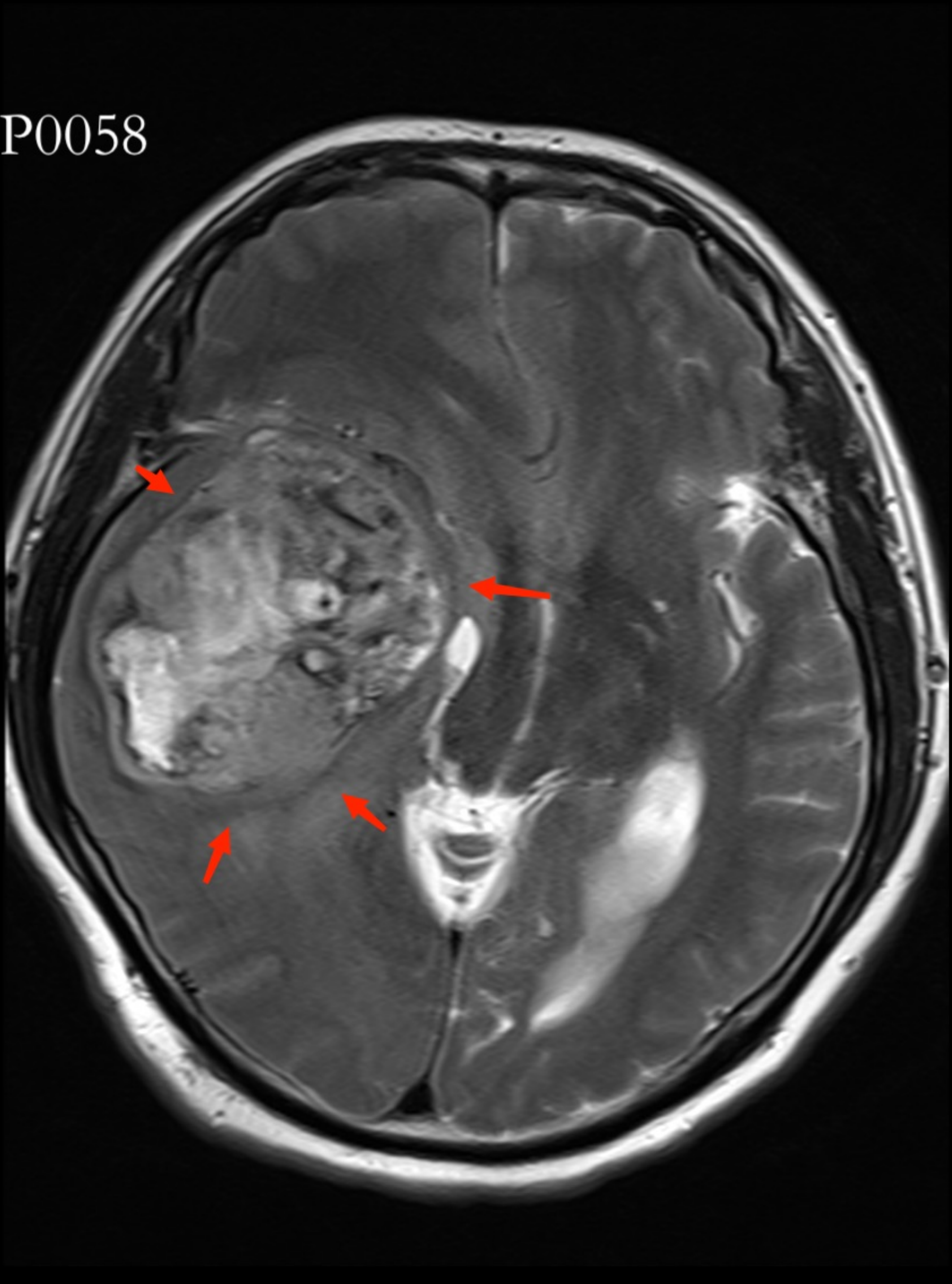
P0058

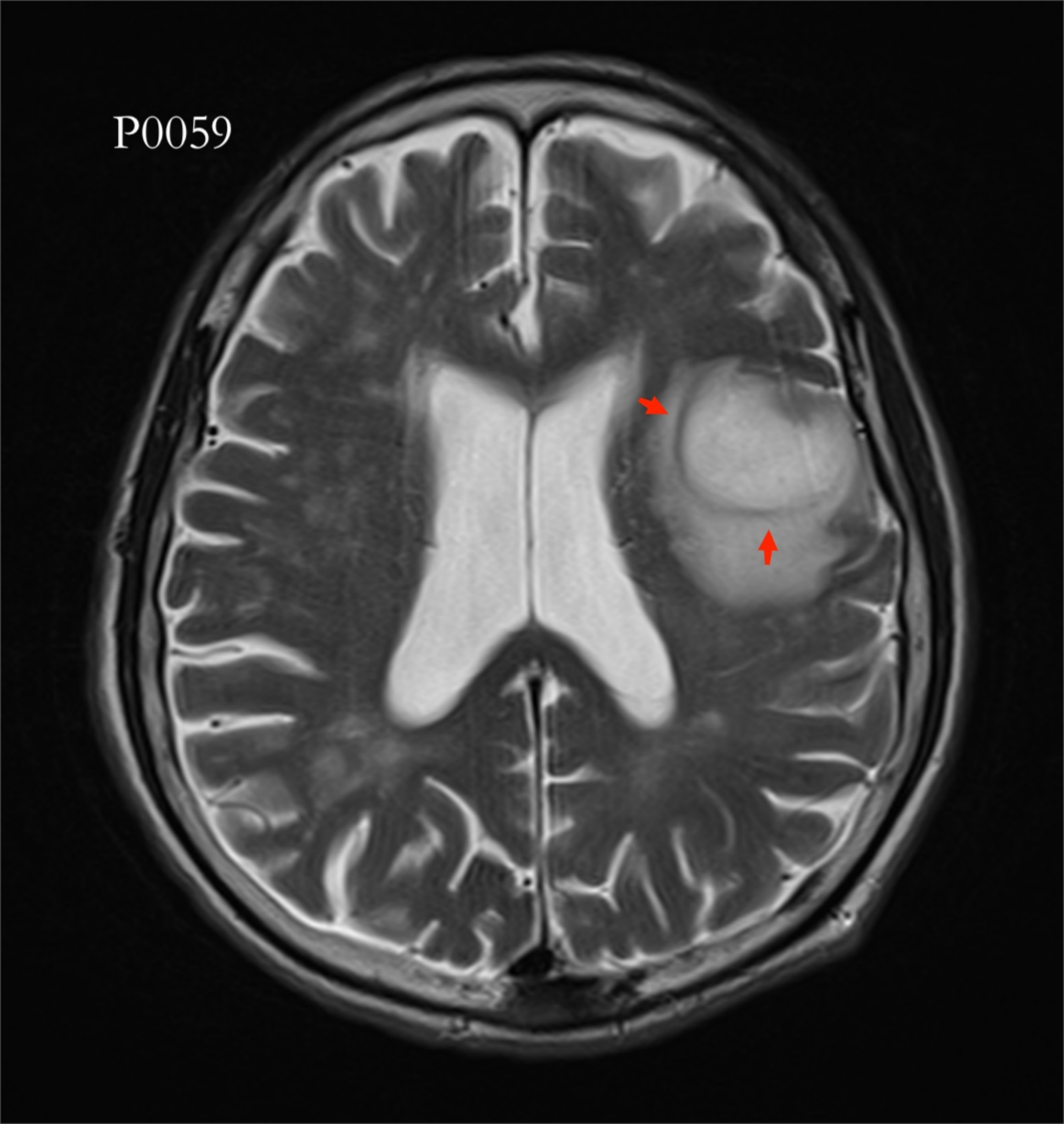
P0059

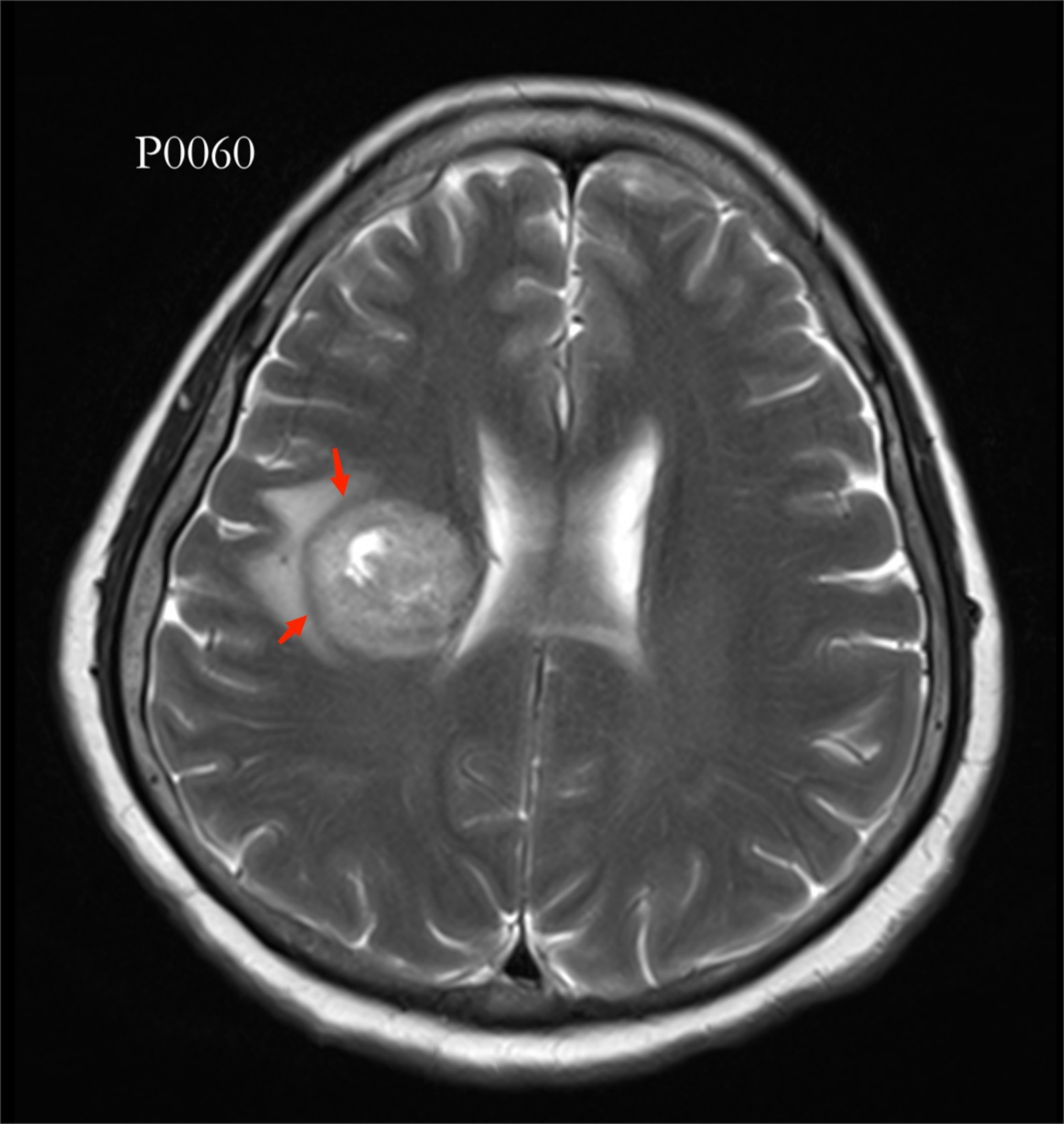
P0060

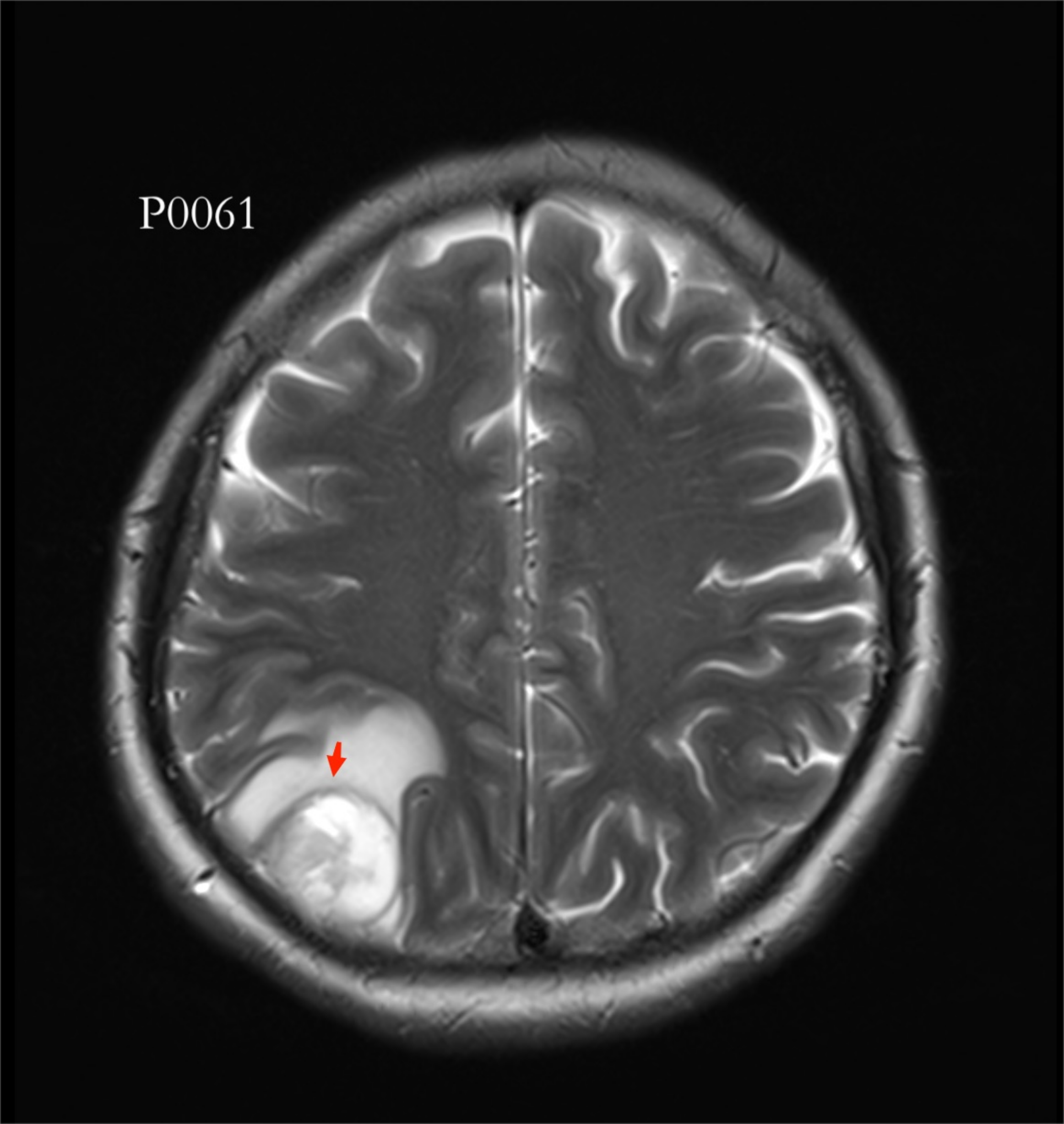
P0061

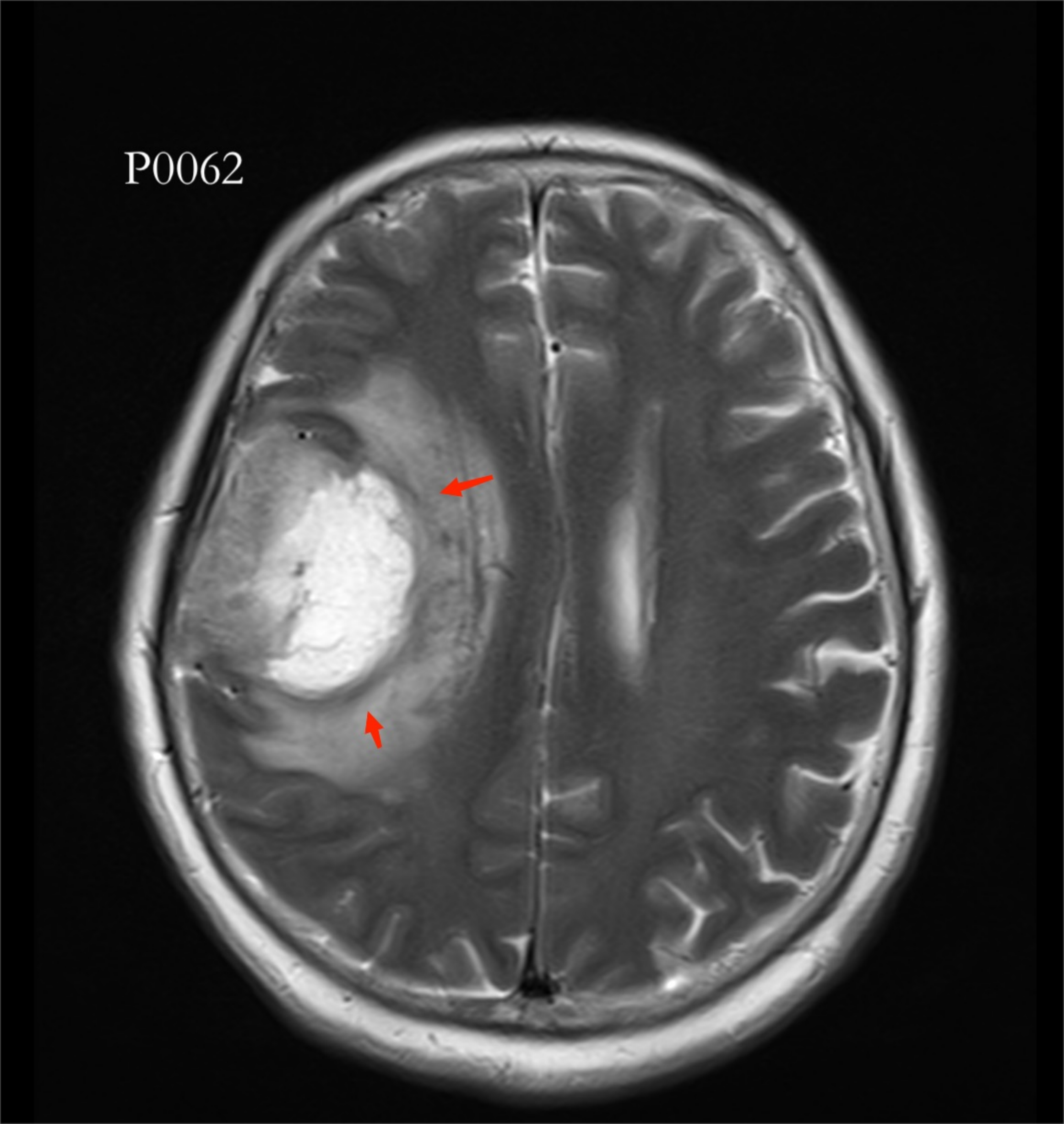
P0062

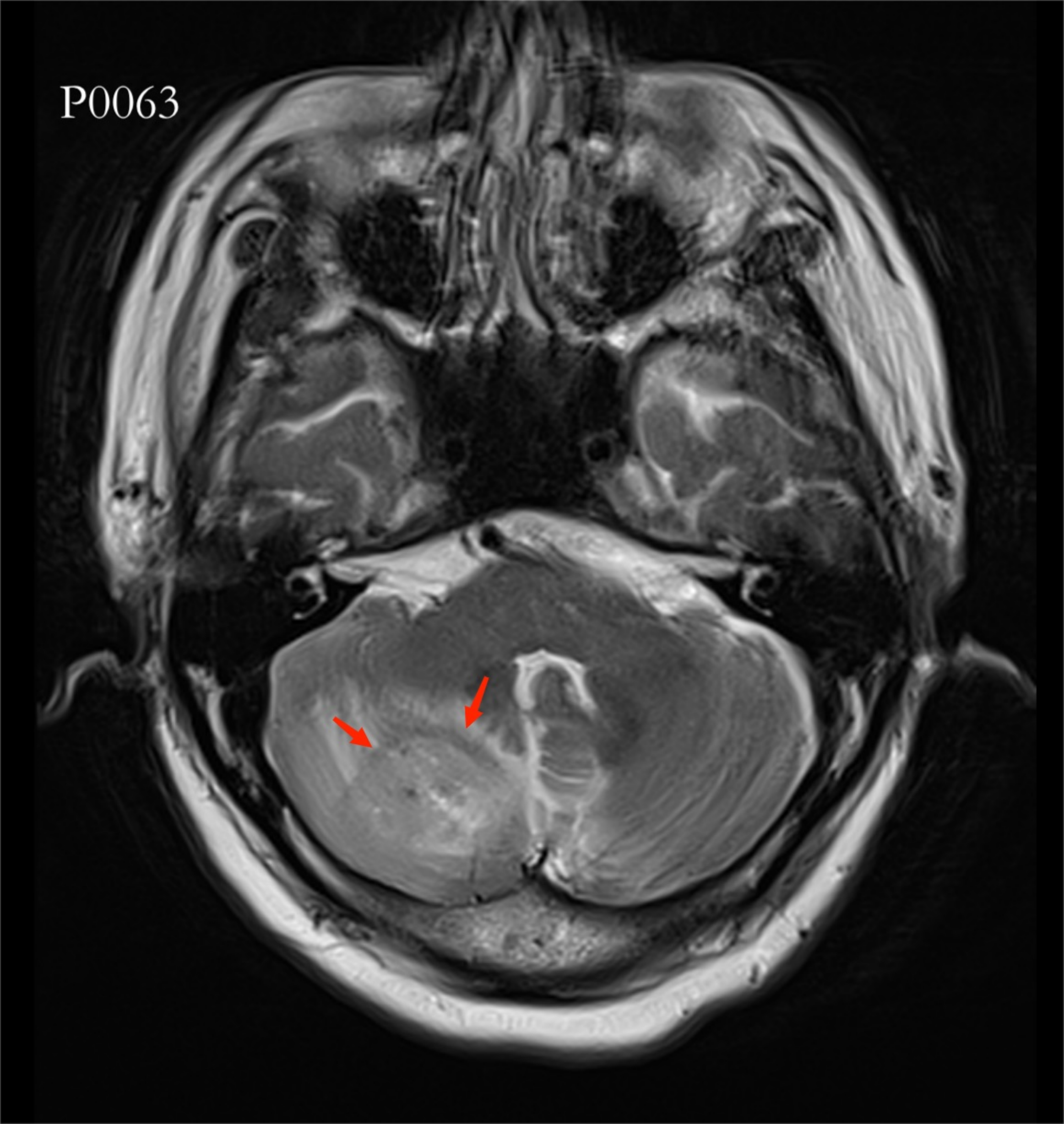
P0063

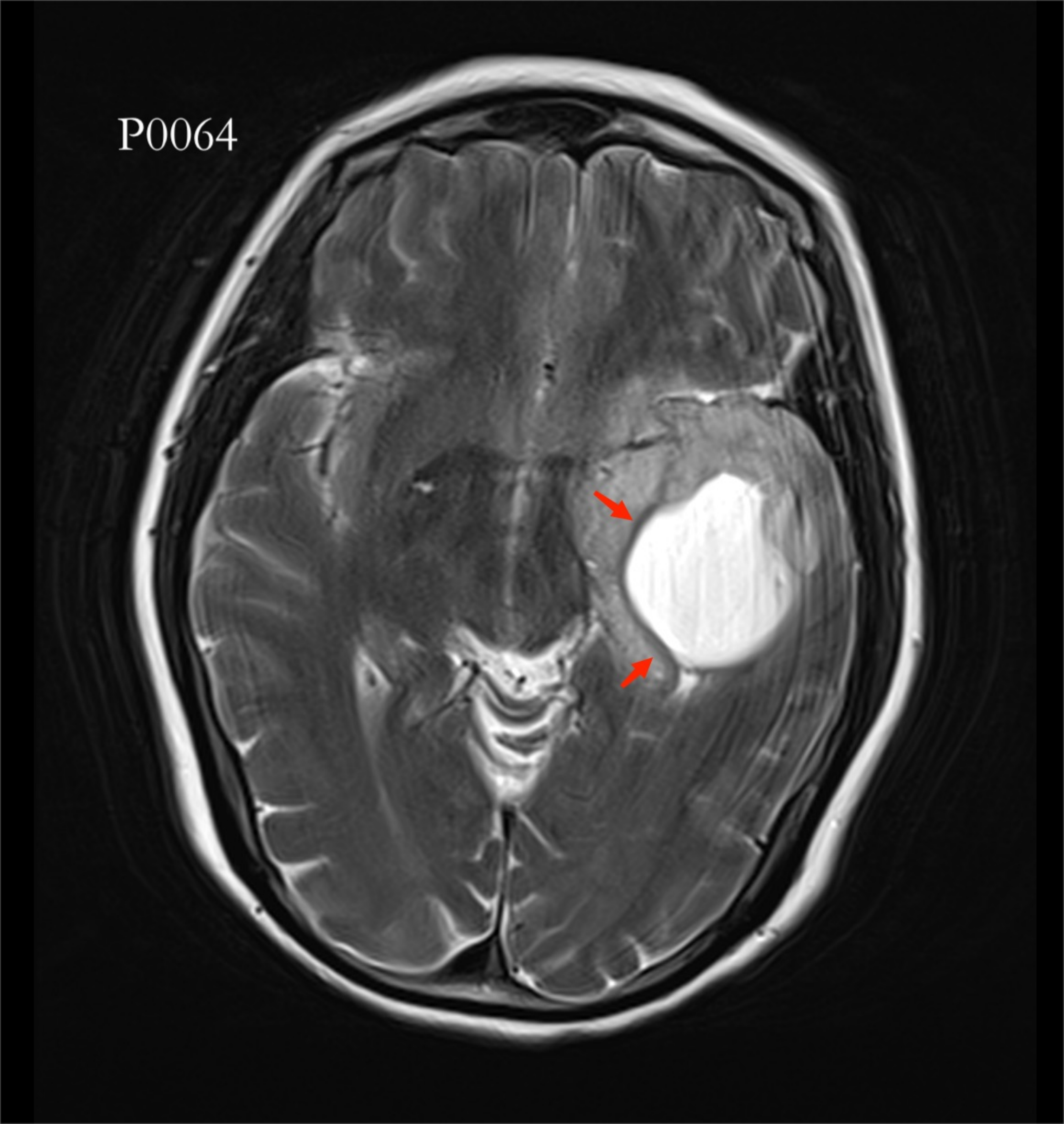
P0064

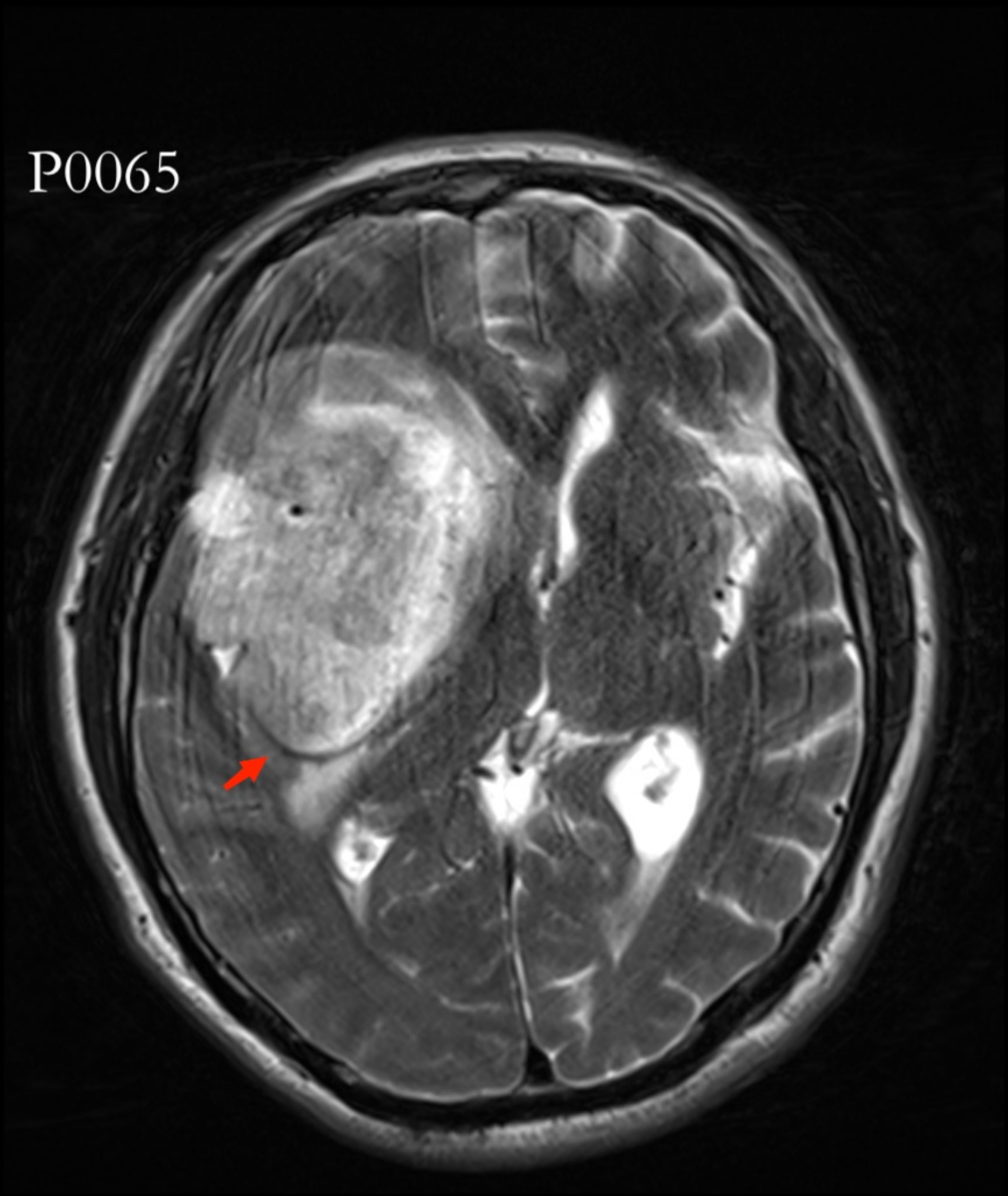
P0065

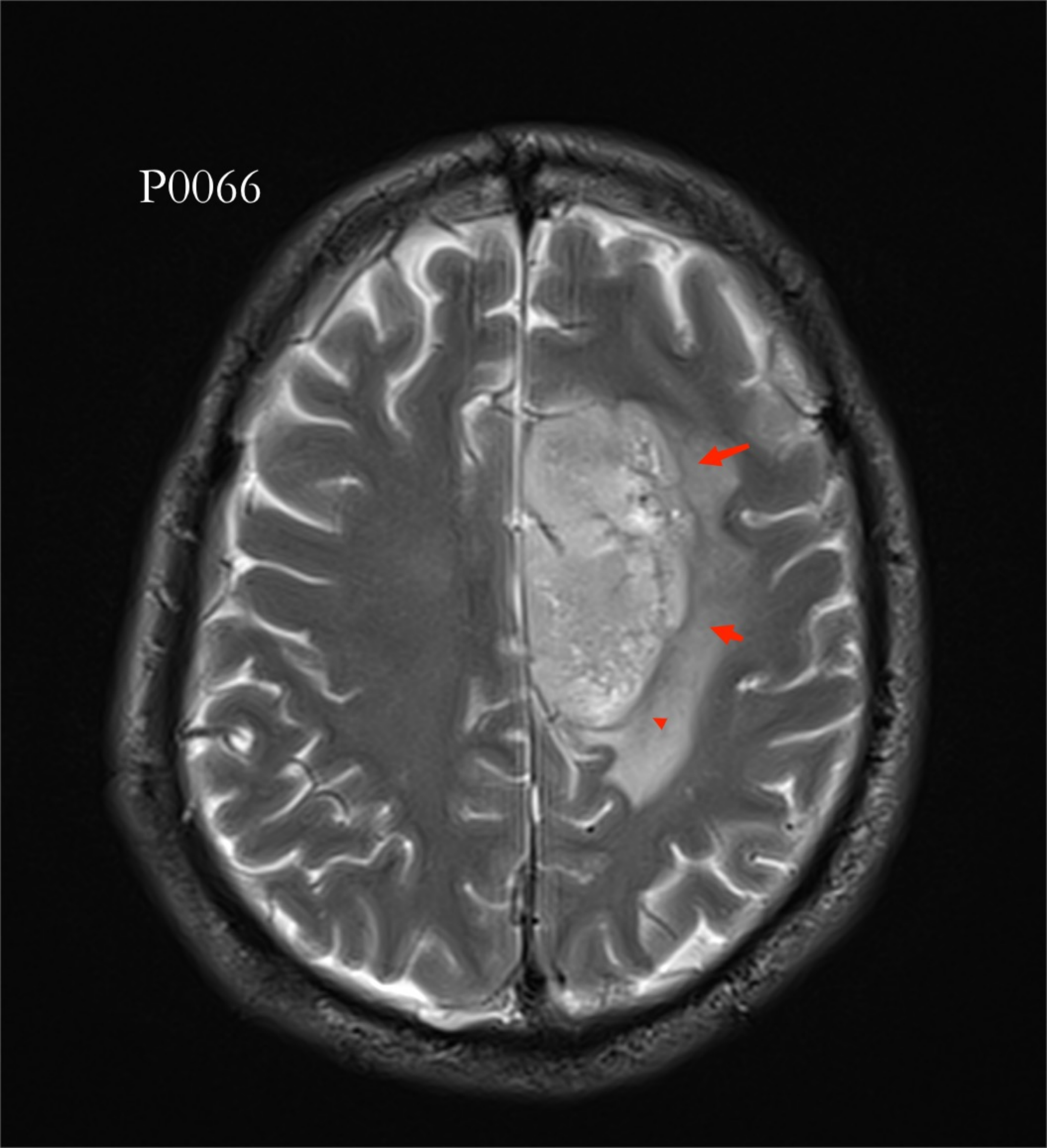
P0066

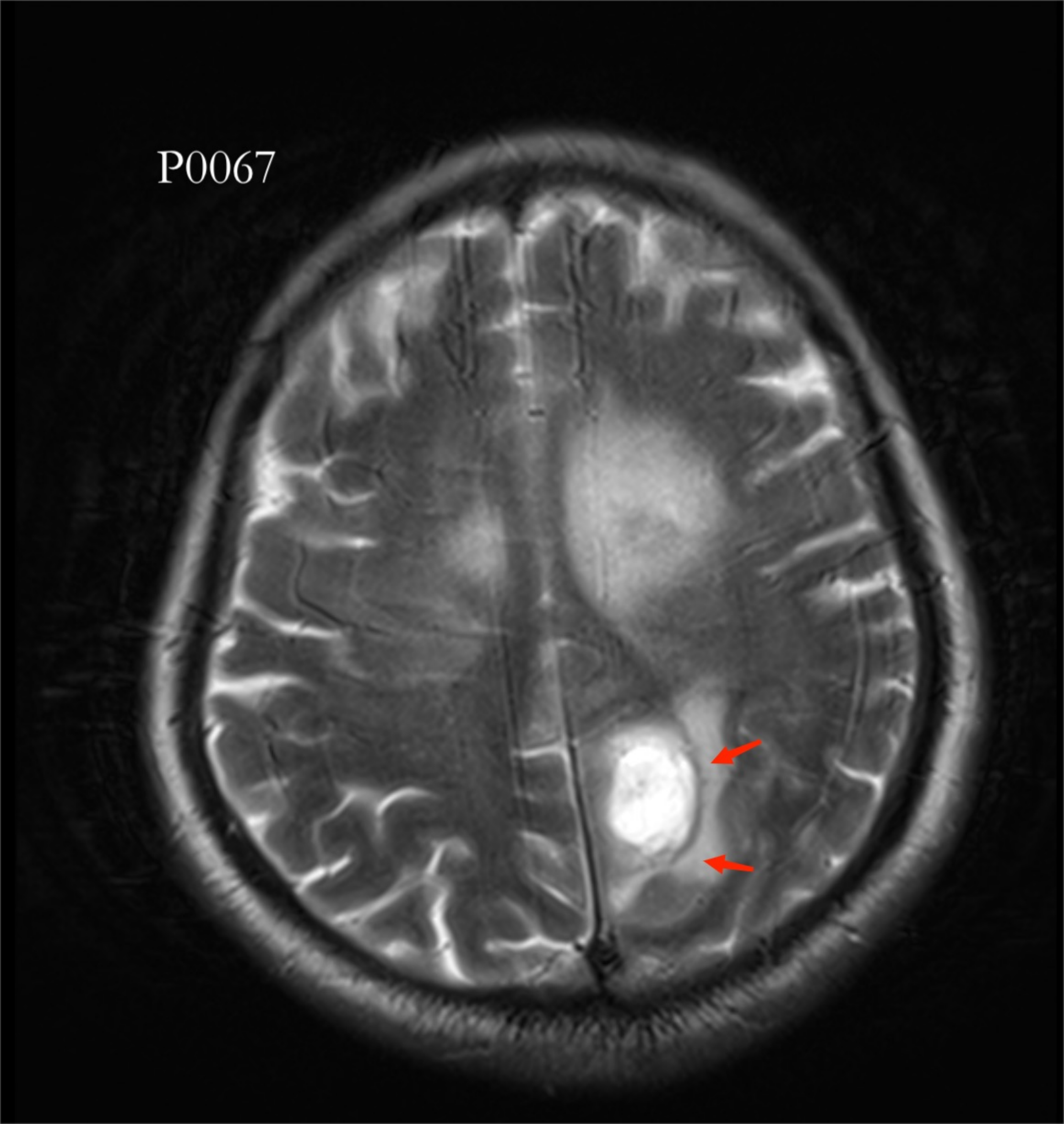
P0067

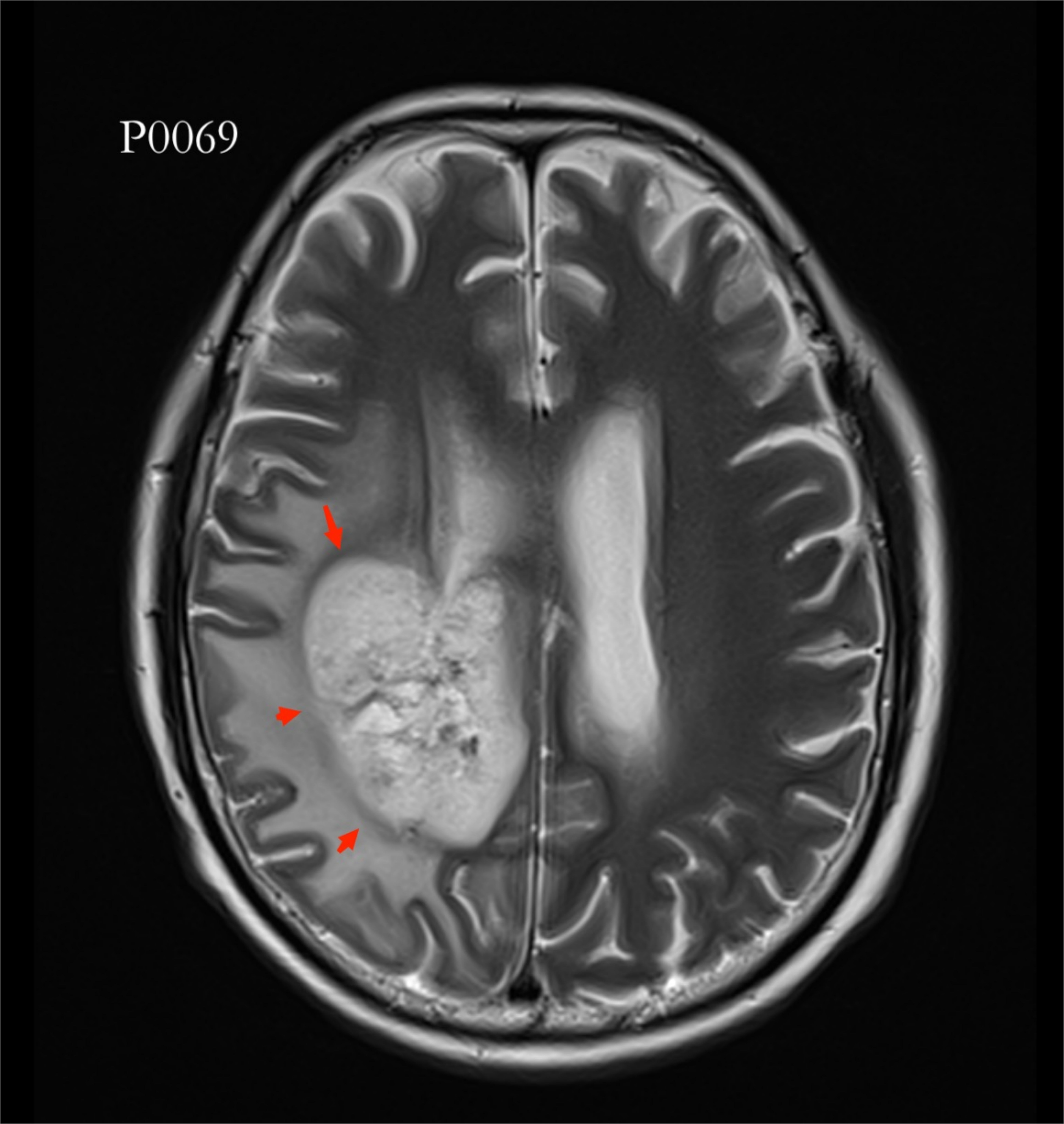
P0069

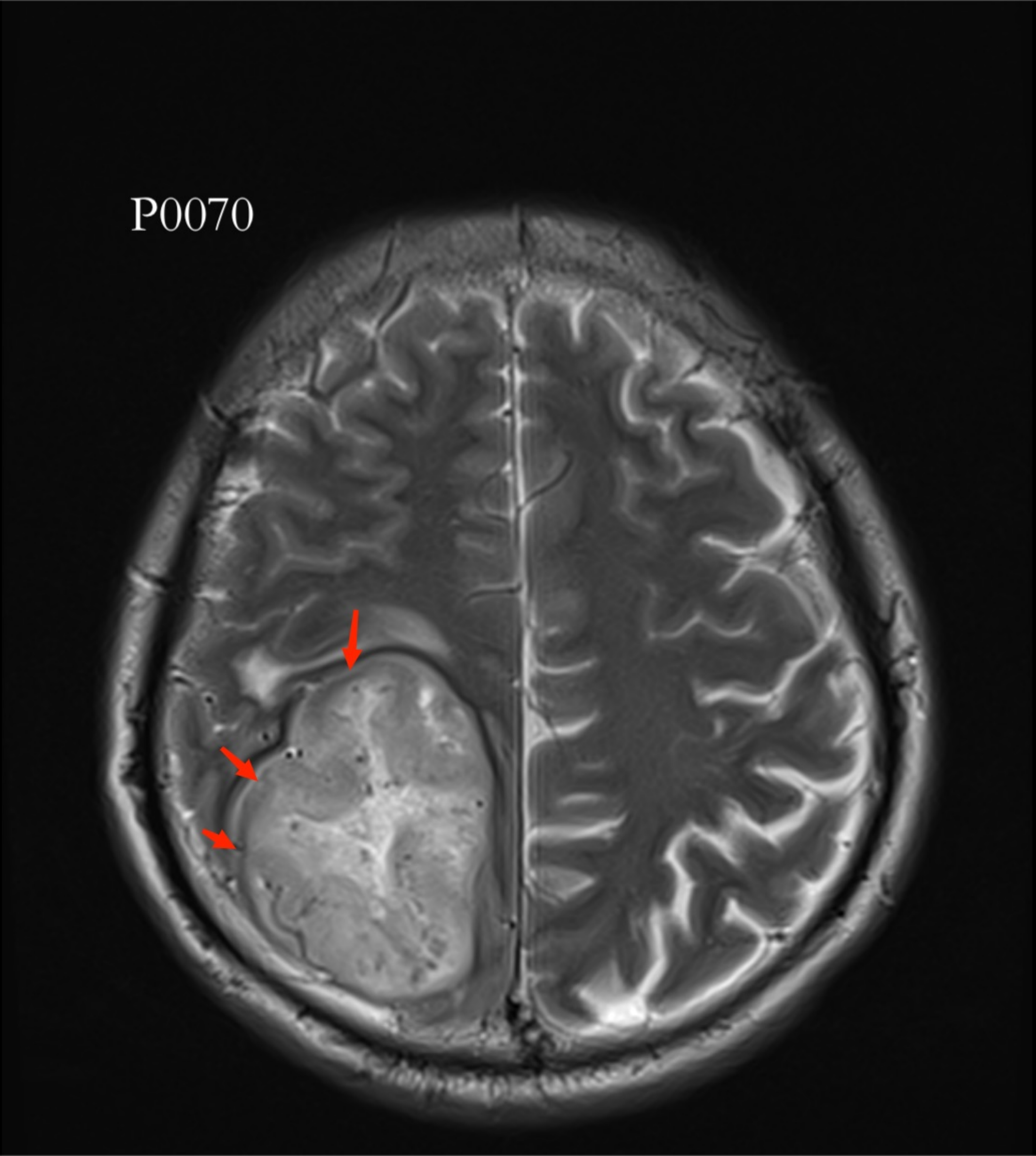
P0070

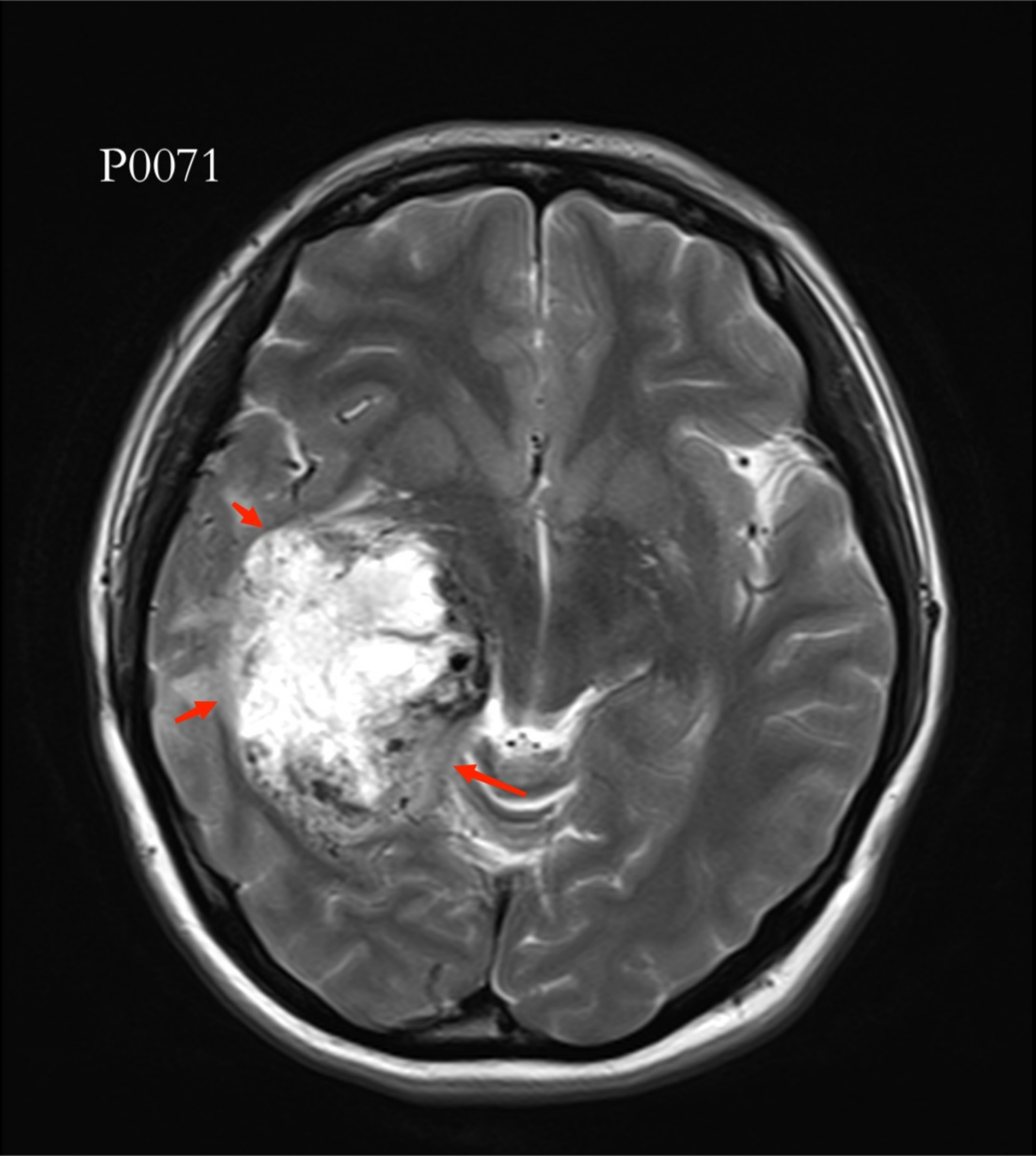
P0071

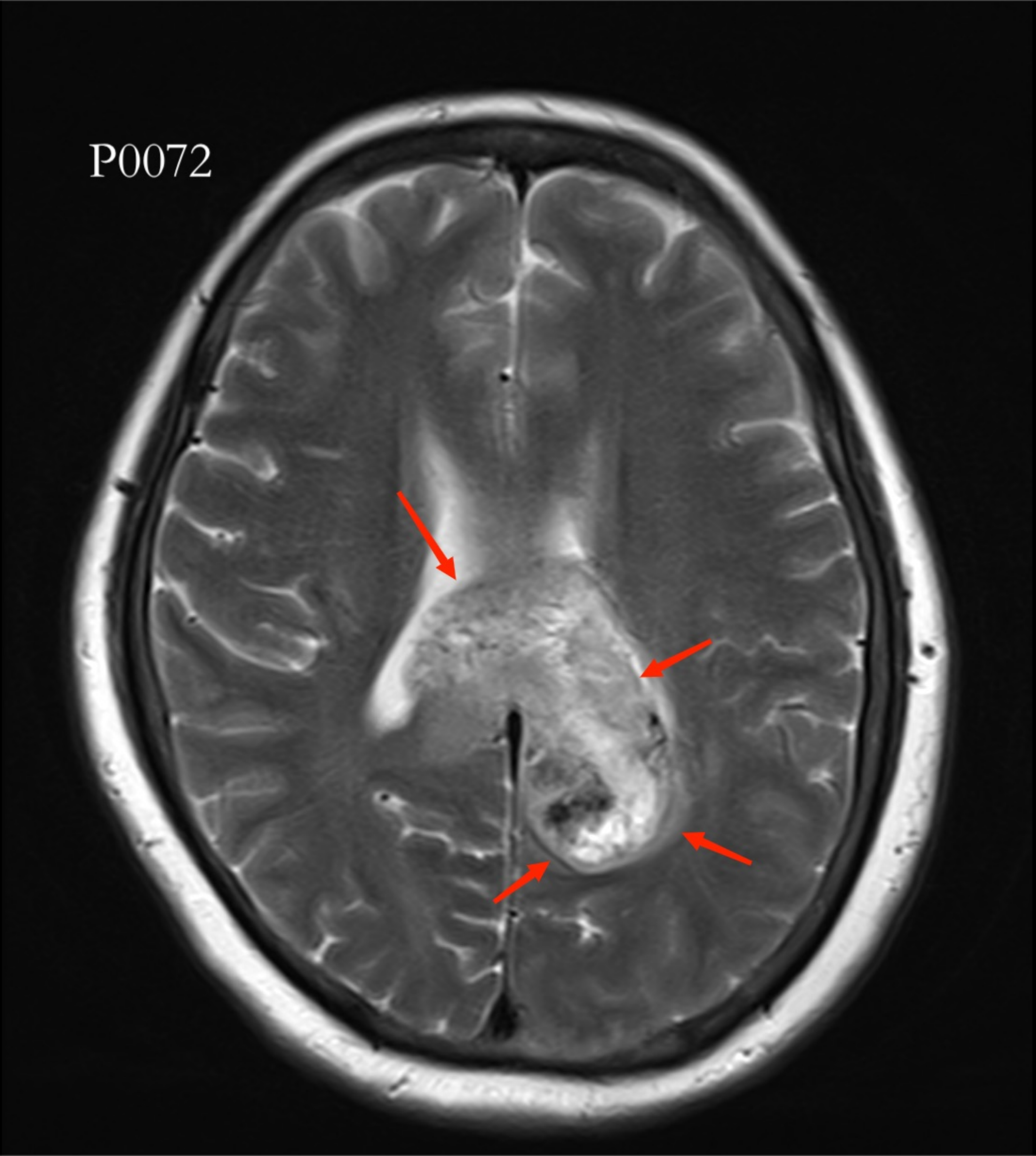
P0072

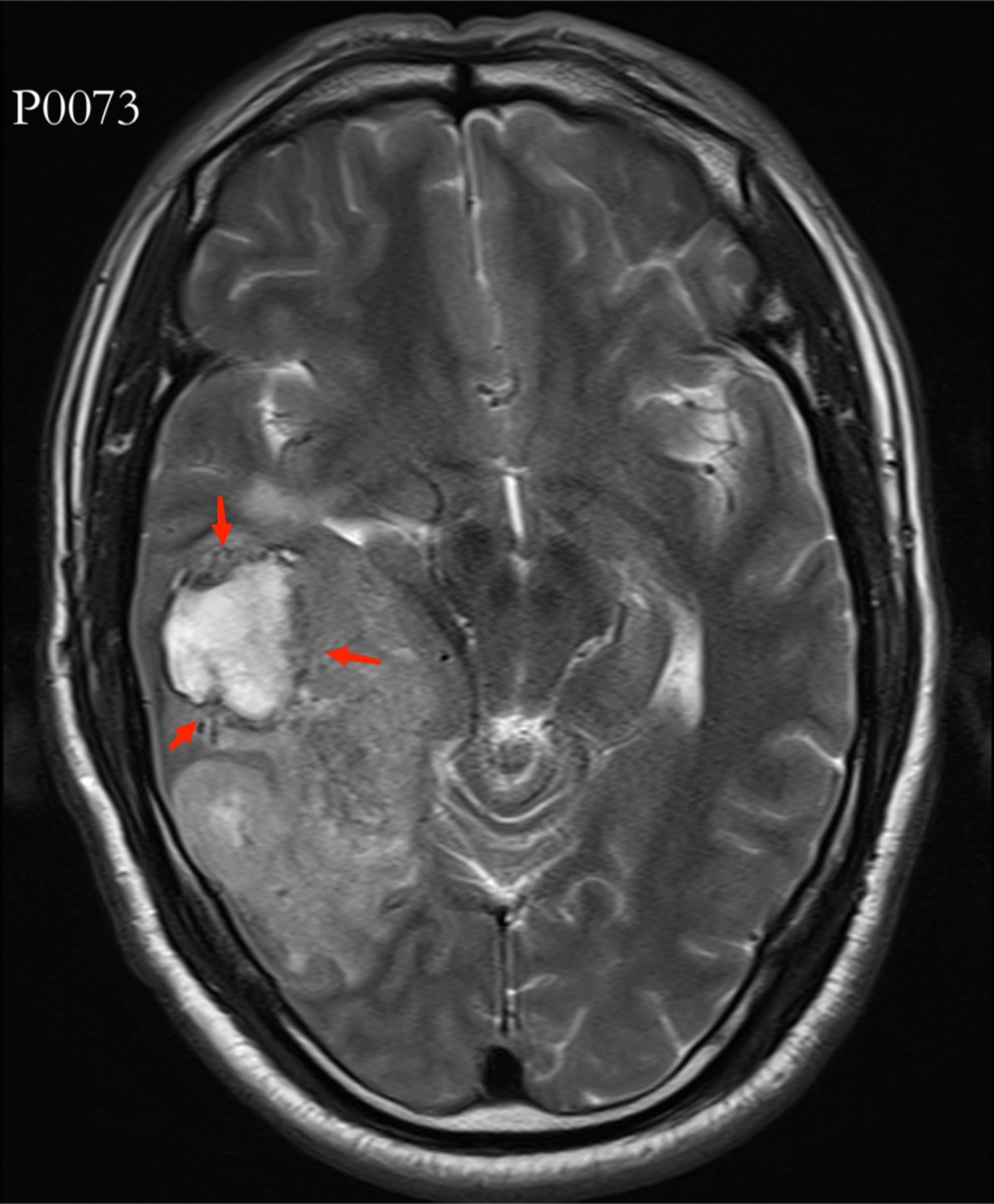
P0073

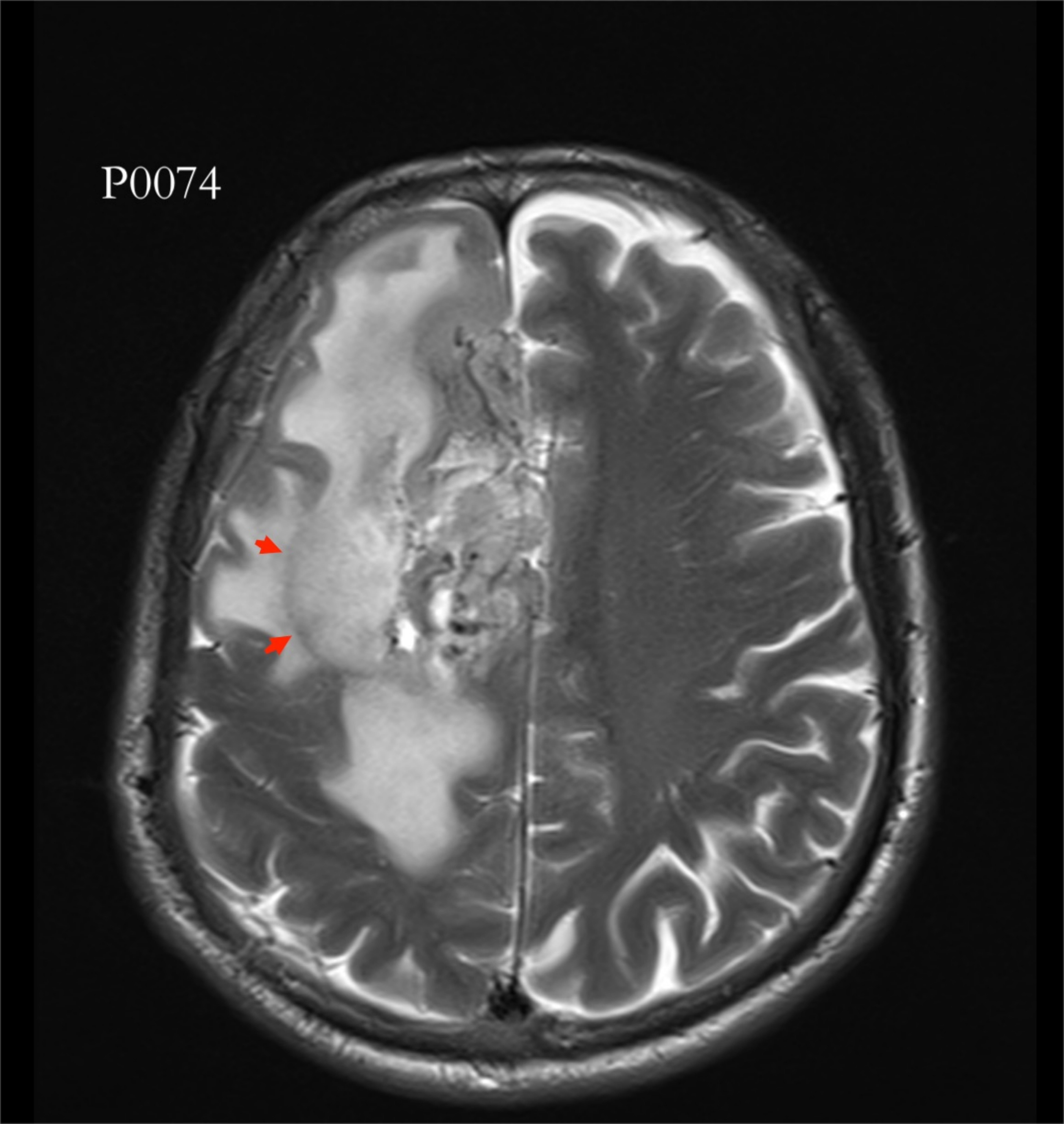
P0074

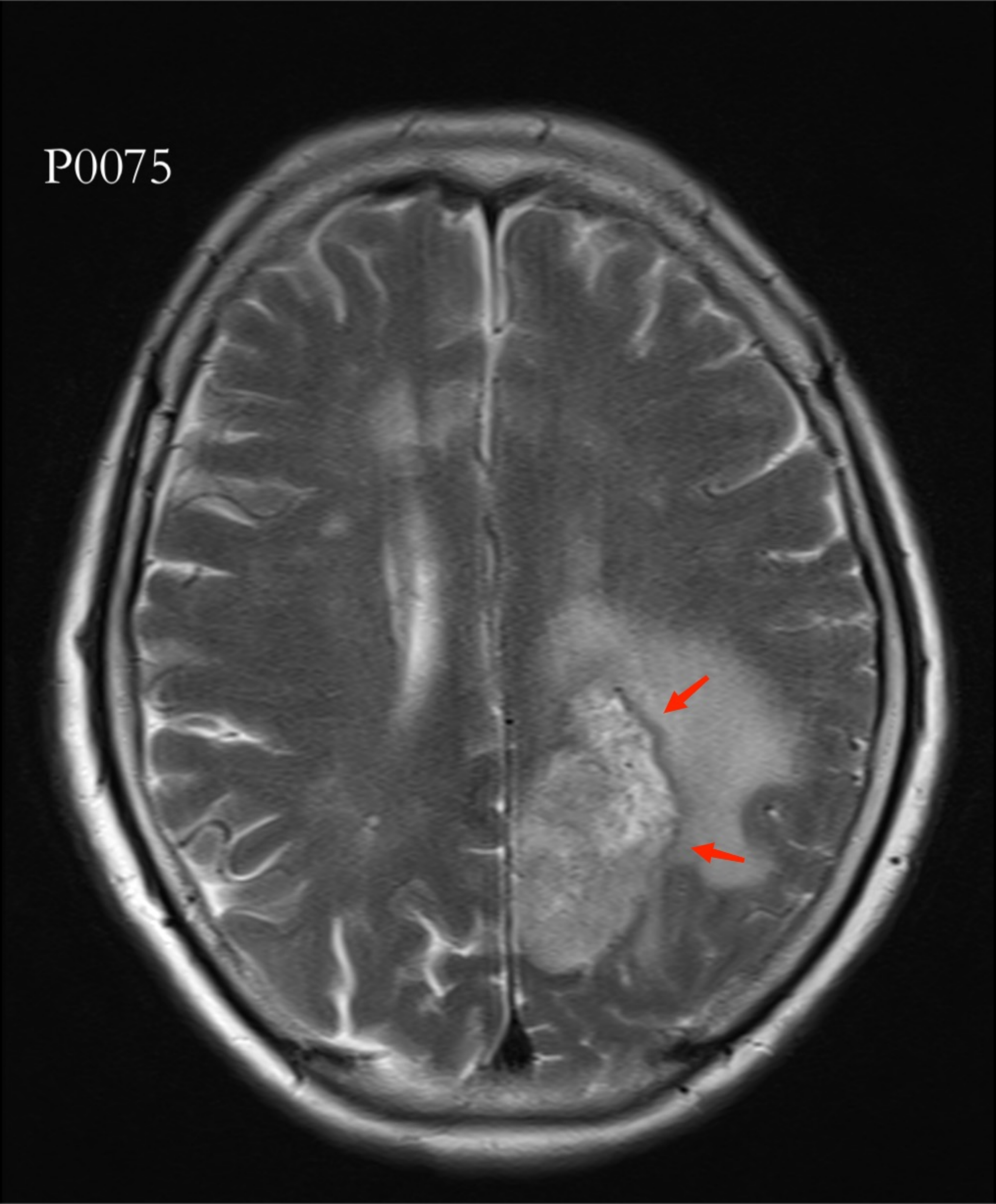
P0075

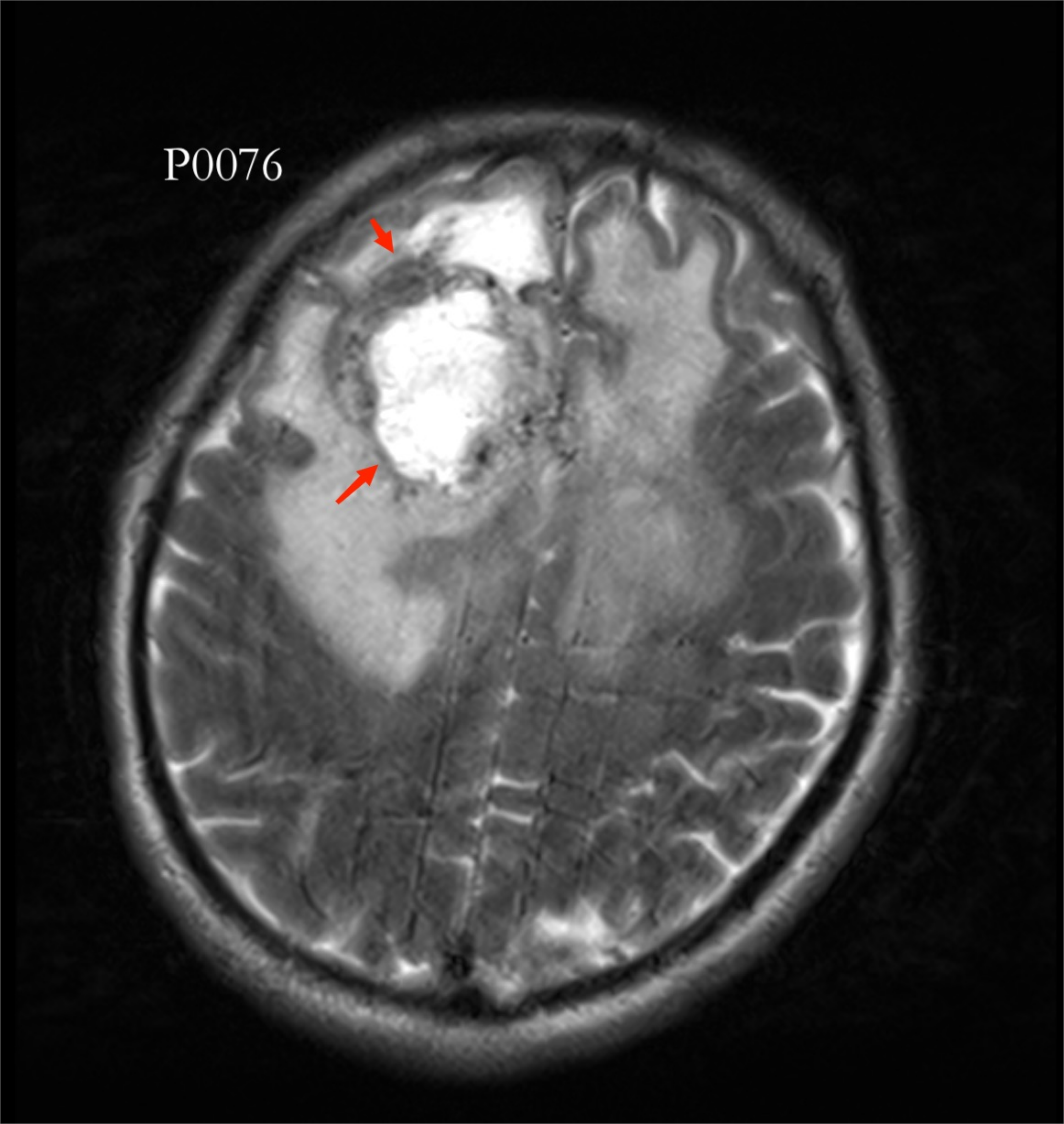
P0076

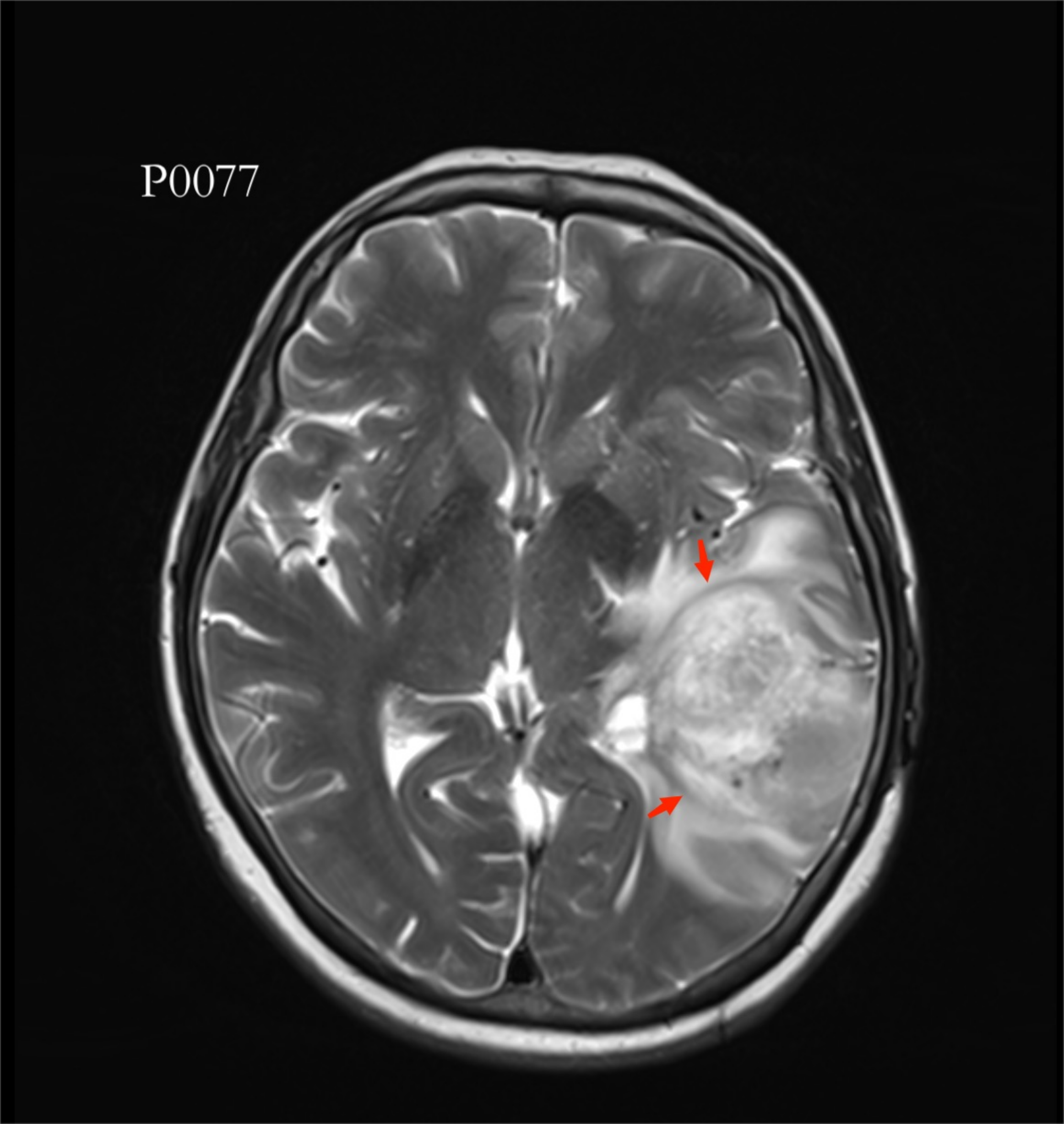
P0077

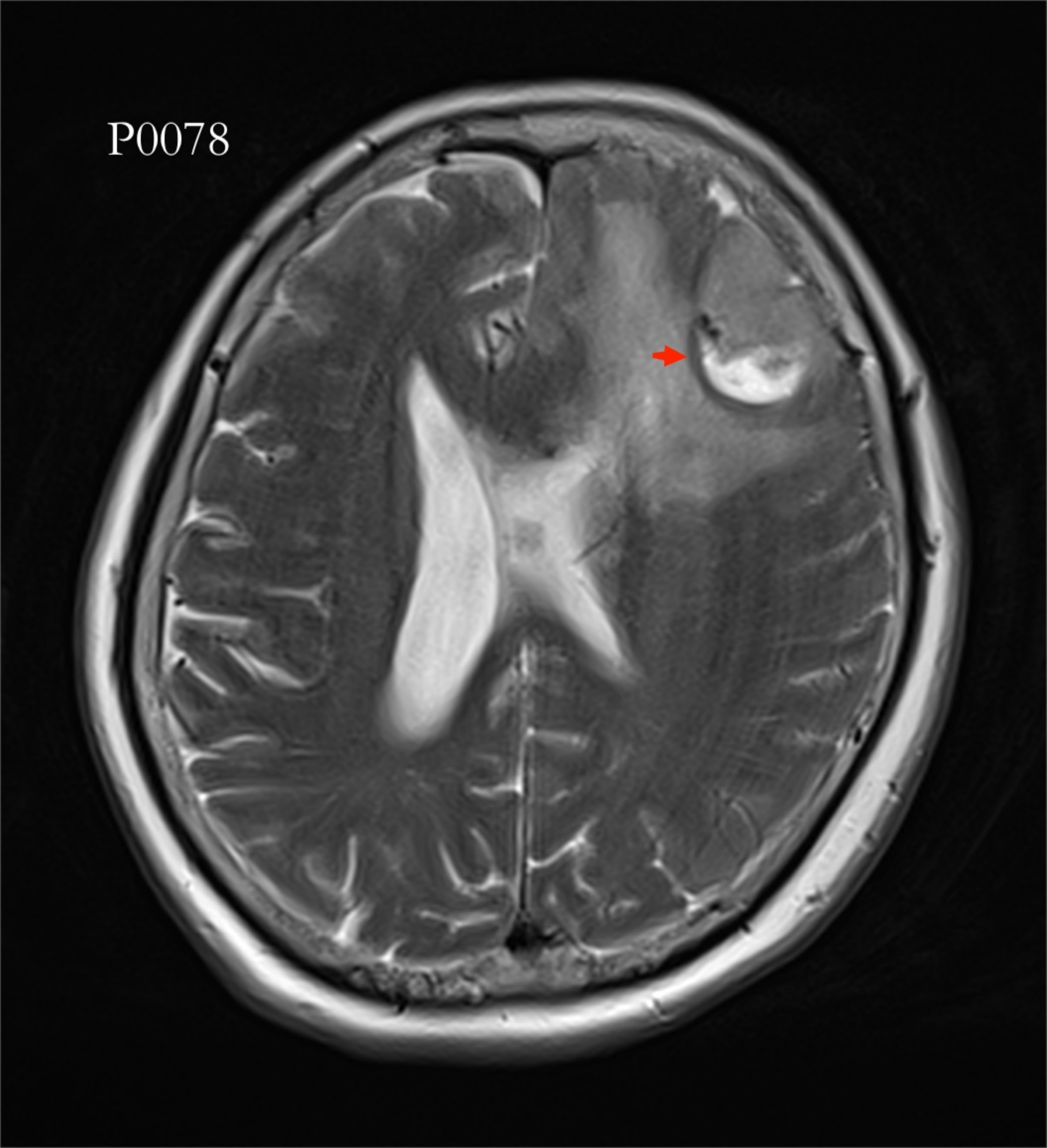
P0078

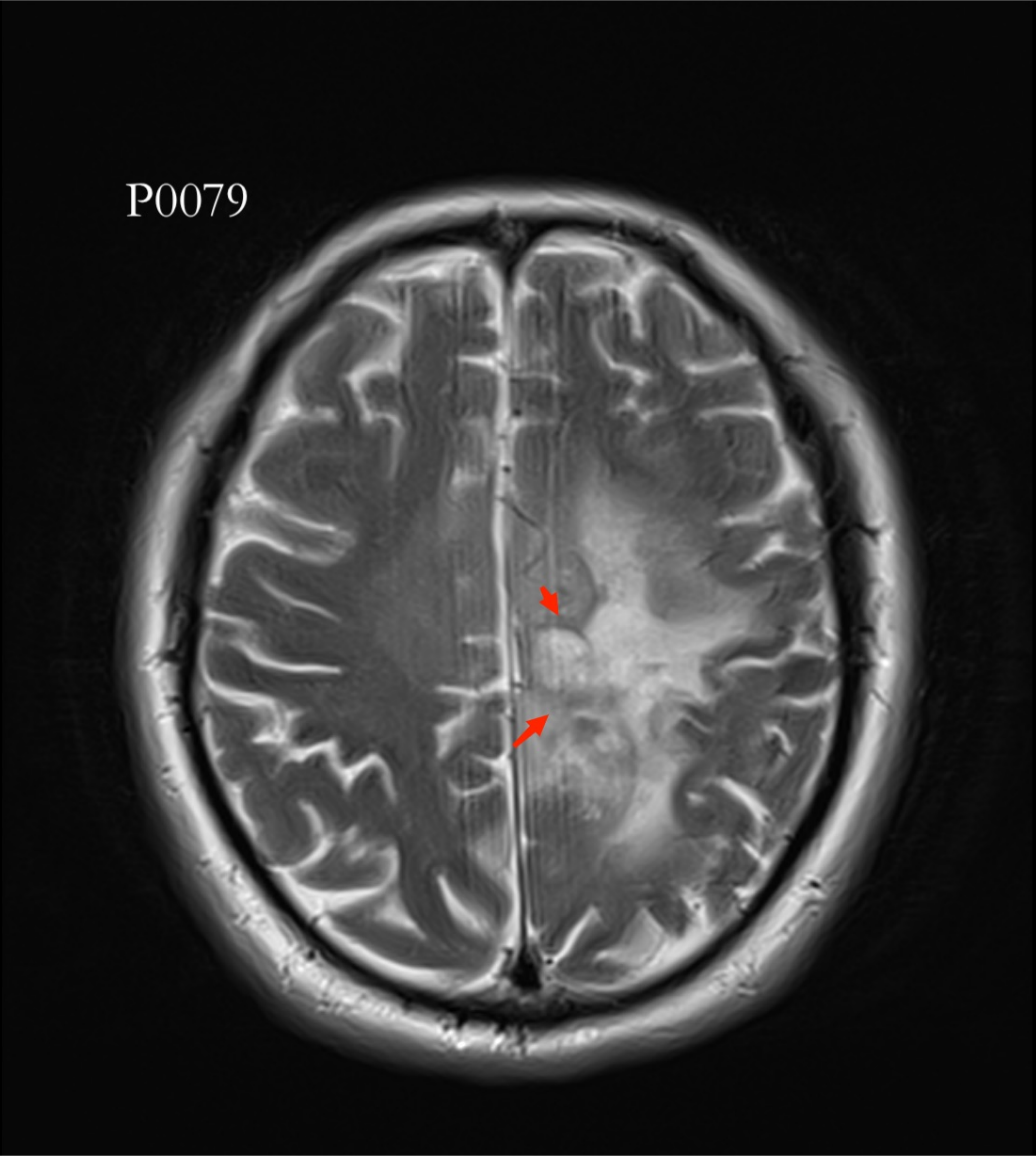
P0079

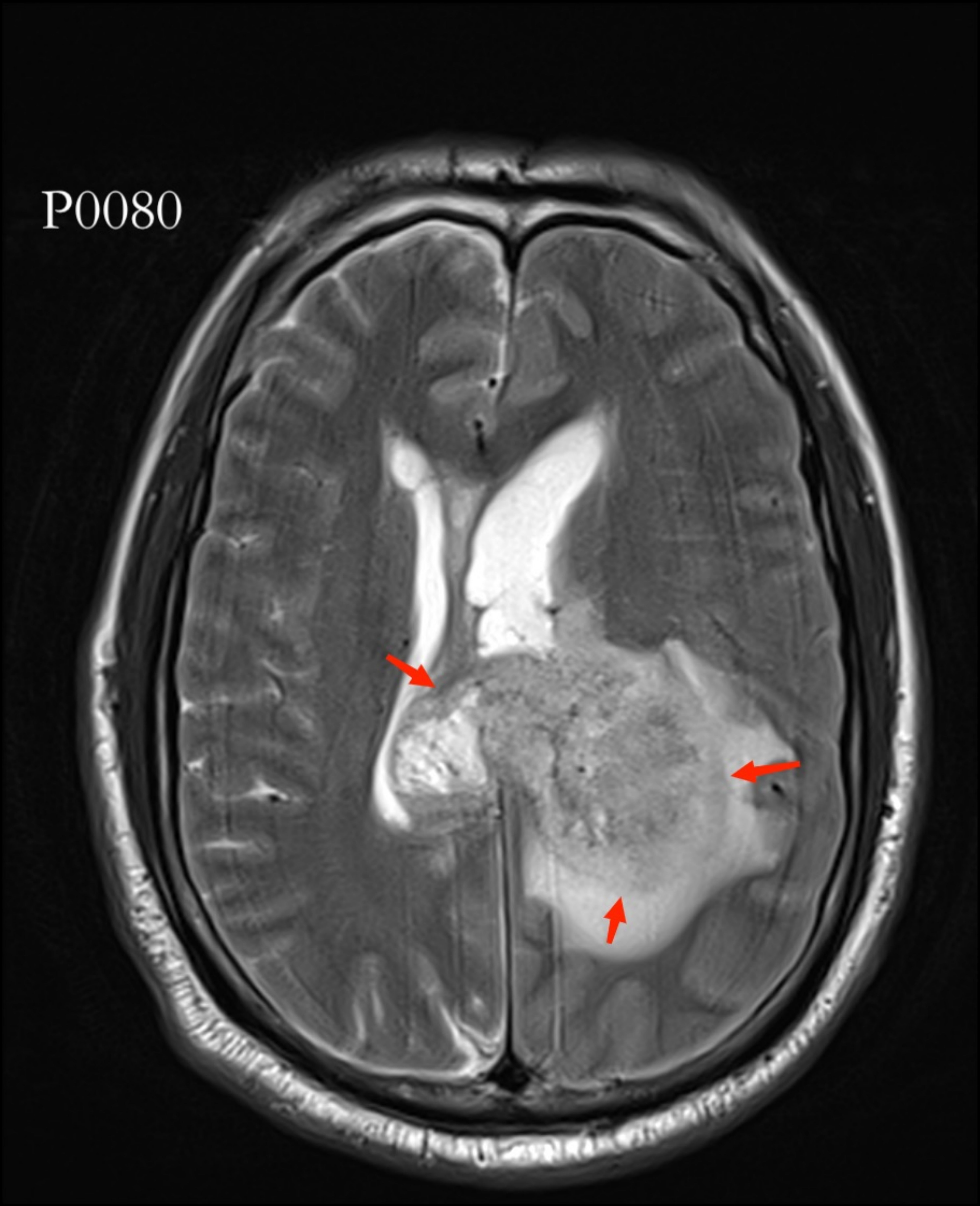
P0080

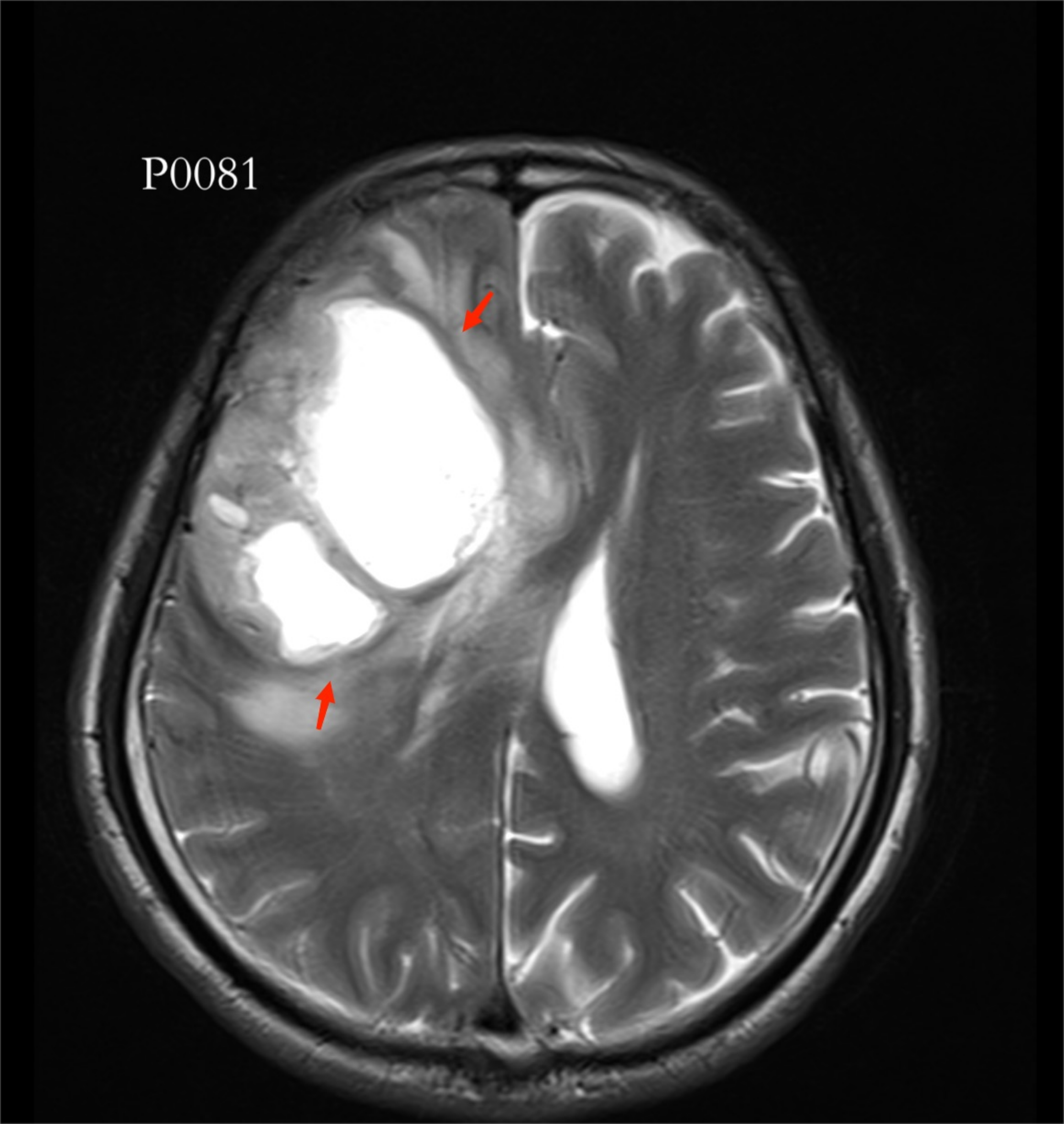
P0081

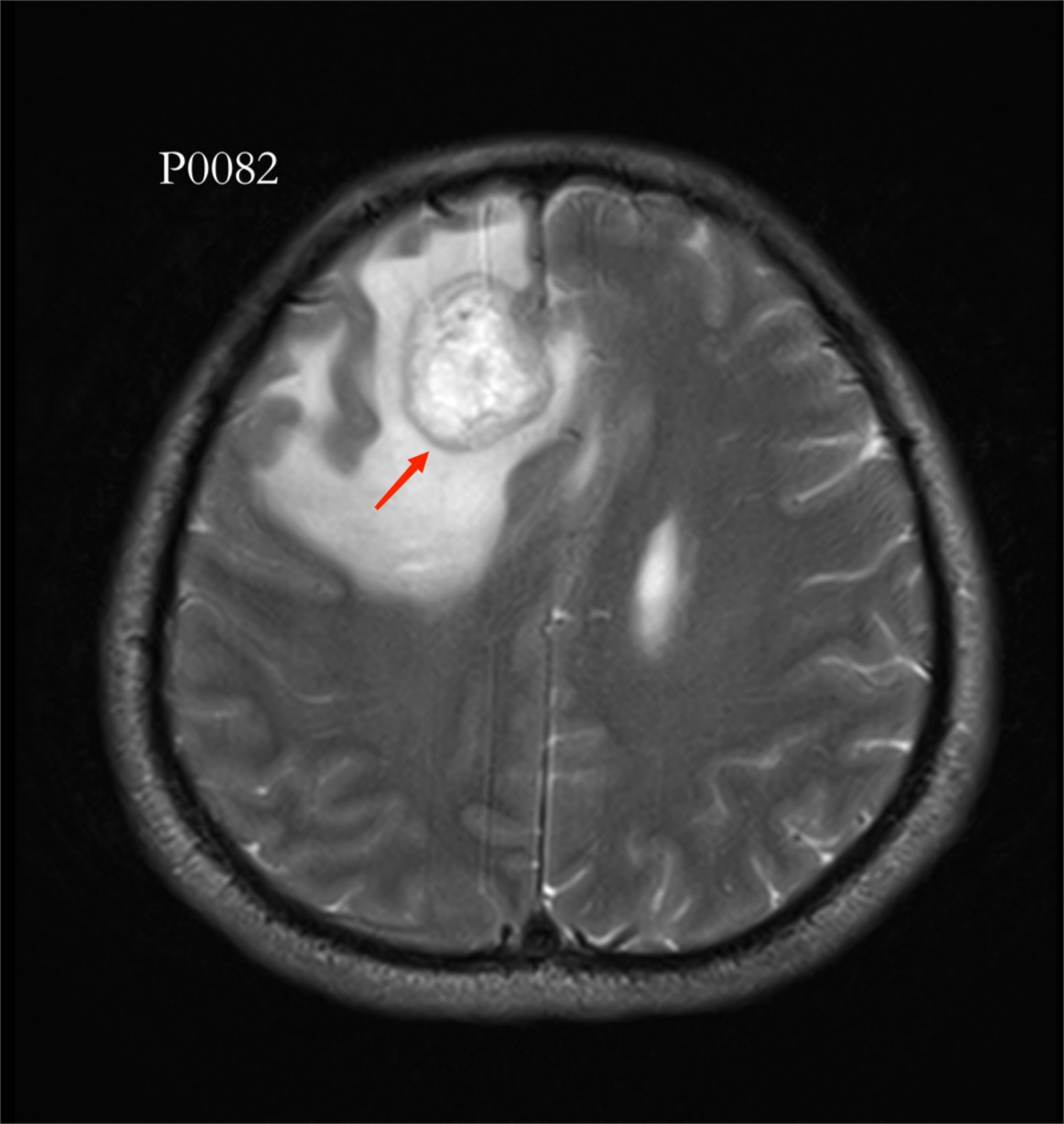
P0082

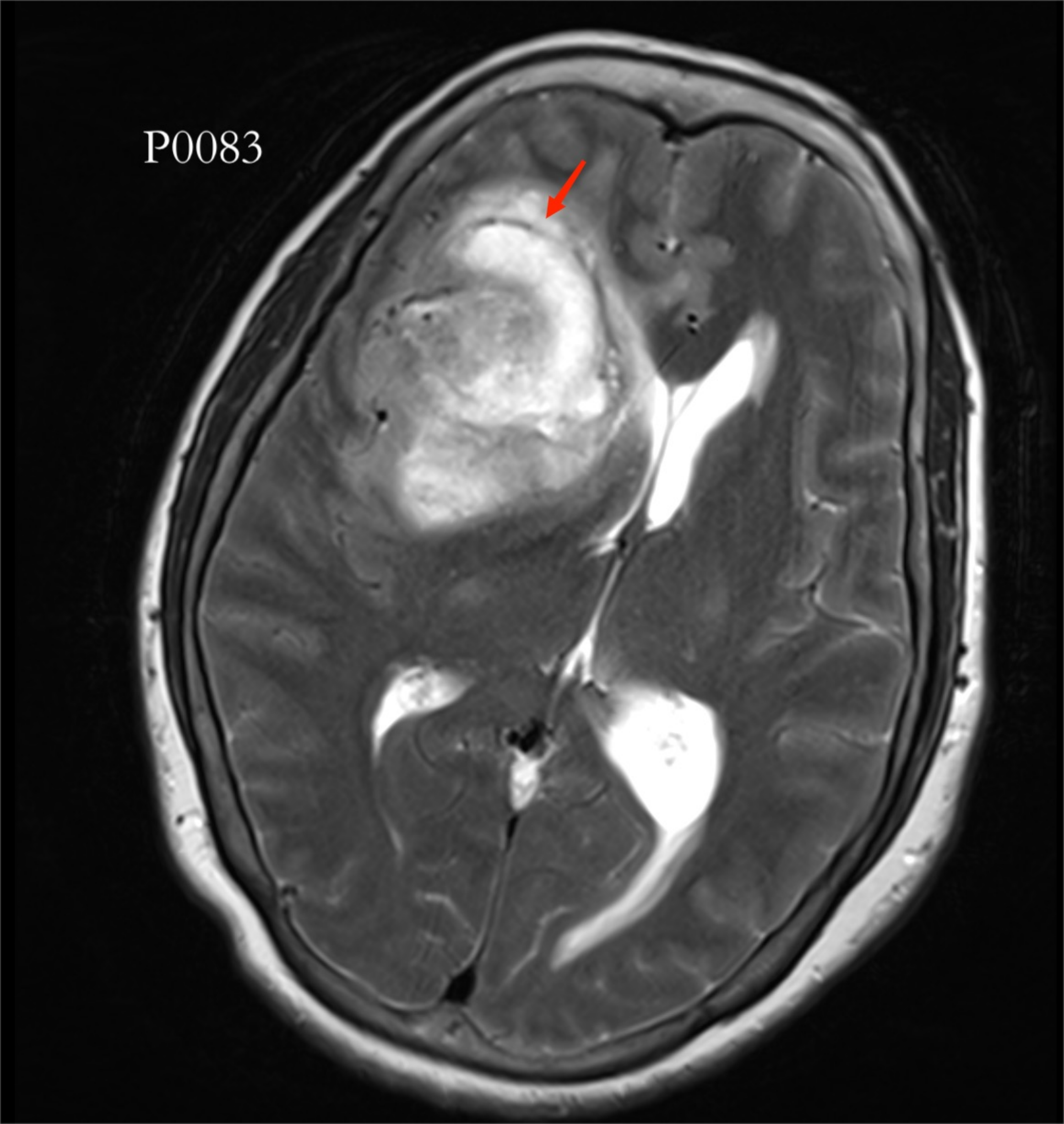
P0083

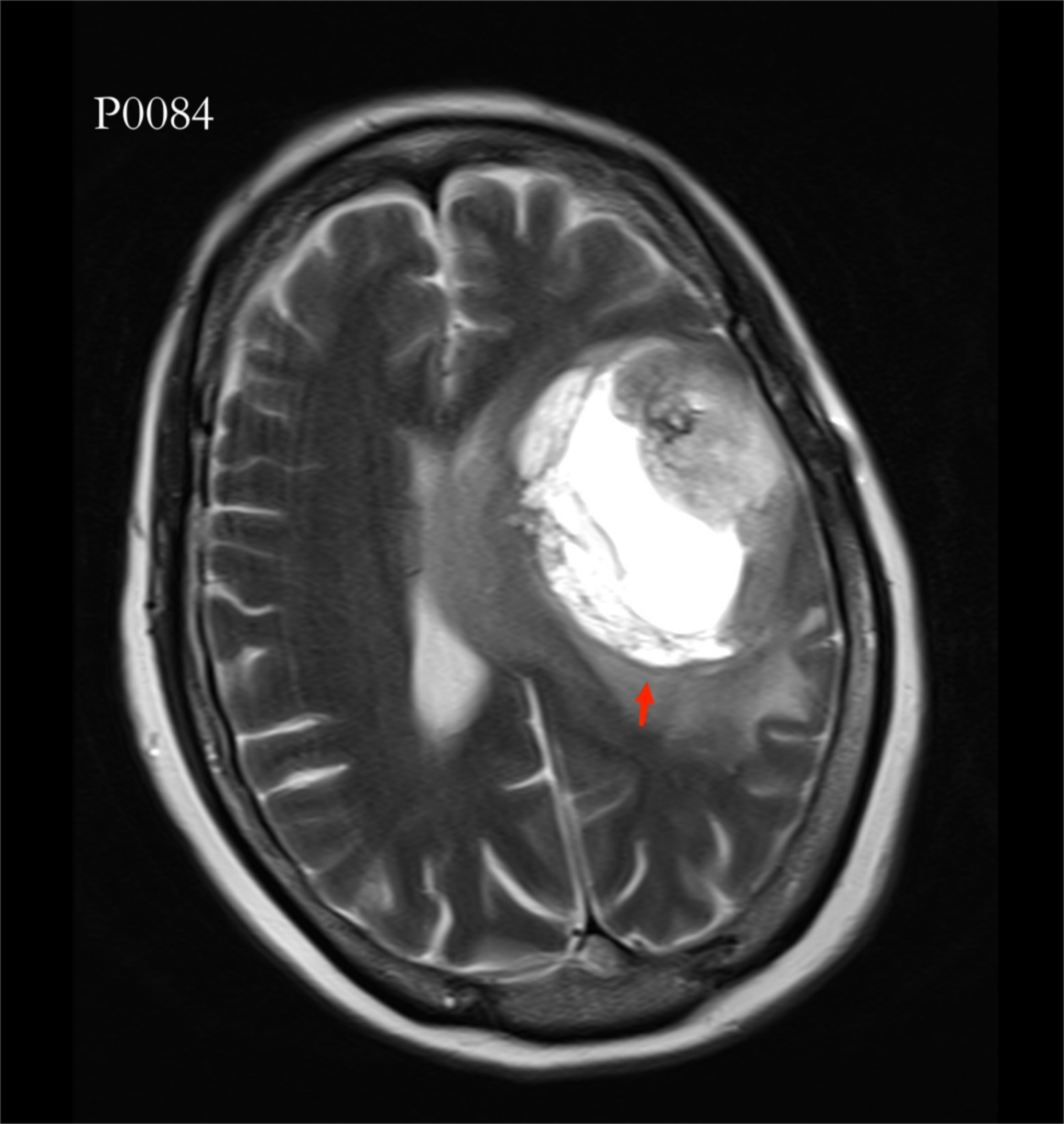
P0084

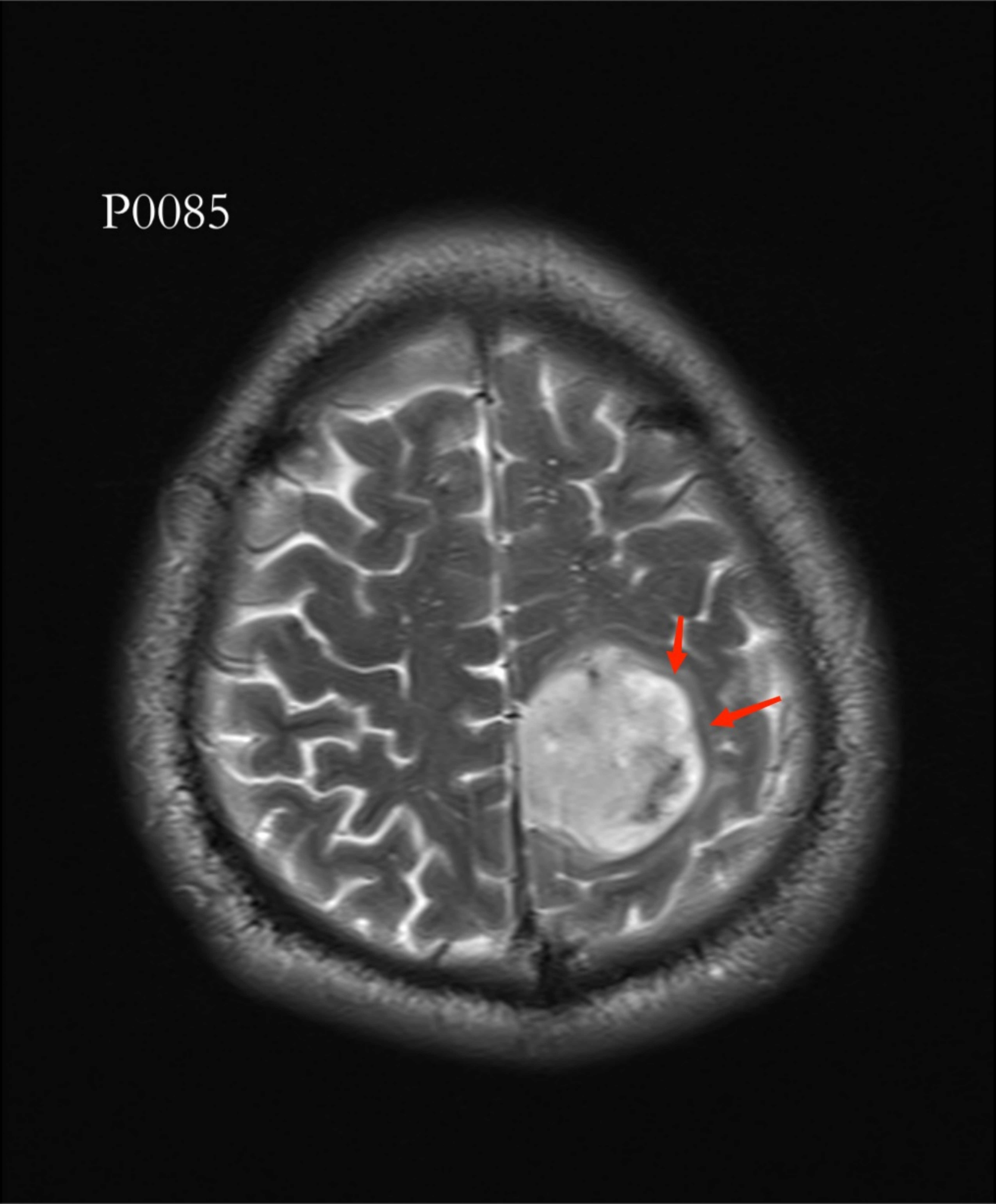
P0085

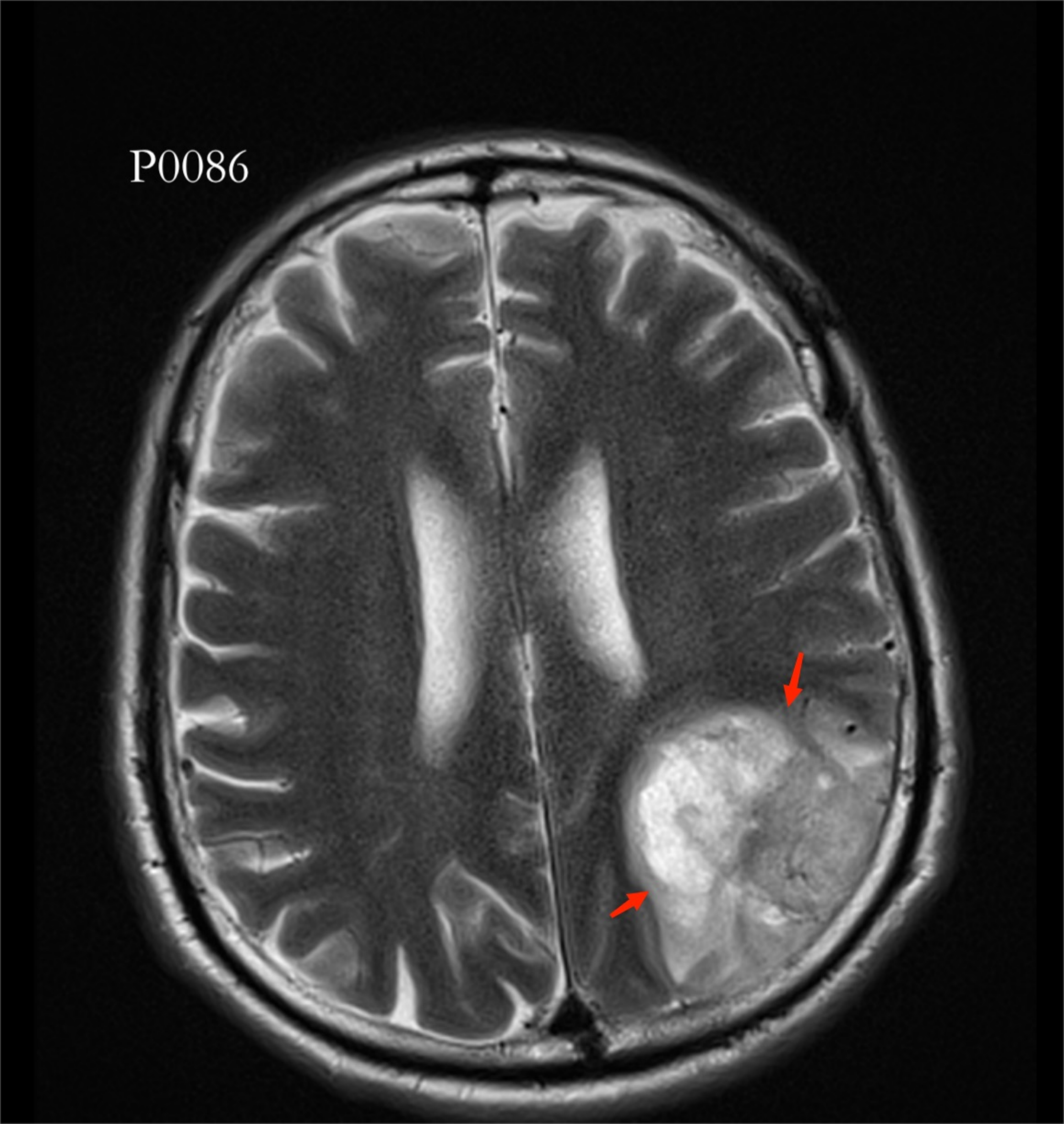
P0086

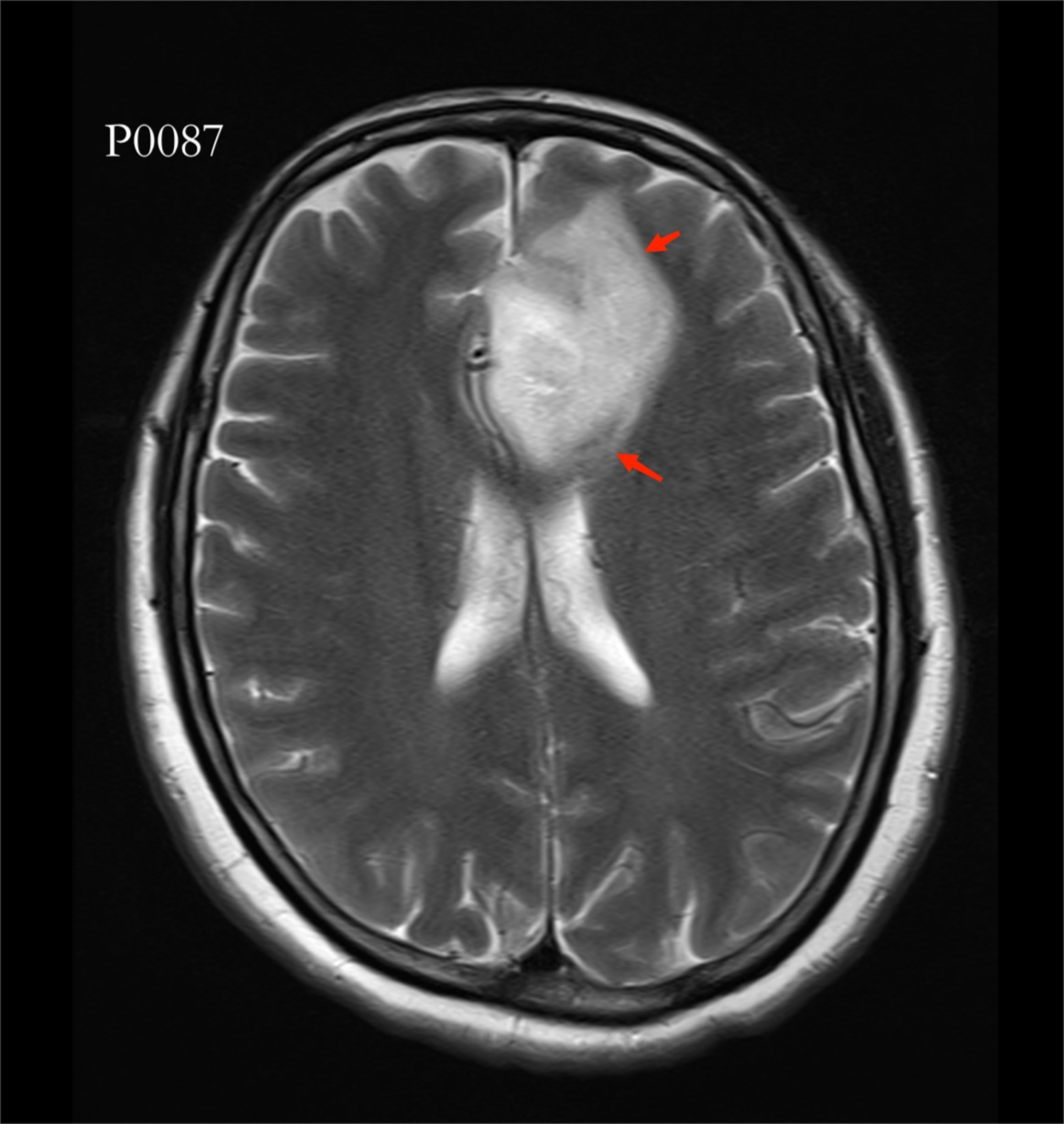
P0087

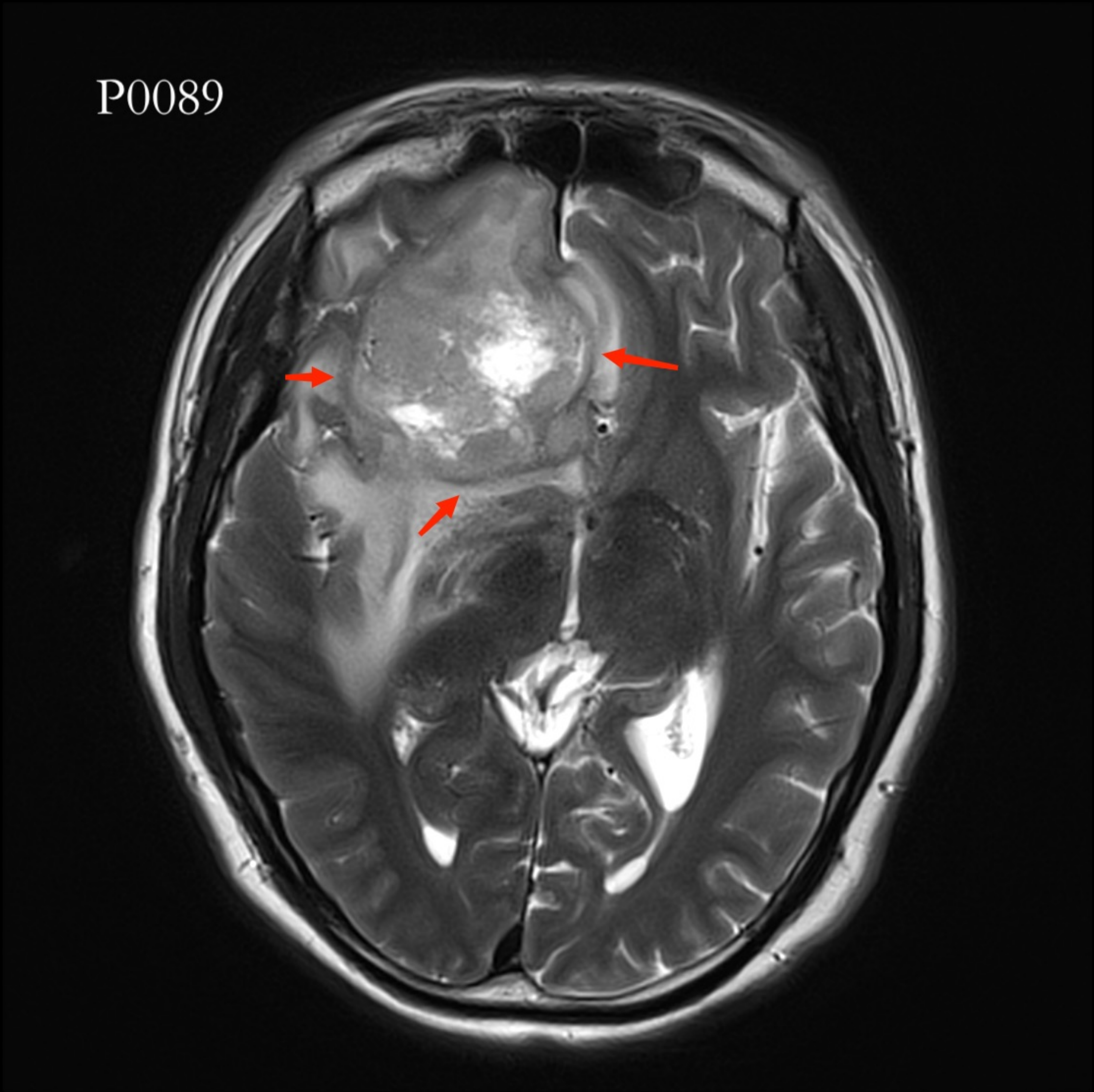
P0089

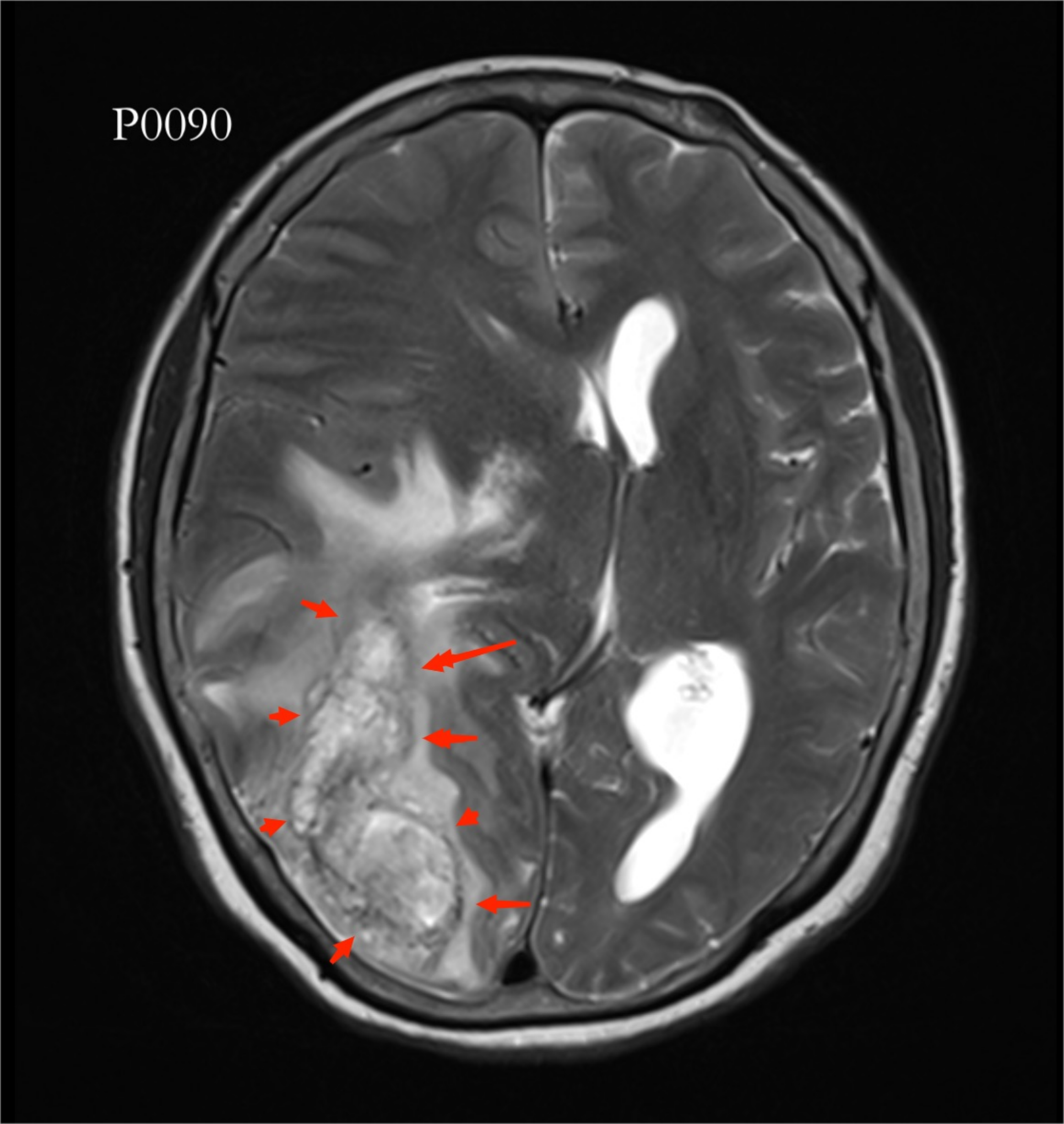
P0090

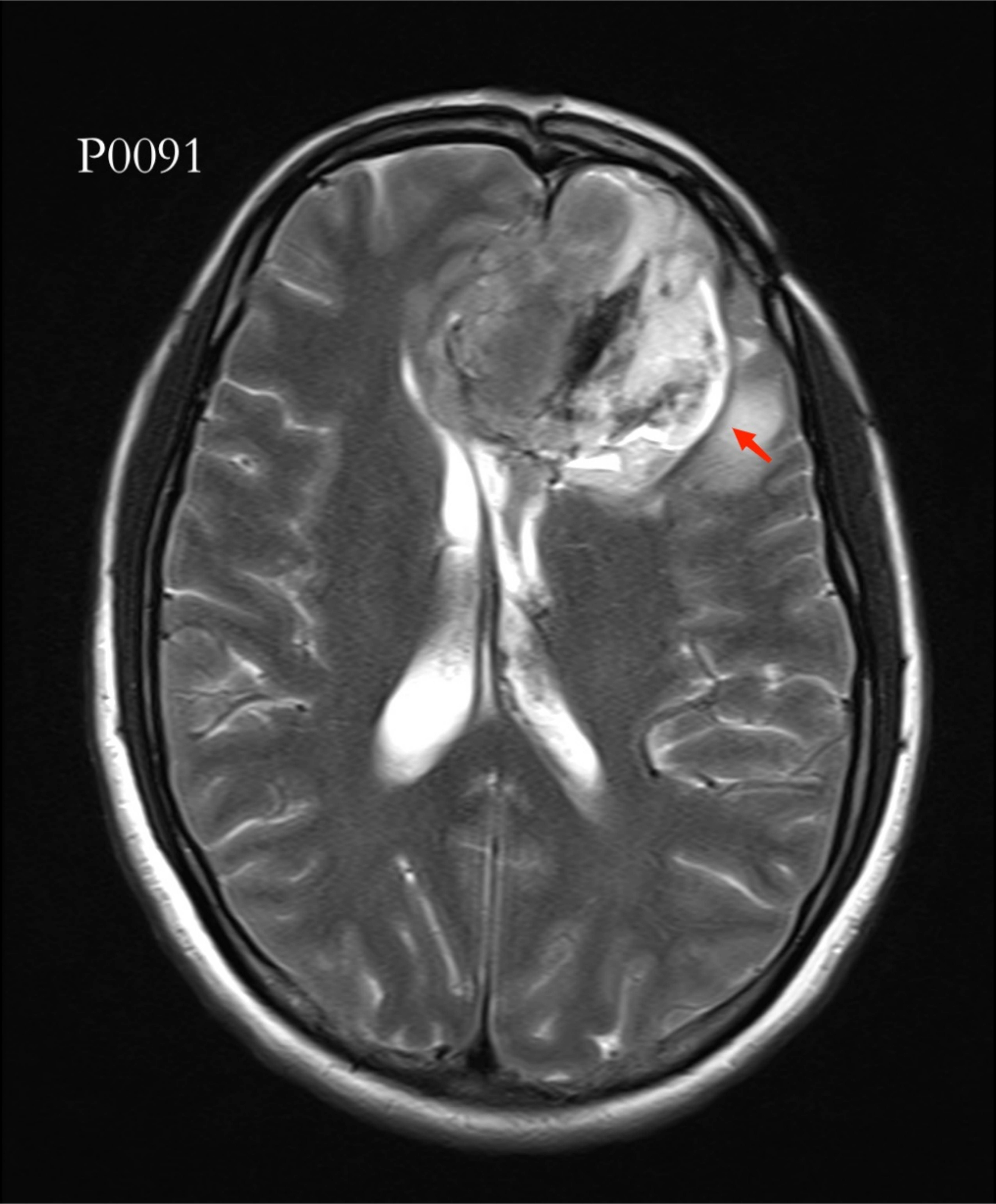
P0091

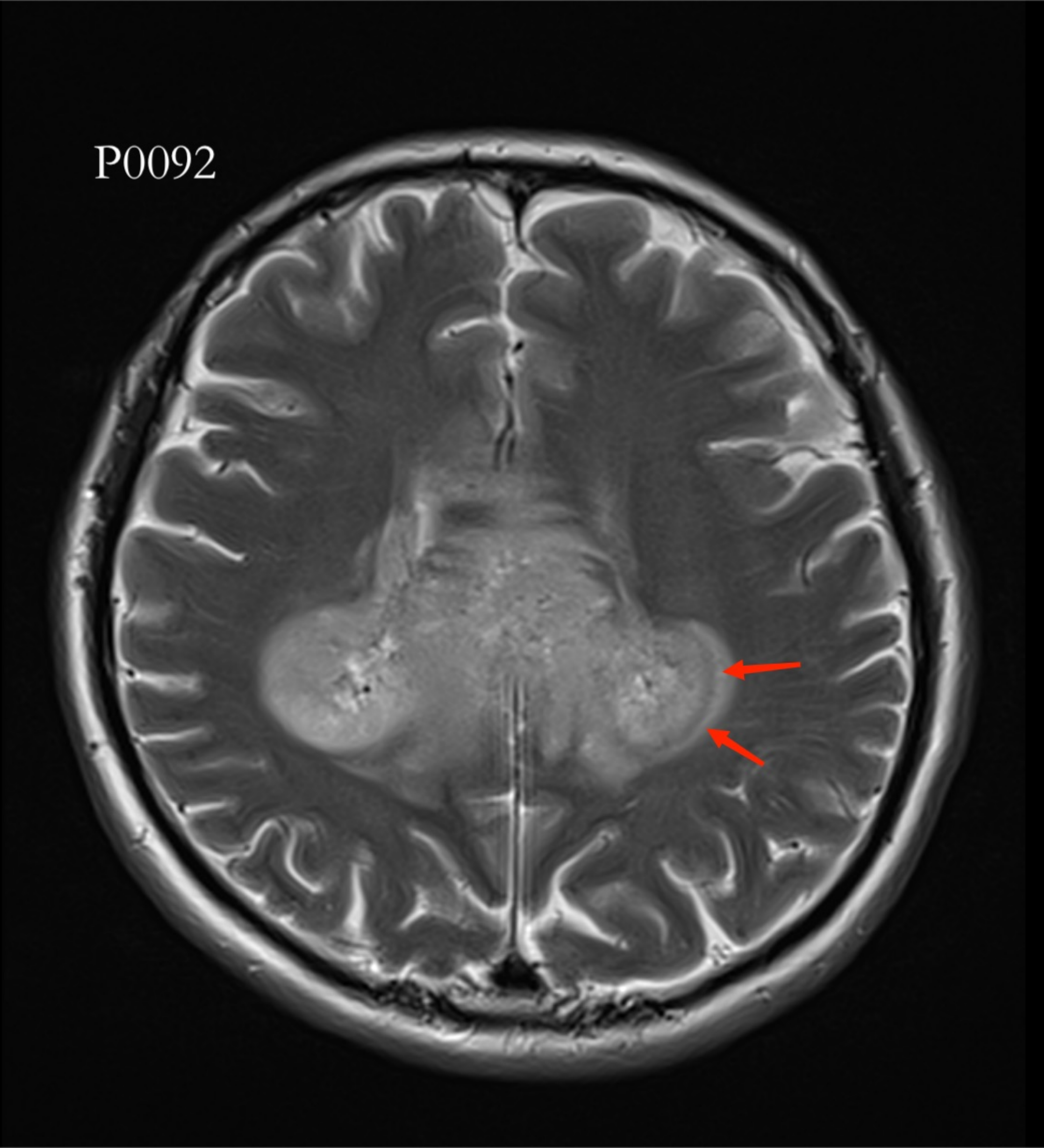
P0092

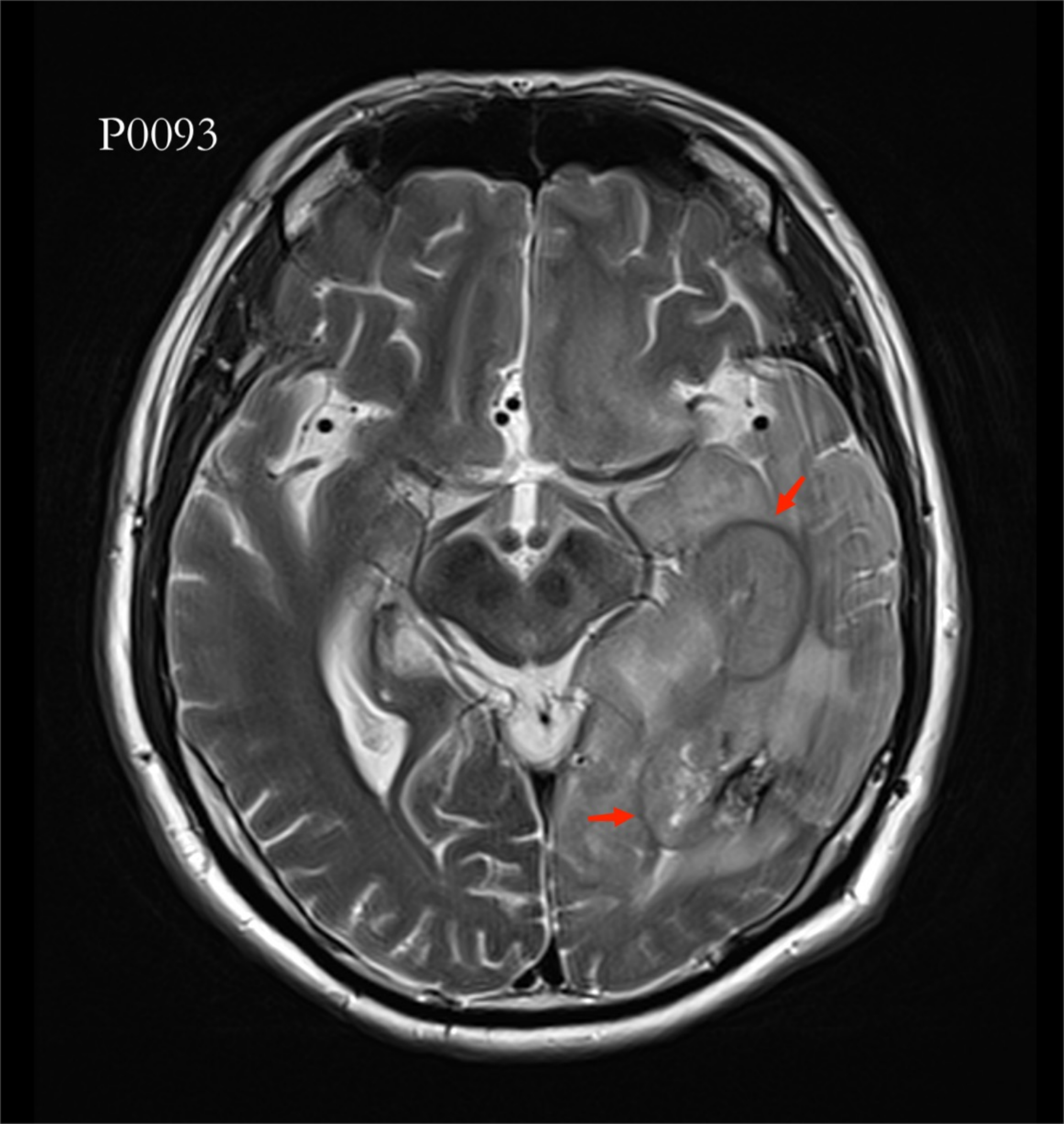
P0093

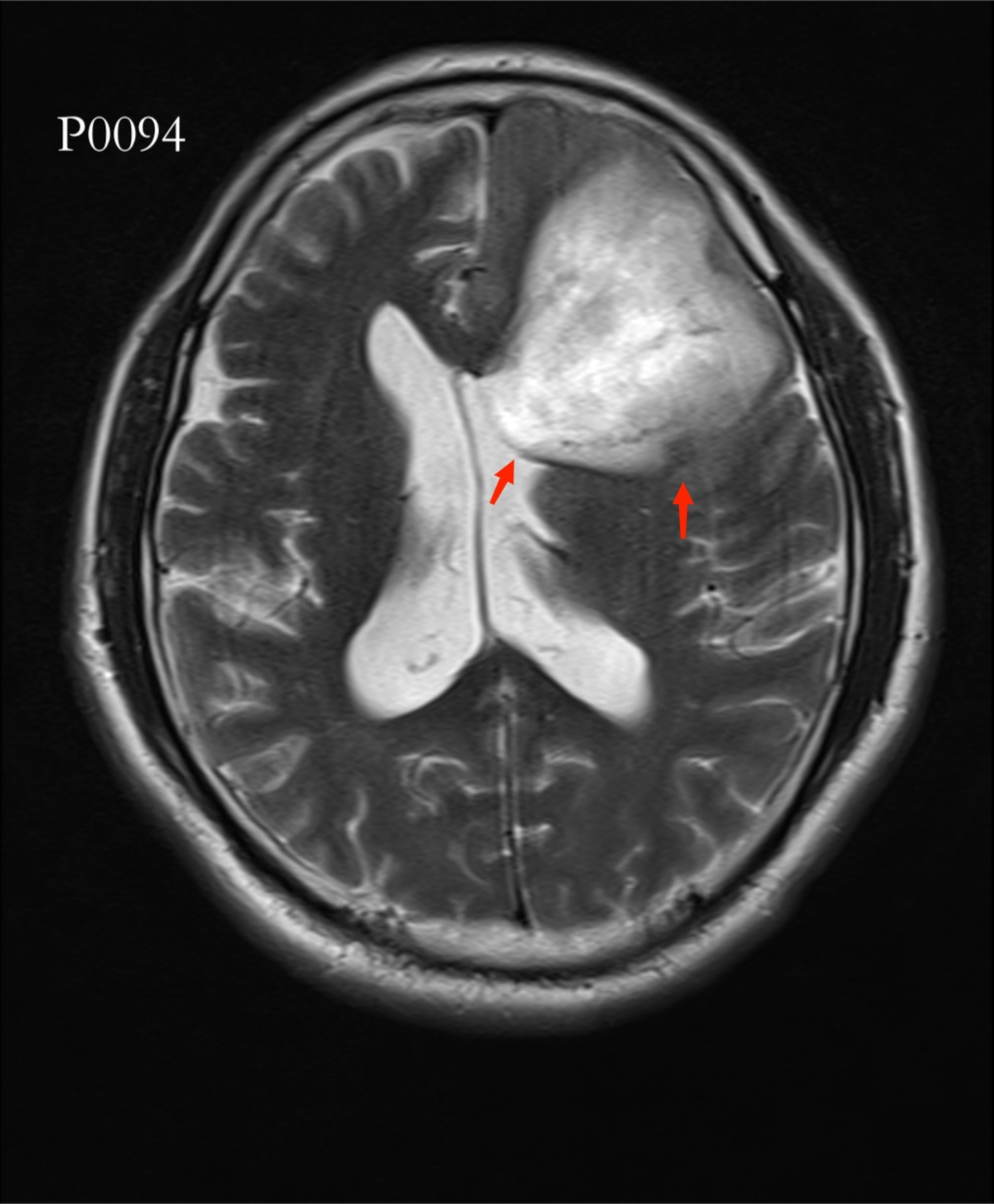
P0094

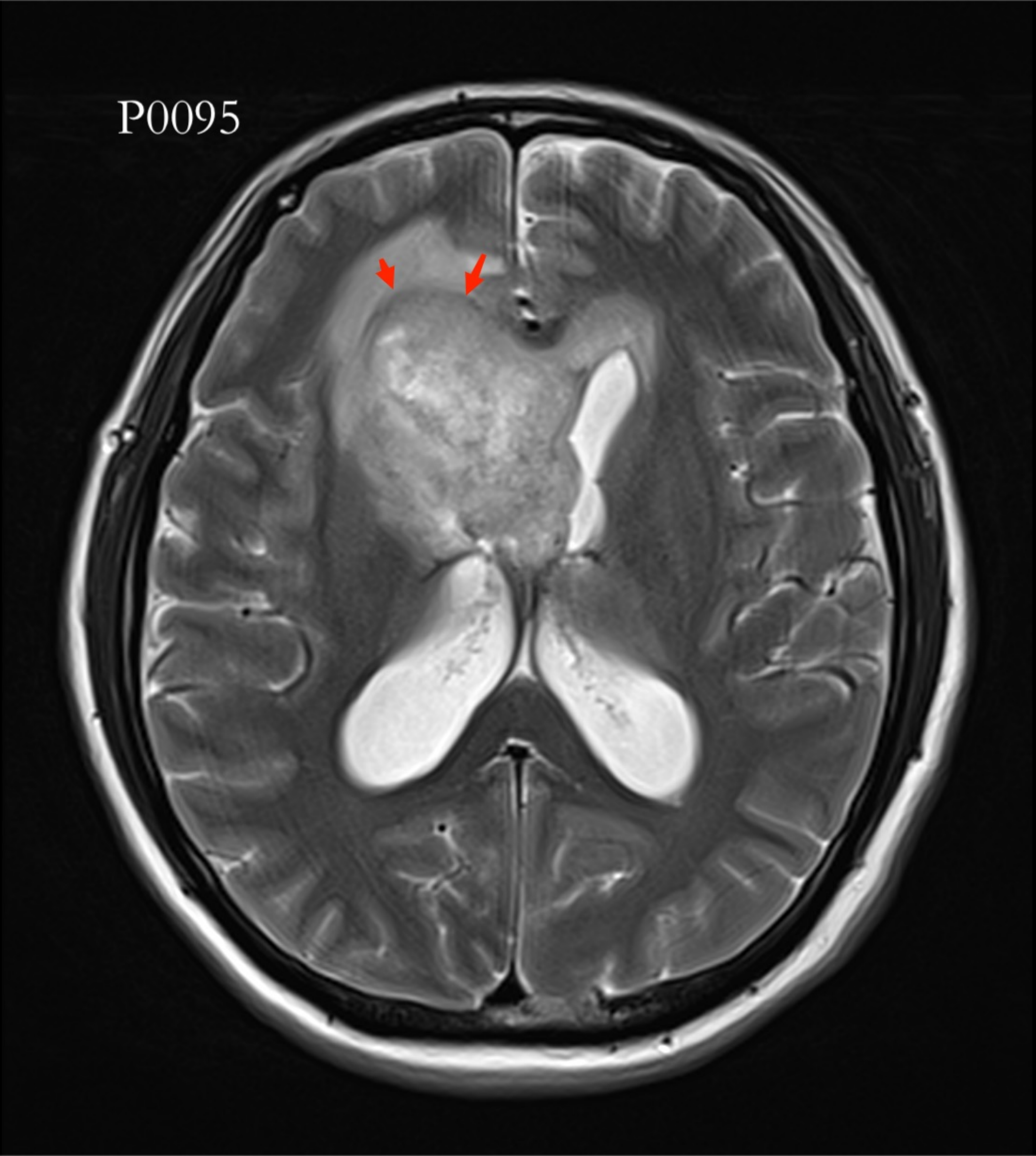
P0095

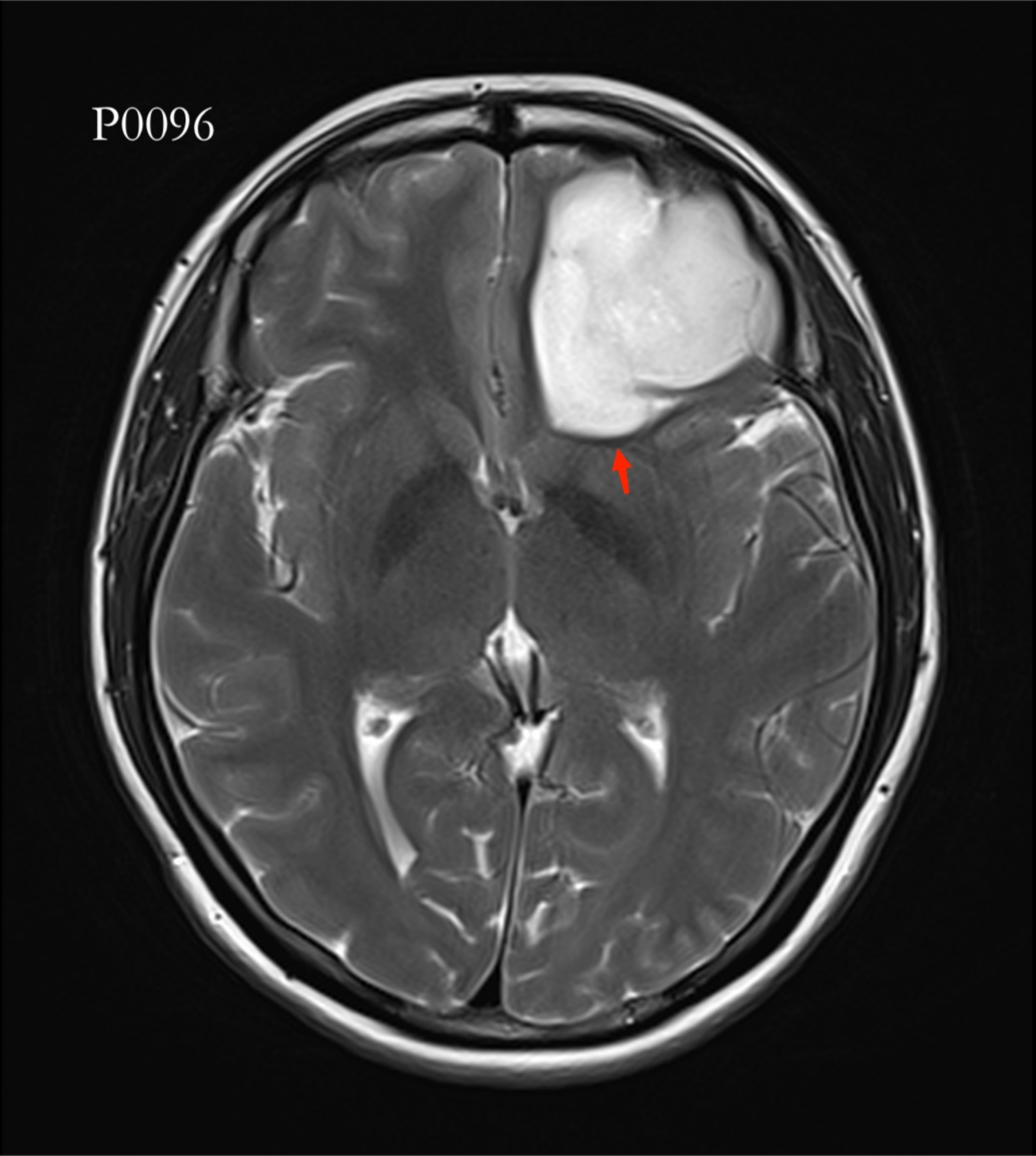
P0096

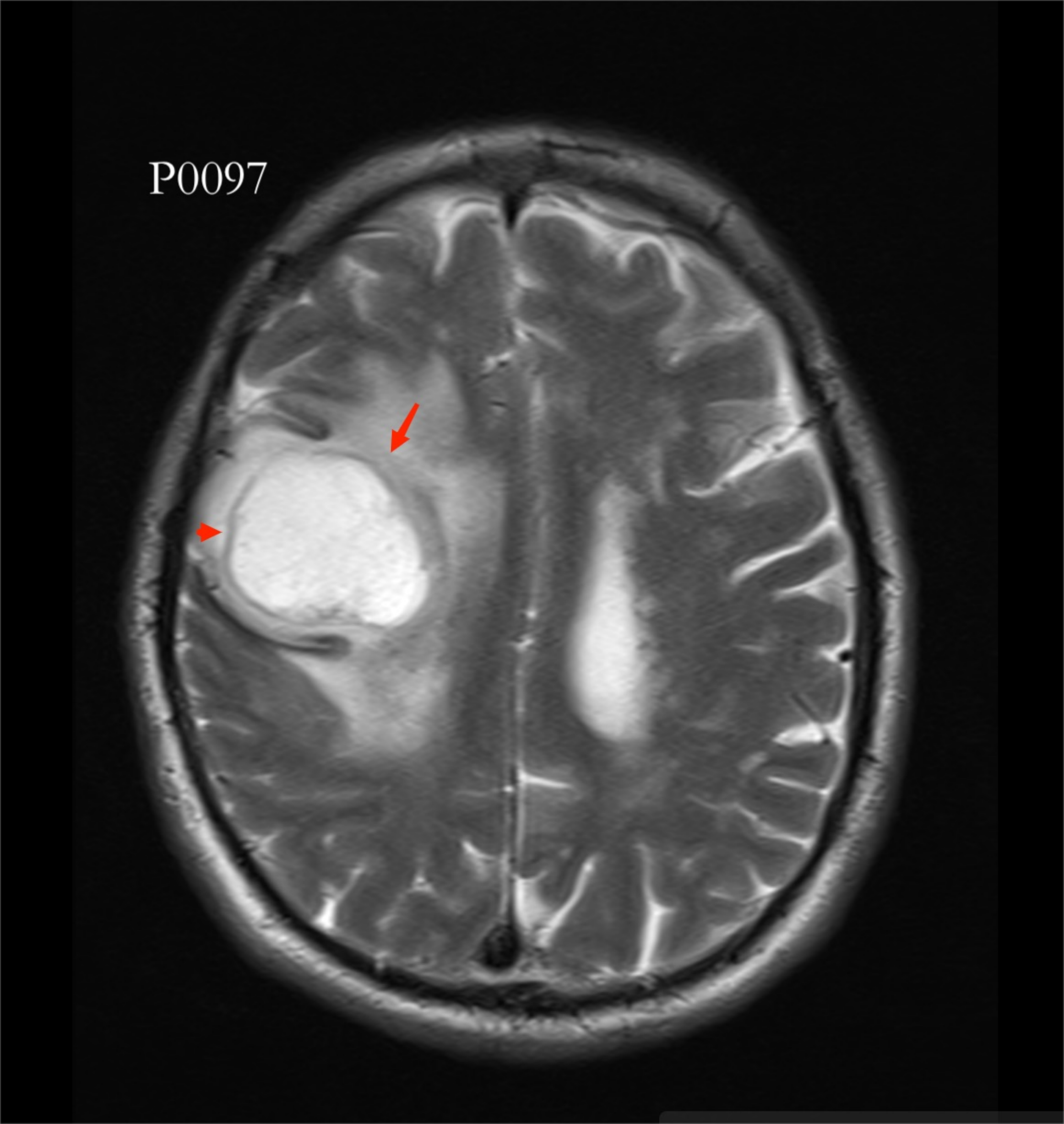
P0097

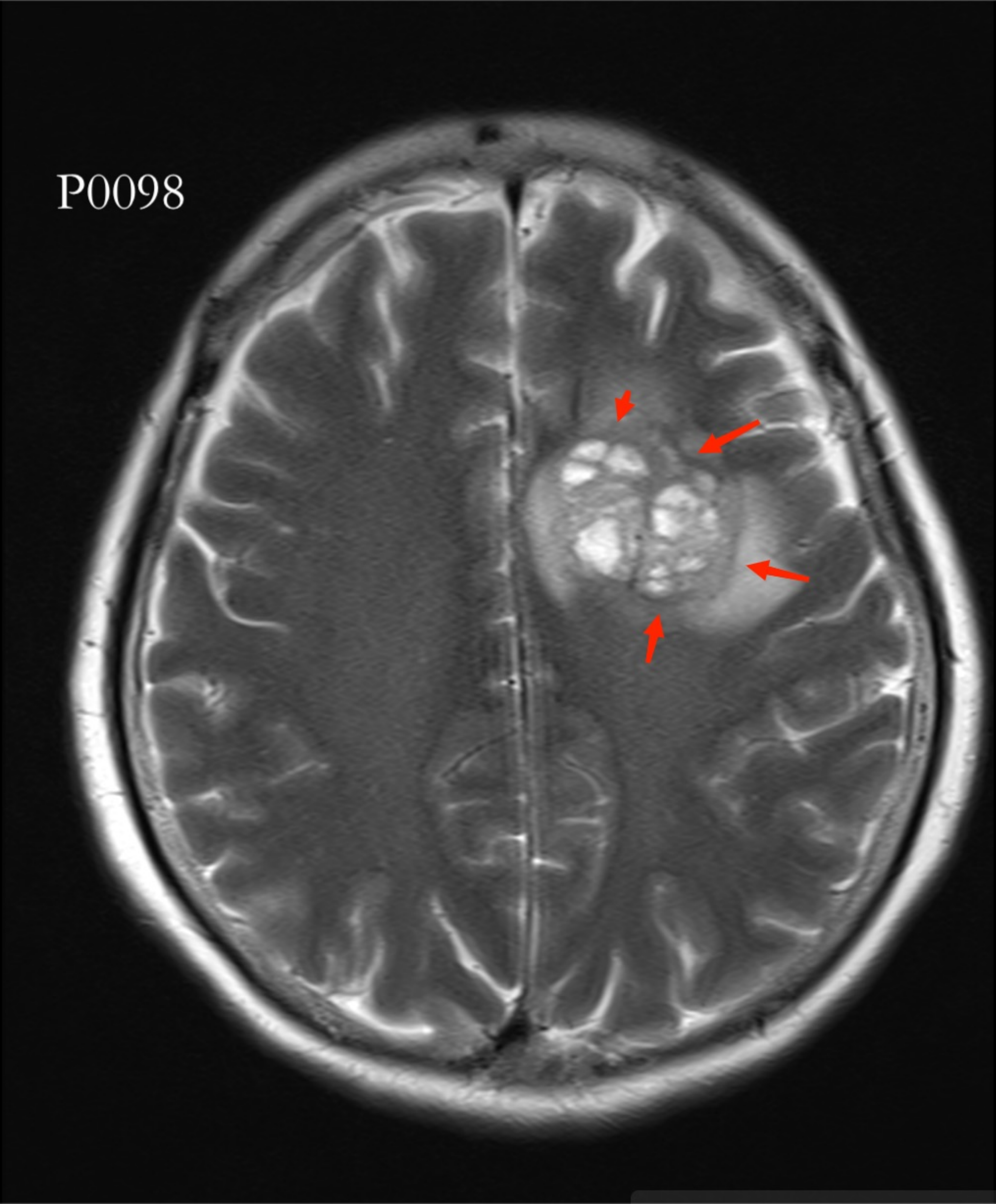
P0098

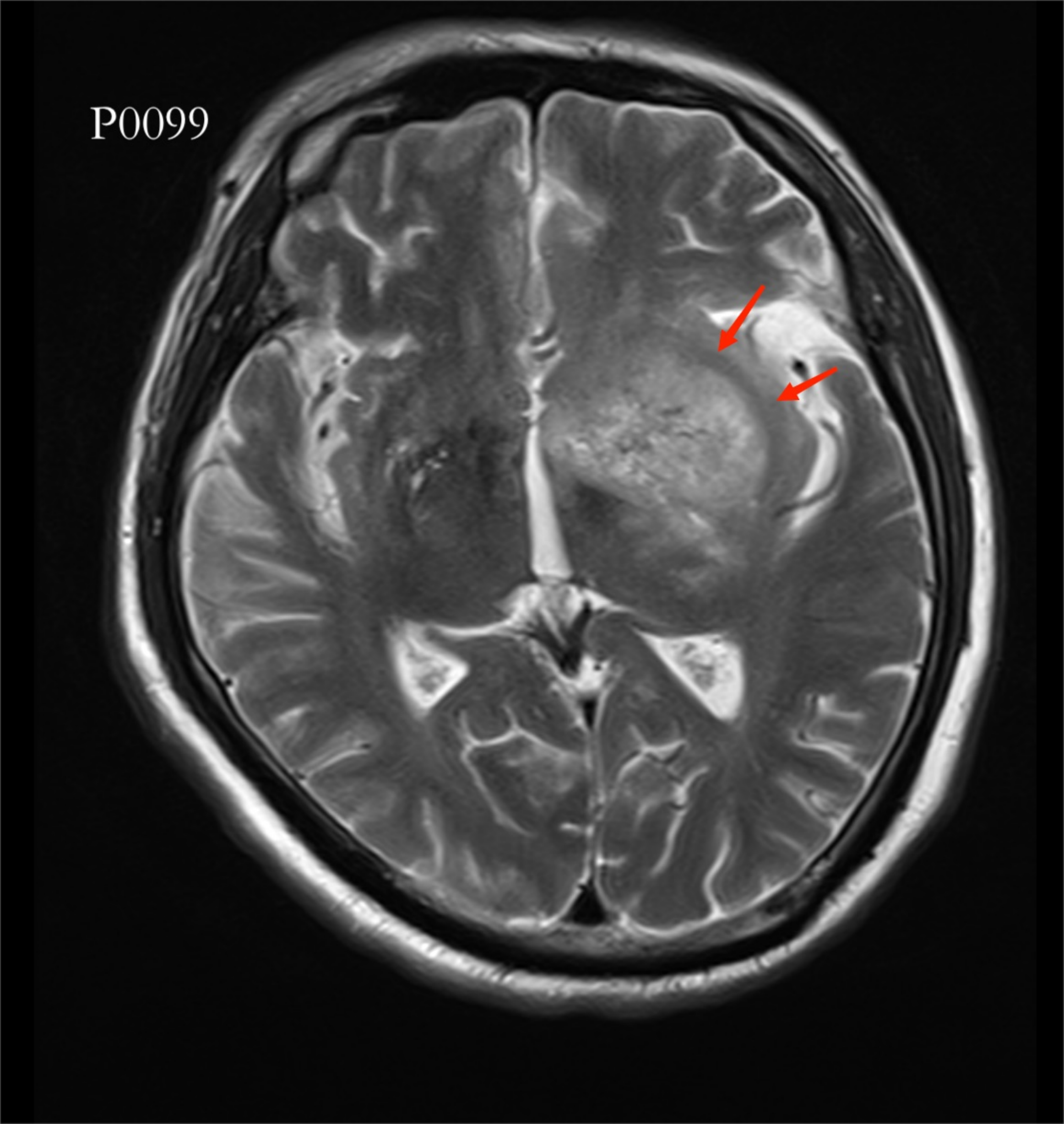
P0099

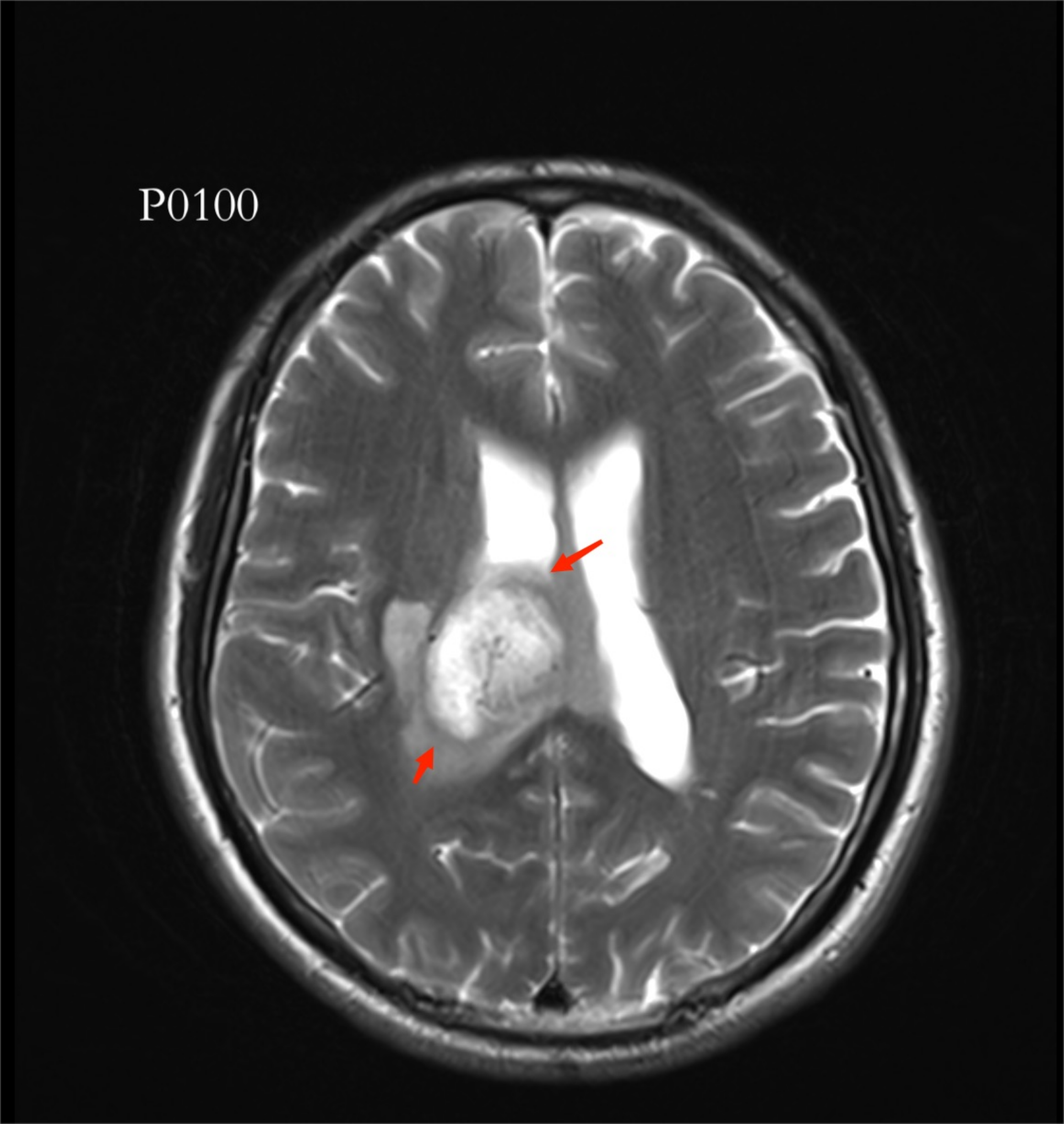
P0100

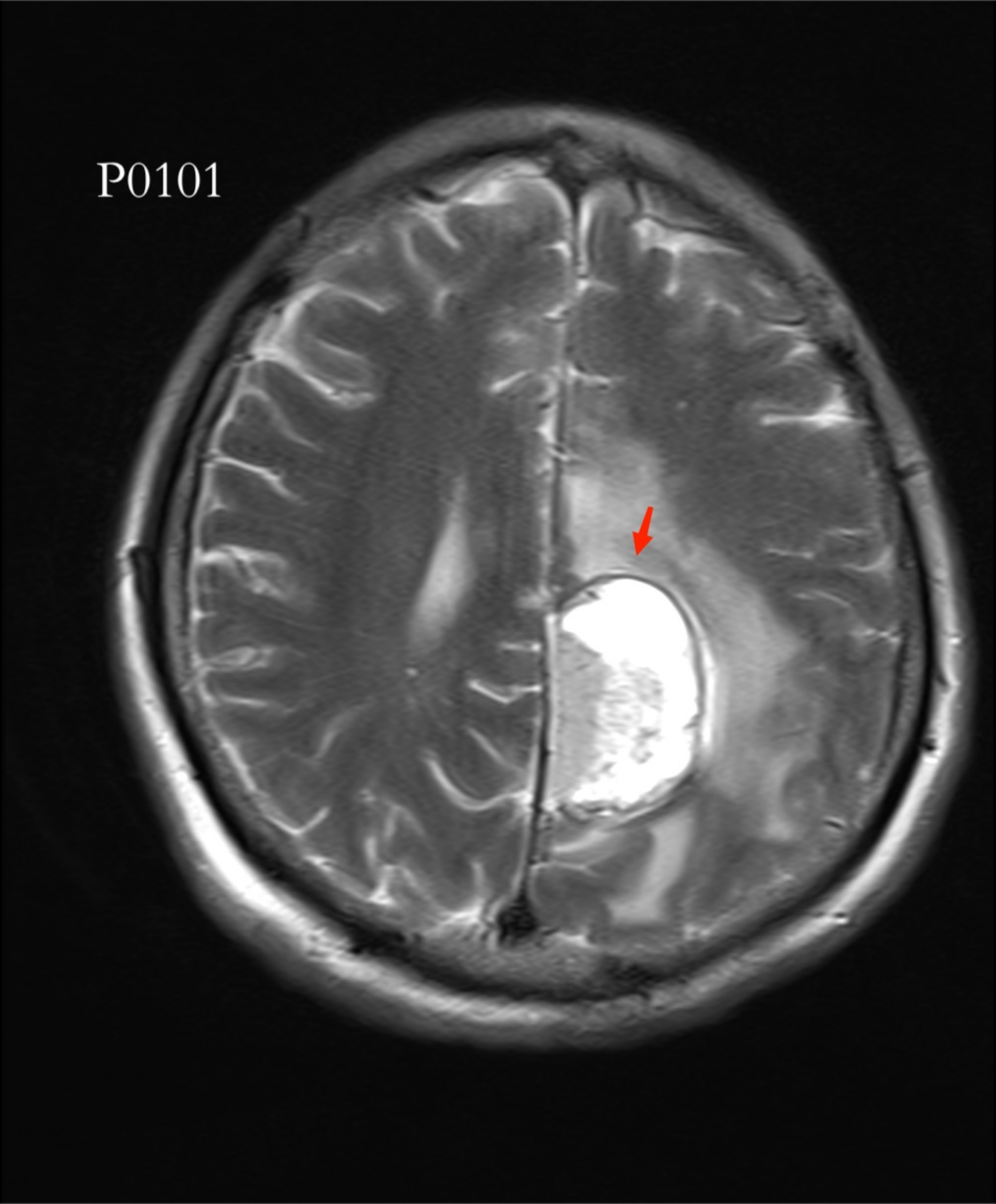
P0101

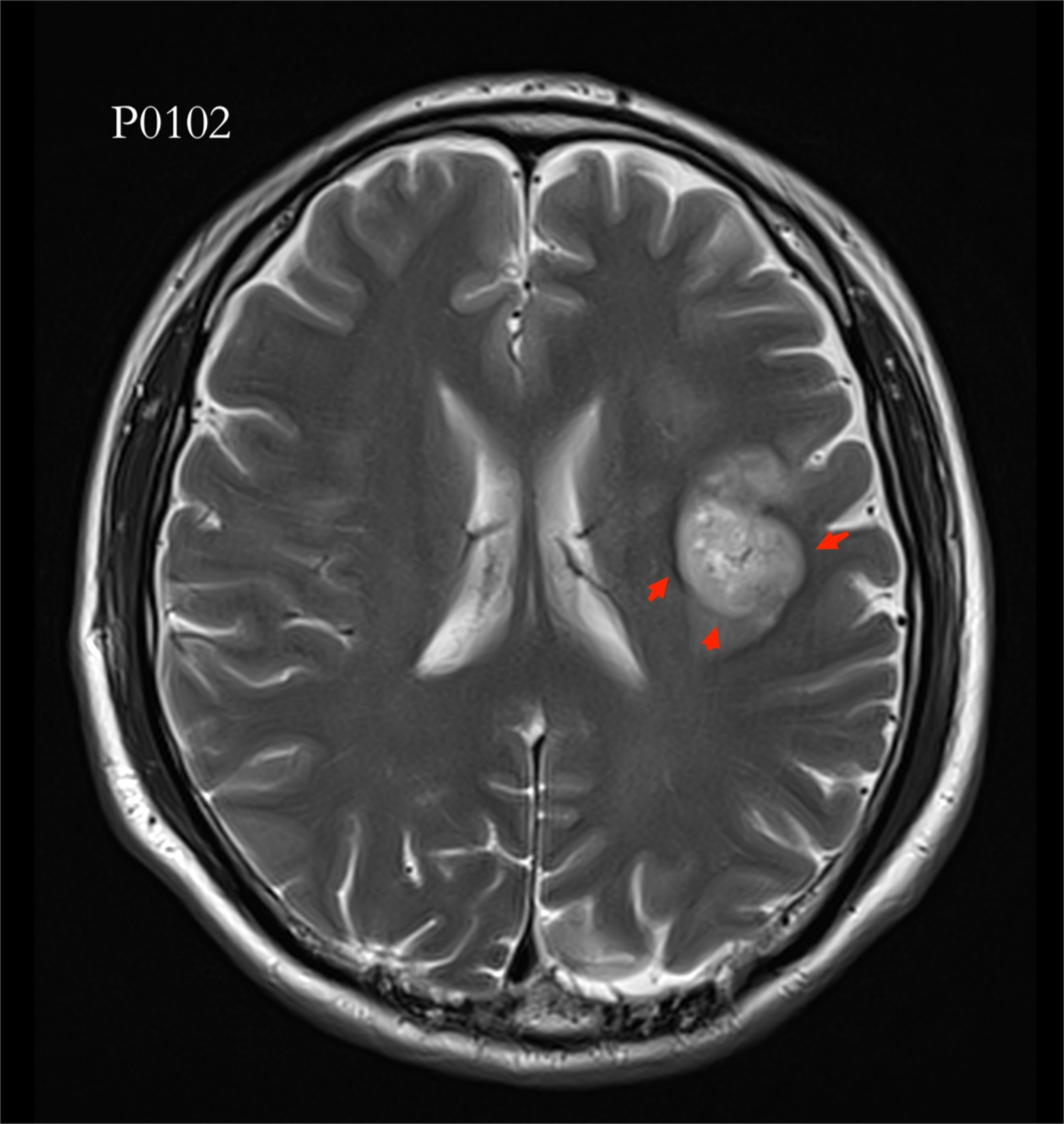
P0102

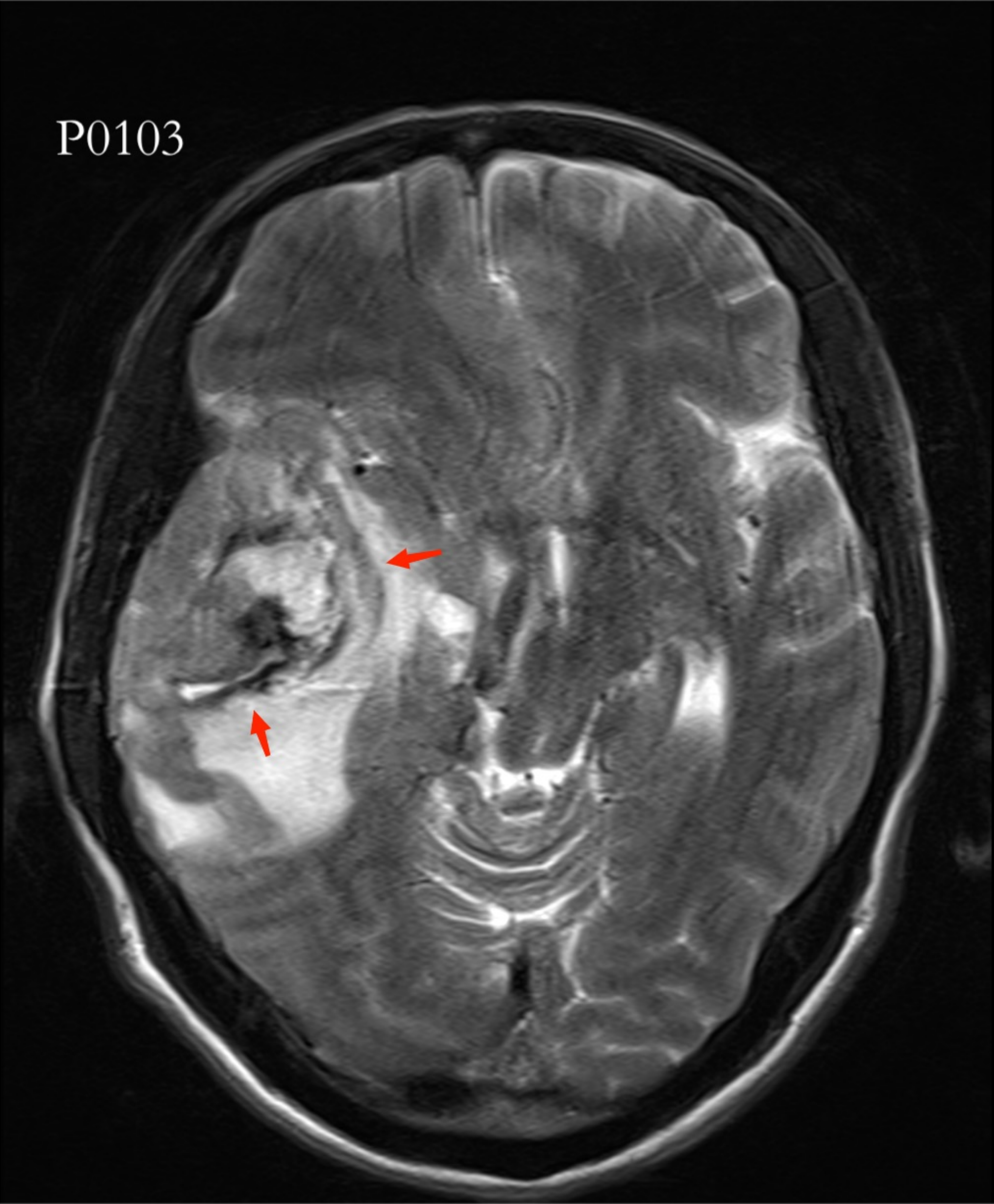
P0103

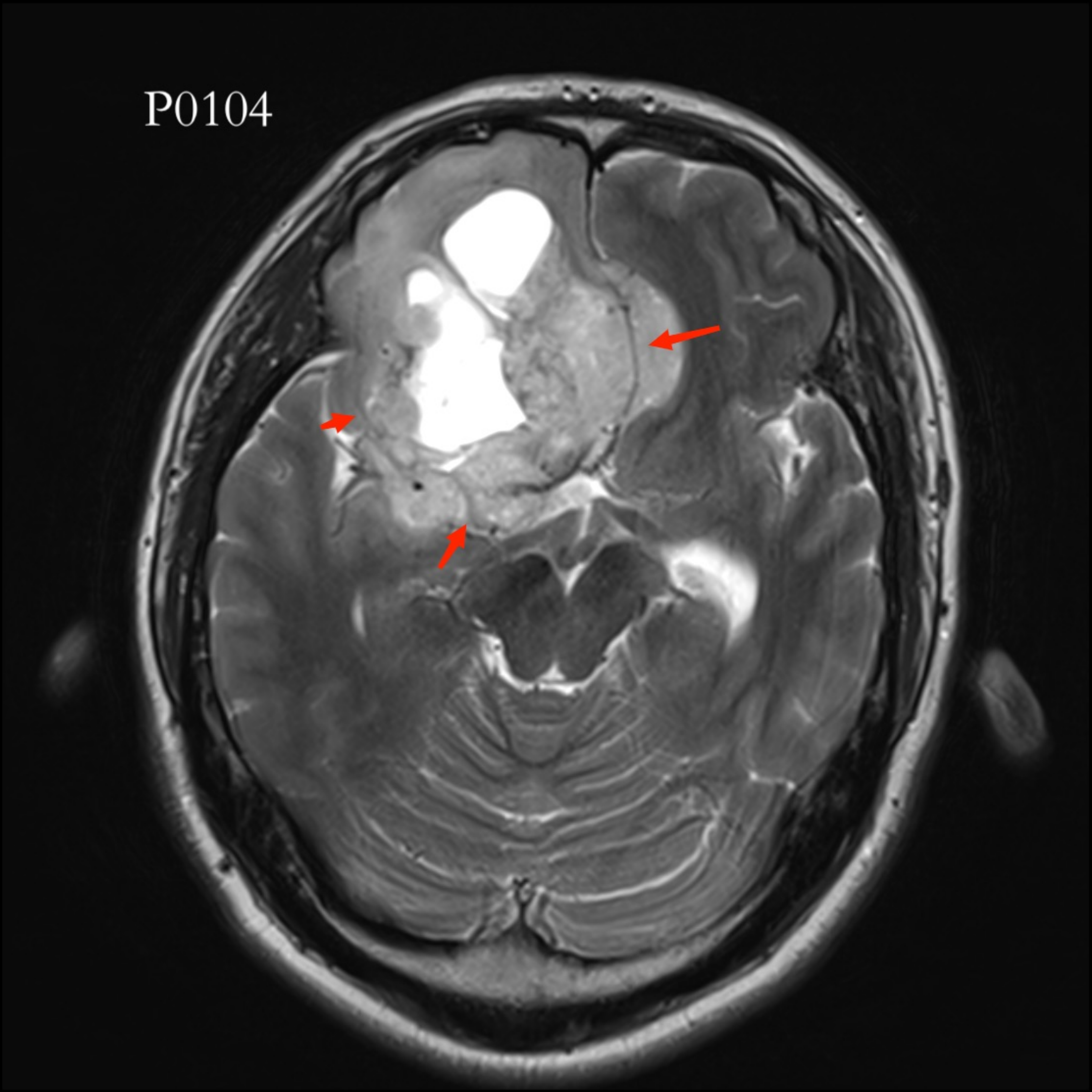
P0104

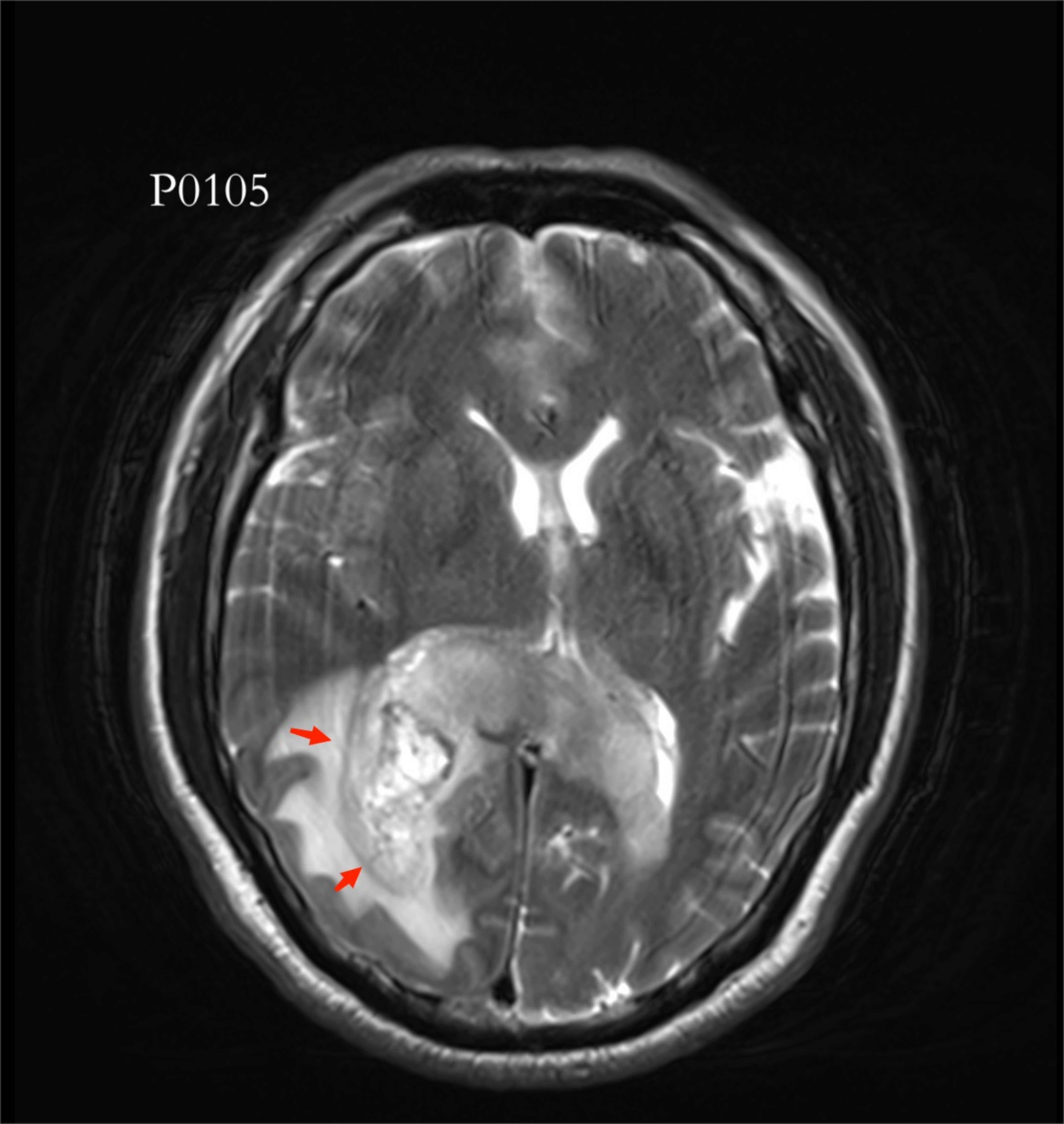
P0105

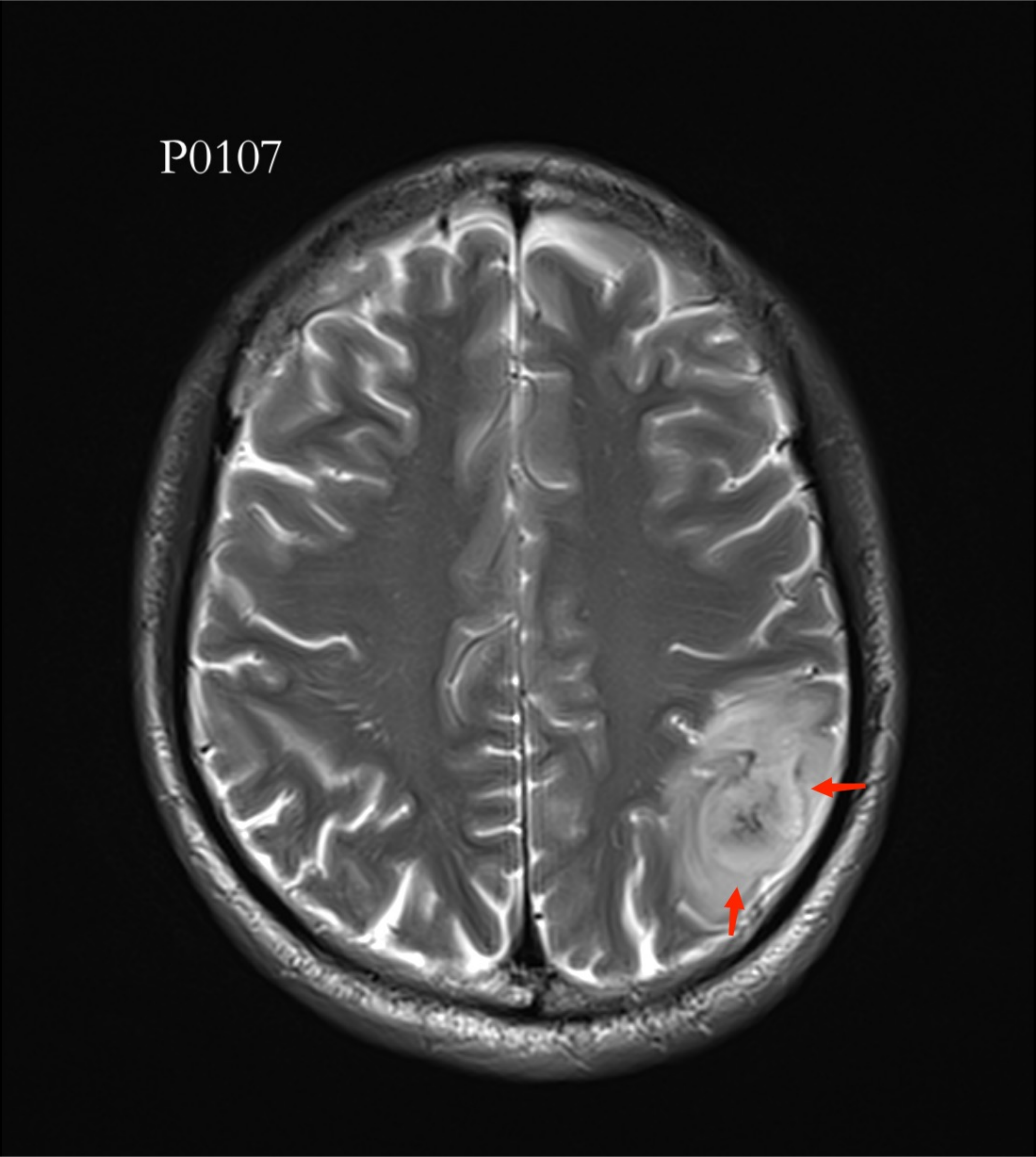
P0107

P0108

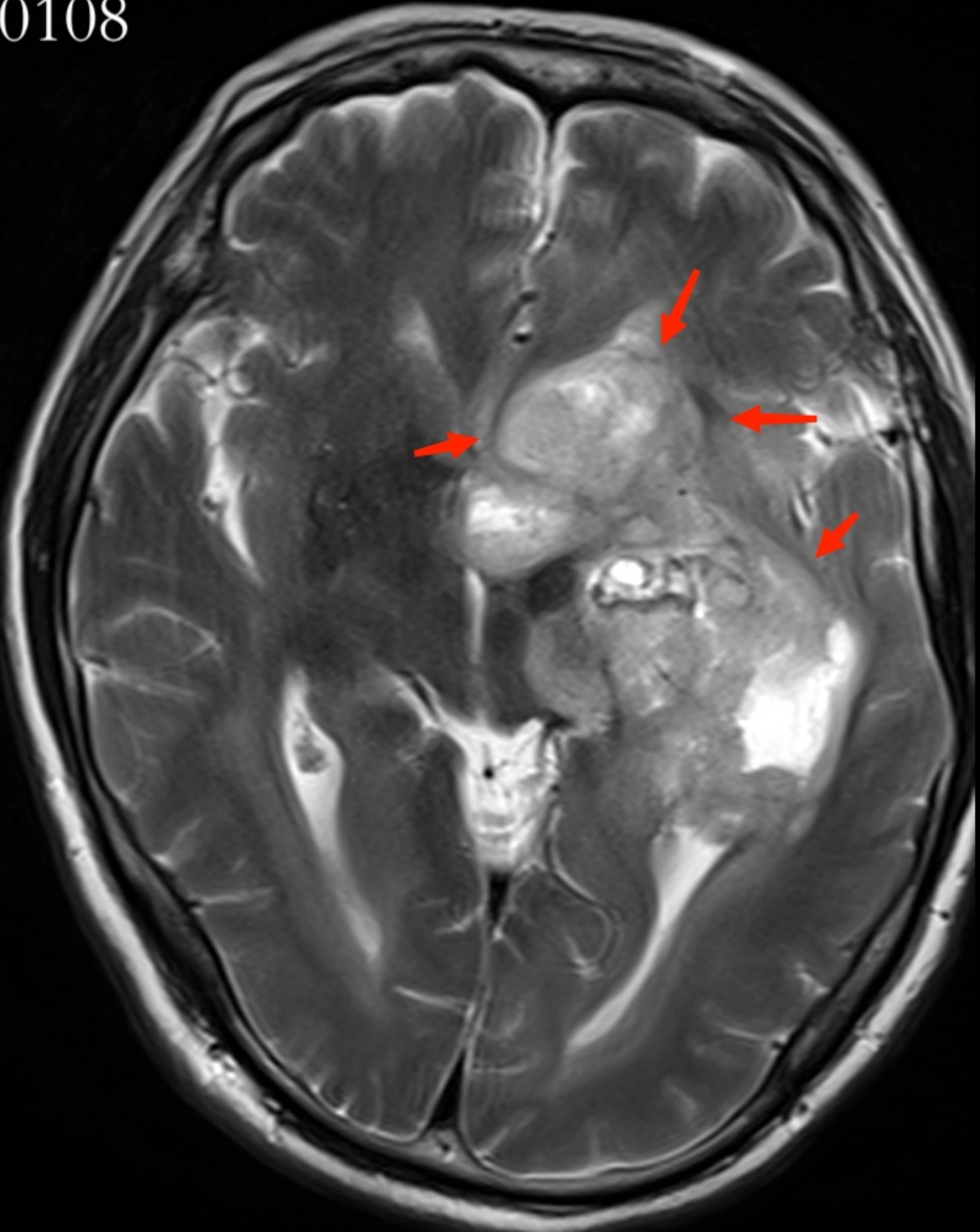

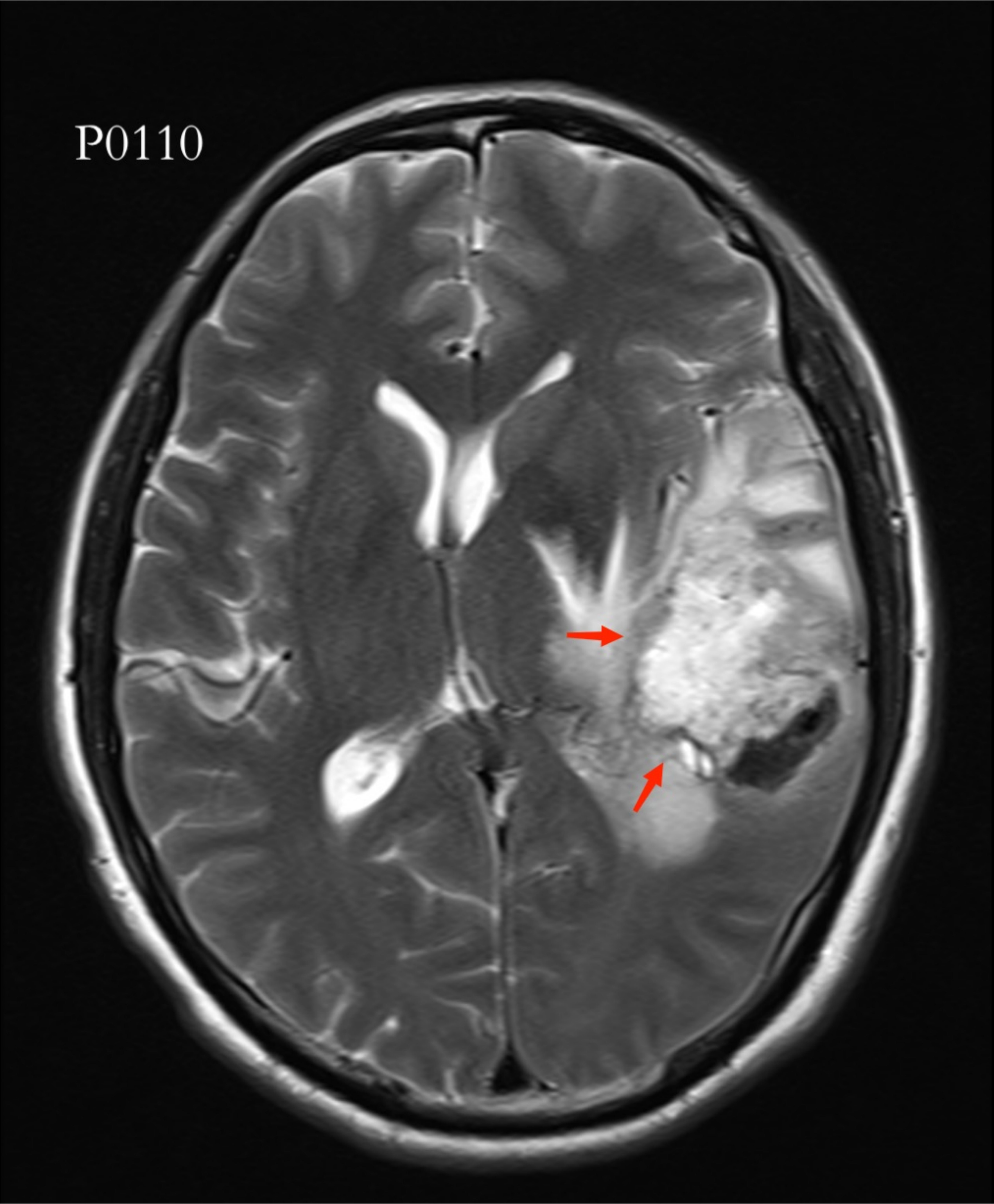
P0110

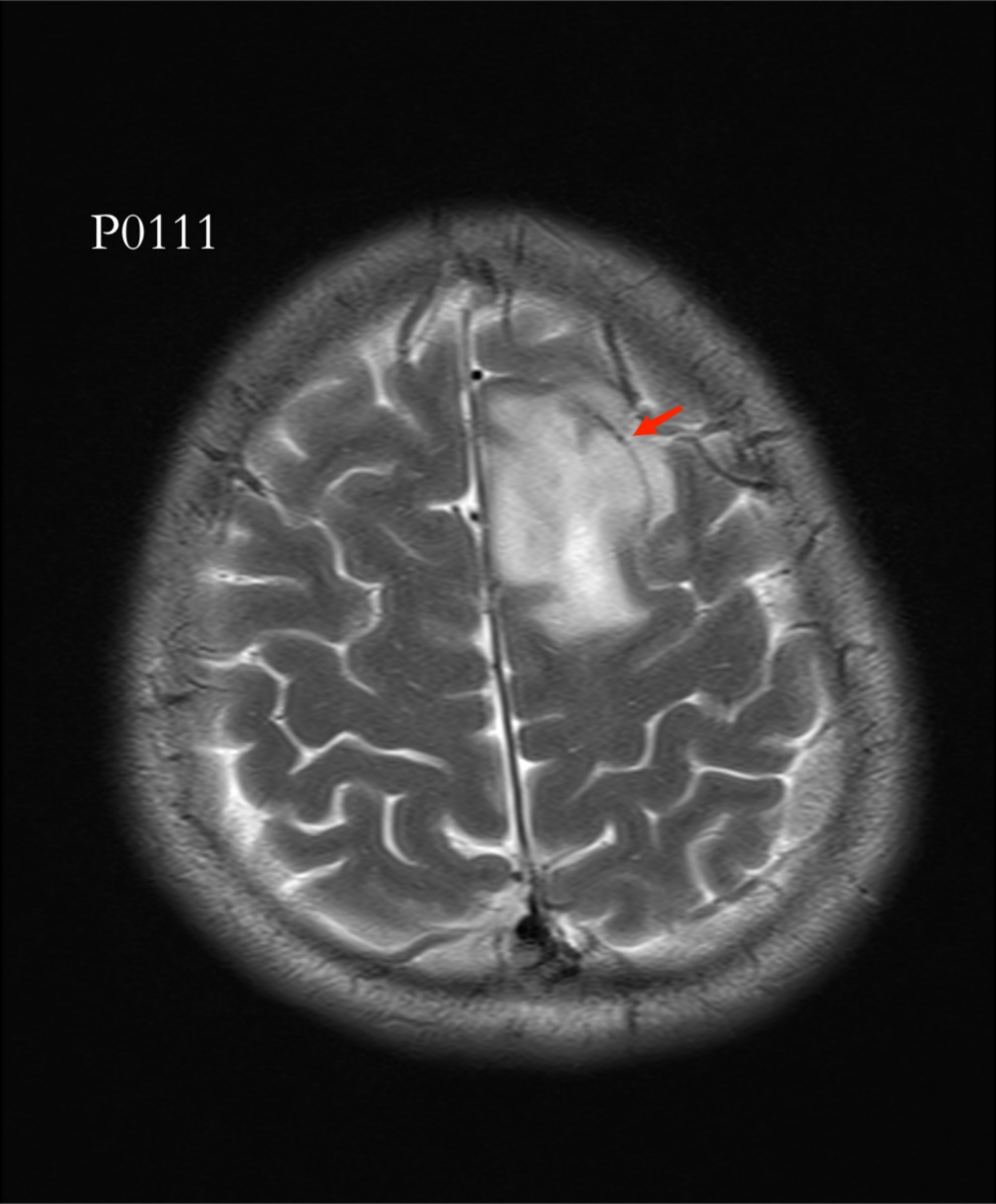
P0111

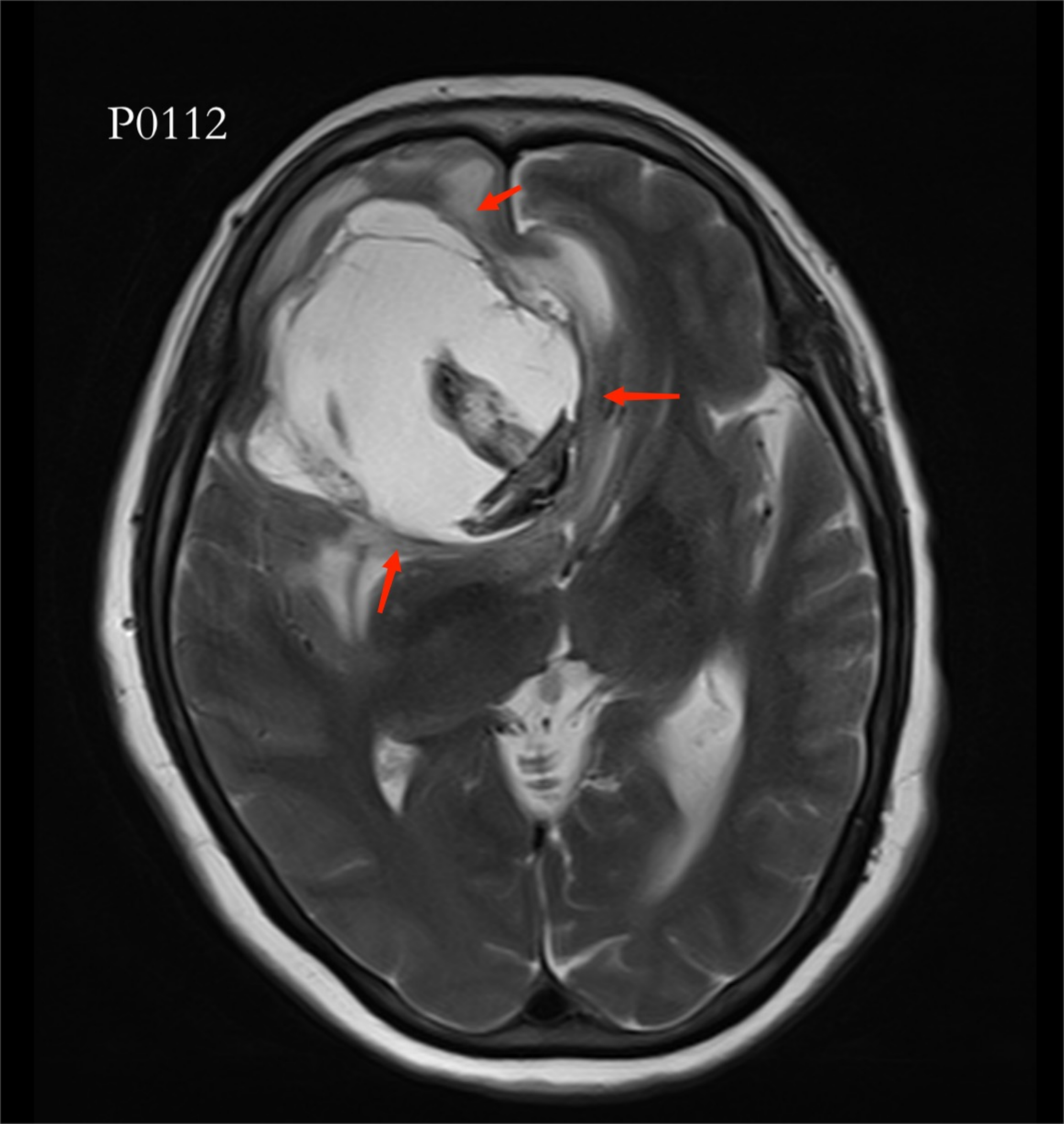
P0112

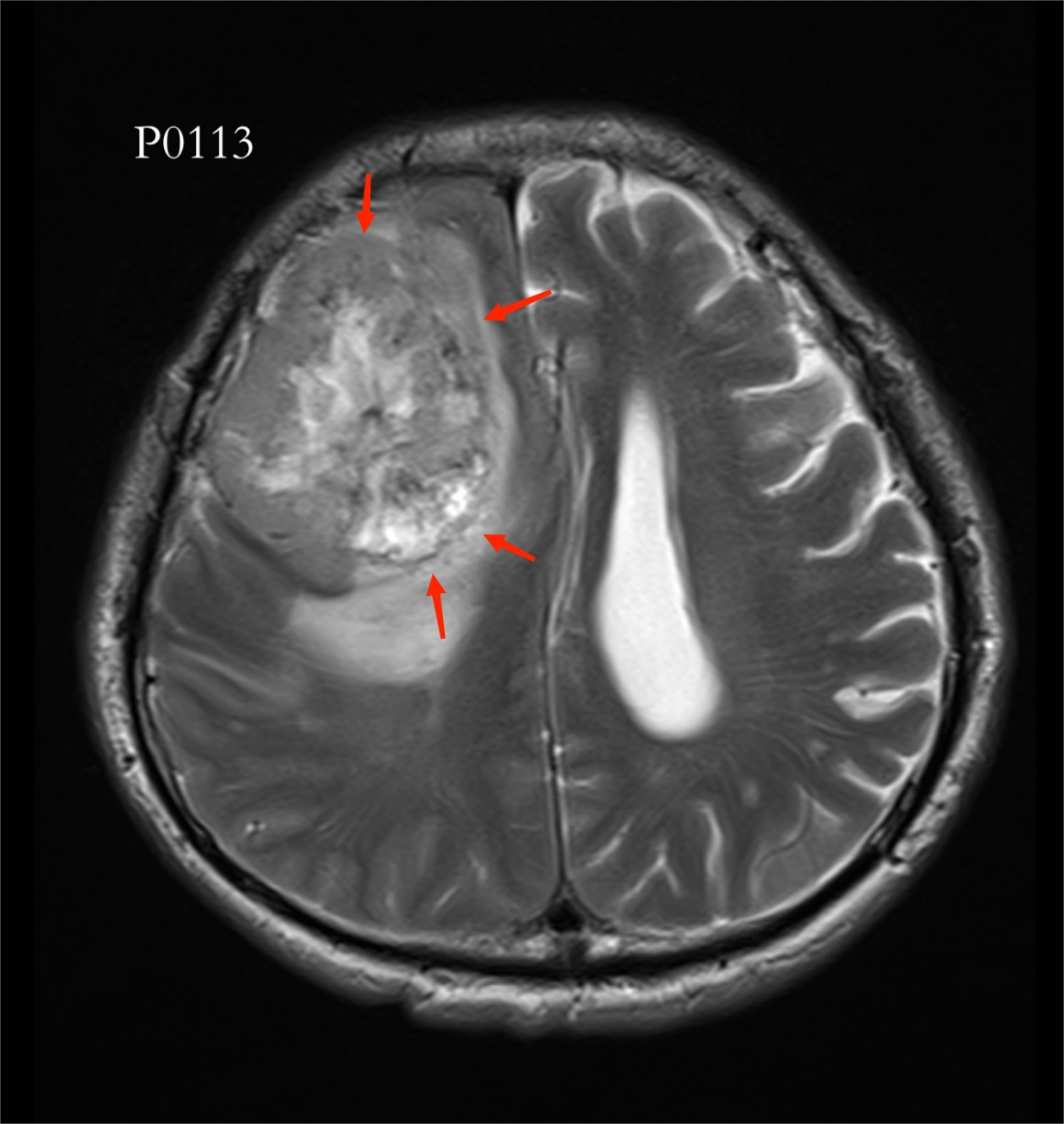
P0113

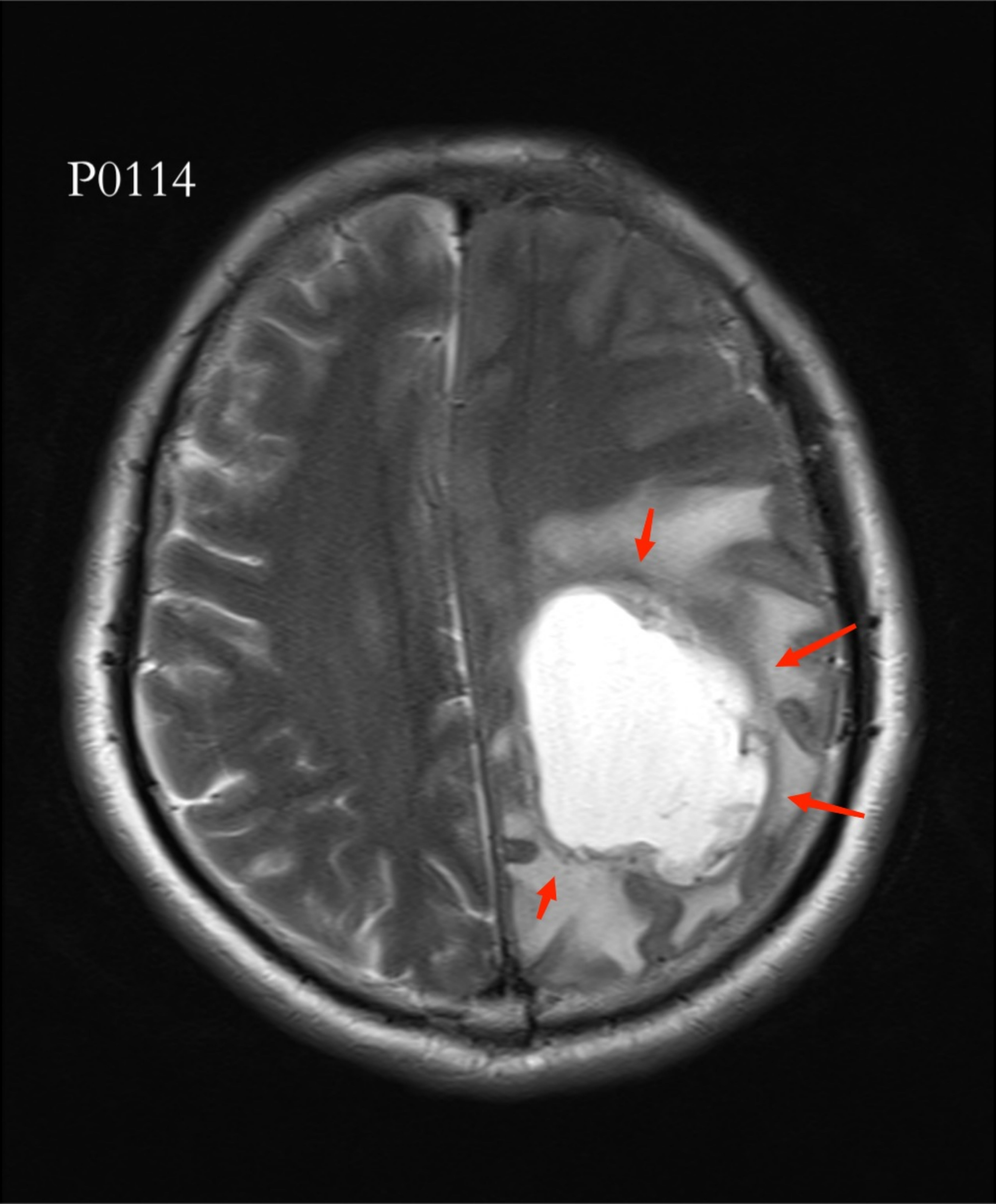
P0114

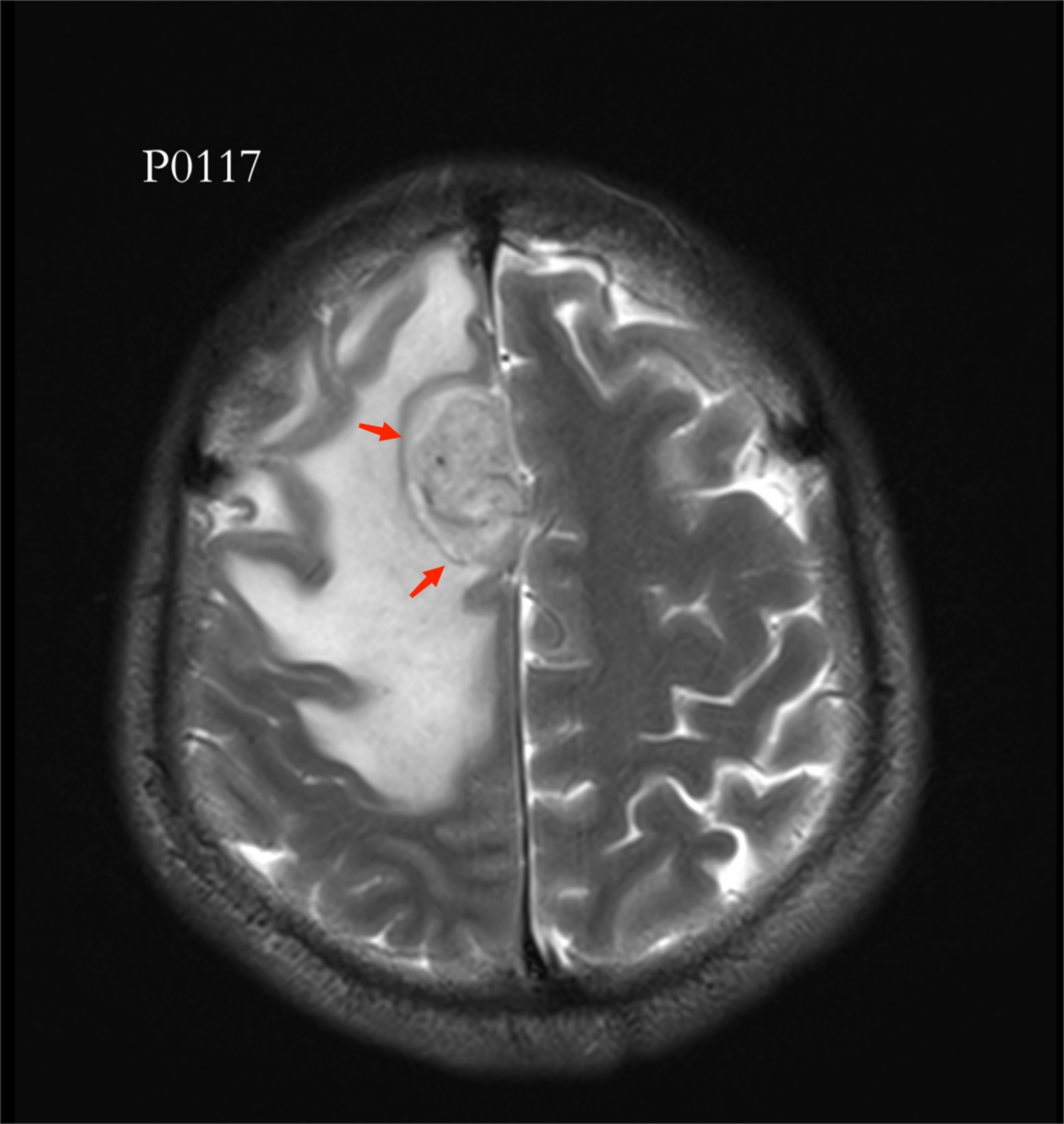
P0117

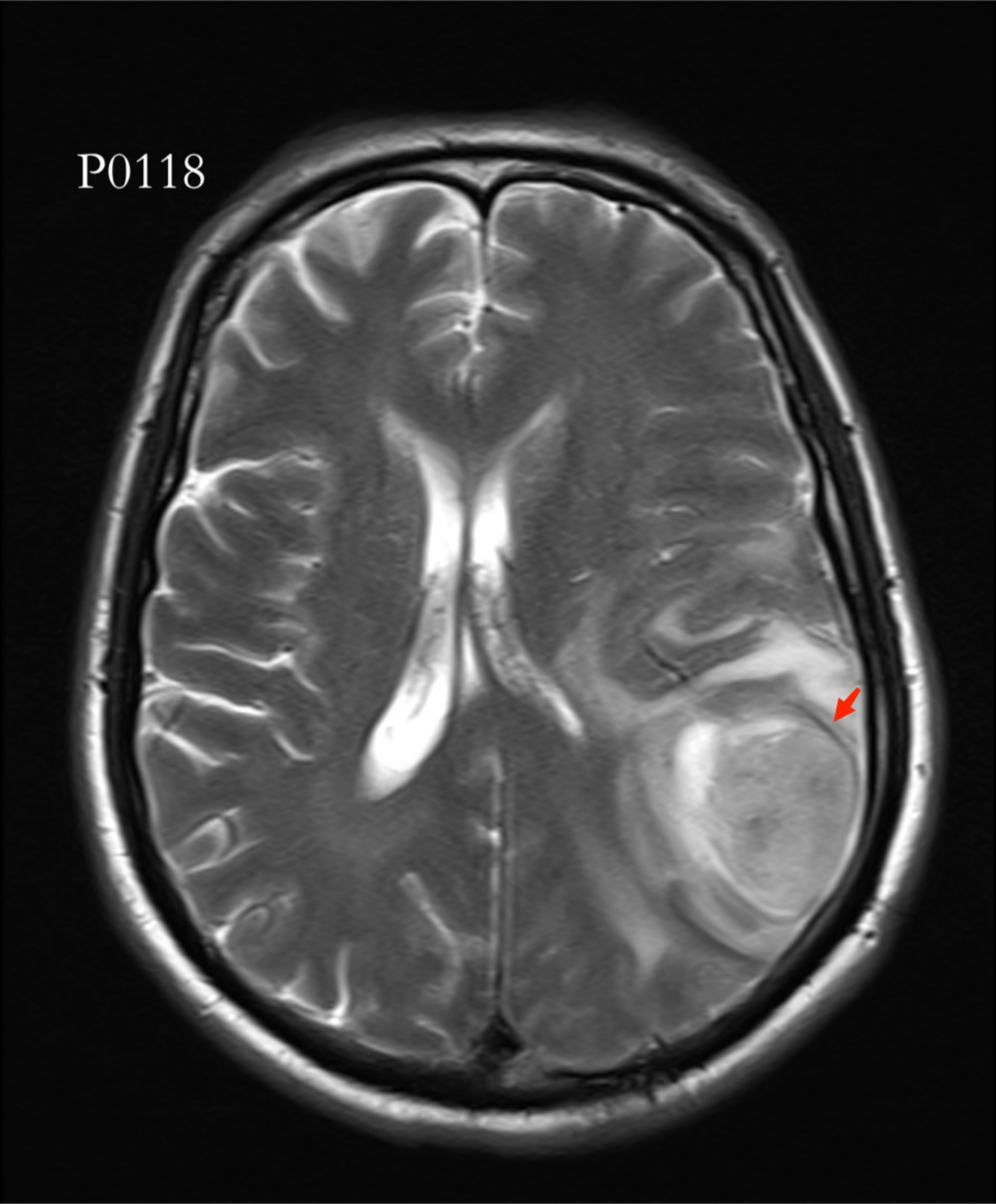
P0118

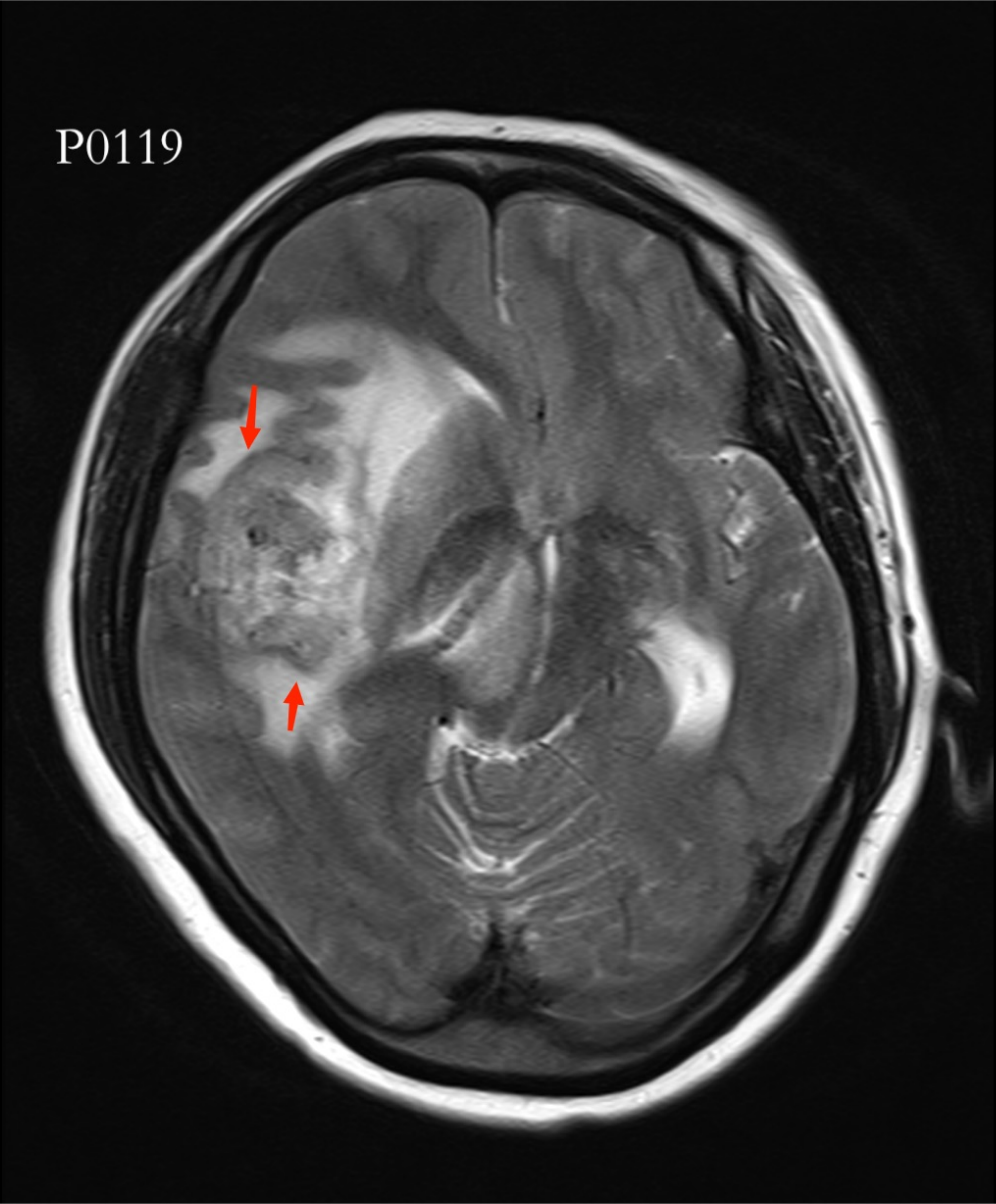
P0119

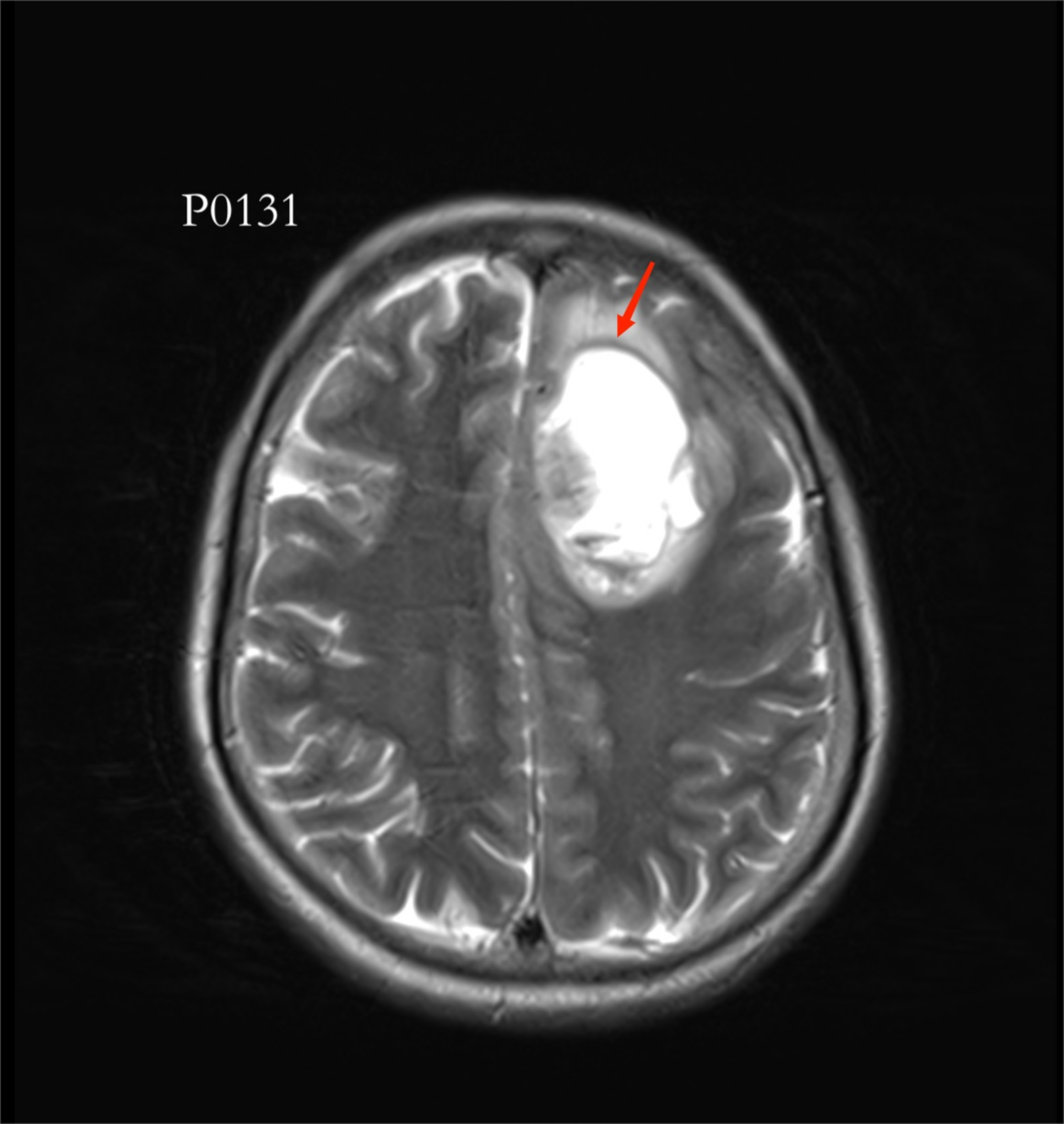
P0131

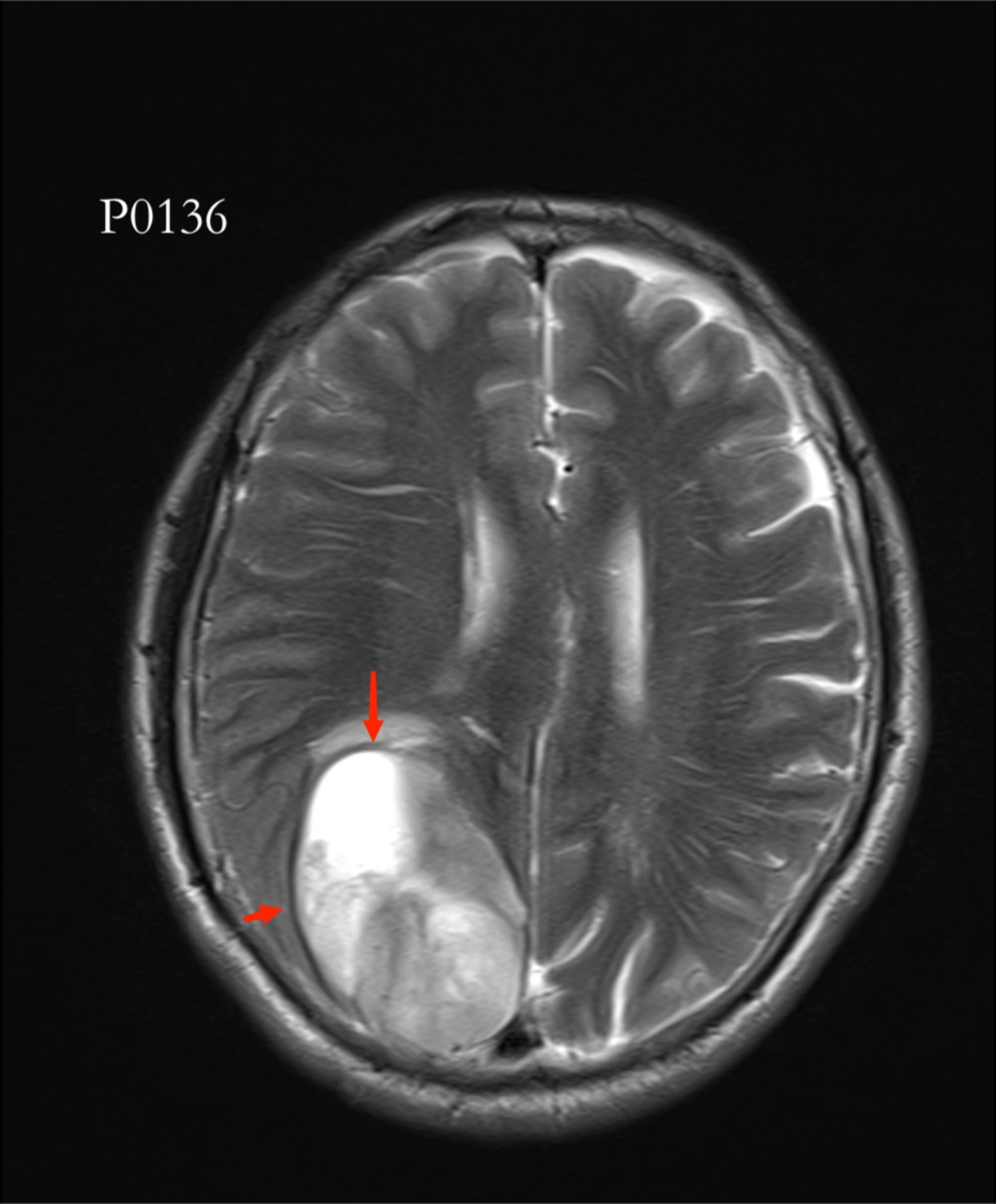
P0136

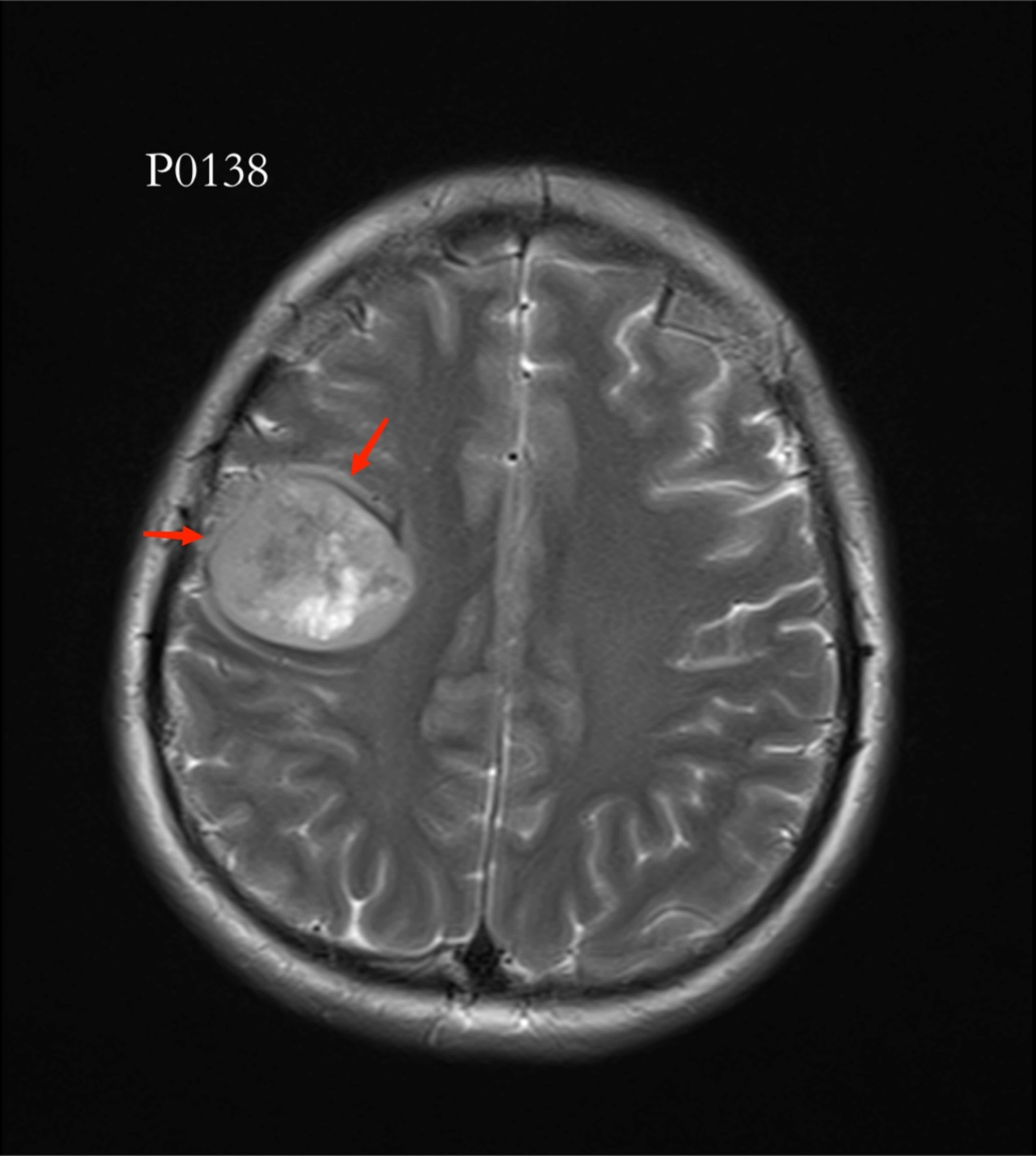
P0138

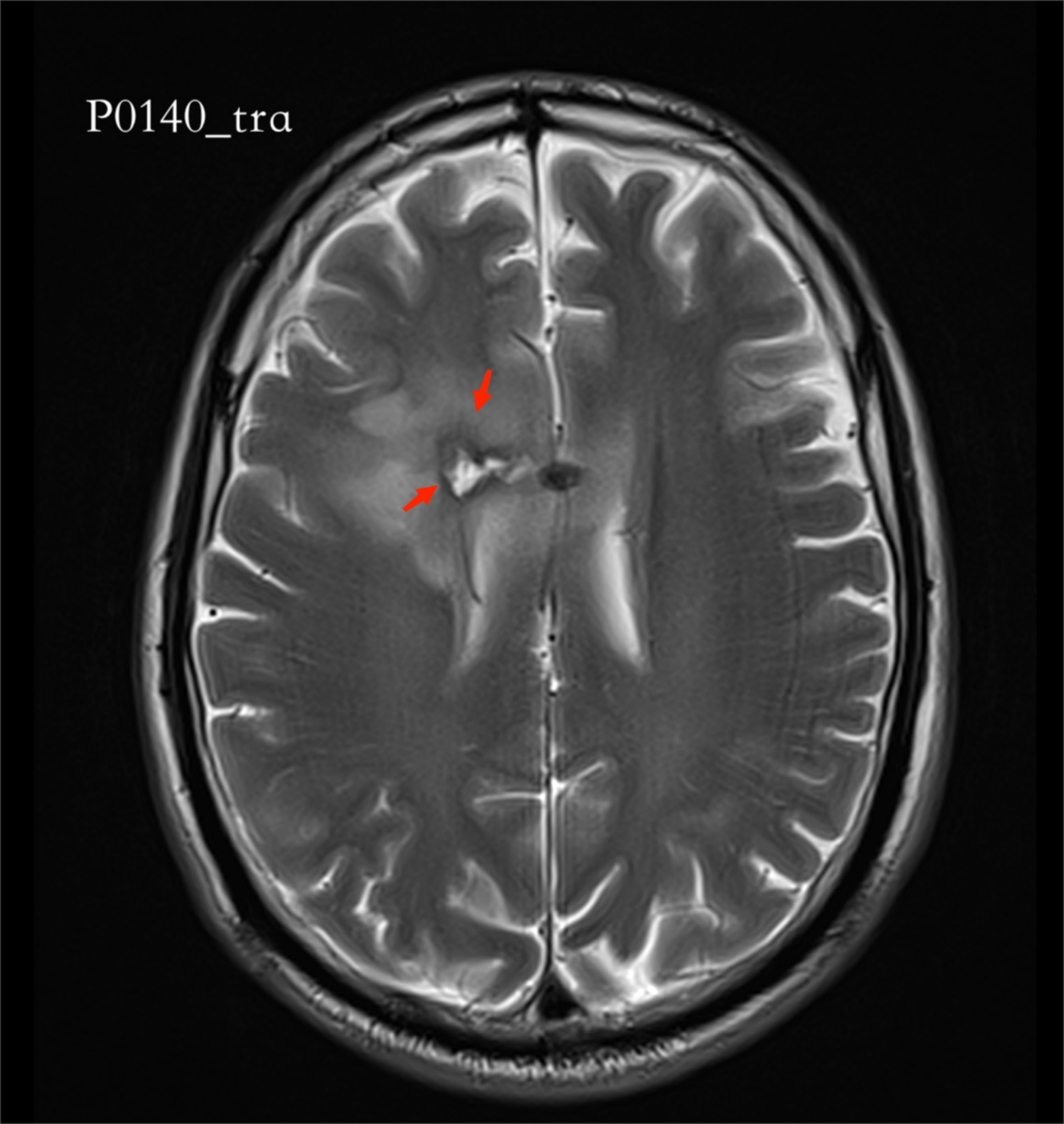
P0140_tra

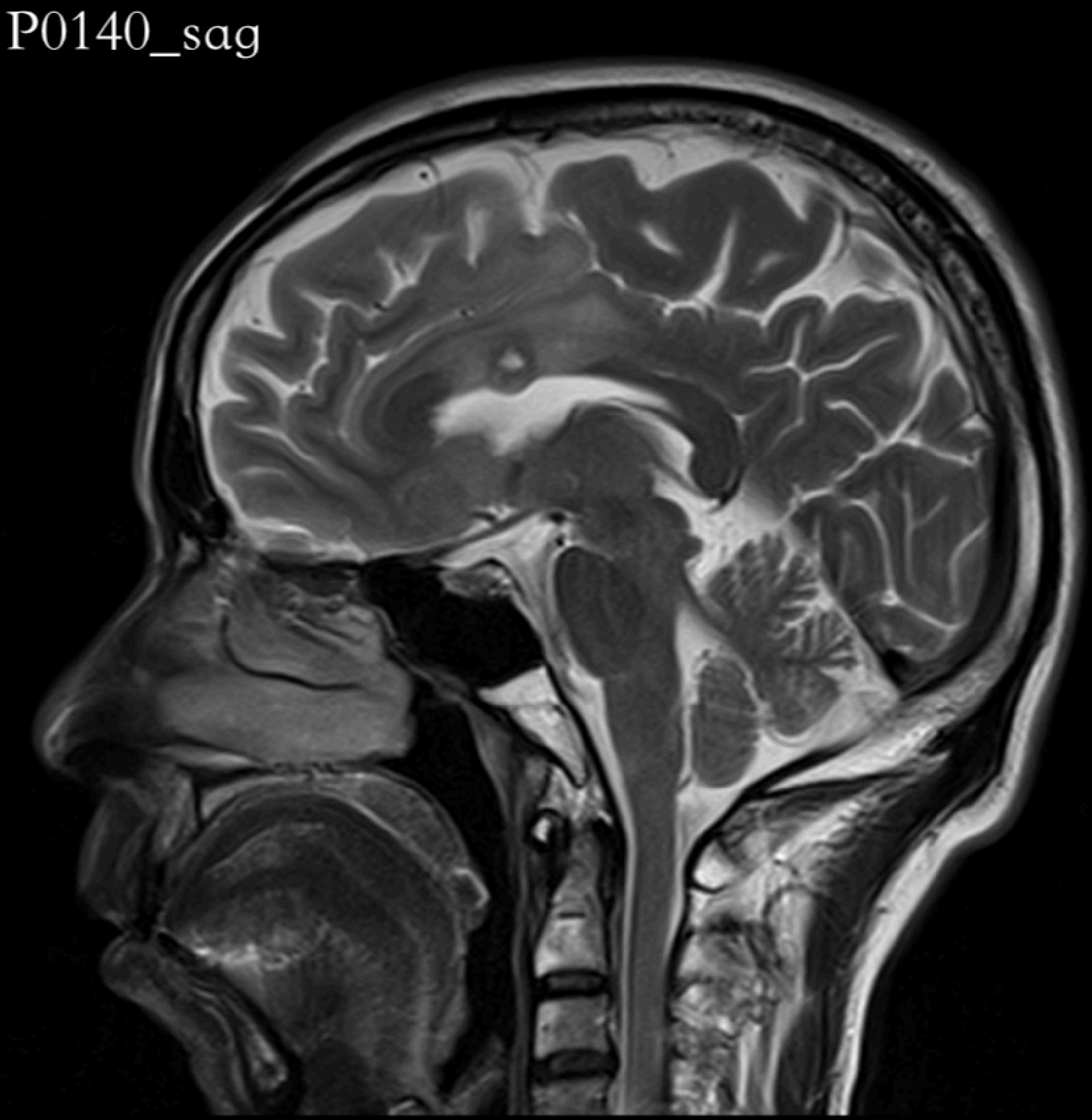
P0140_sag

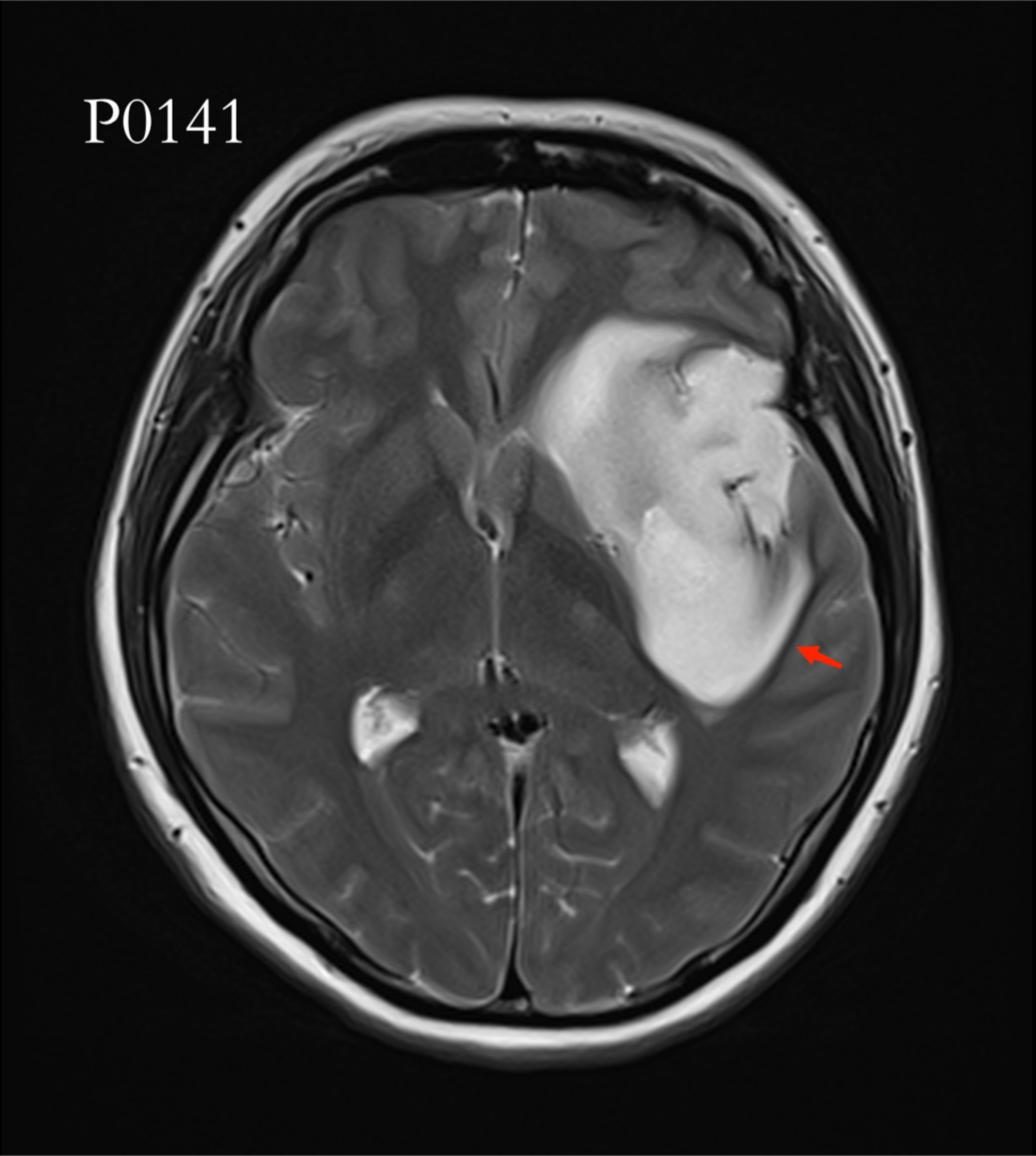
P0141

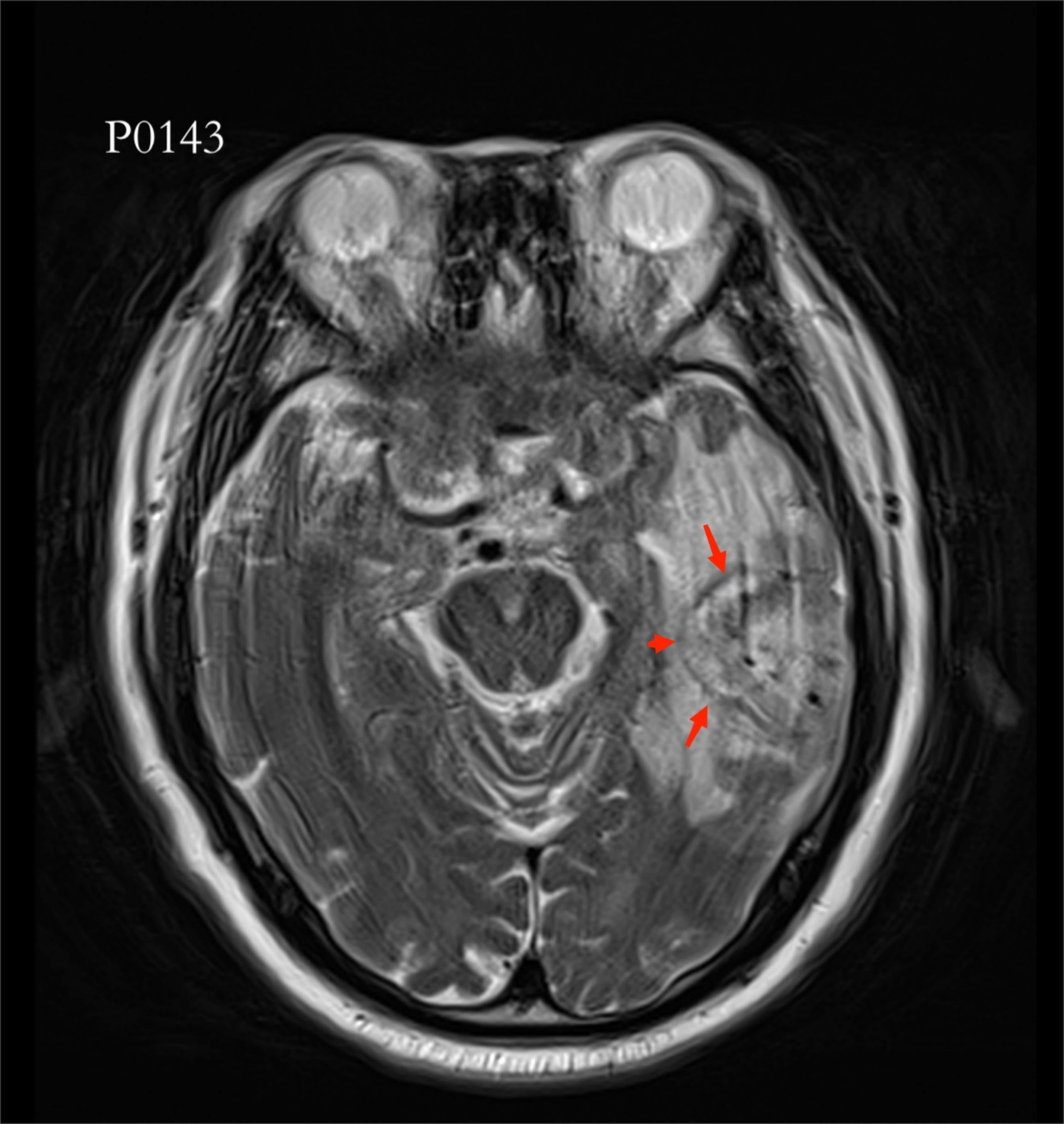
P0143

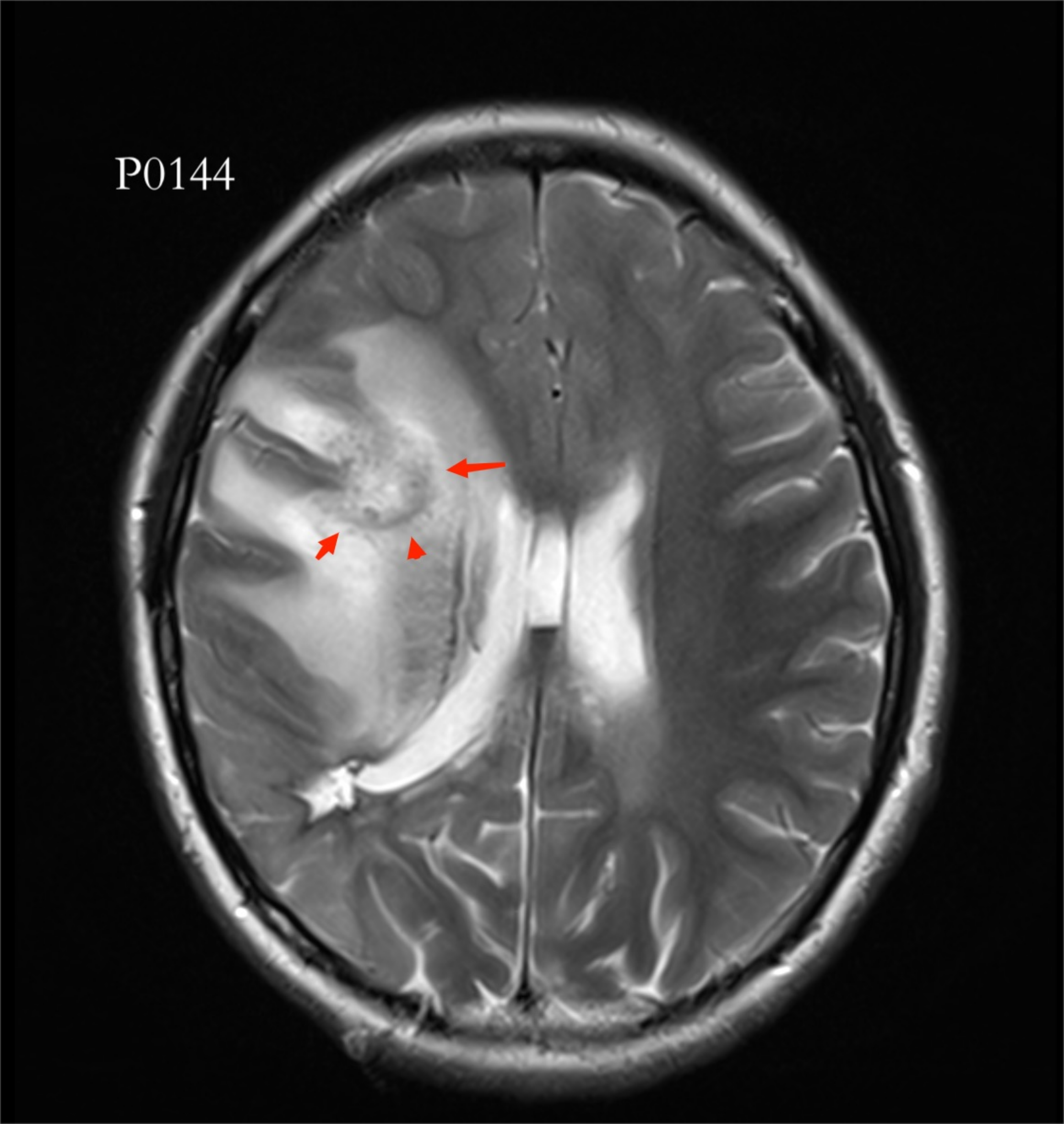

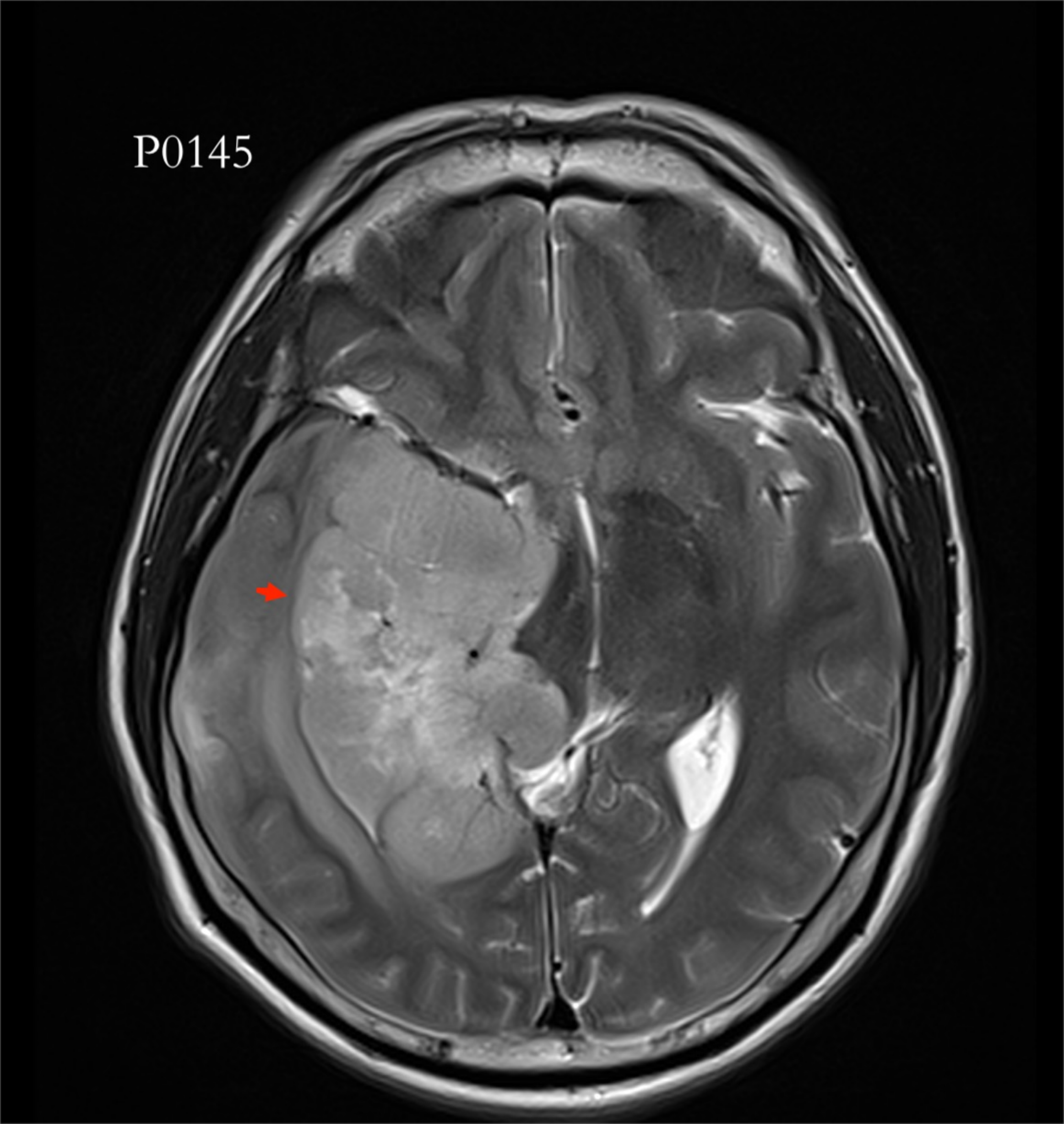
P0145

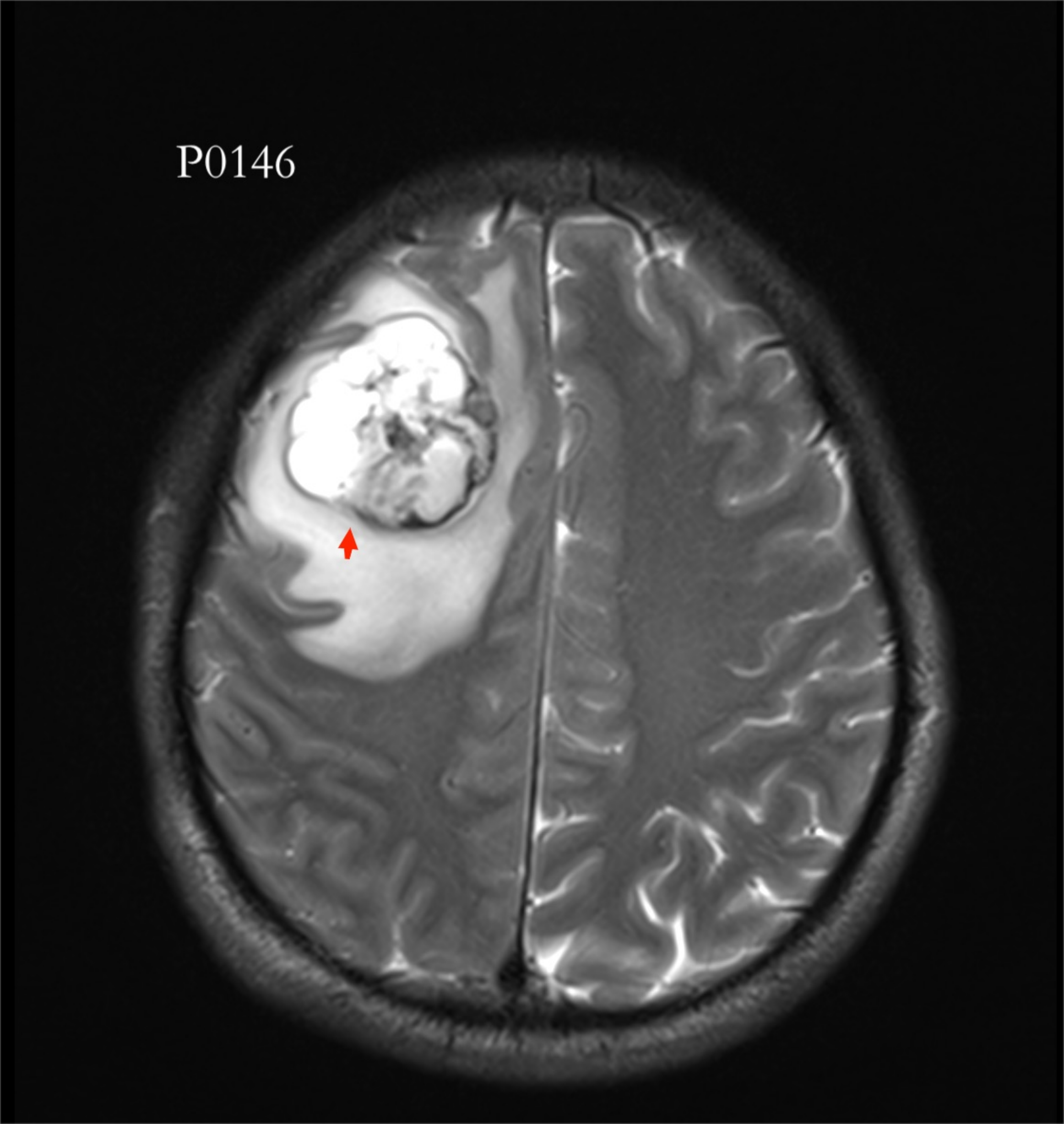
P0146

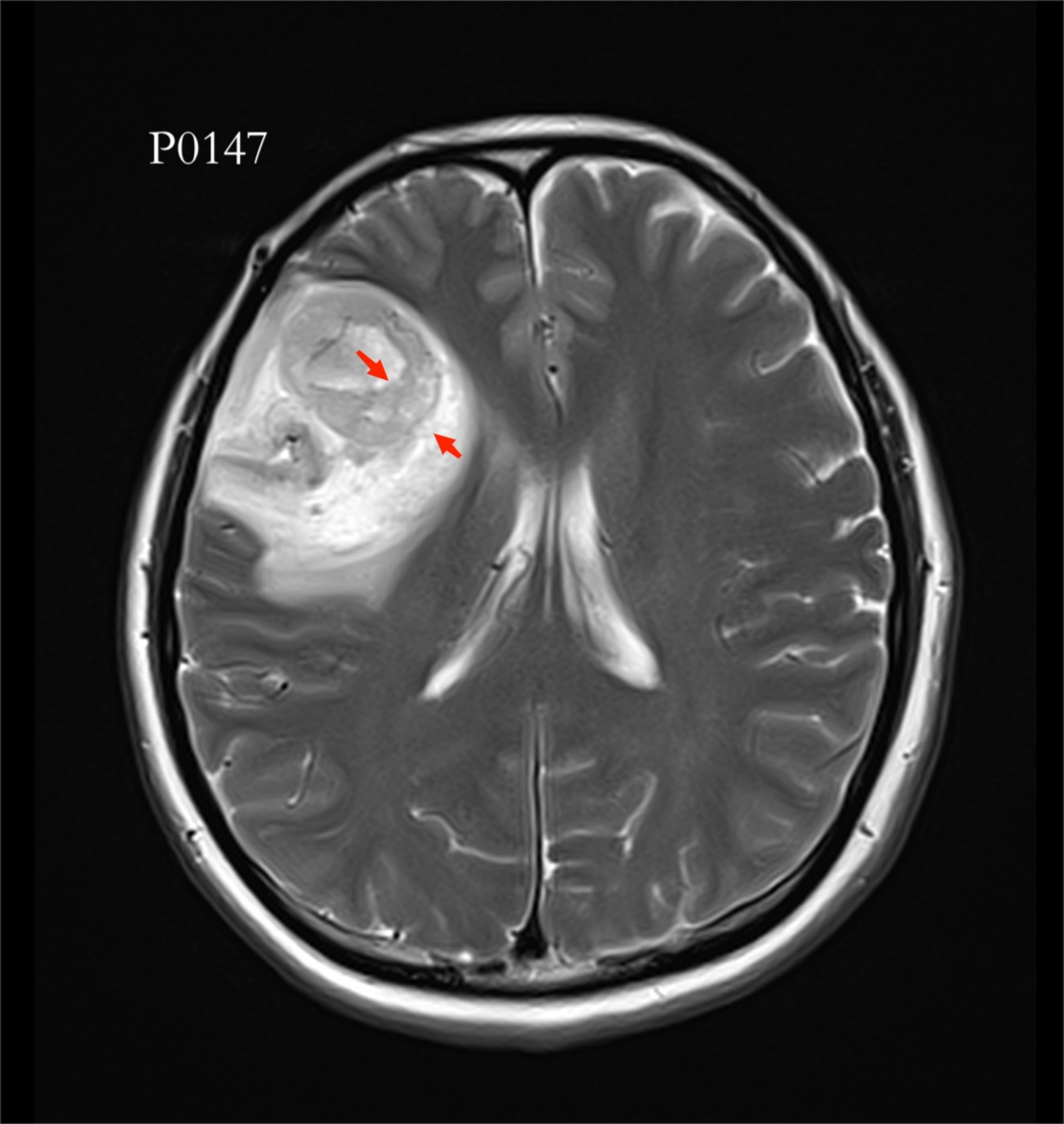
P0147

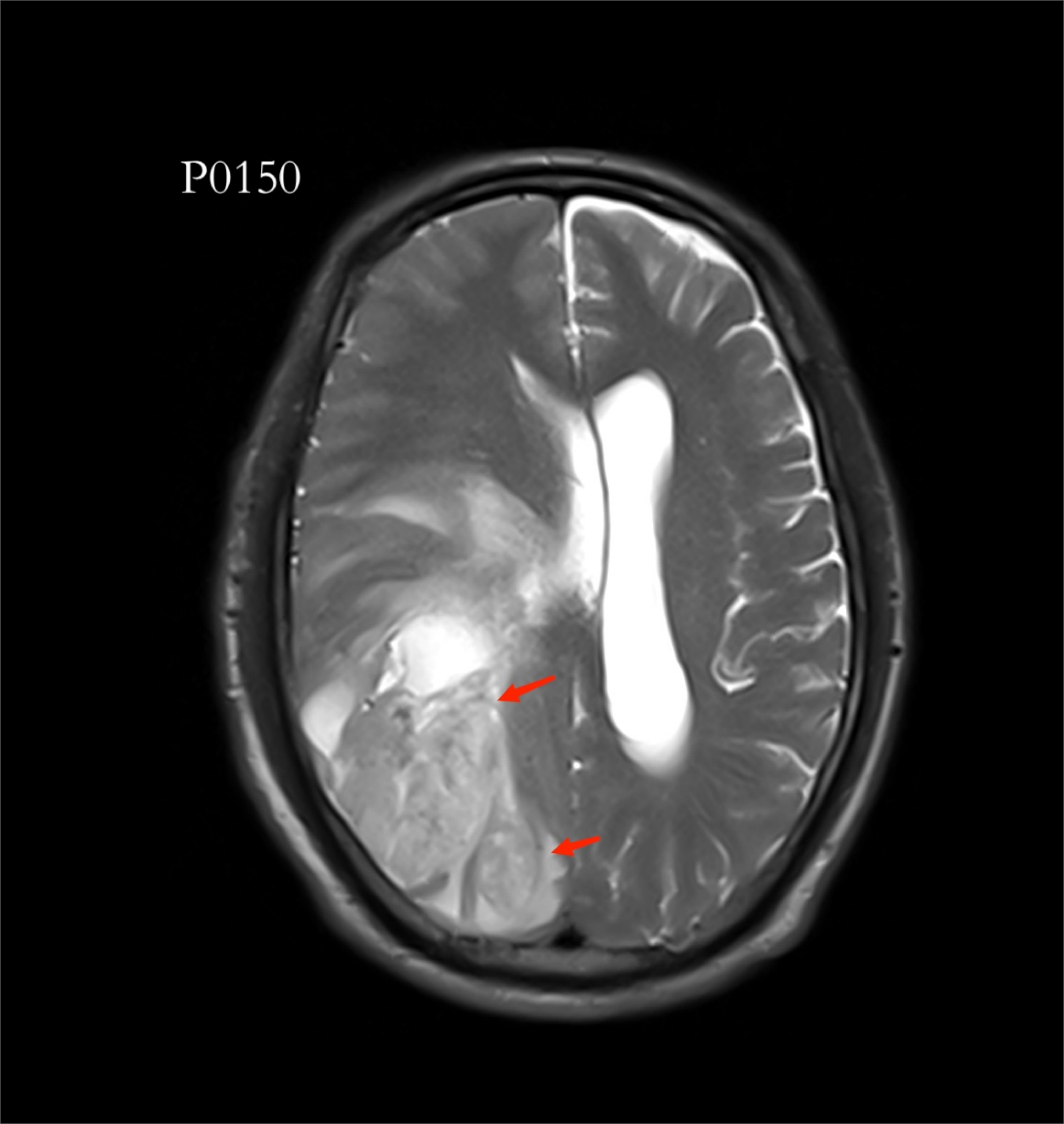
P0150

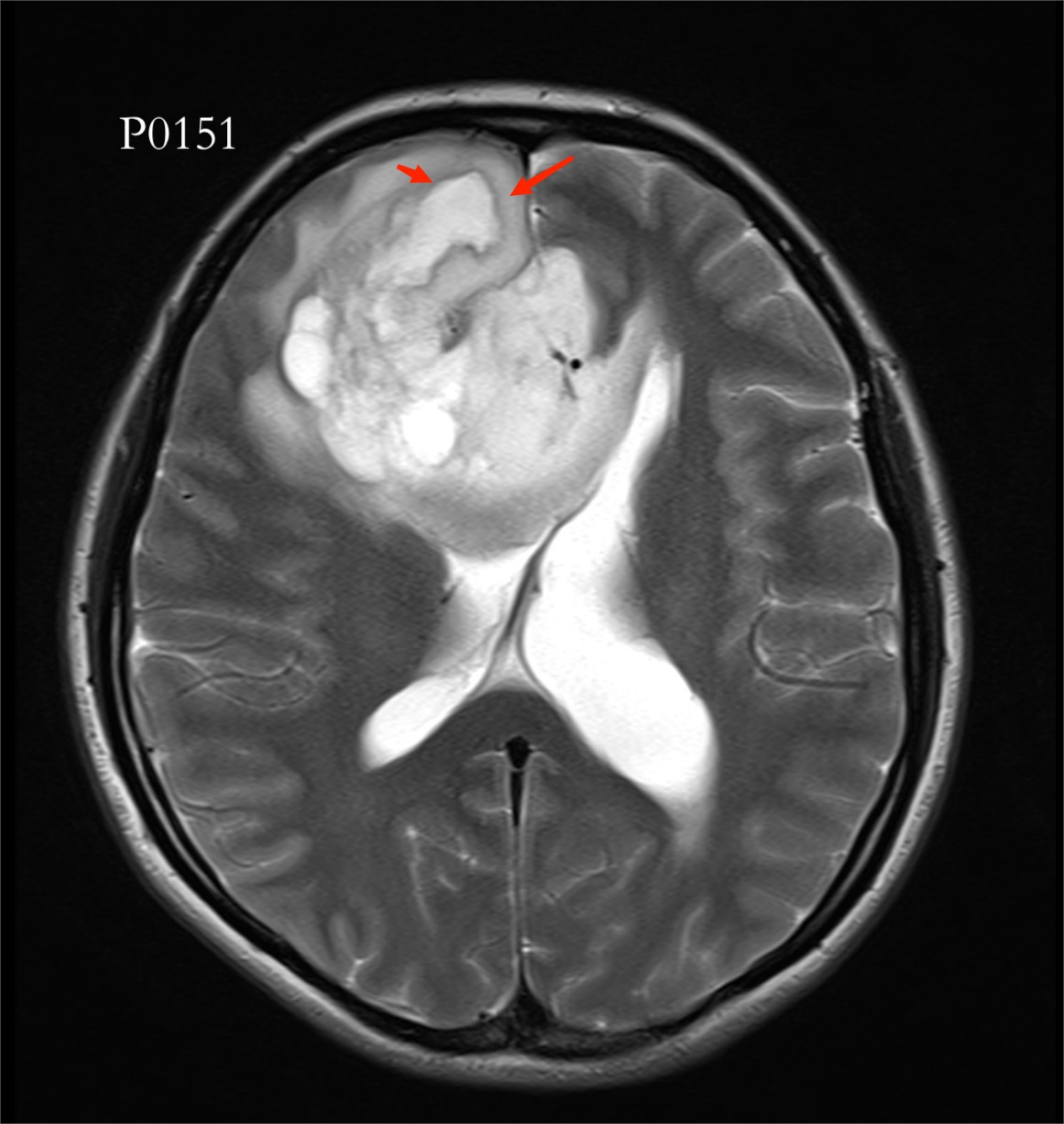
P0151

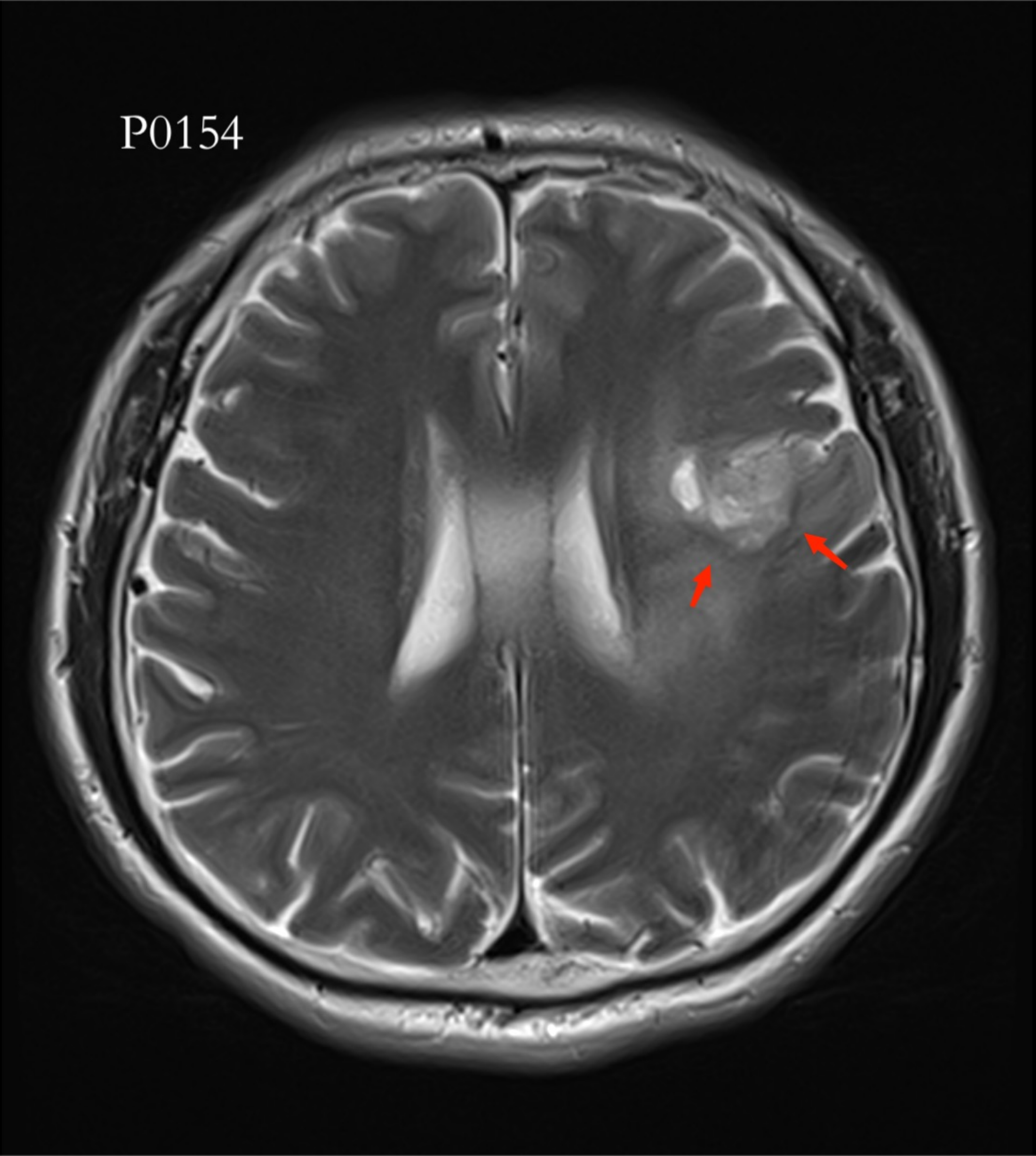
P0154

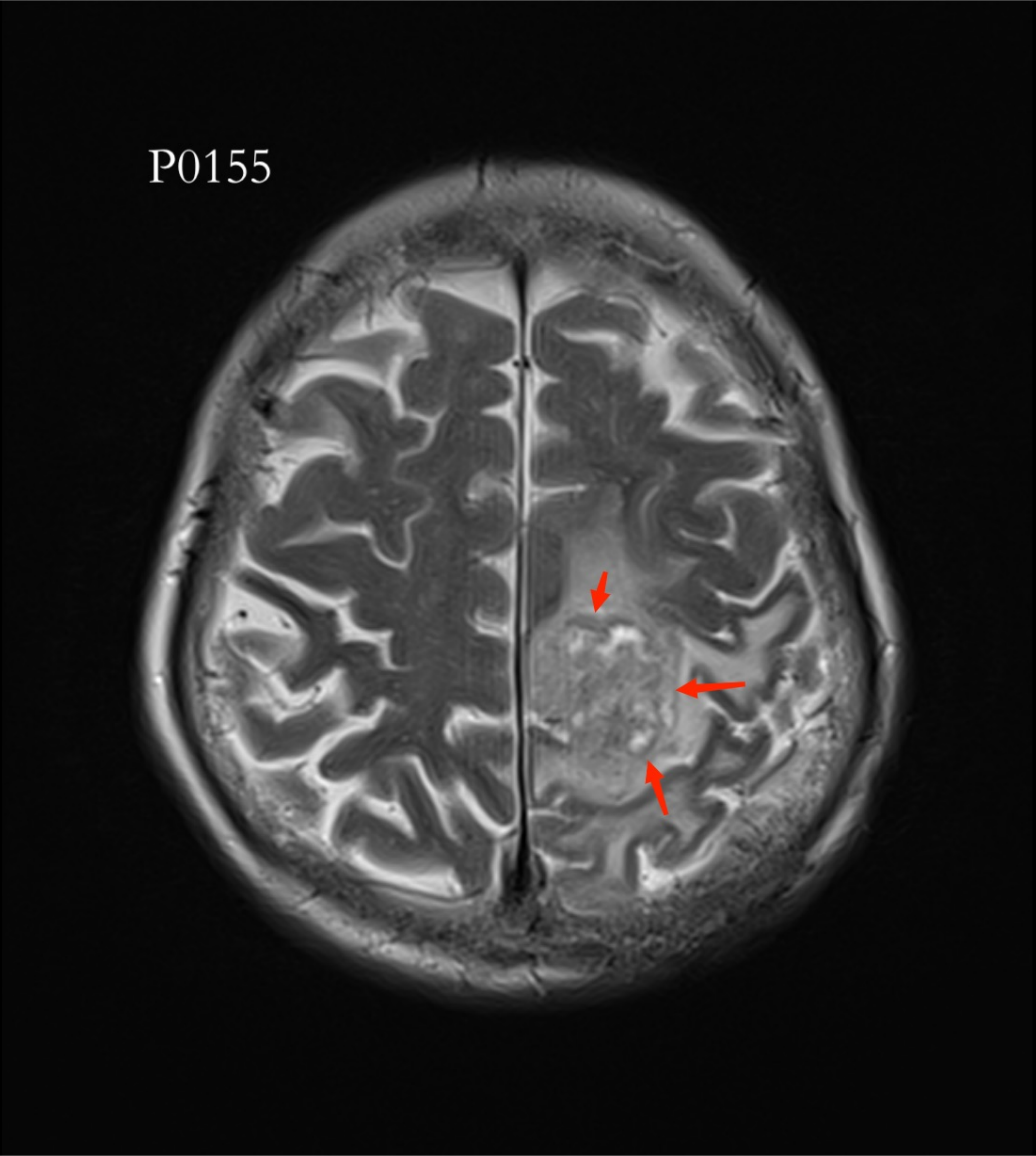
P0155

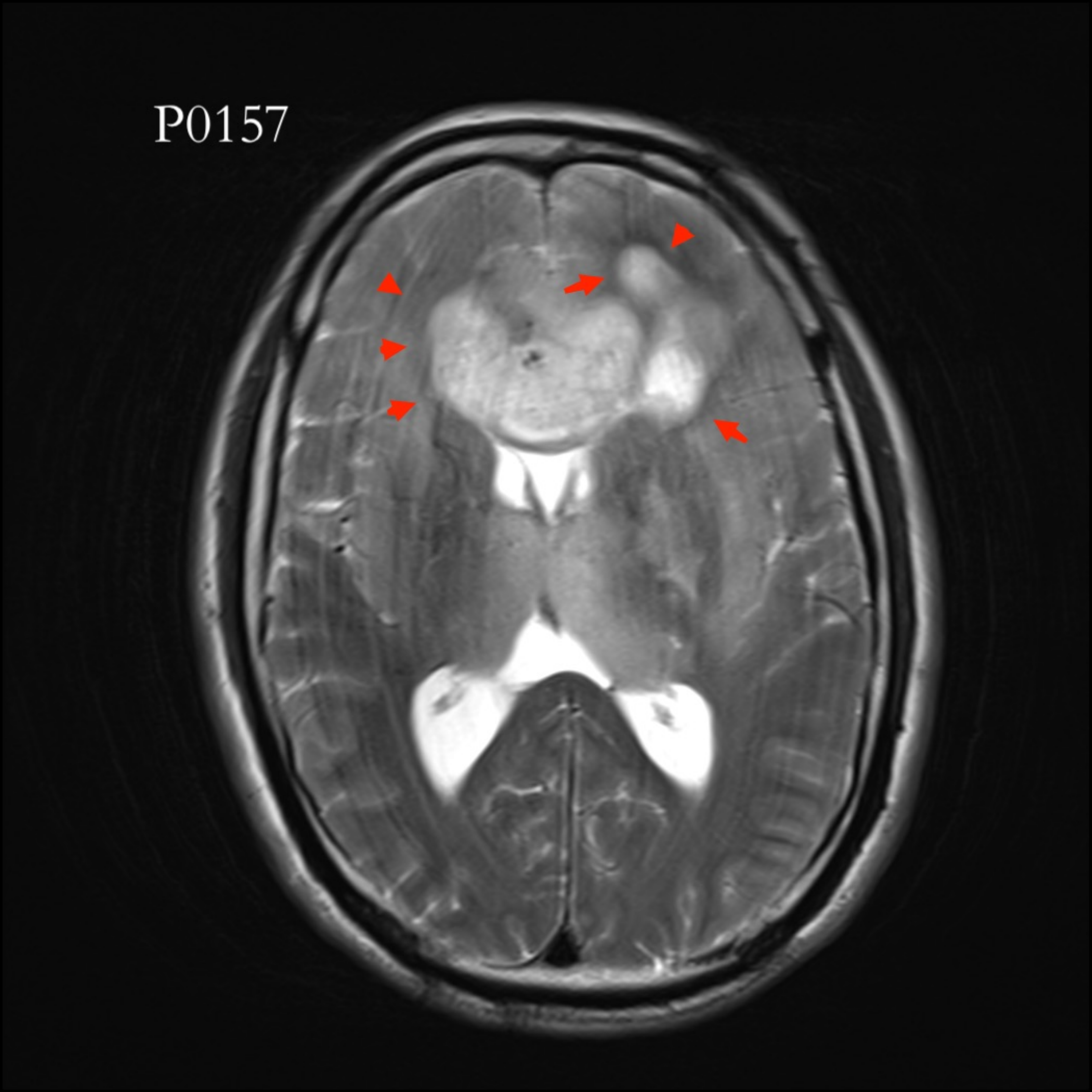
P0157

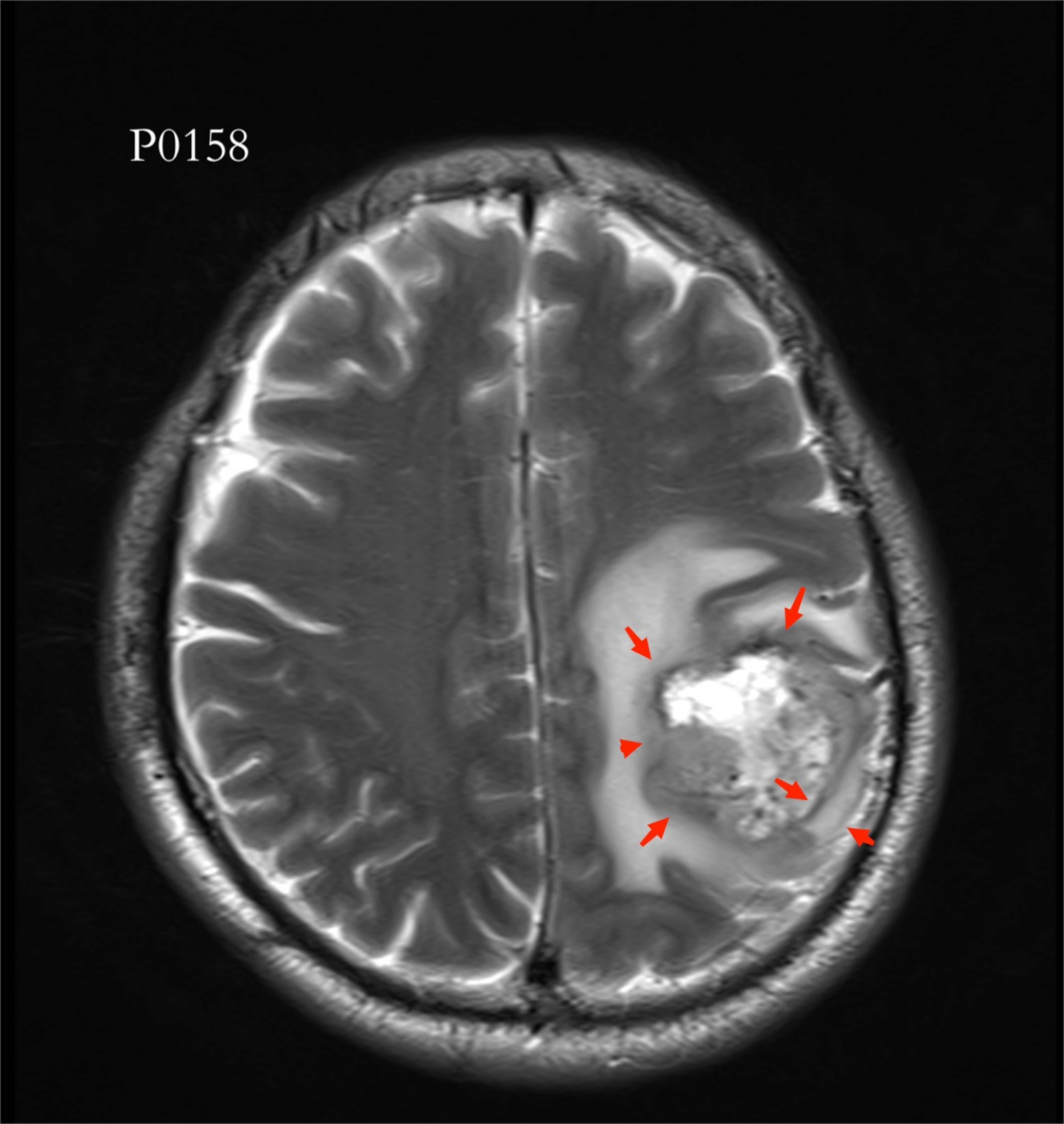
P0158

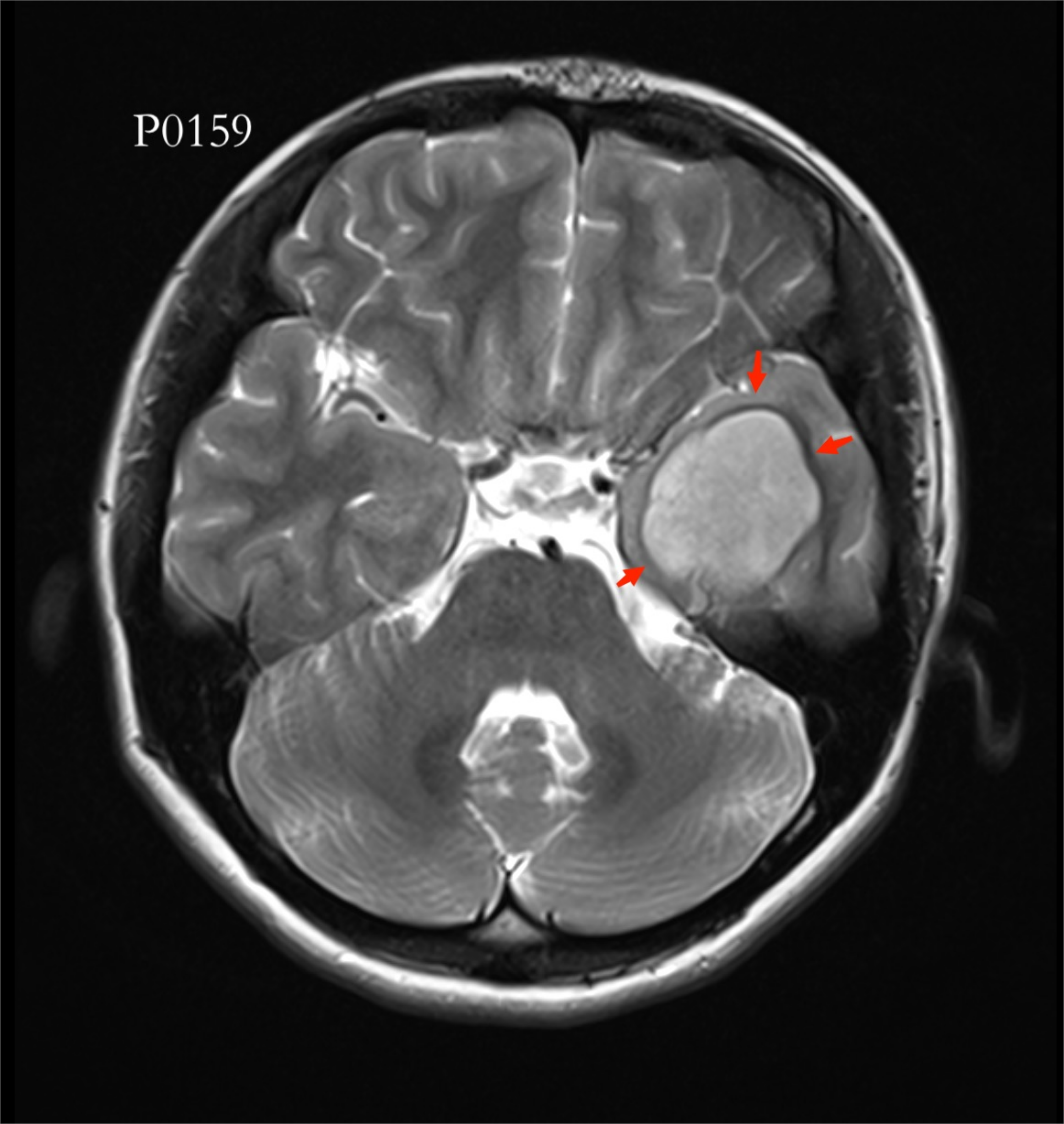
P0159

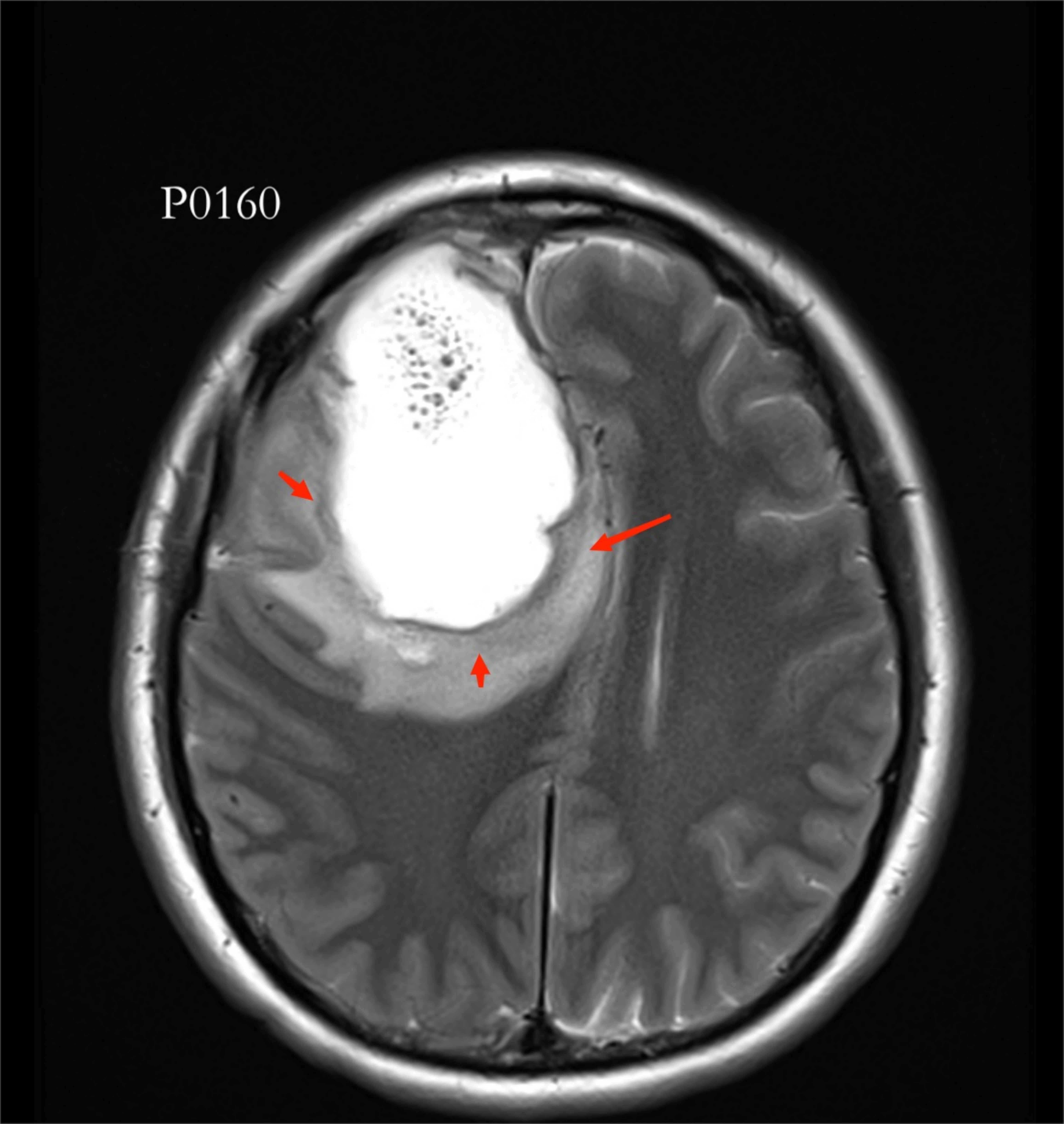
P0160

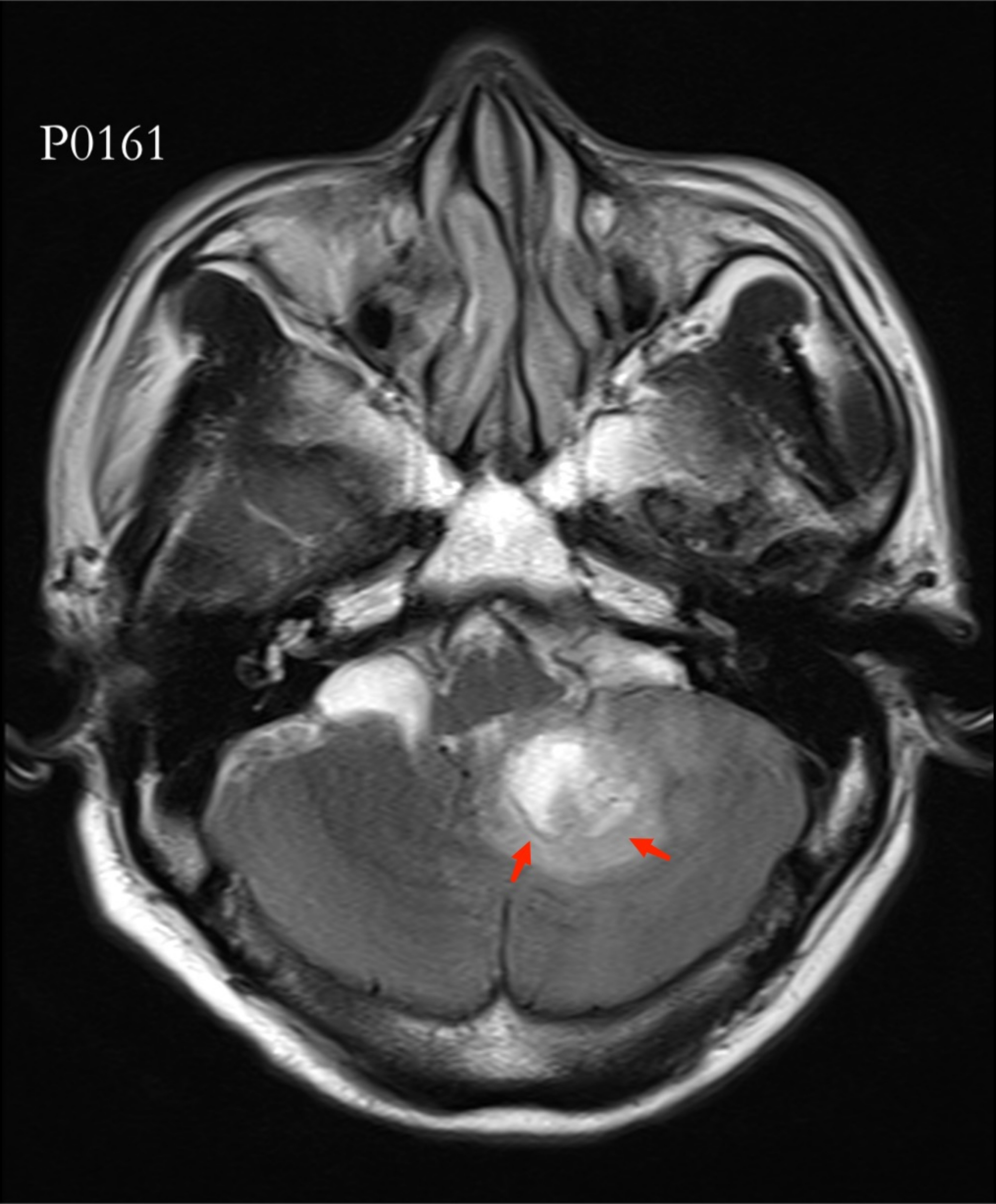
P0161

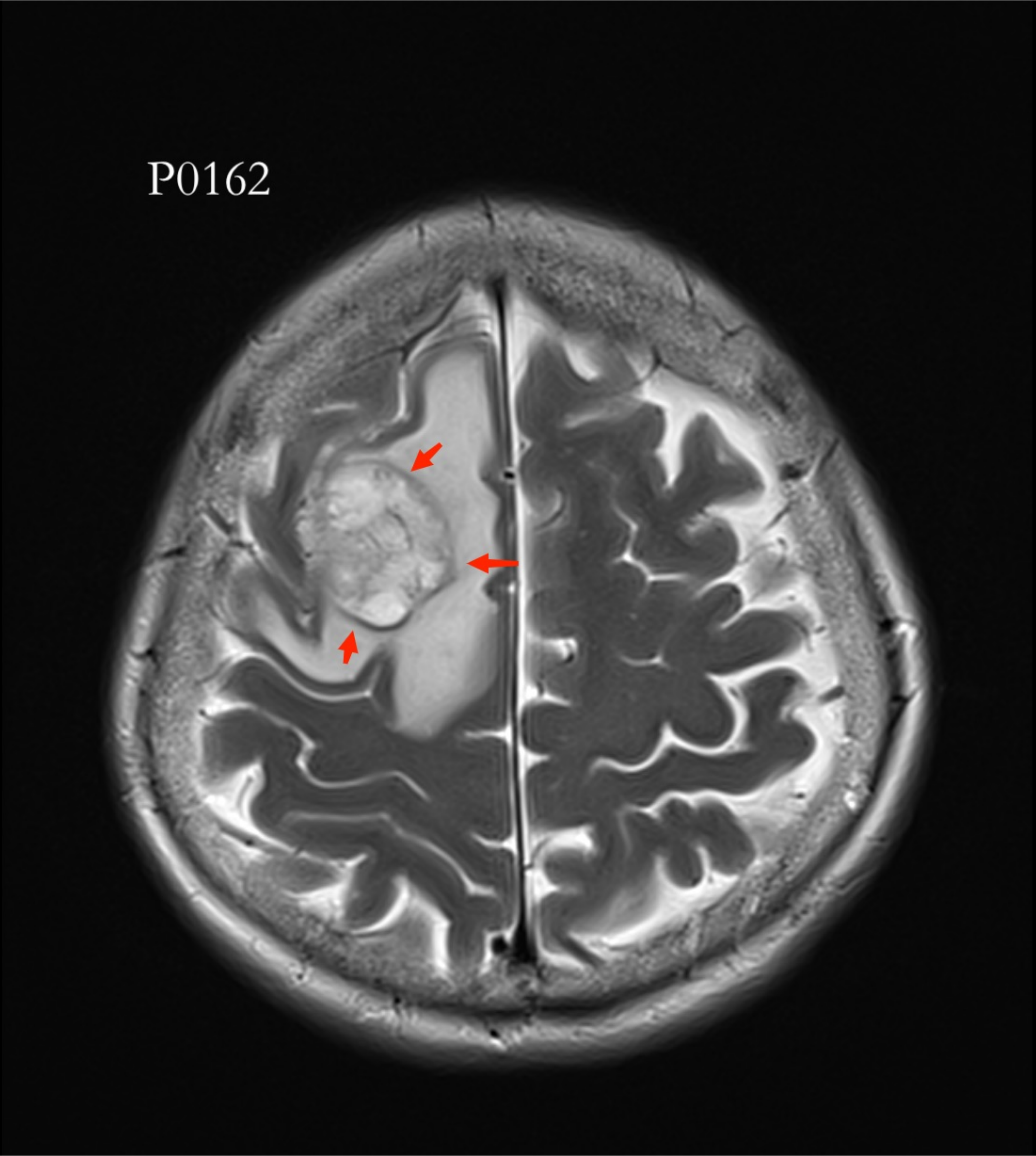
P0162

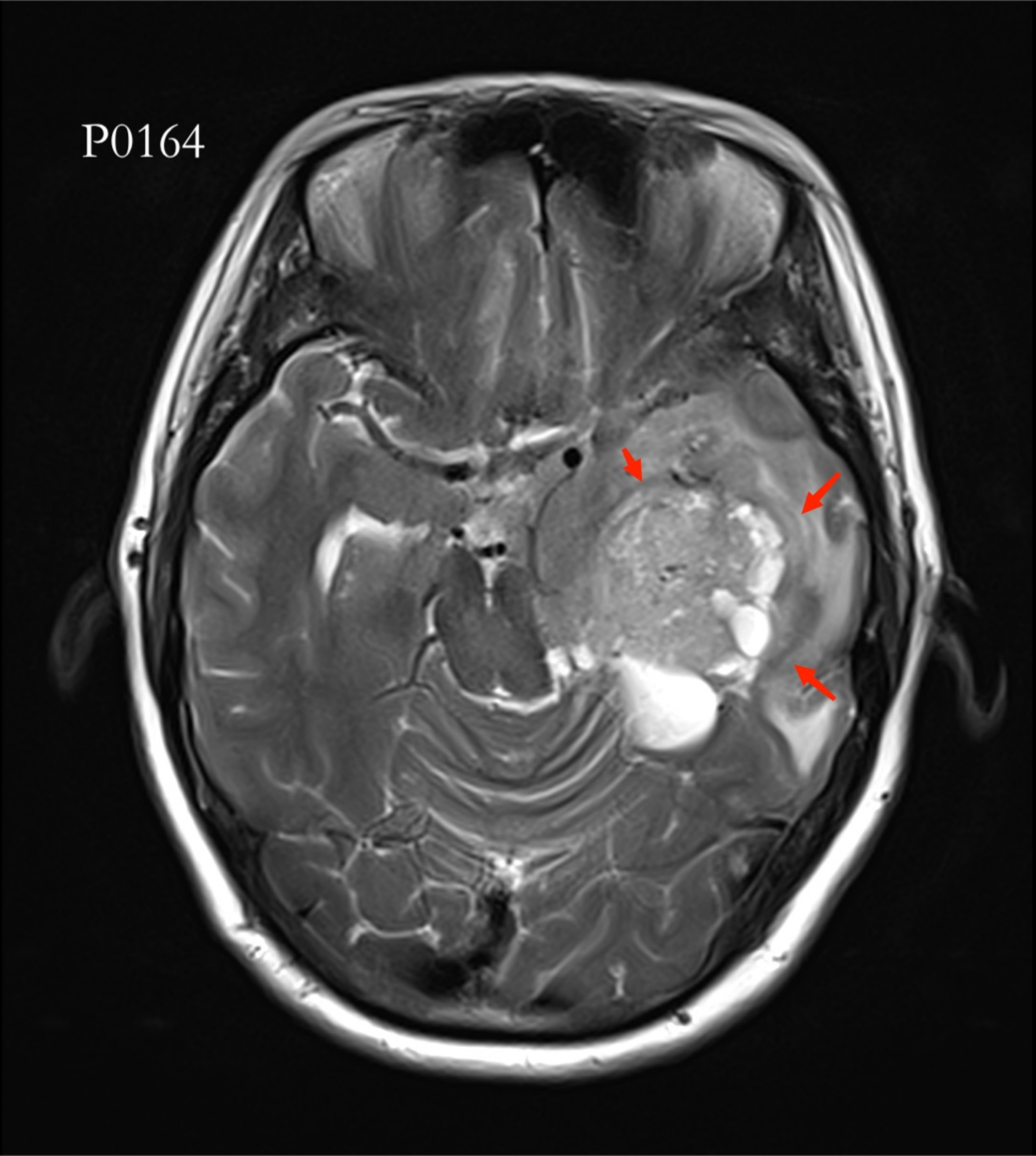
P0164

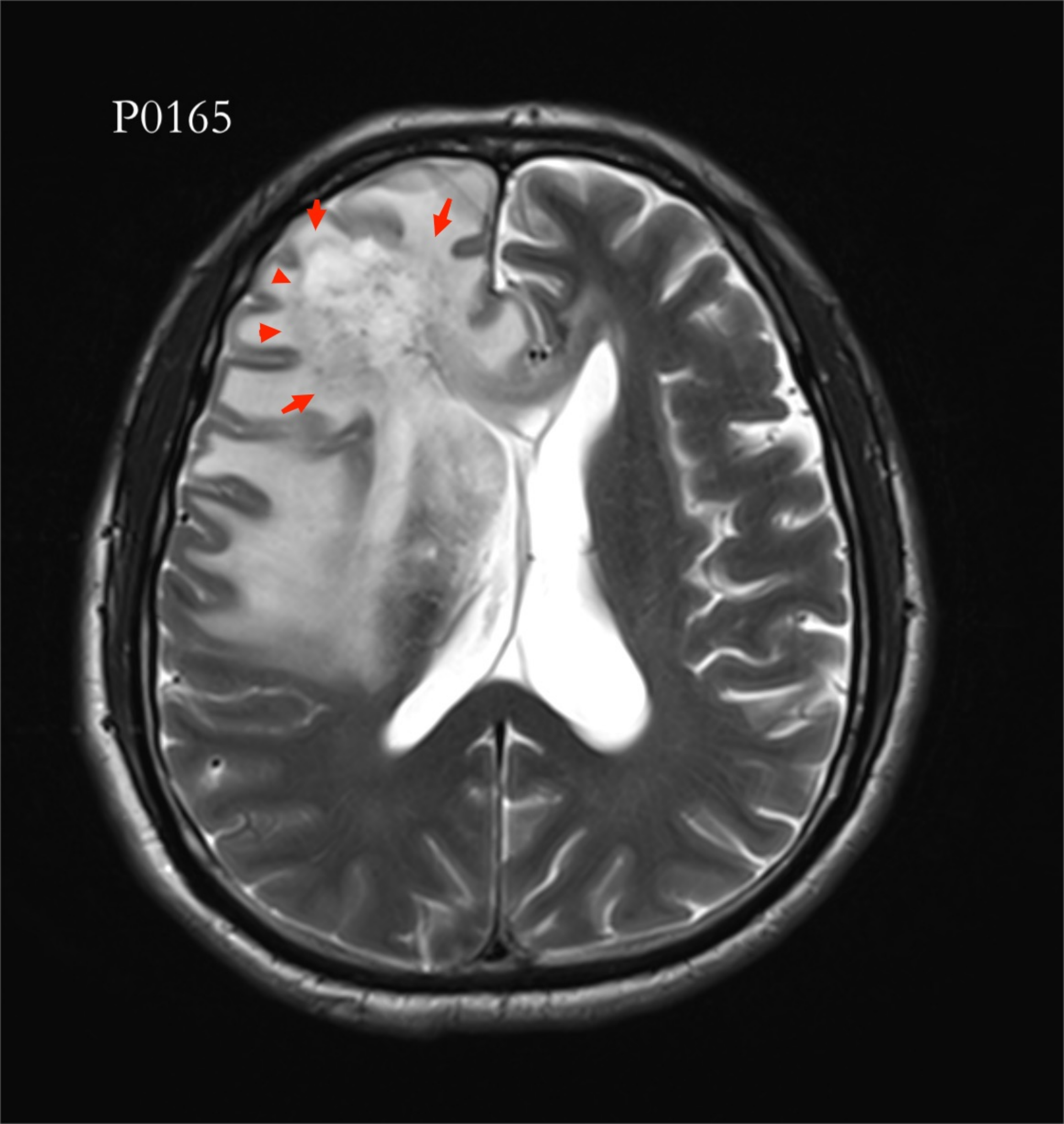
P0165

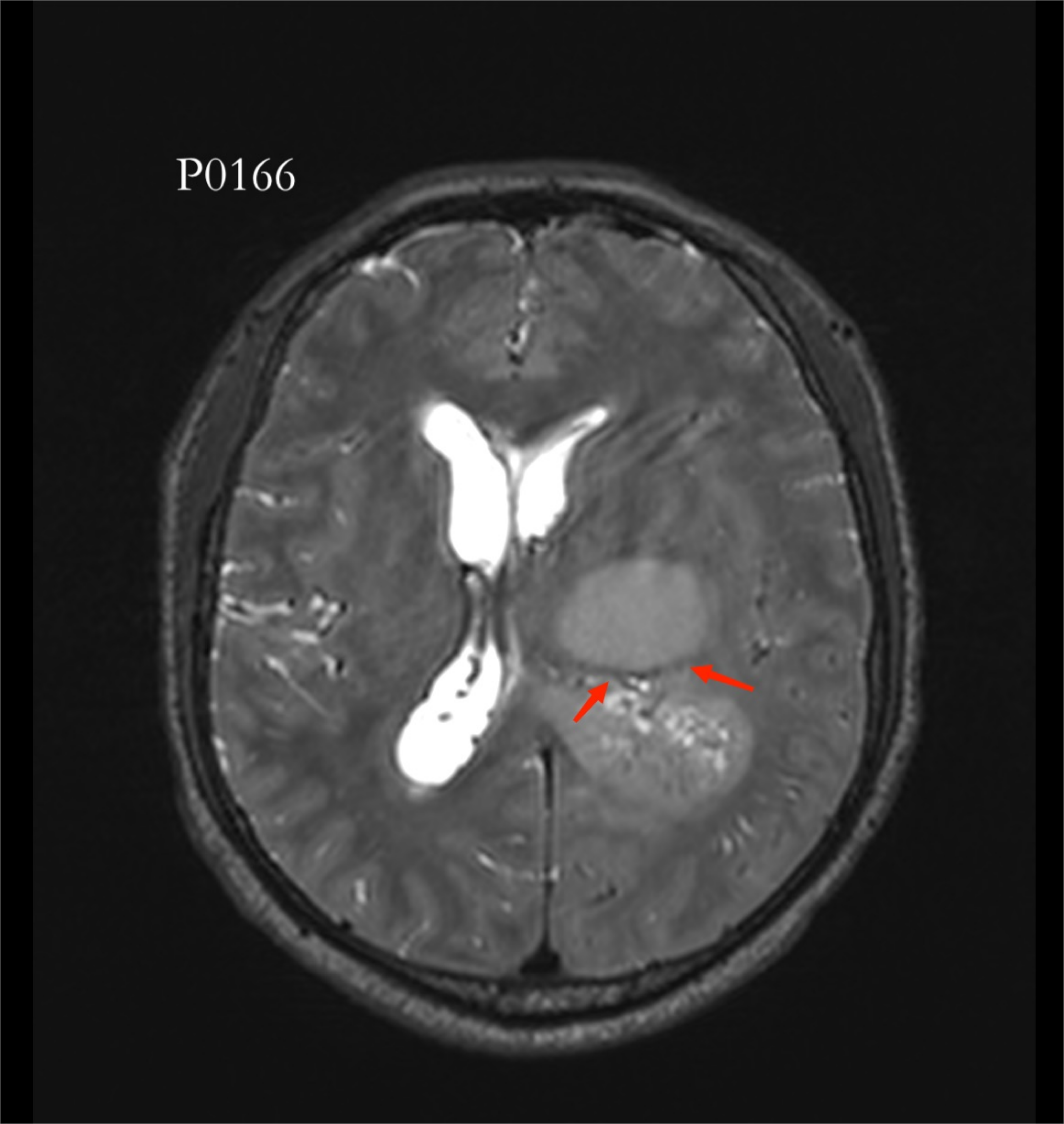
P0166

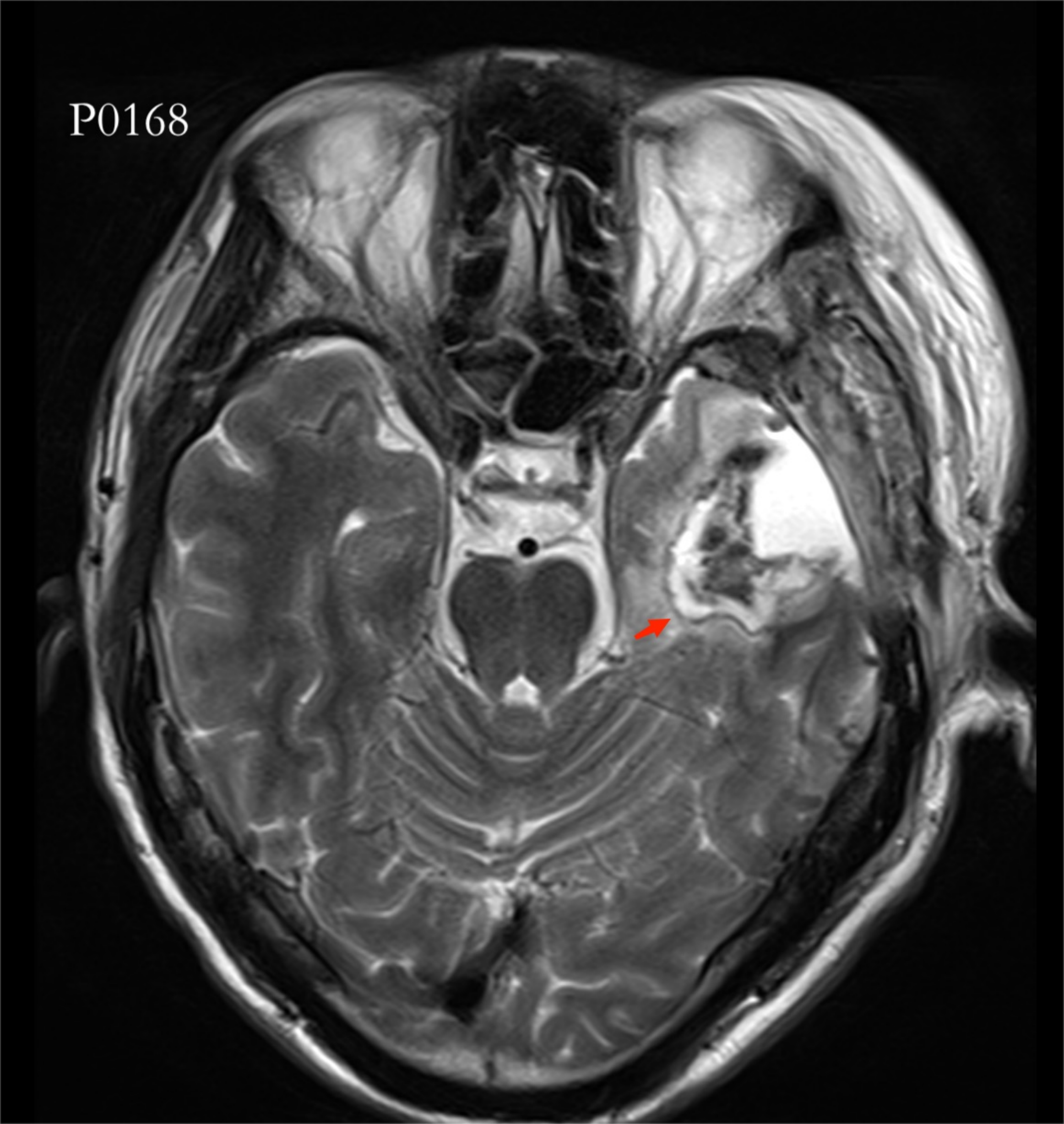
P0168

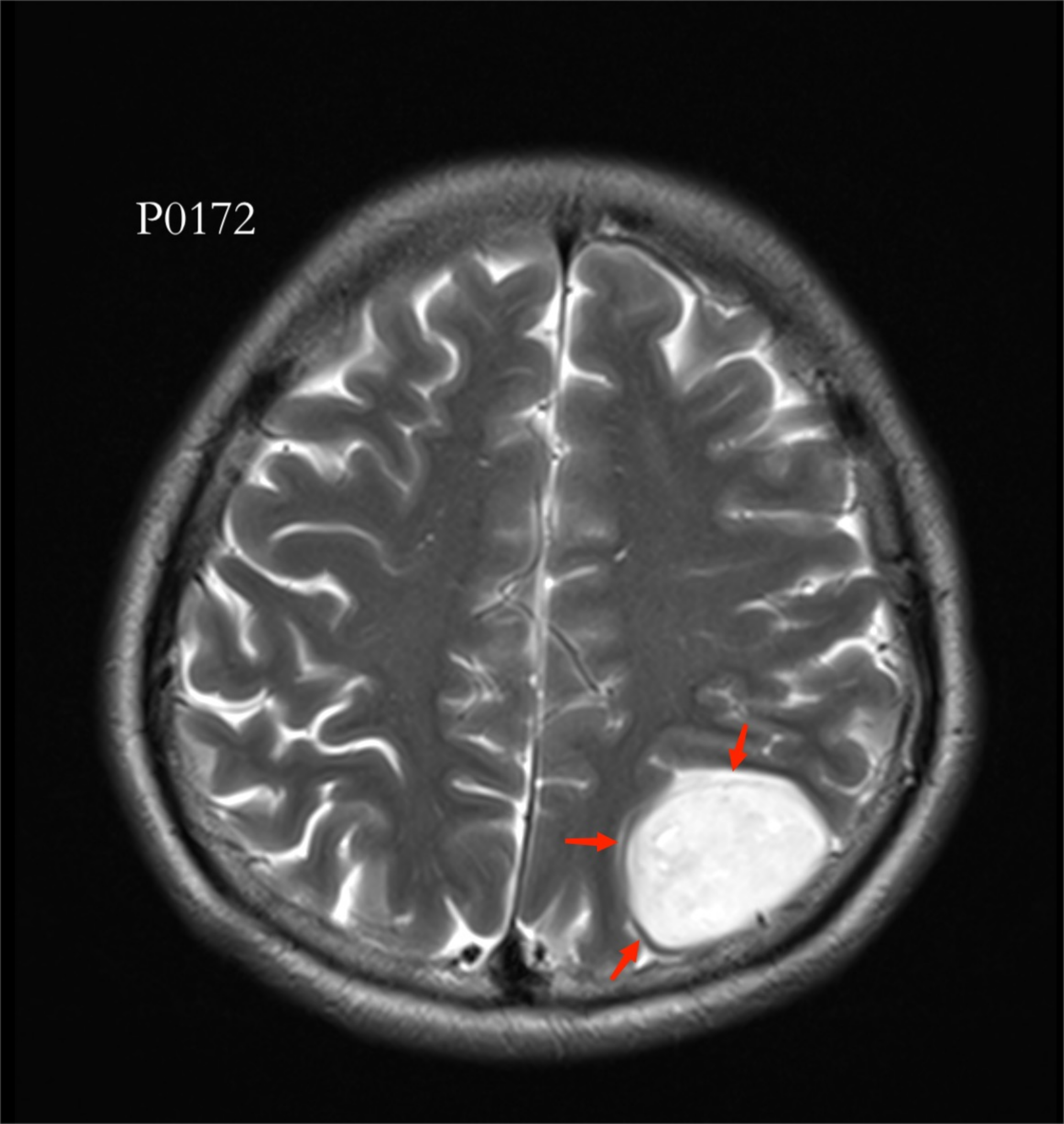
P0172

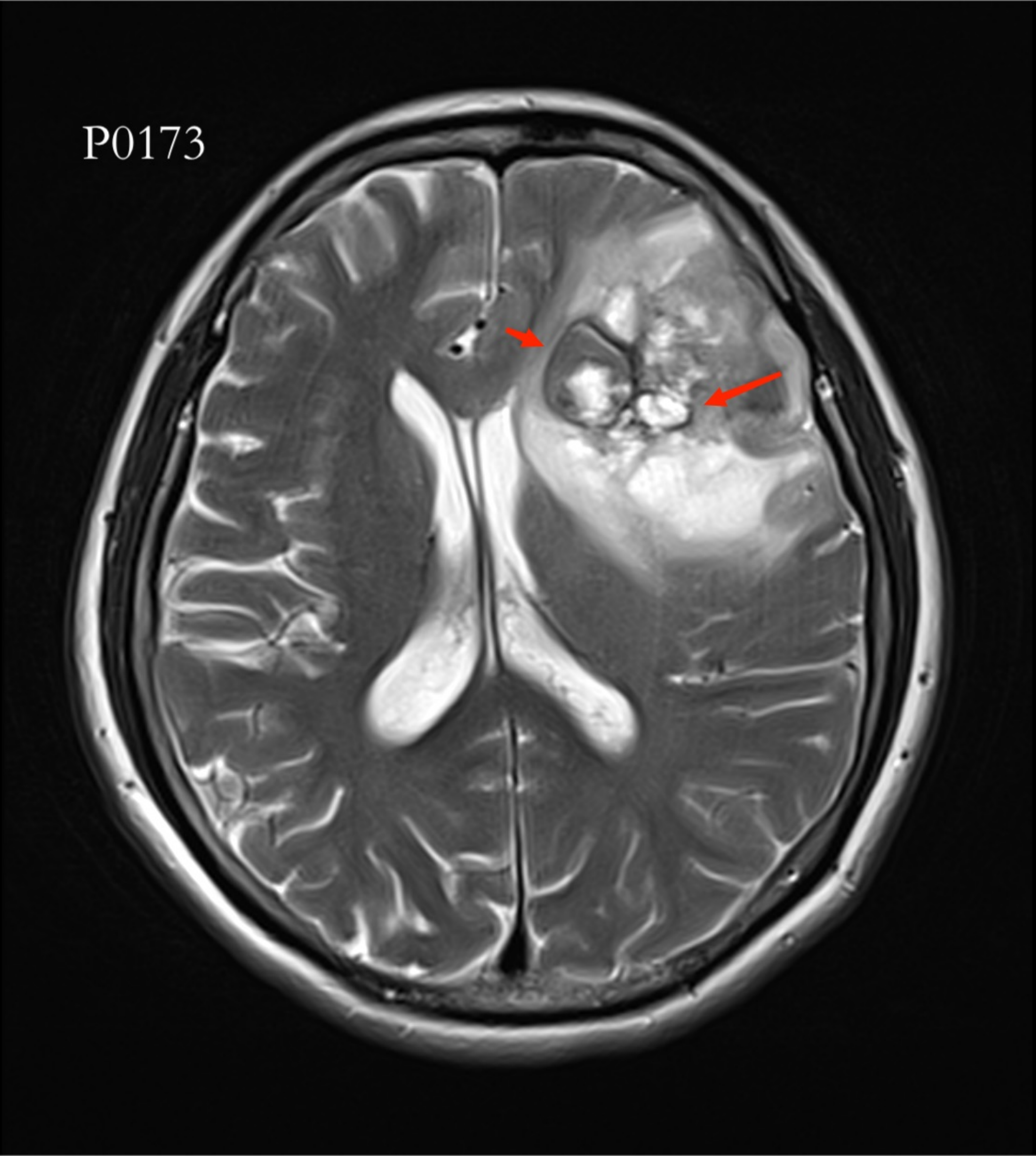
P0173

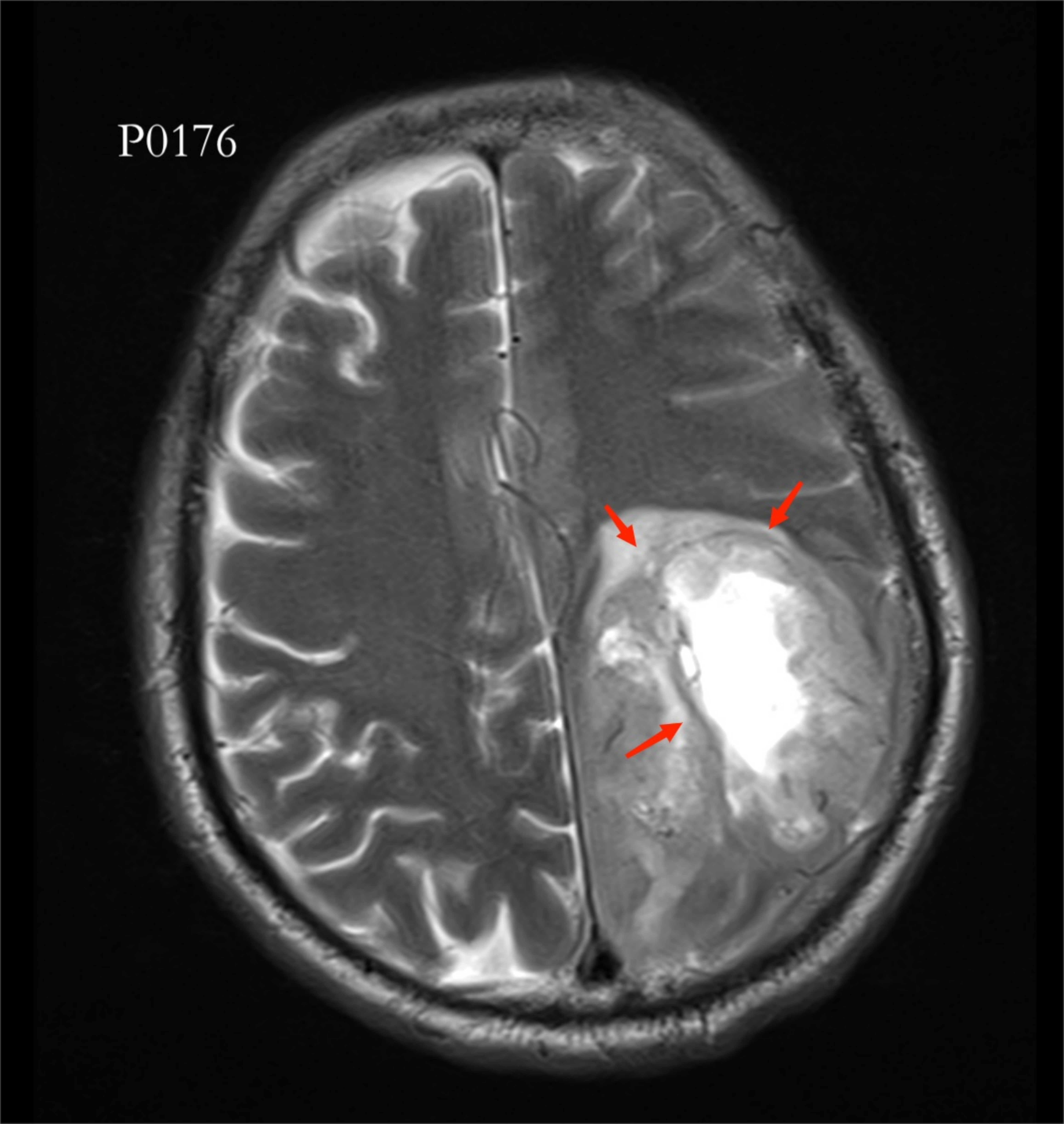
P0176

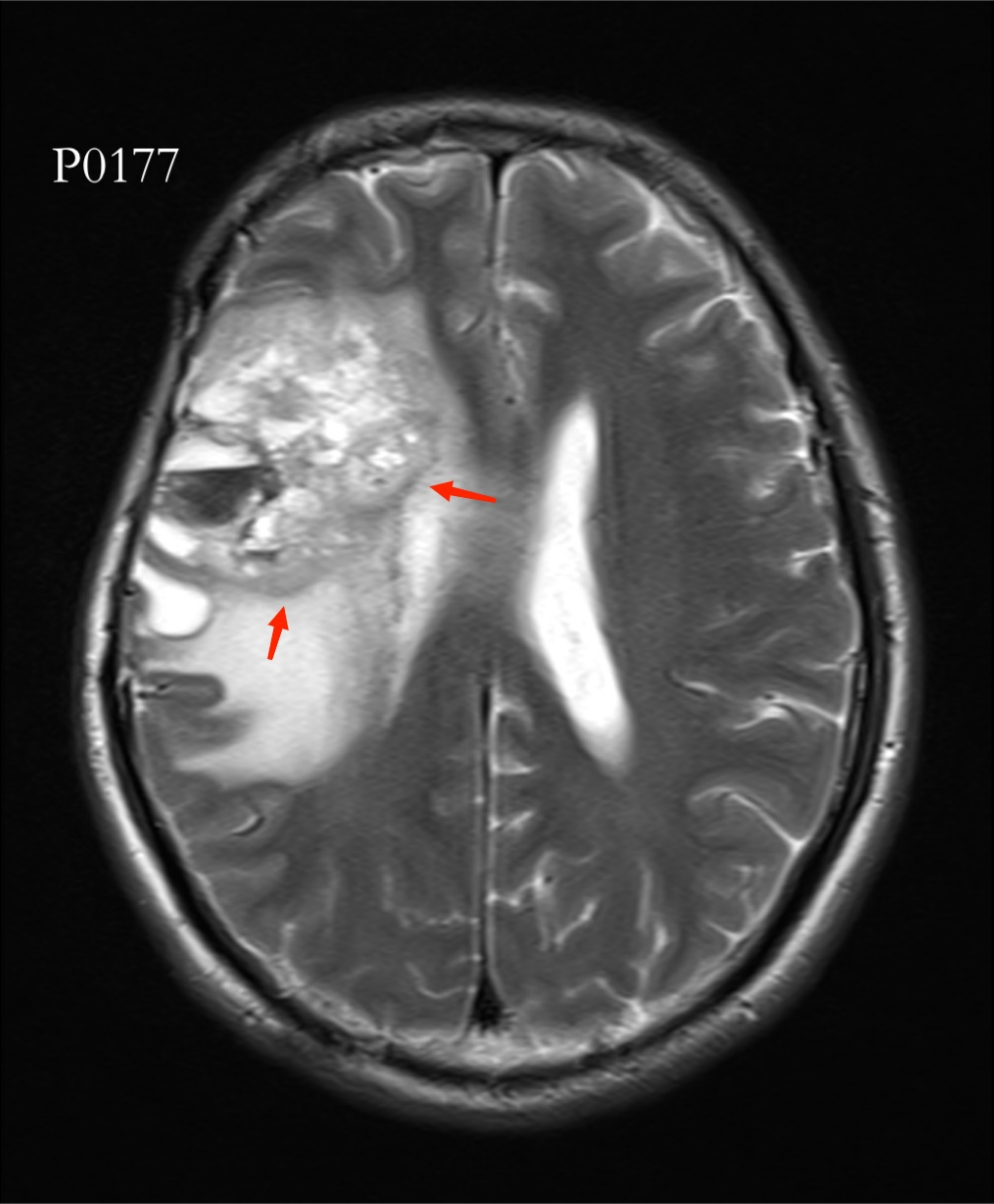
P0177

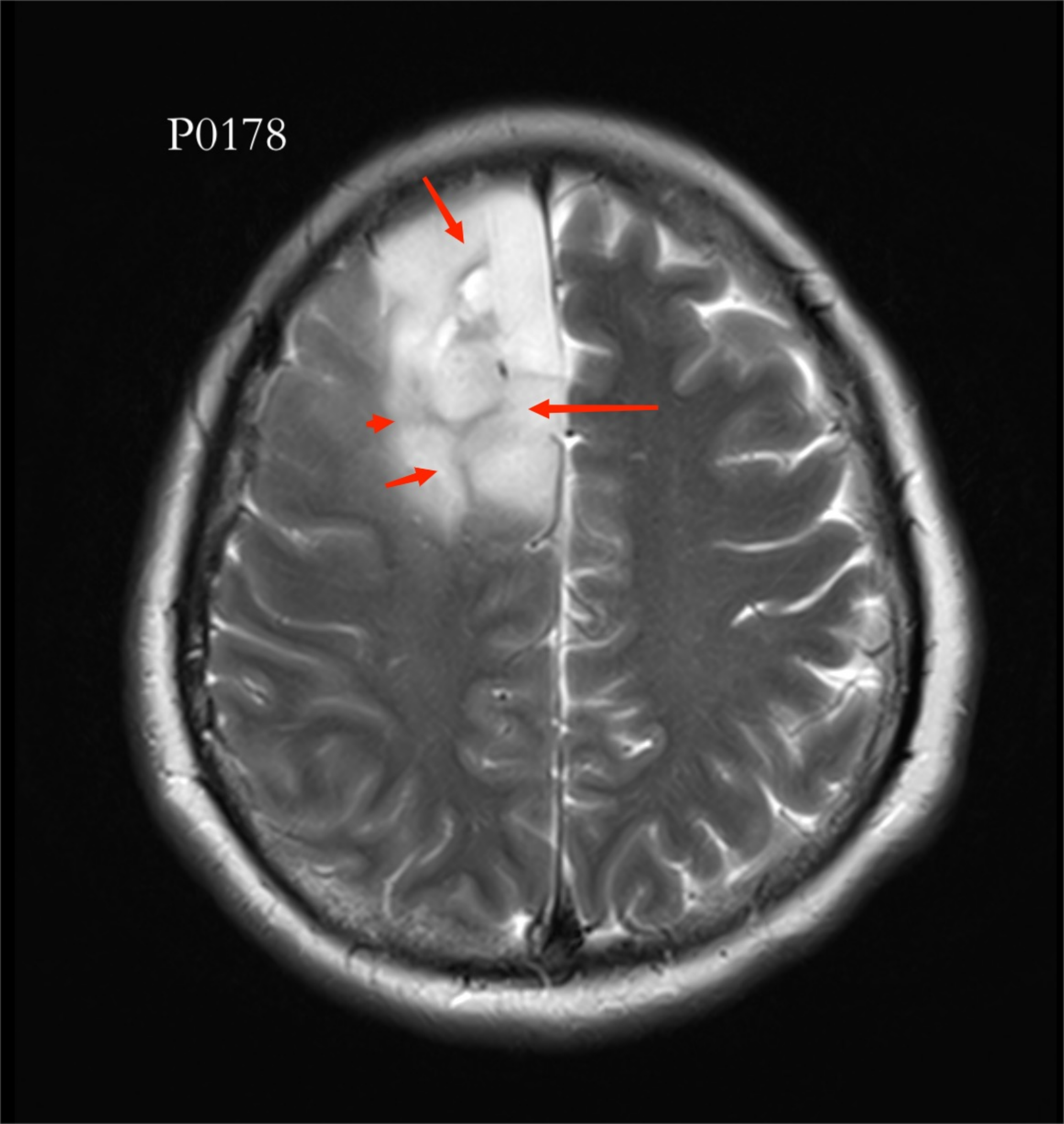
P0178

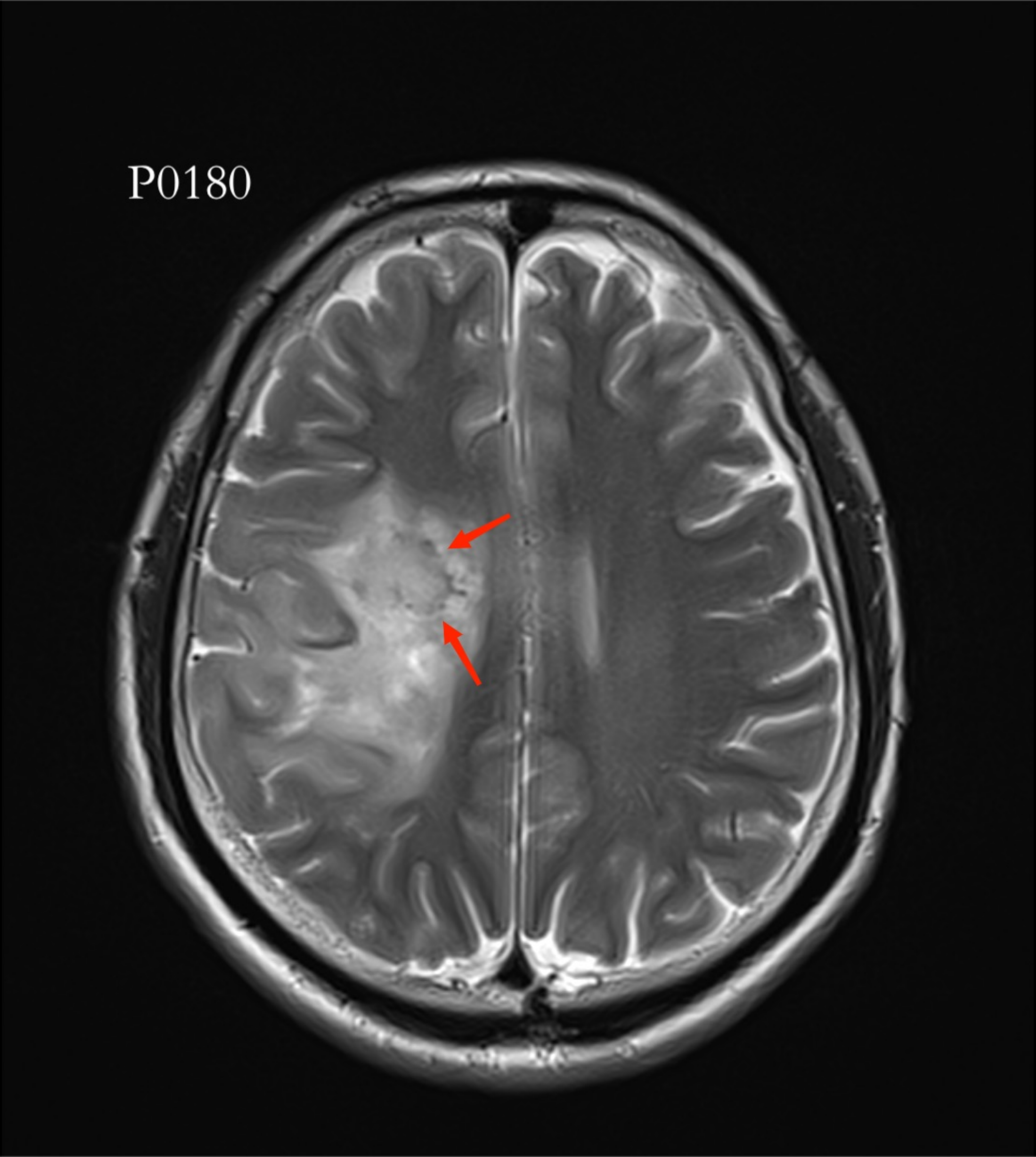
P0180

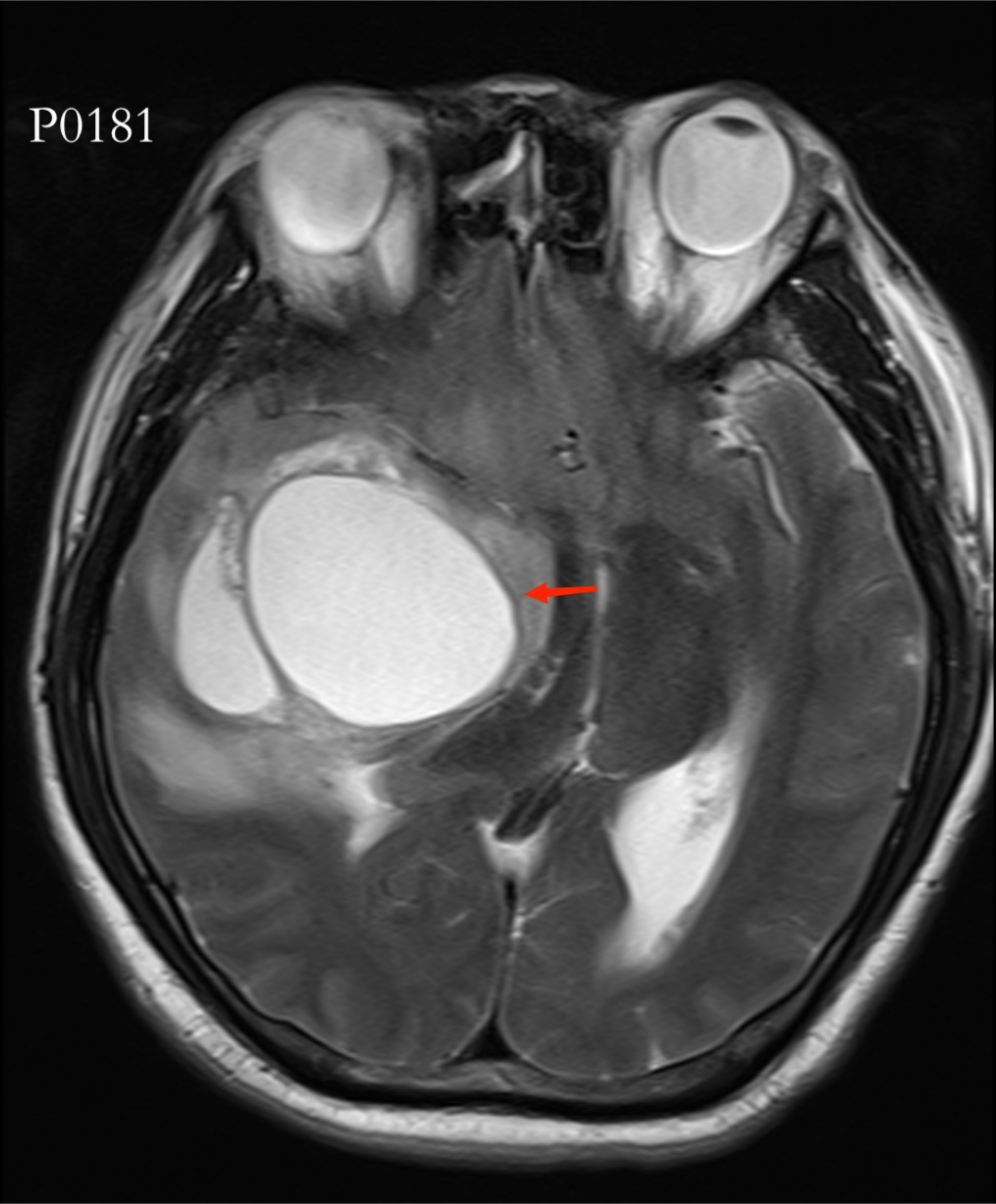
P0181

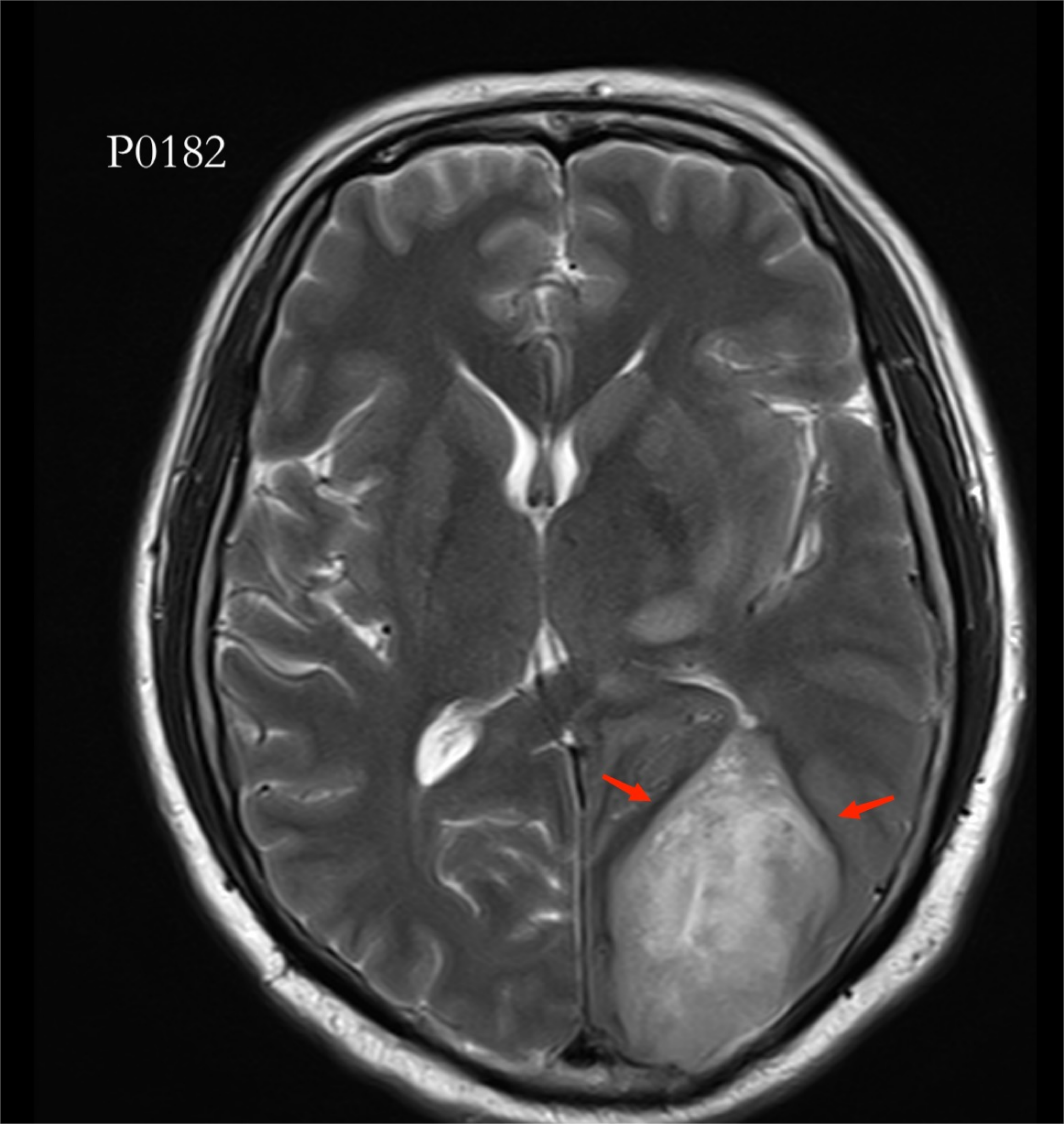
P0182

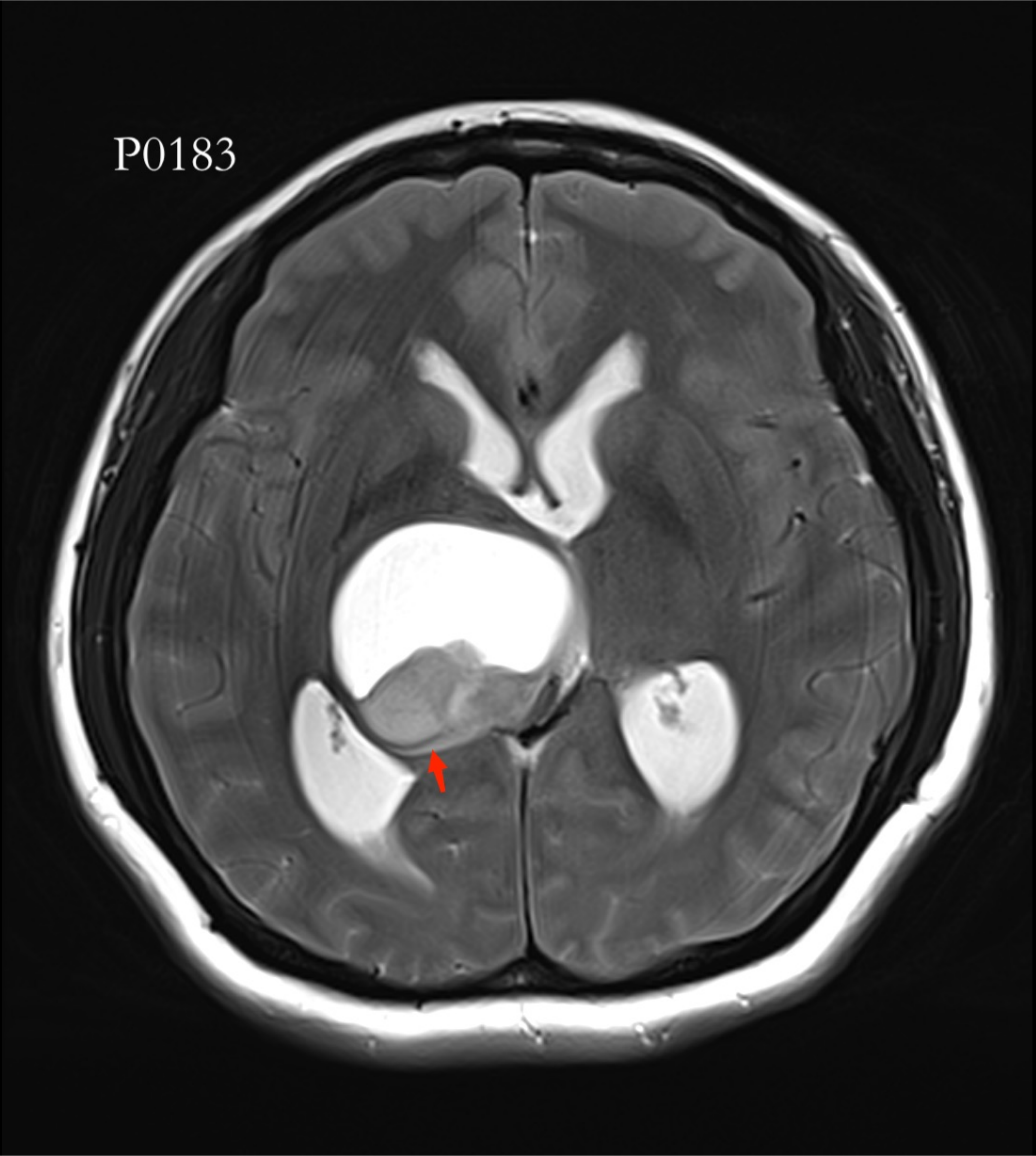
P0183

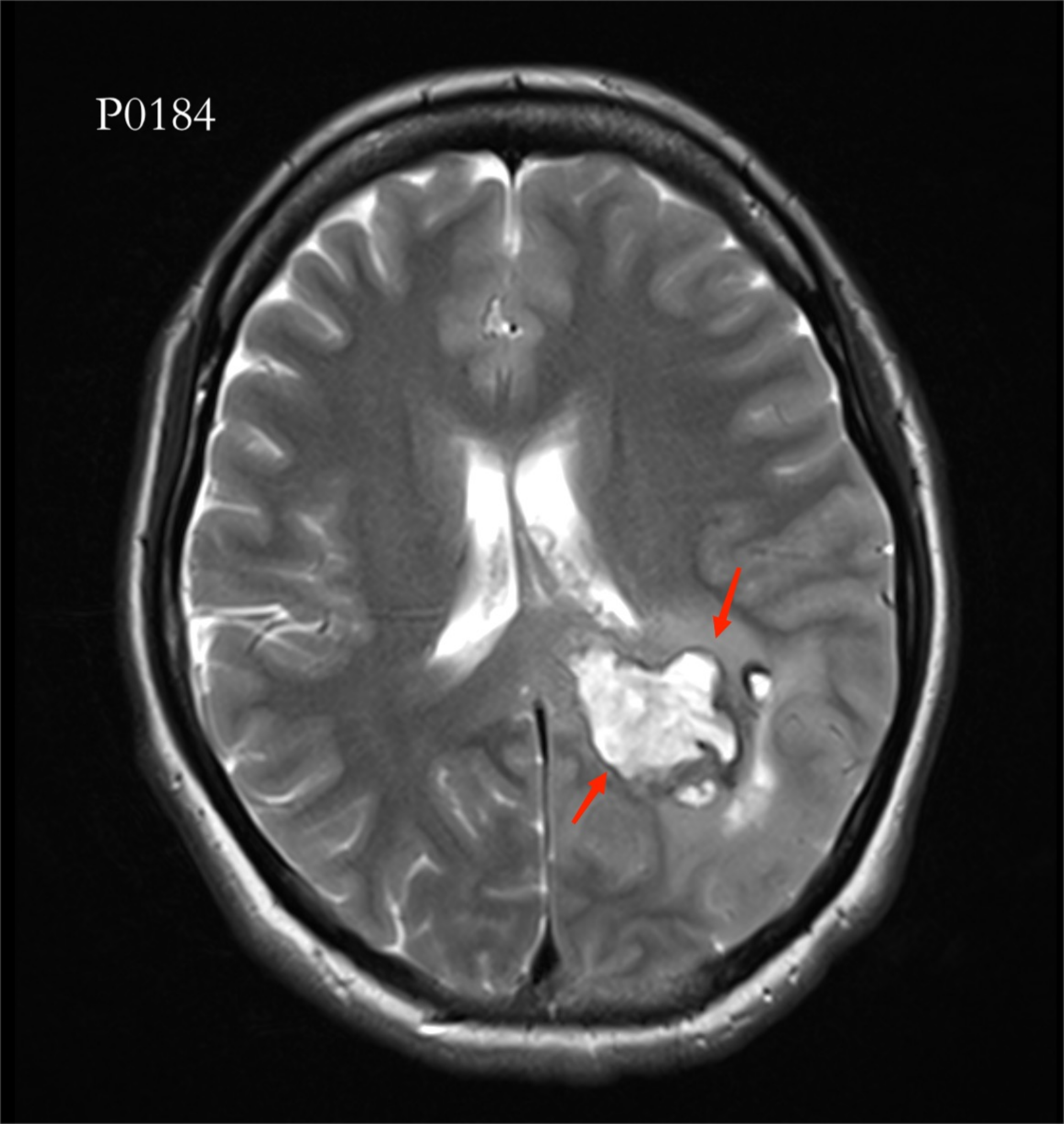
P0184

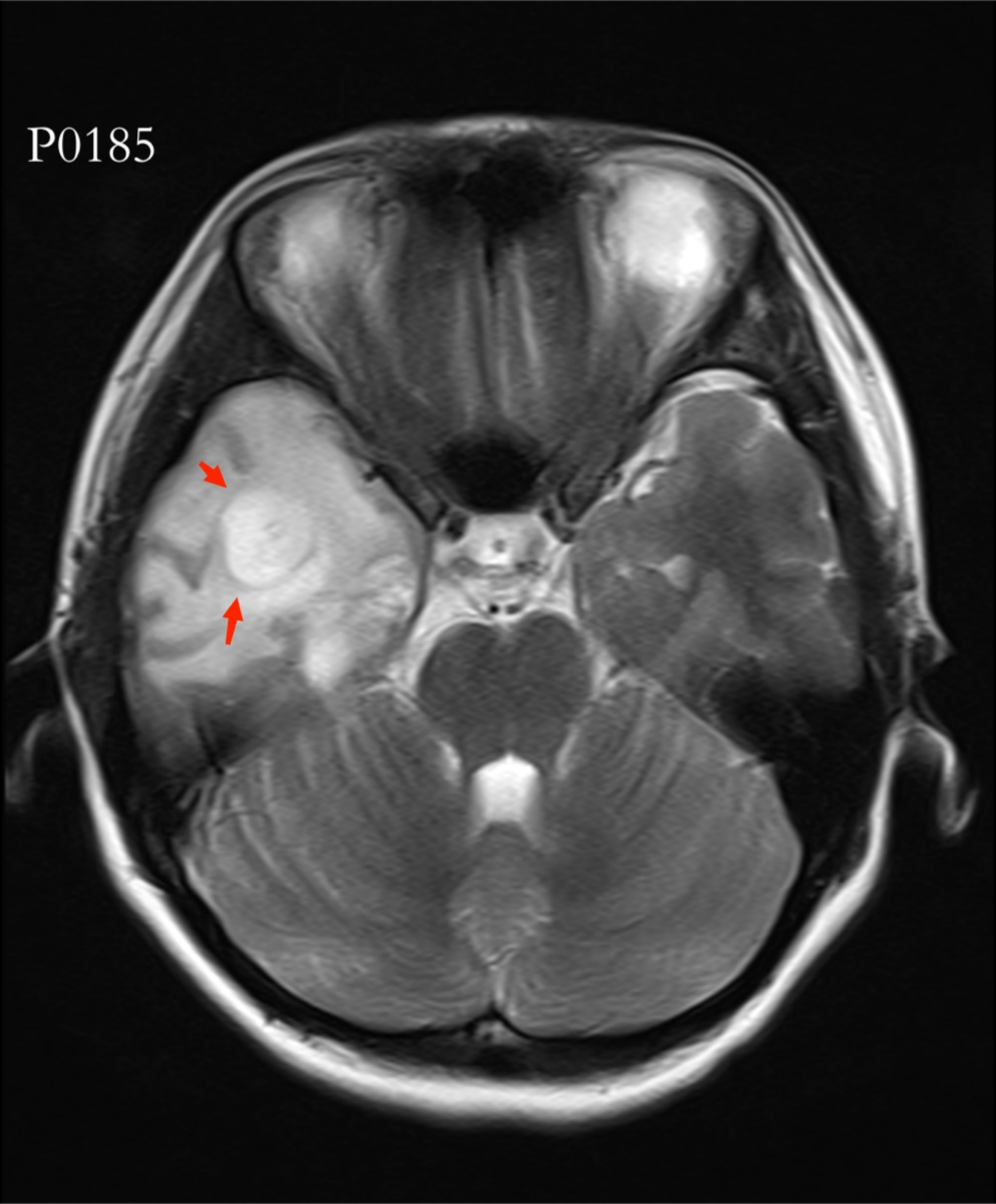
P0185

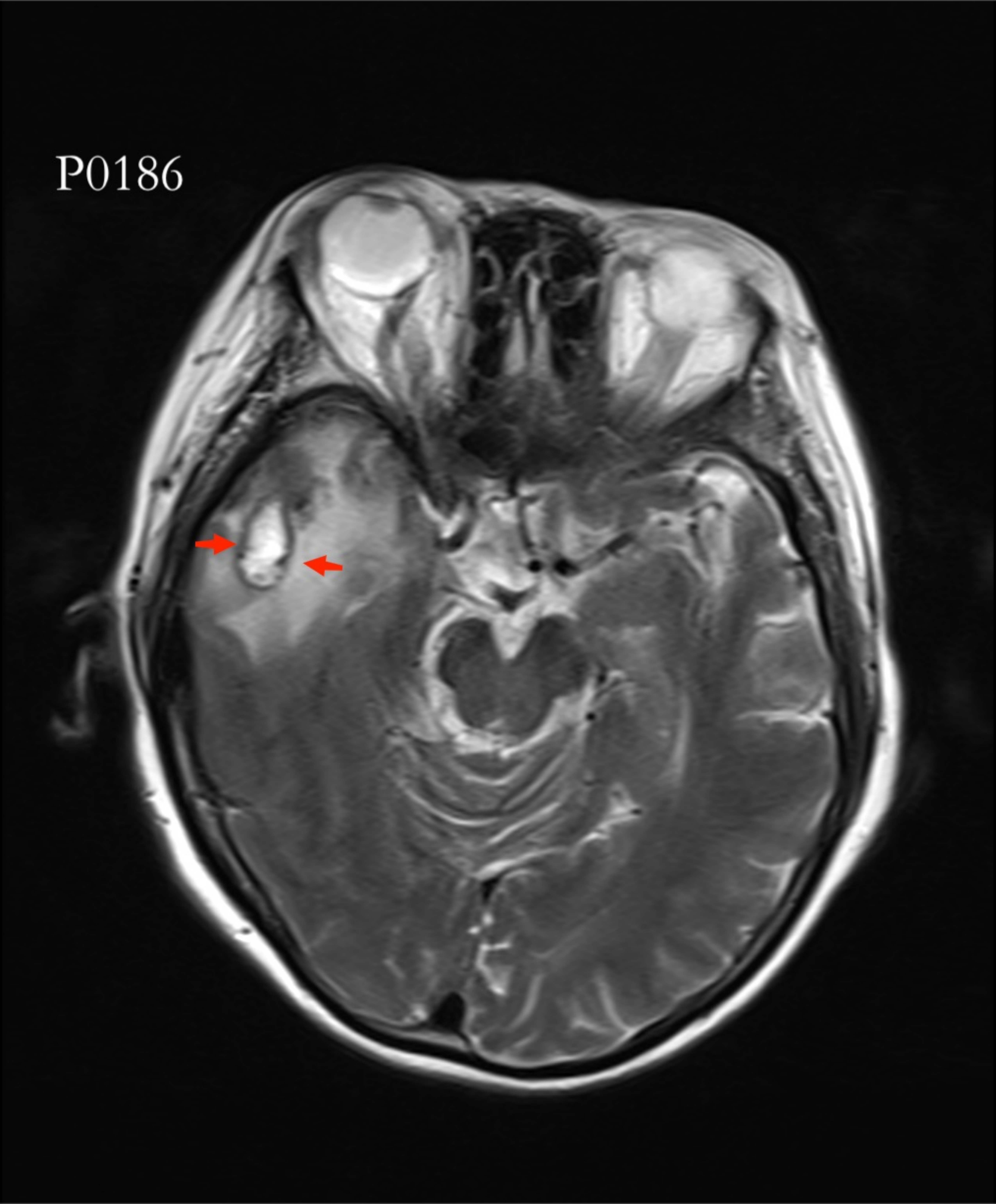
P0186

P0187

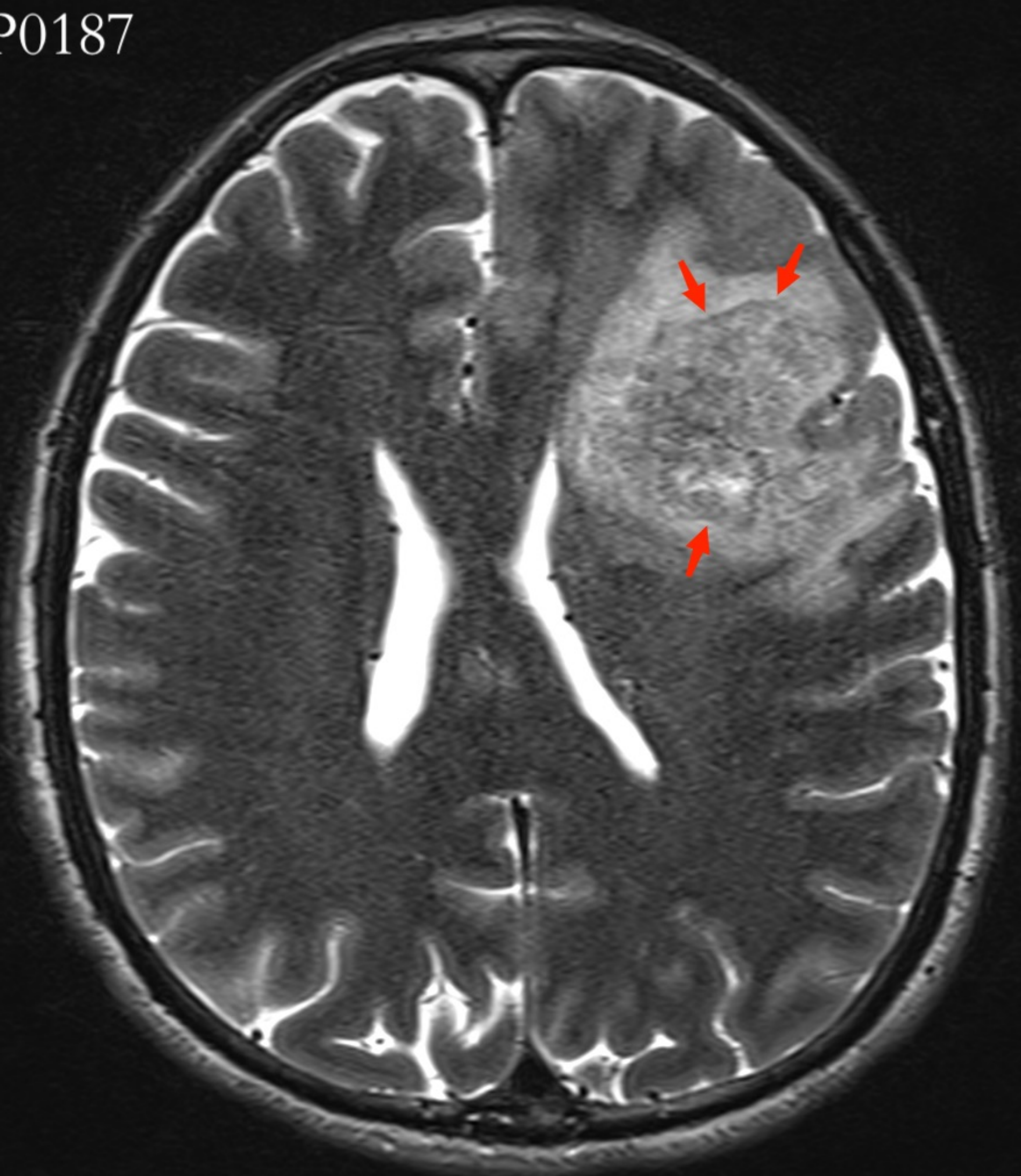

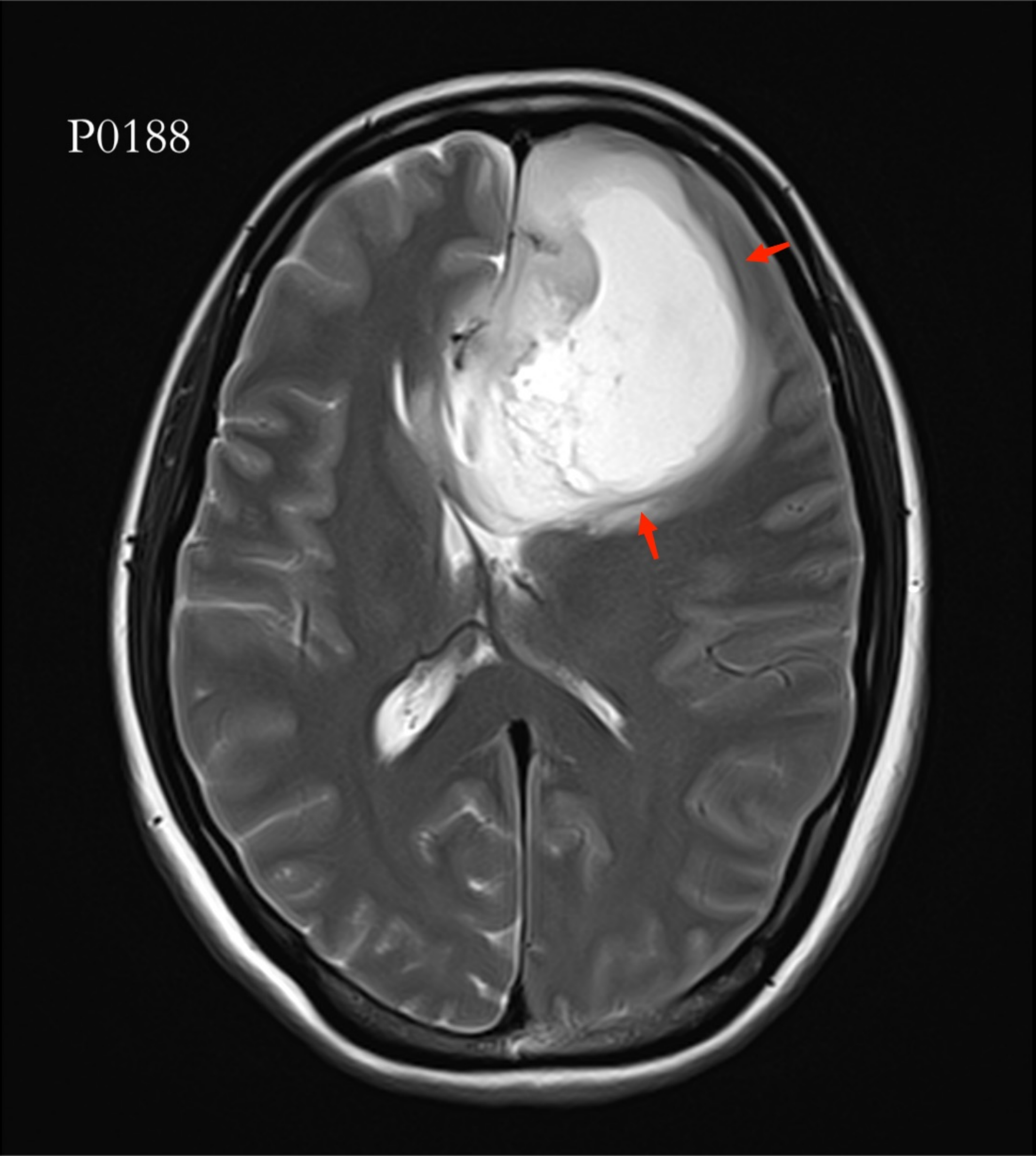
P0188

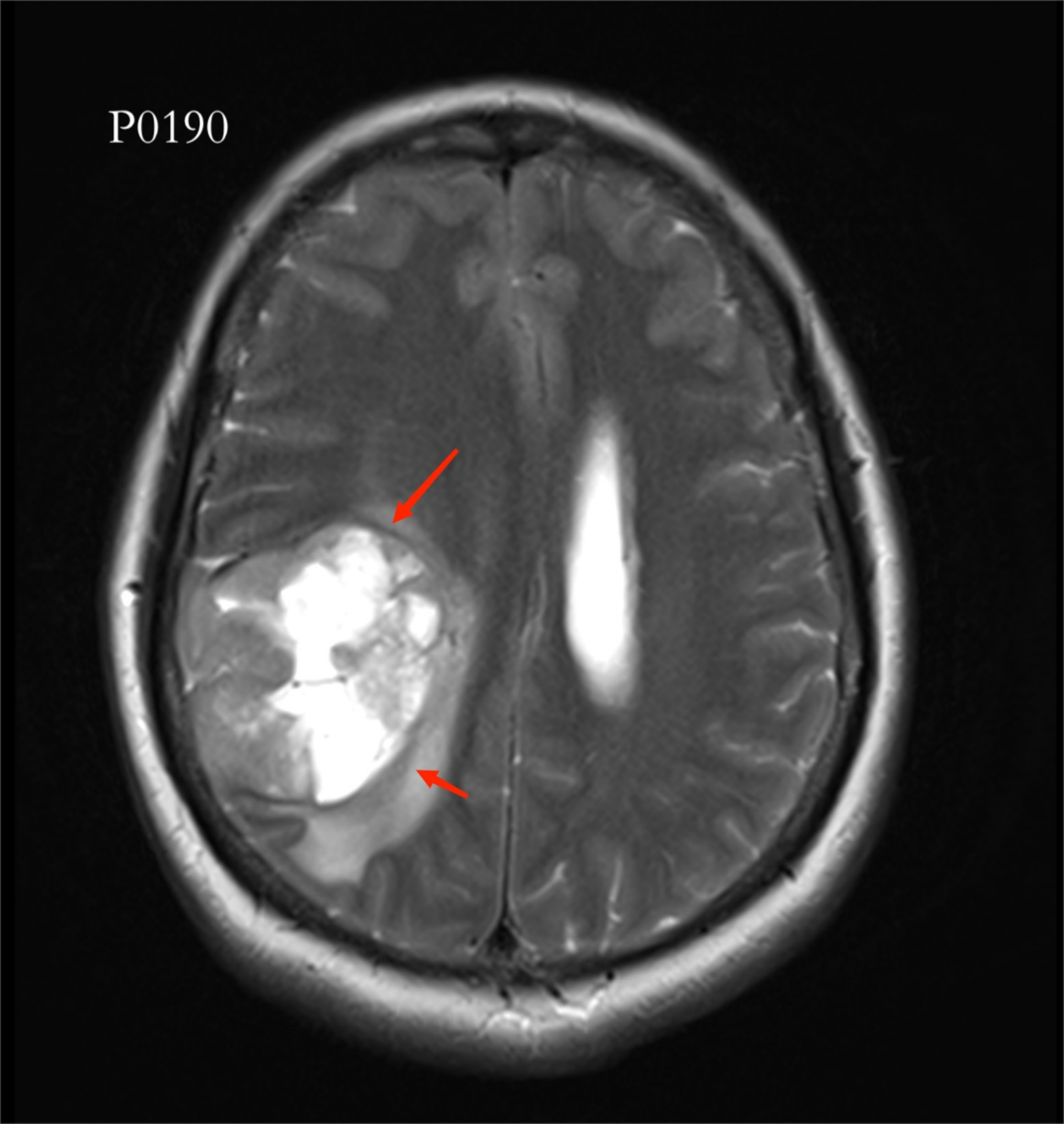
P0190

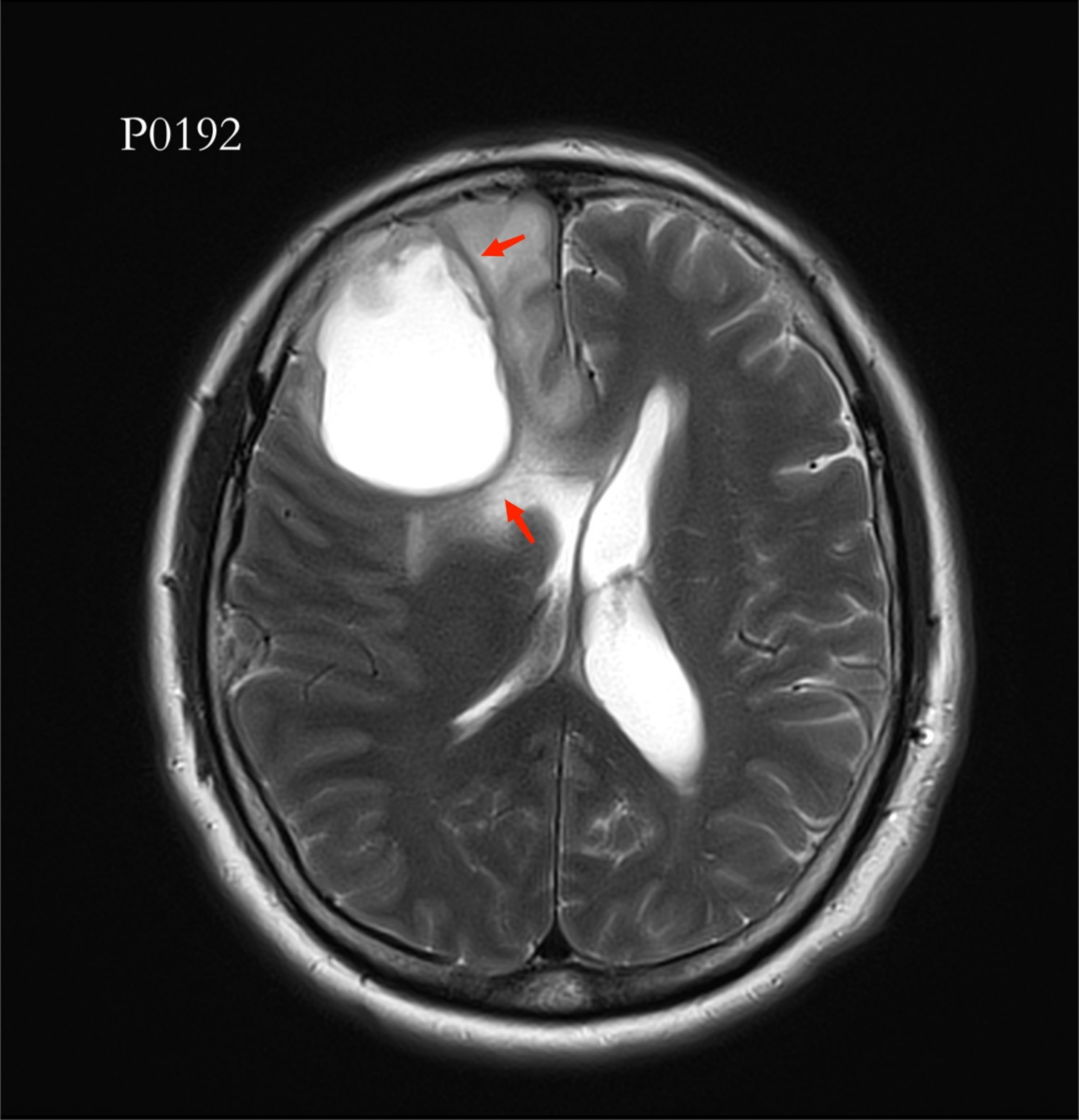
P0192

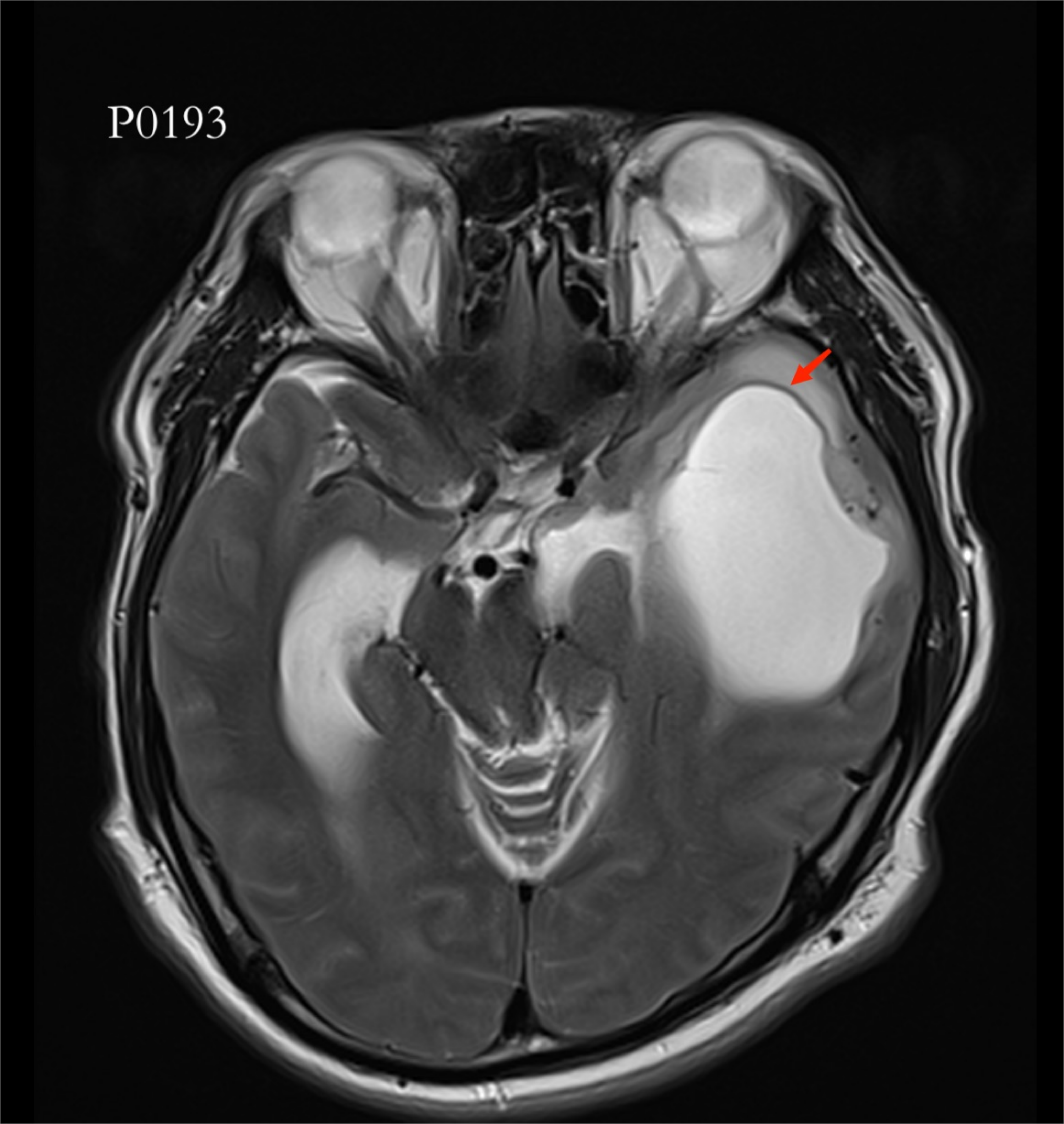
P0193

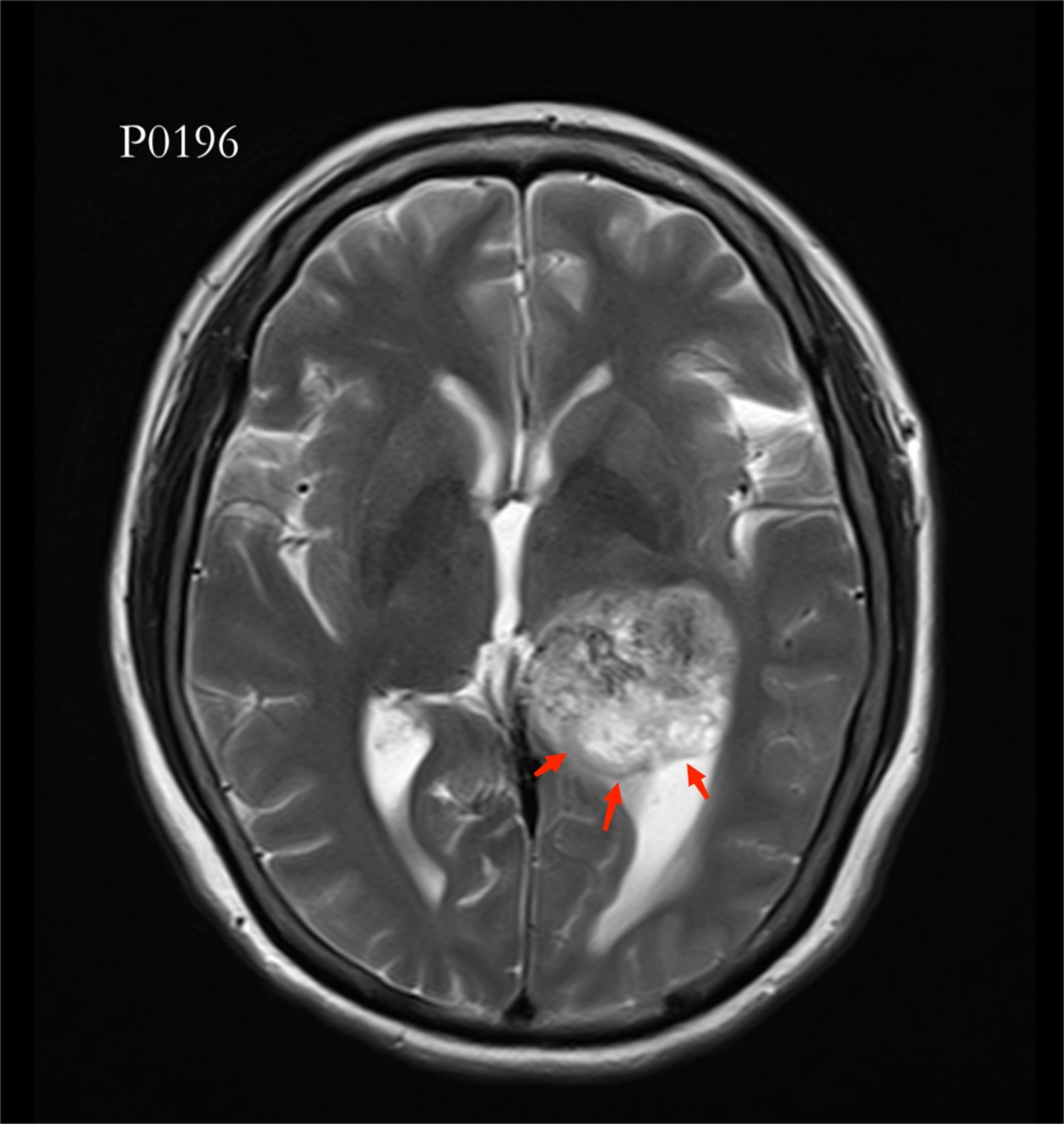
P0196

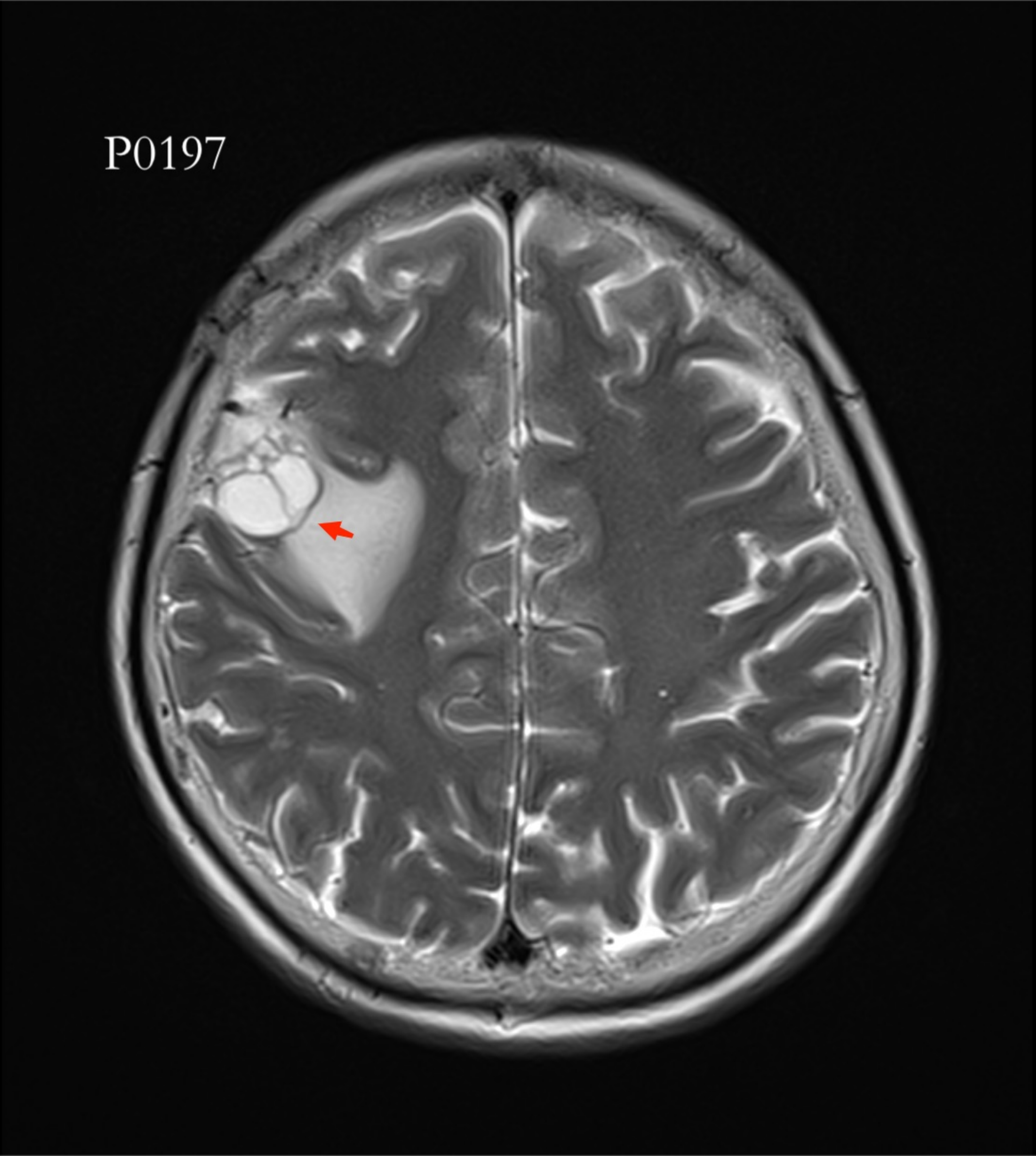
P0197

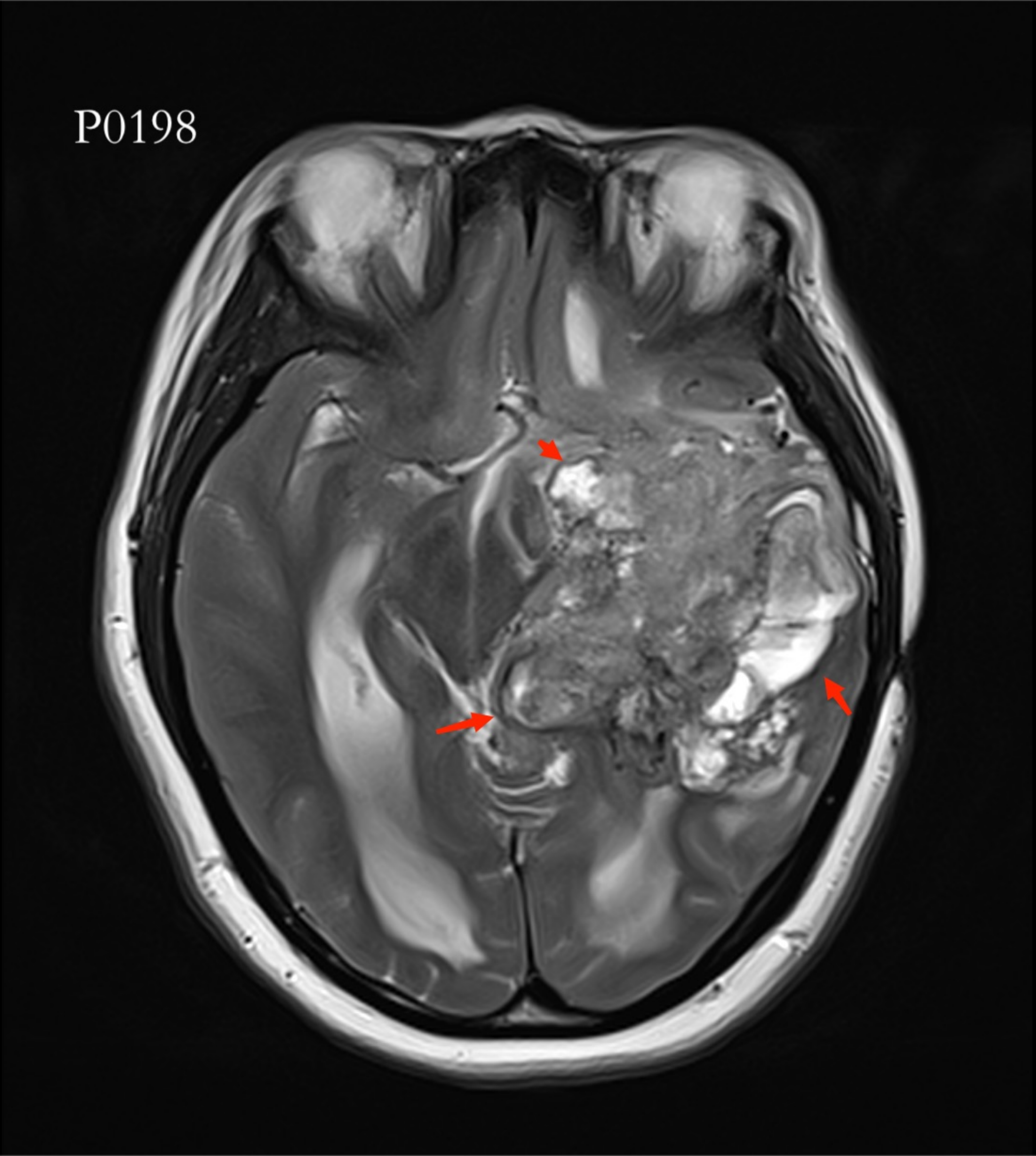
P0198

P0201

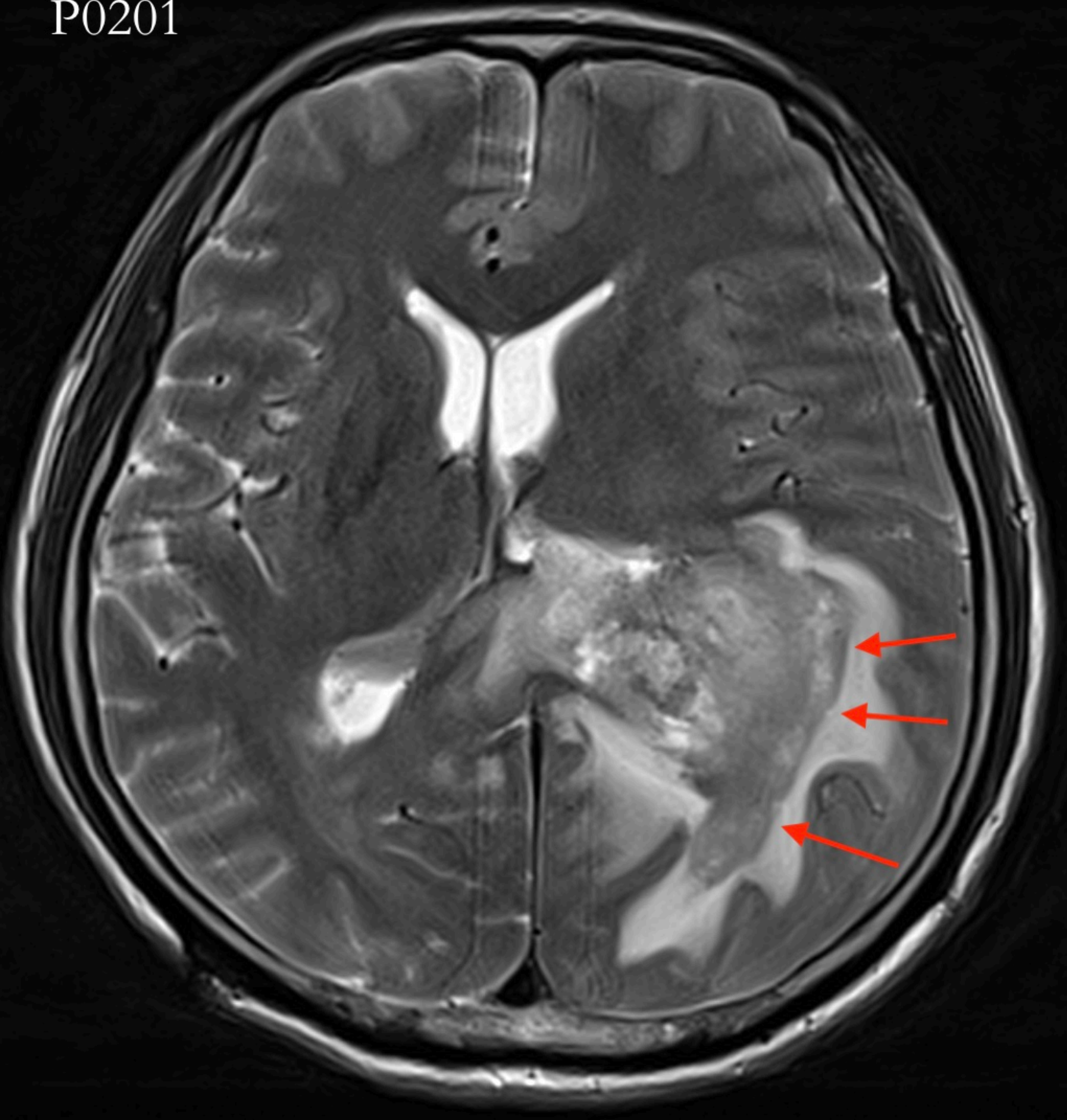

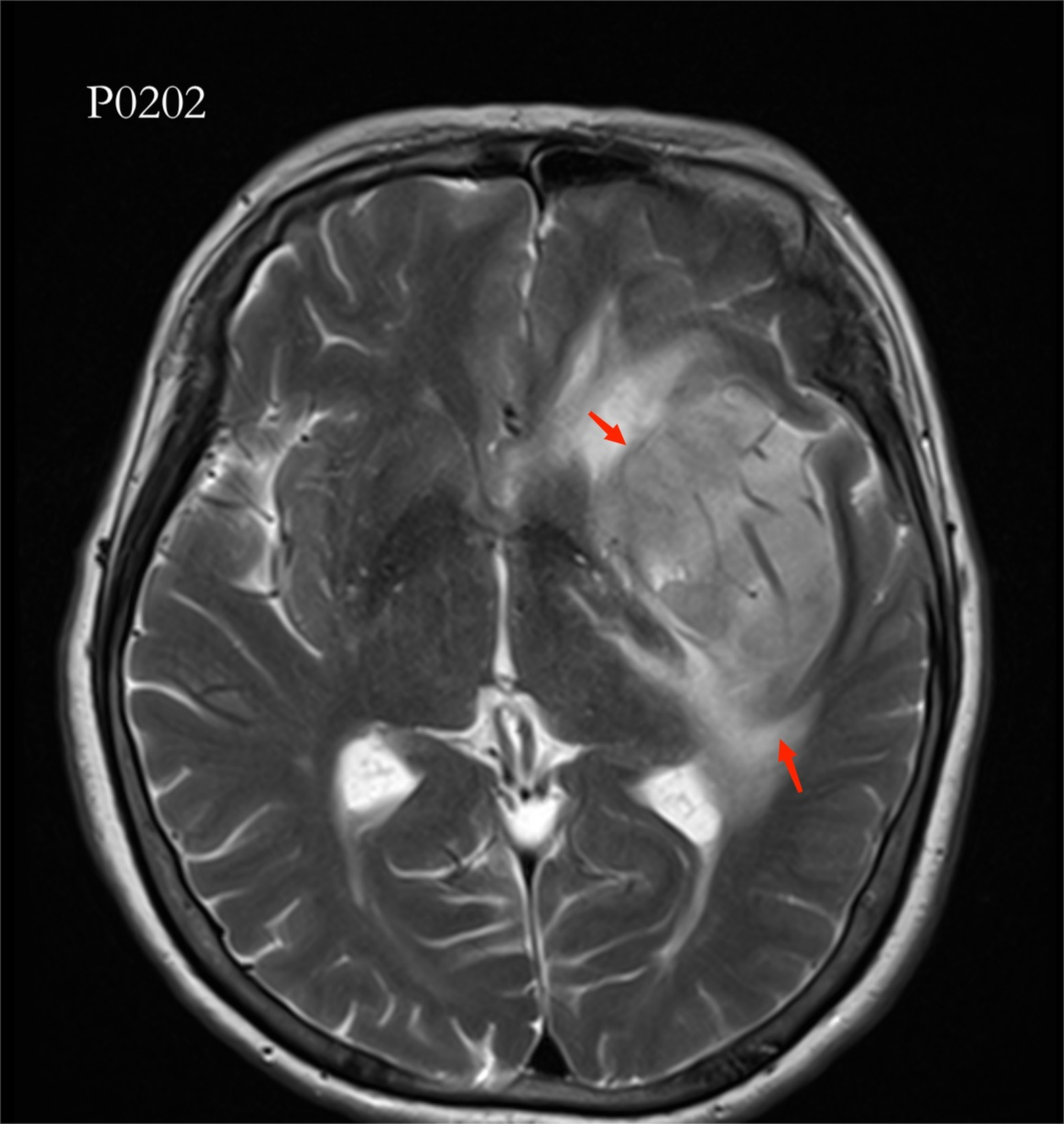
P0202

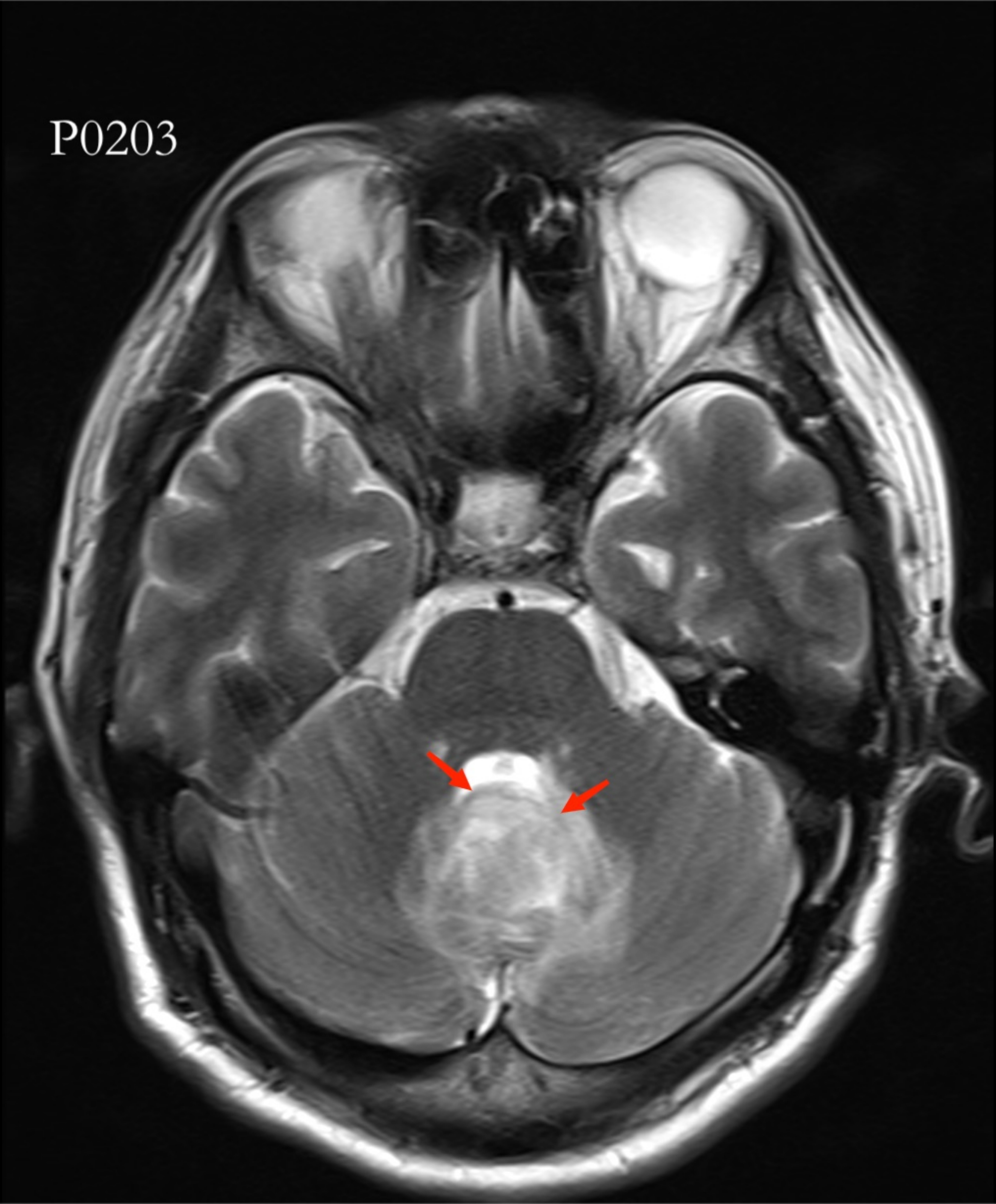
P0203

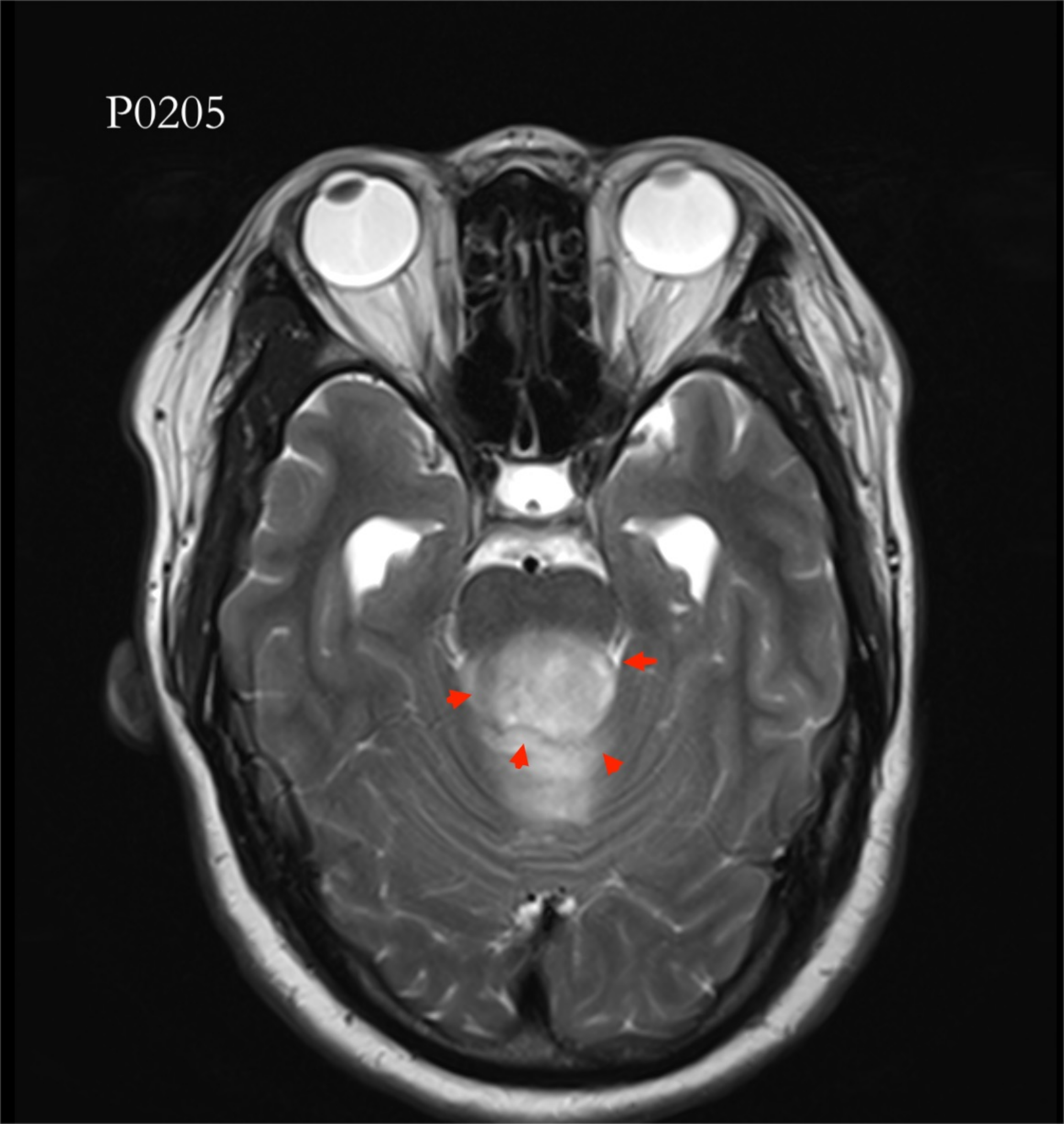
P0205

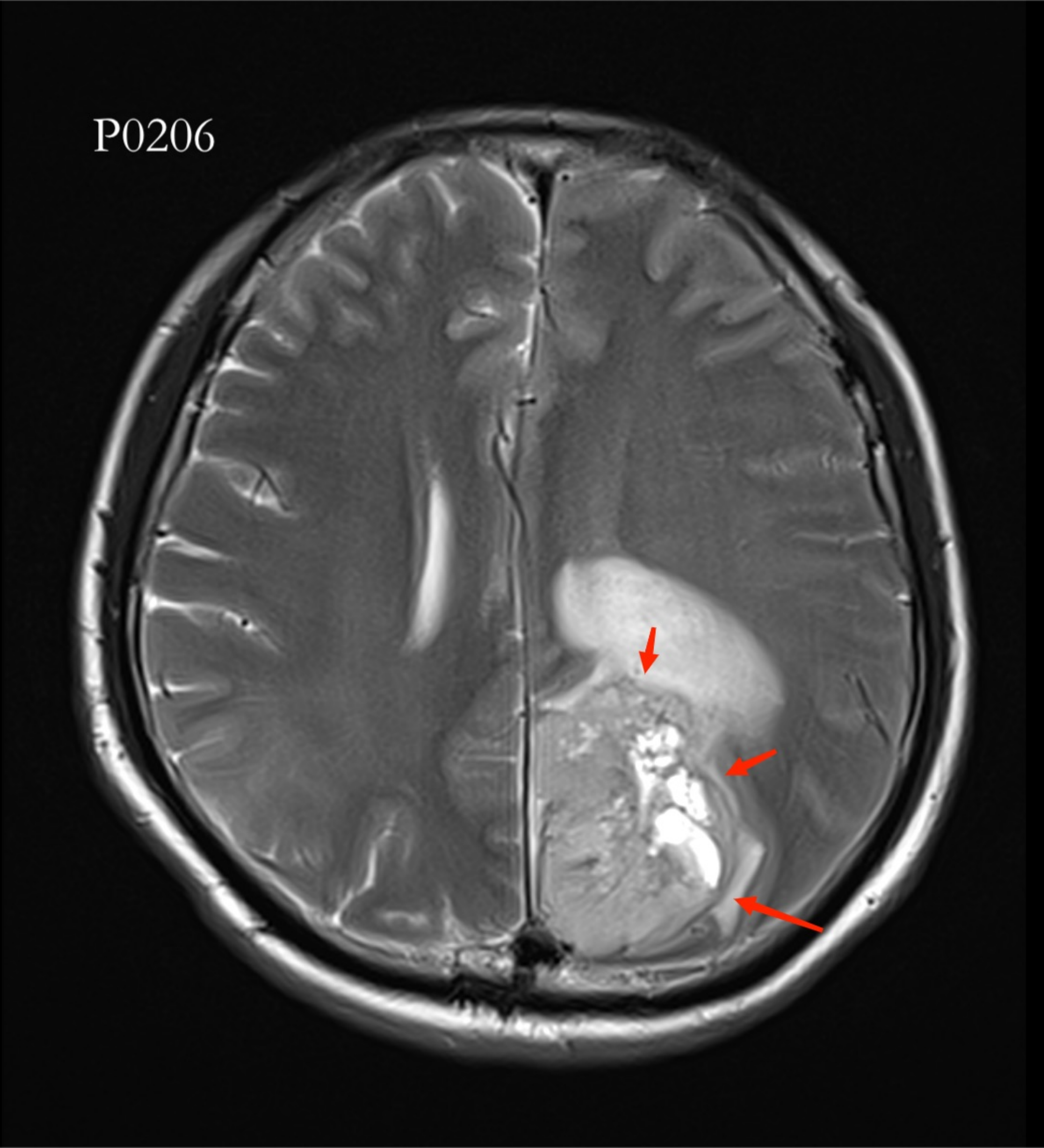
P0206

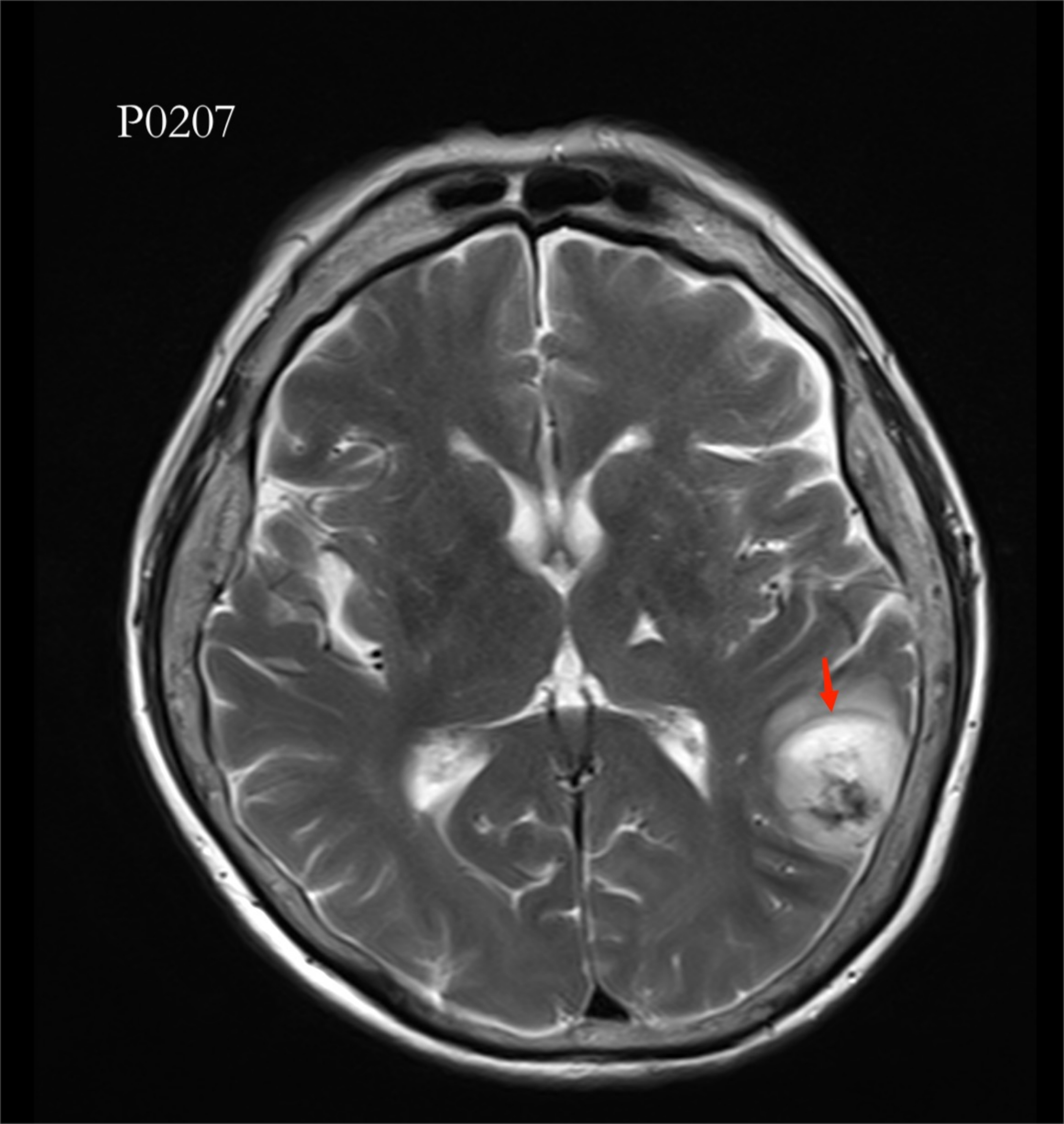
P0207
