# Supplementary figures and images for: Identification of T2W hypointense ring as a novel noninvasive indicator for glioma grade and IDH genotype
Source: Cancer Imaging. 2024 Jun 28;24:80. doi: 10.1186/s40644-024-00726-3 (PMC11212435; doi:10.1186/s40644-024-00726-3)

P0001

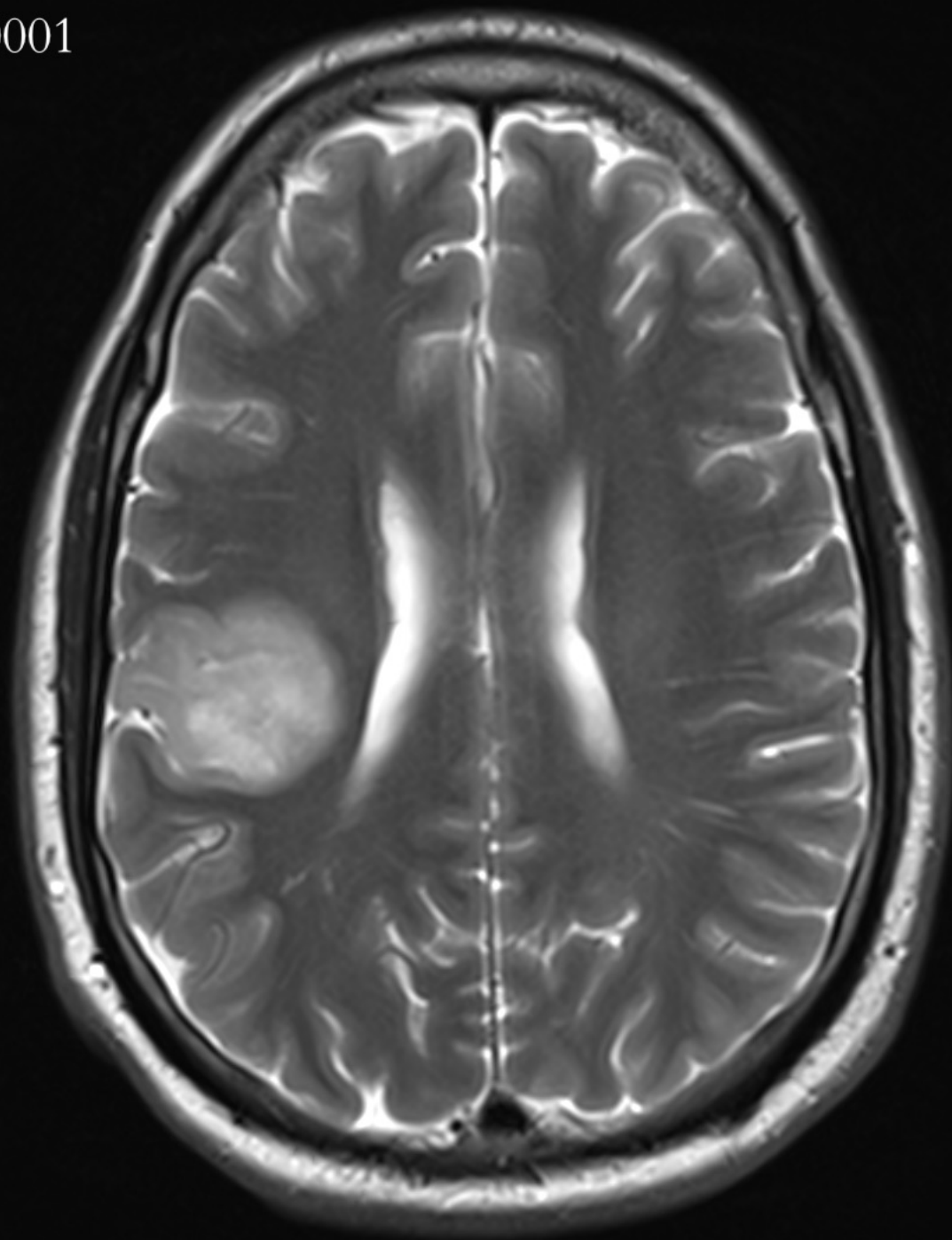

P0005

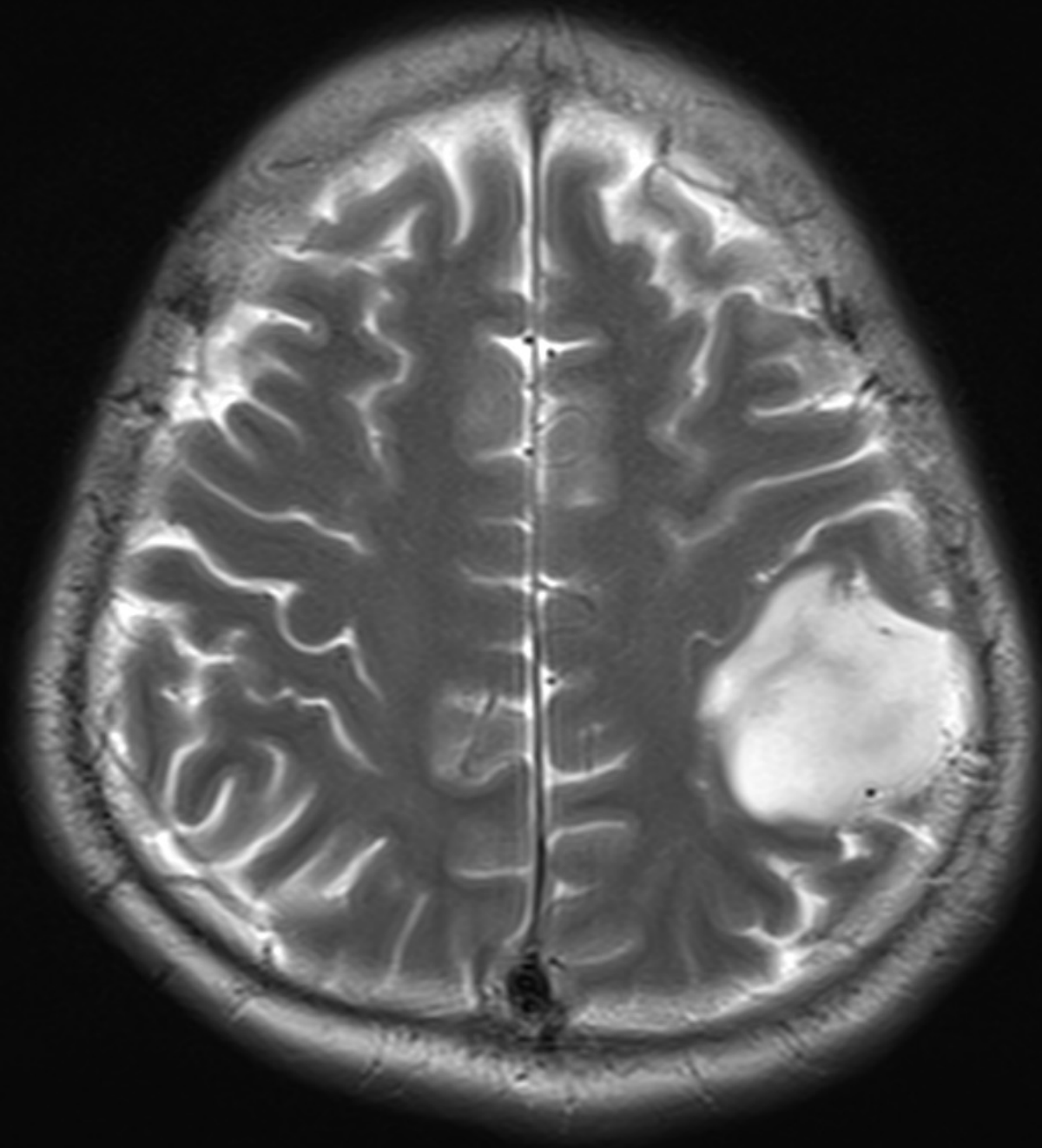

P0008

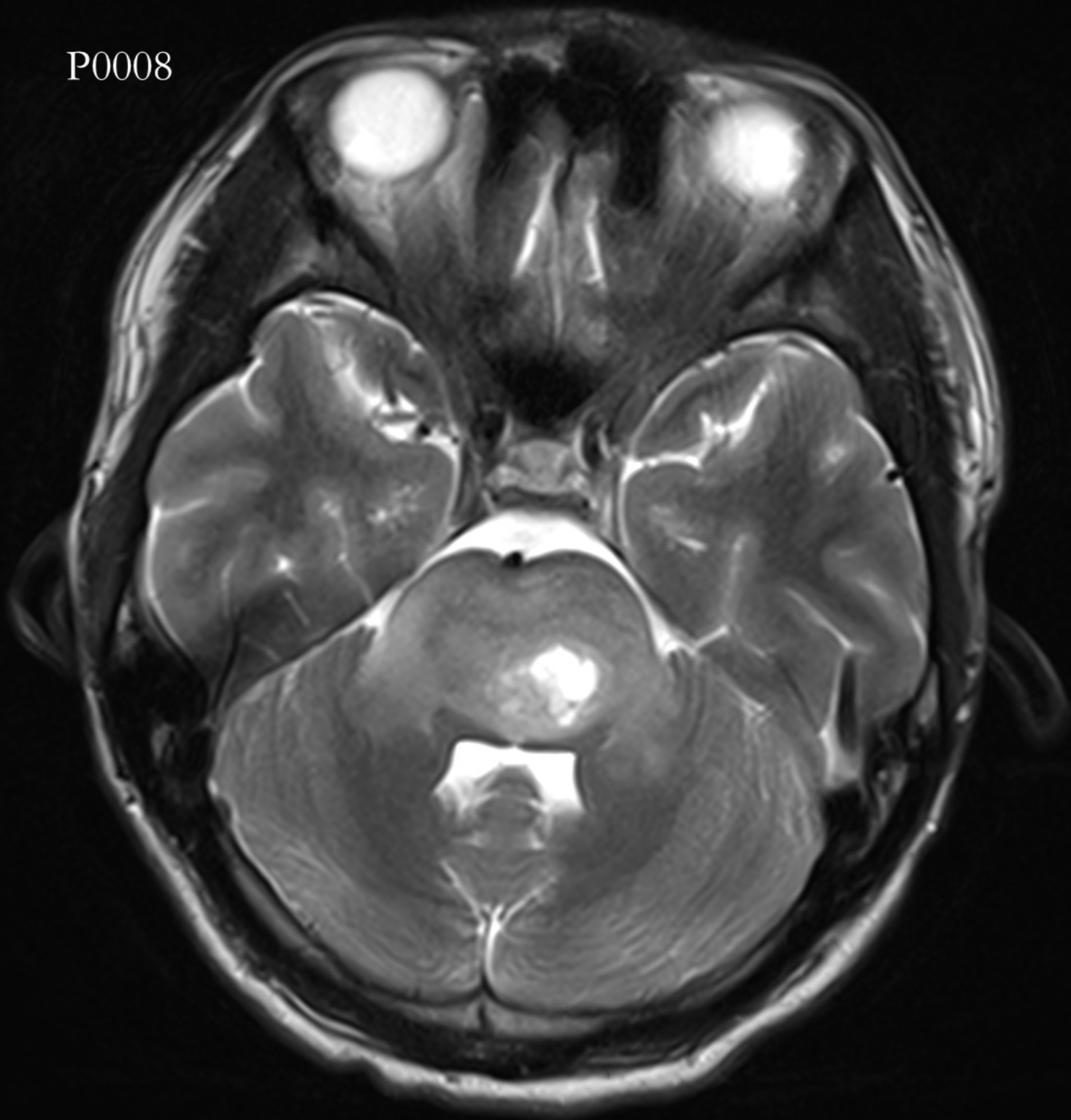

P0011

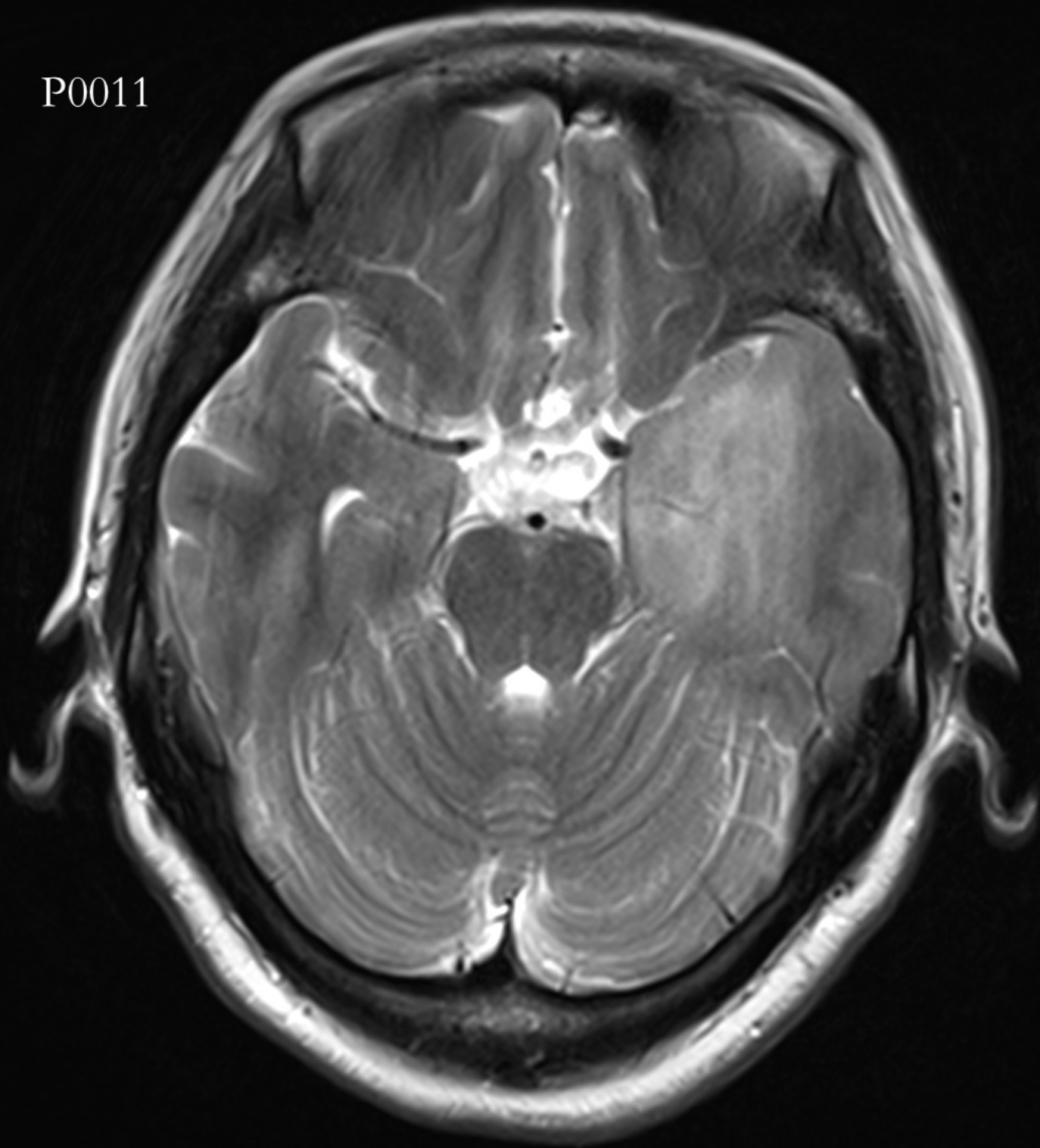

P0013

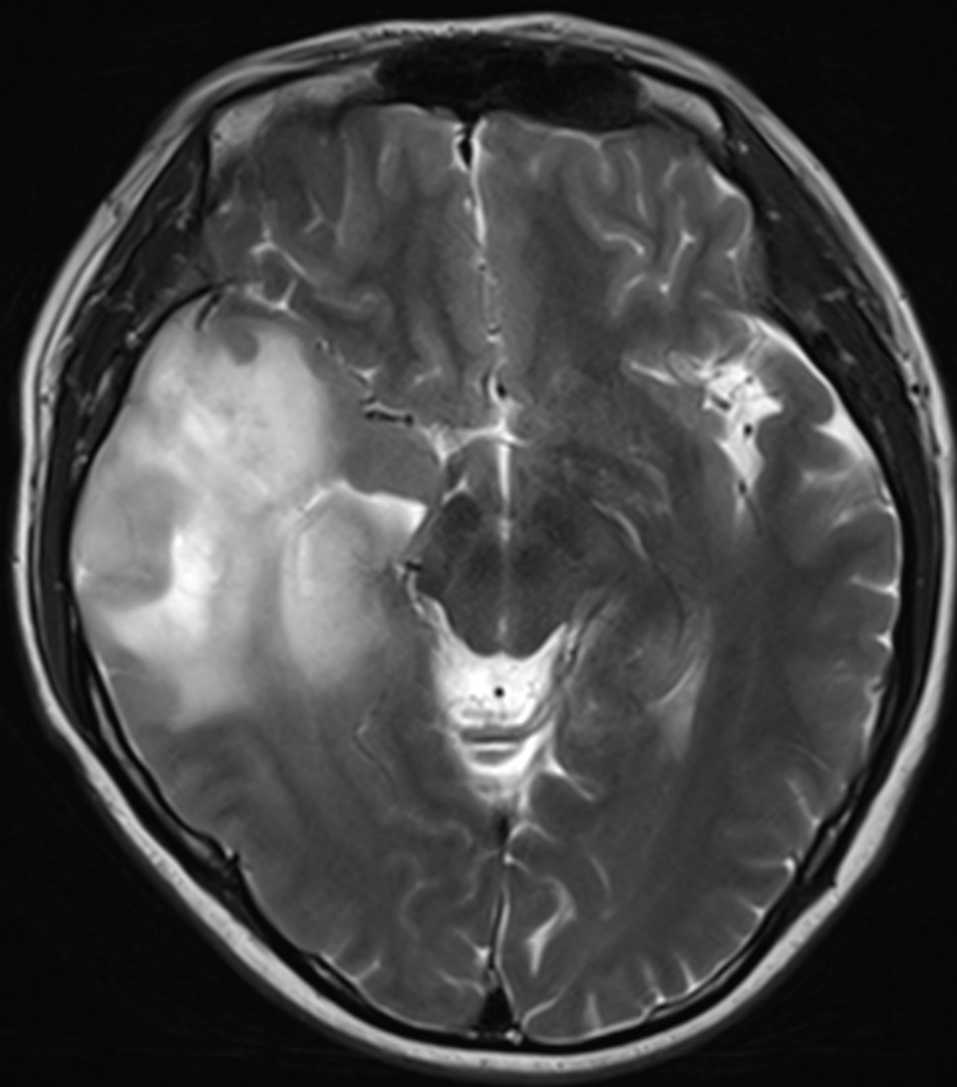

P0017

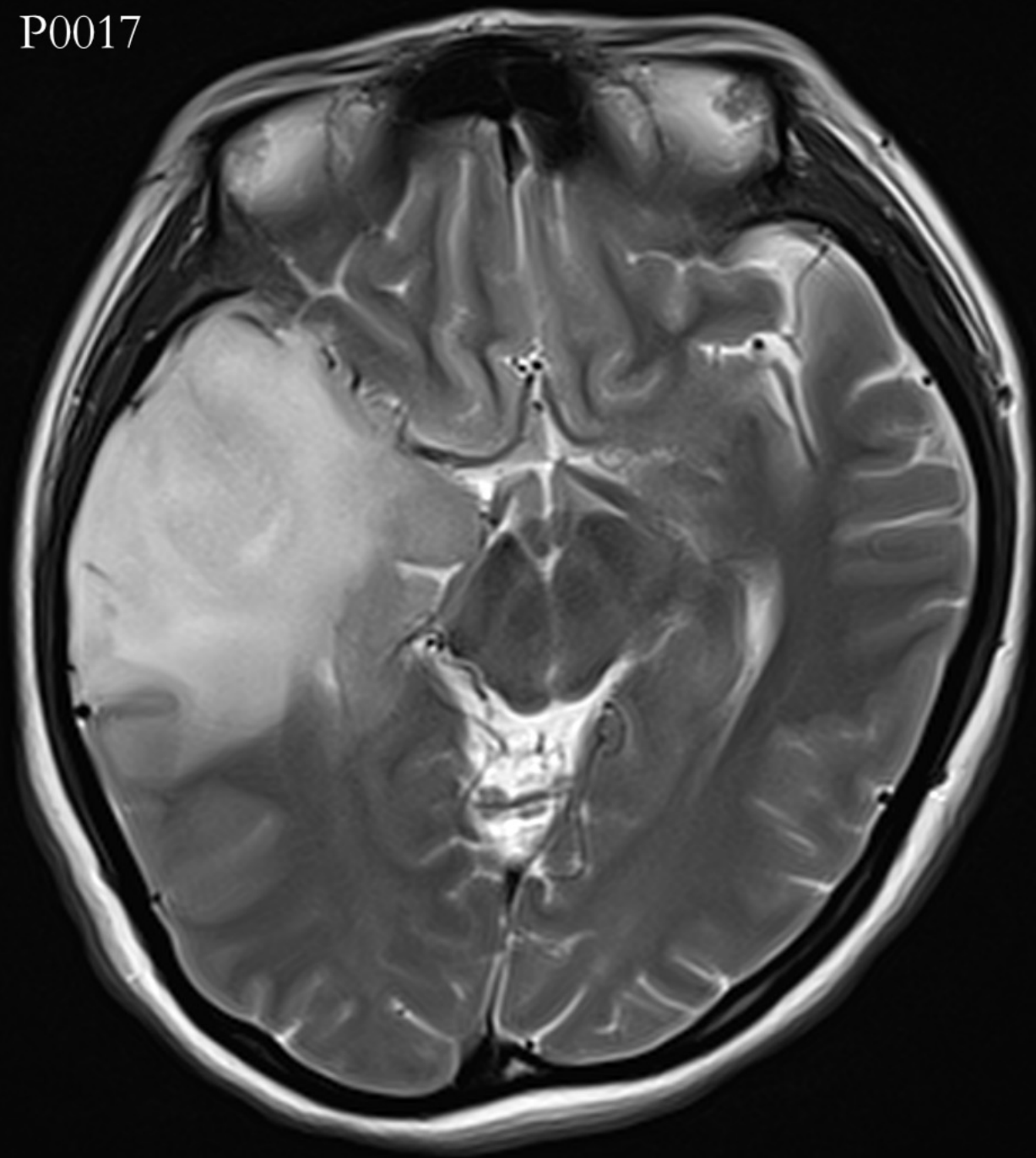

P0023

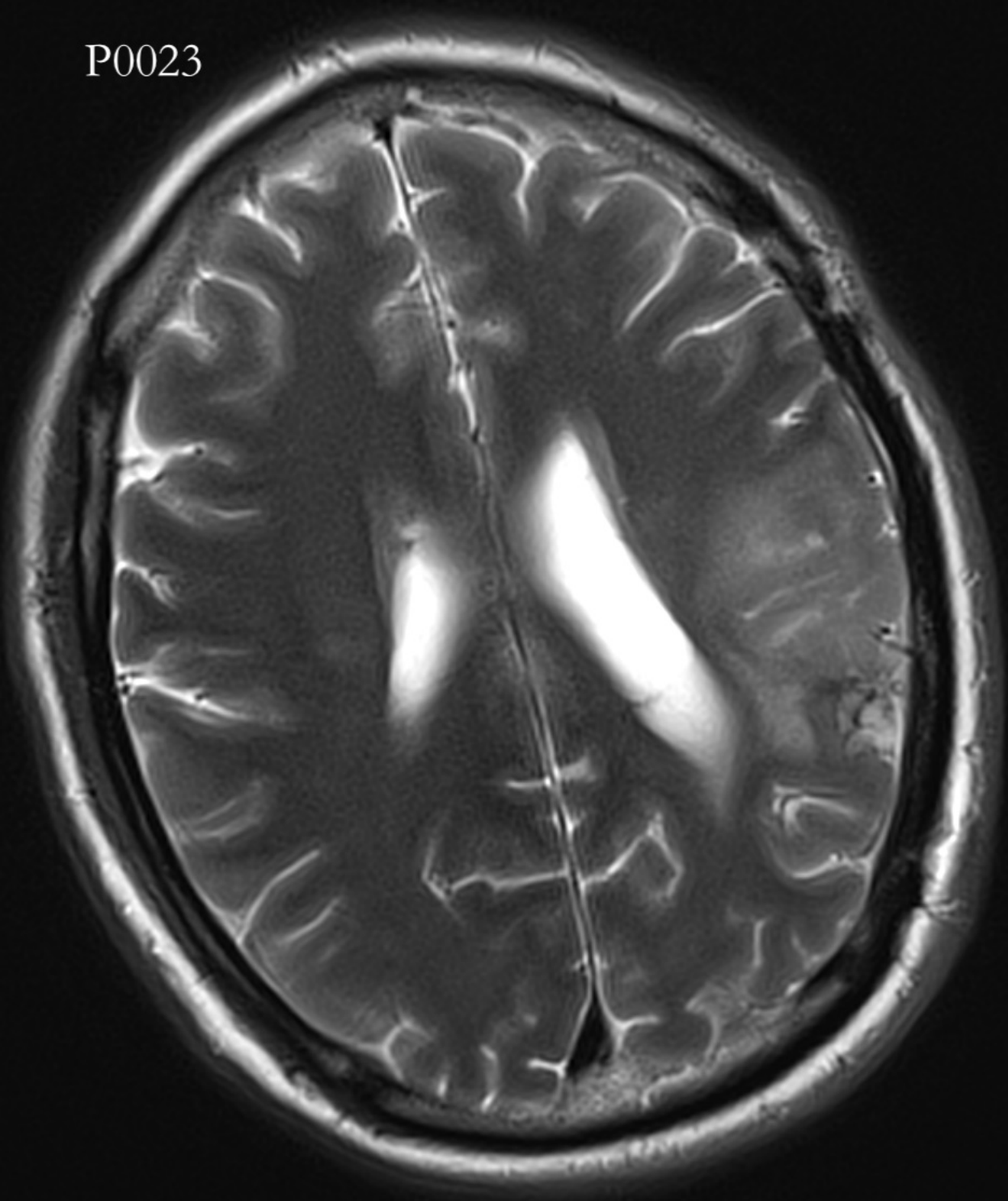

P0025

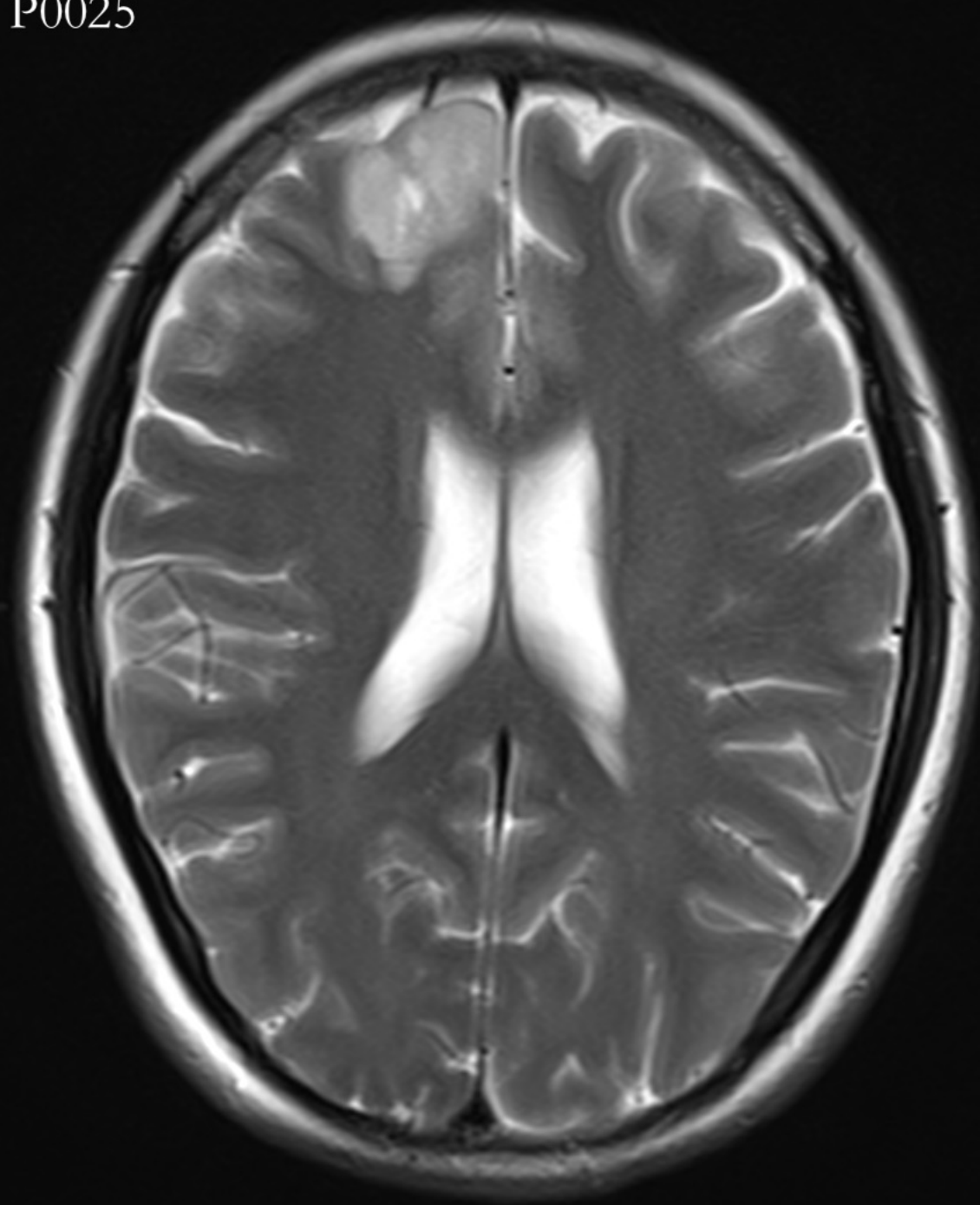

P0028

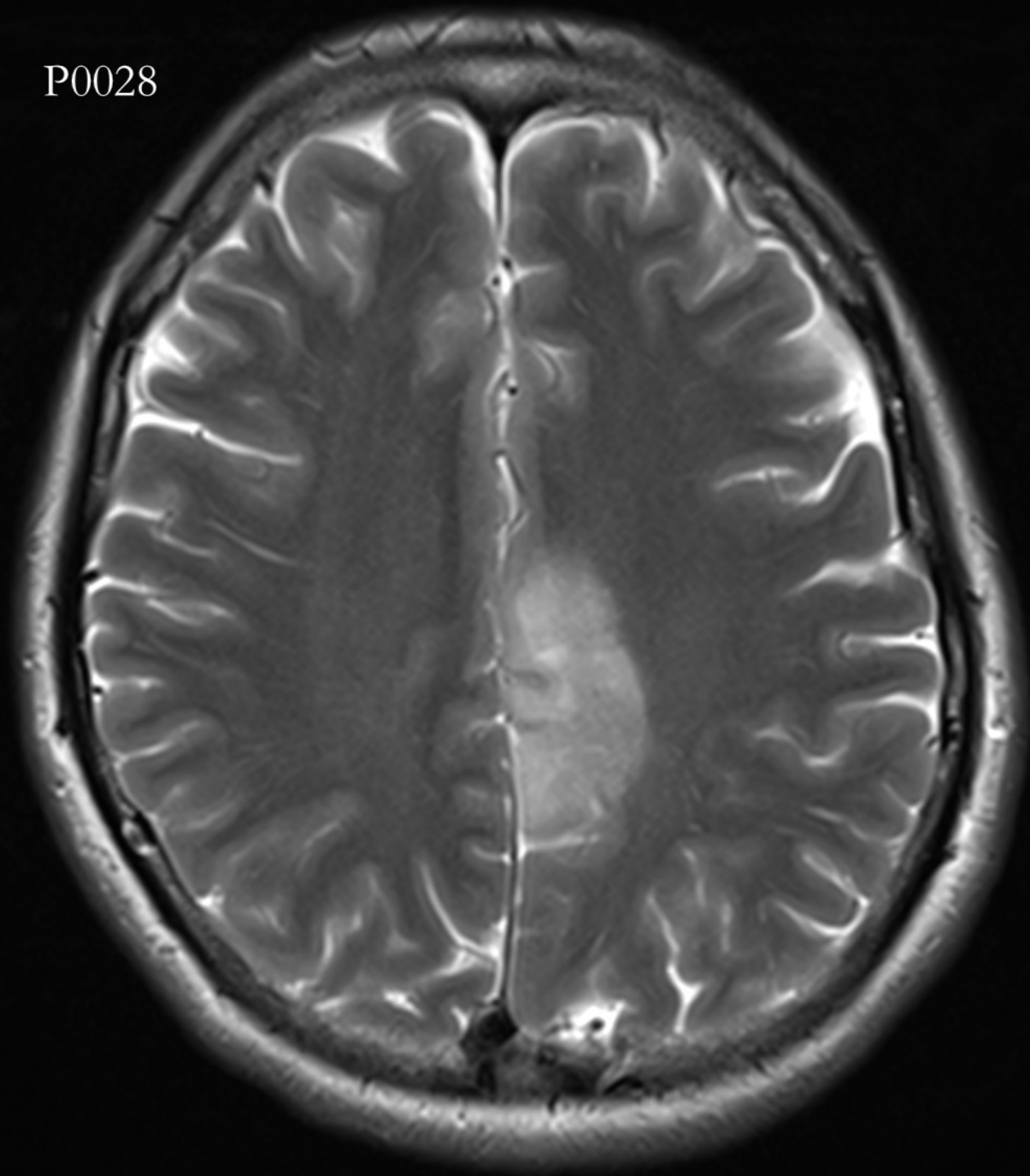

P0030

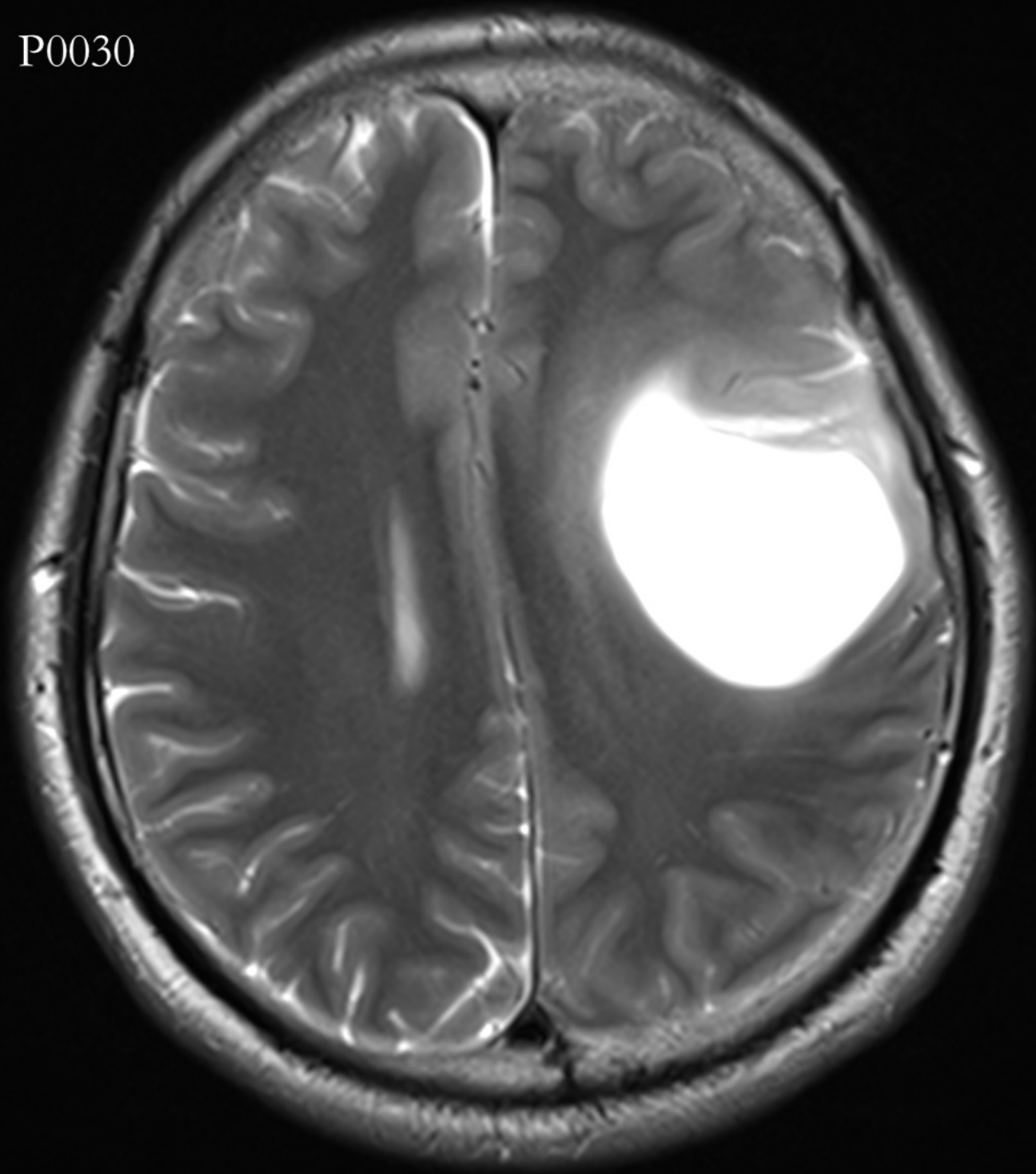

P0033

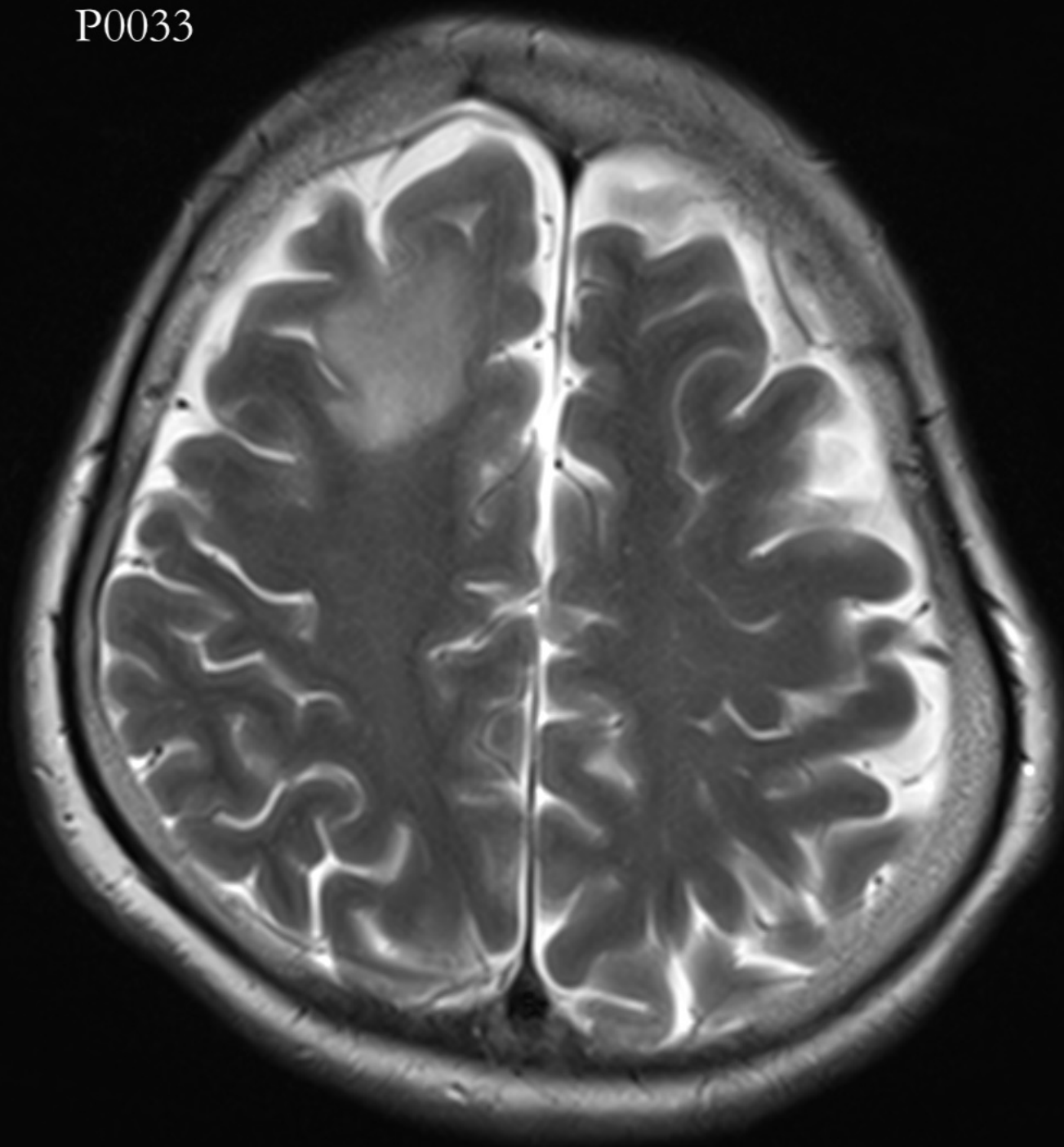

P0034

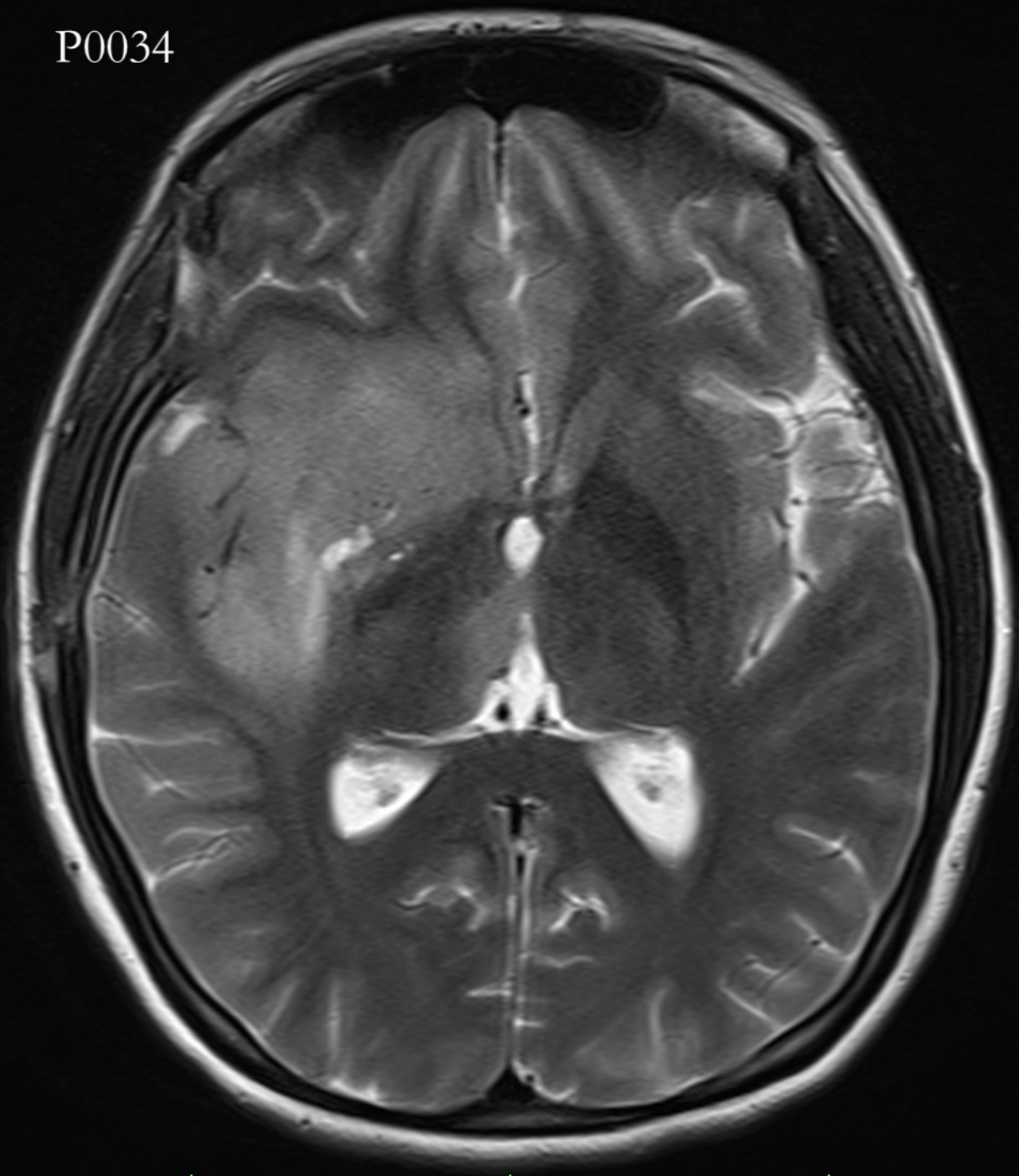

P0035

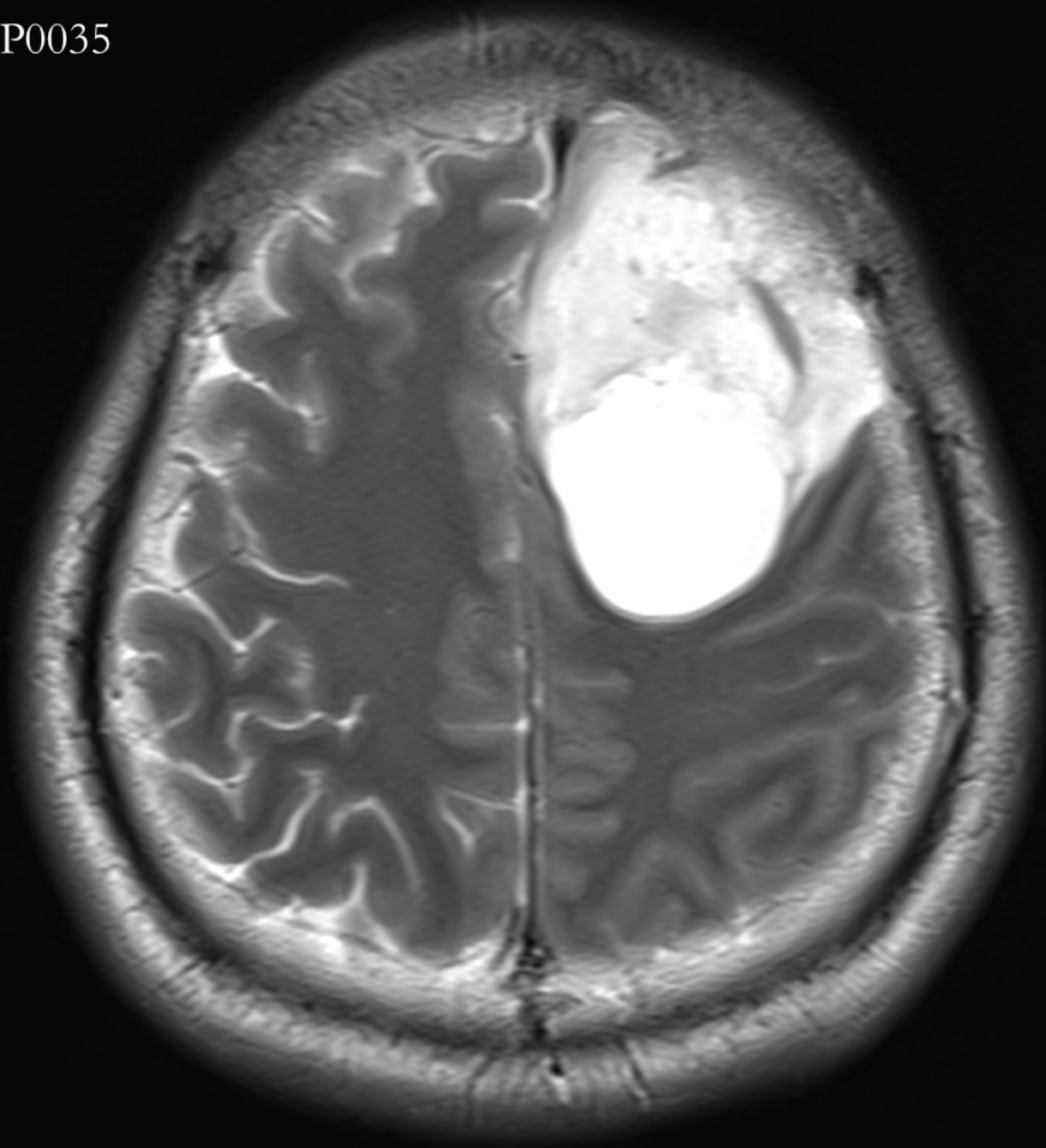

P0038

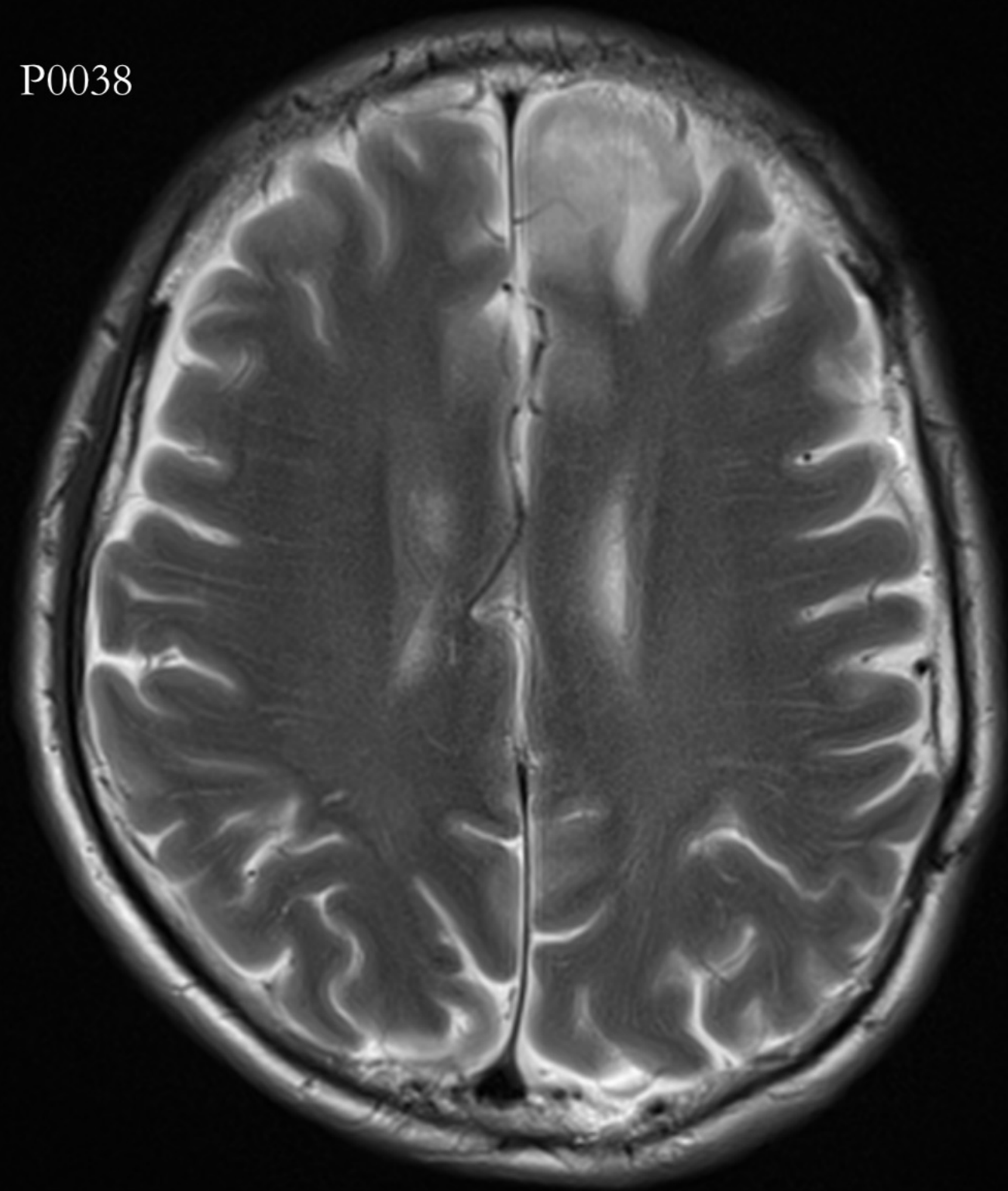

P0040

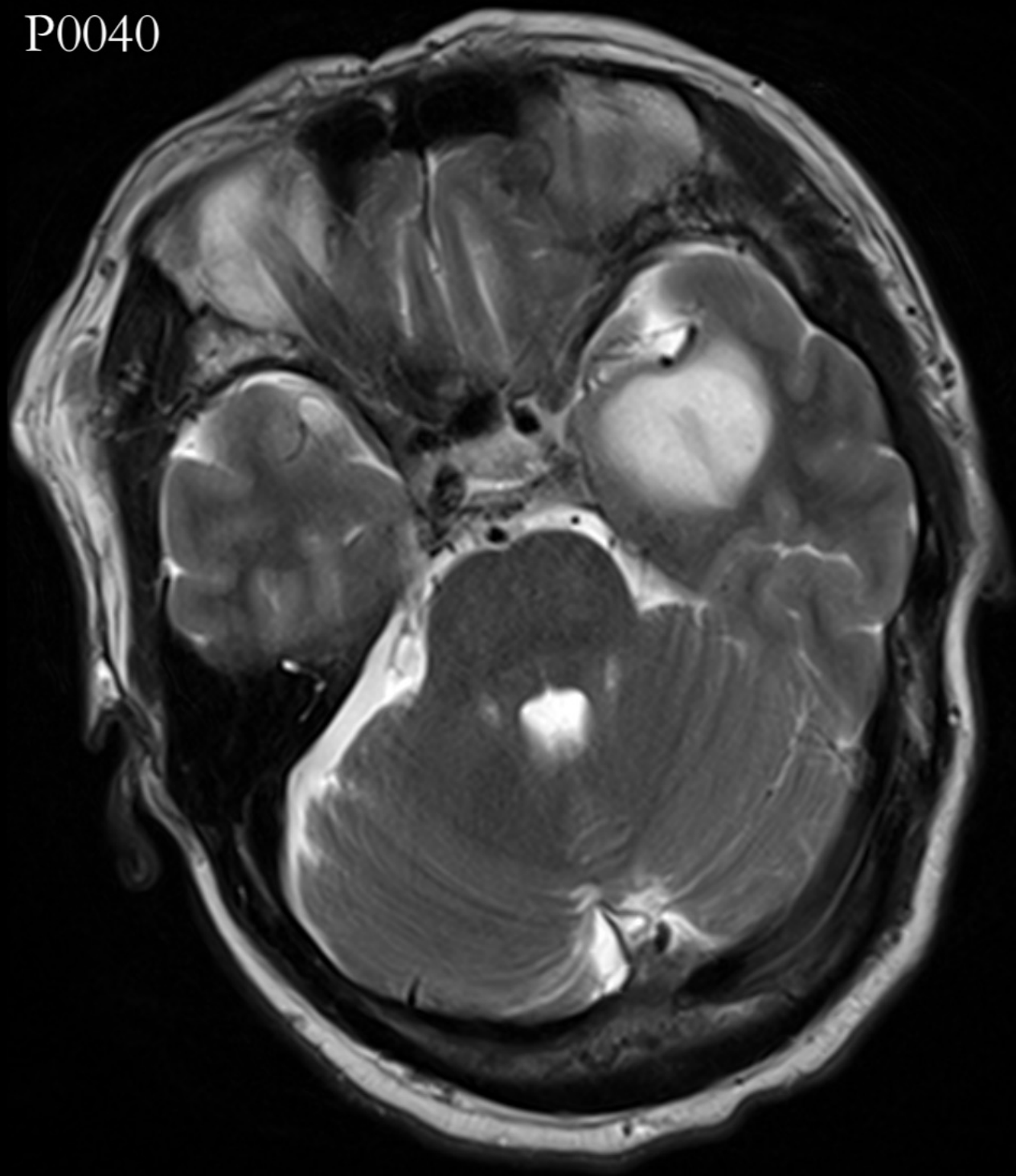

P0042

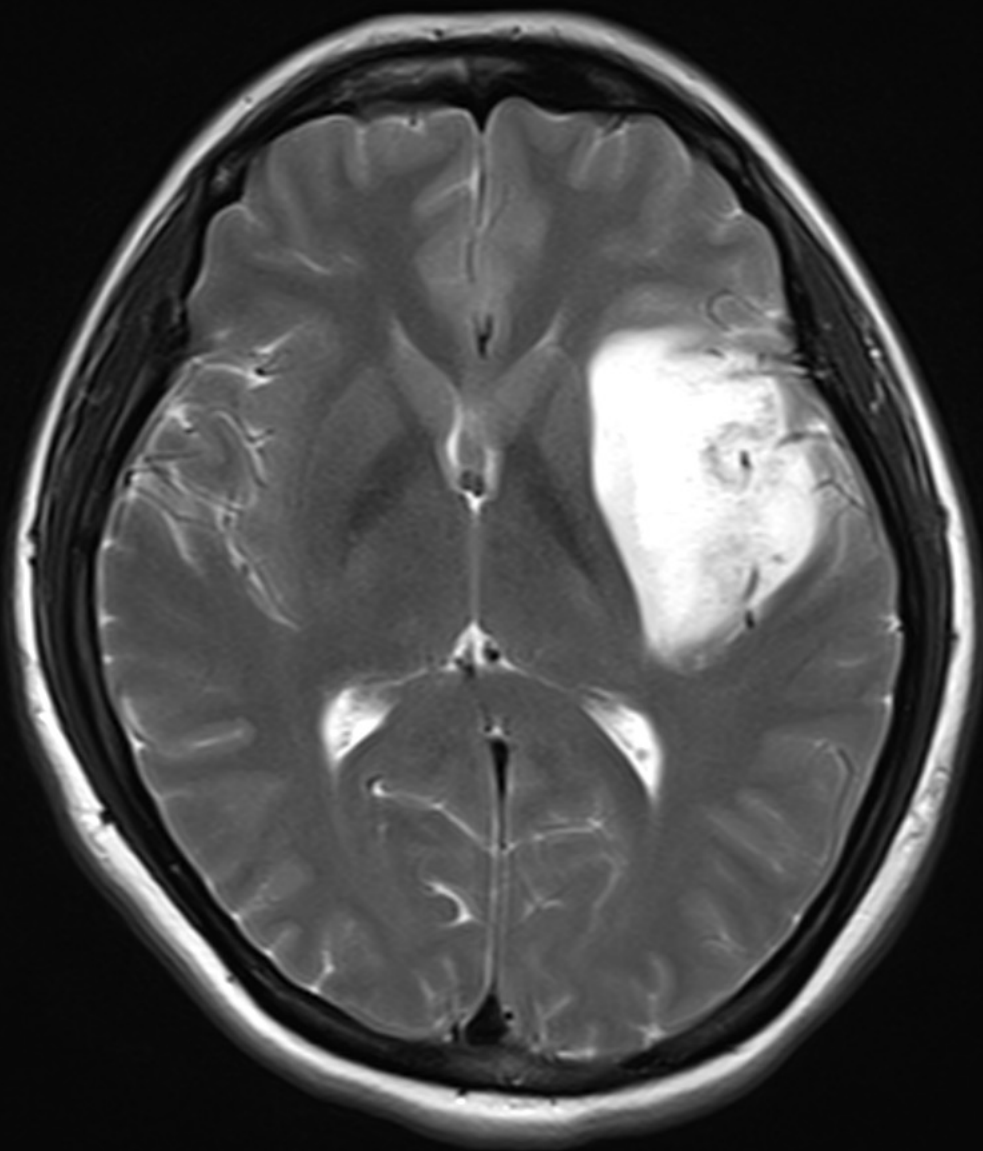

P0043

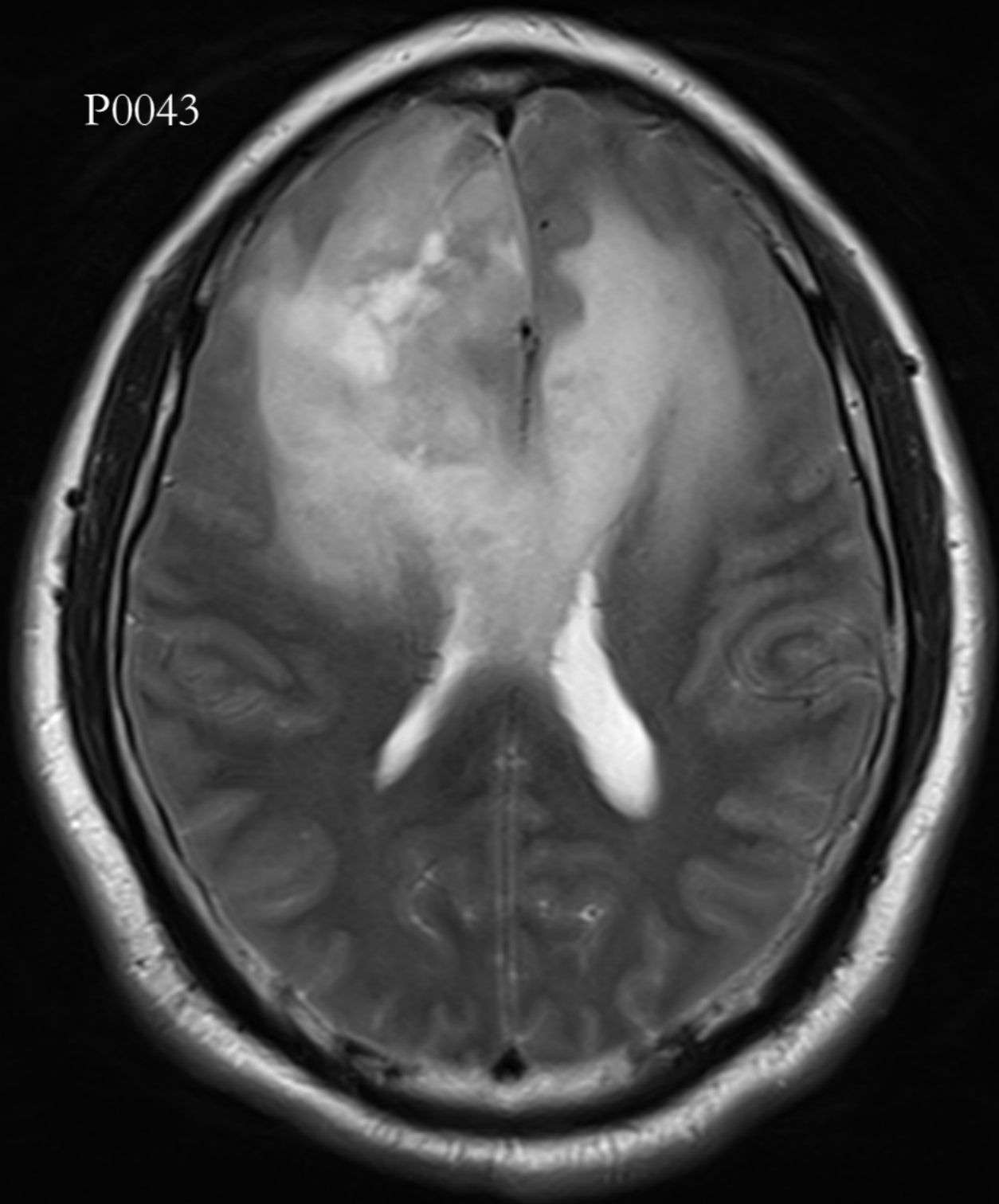

P0047

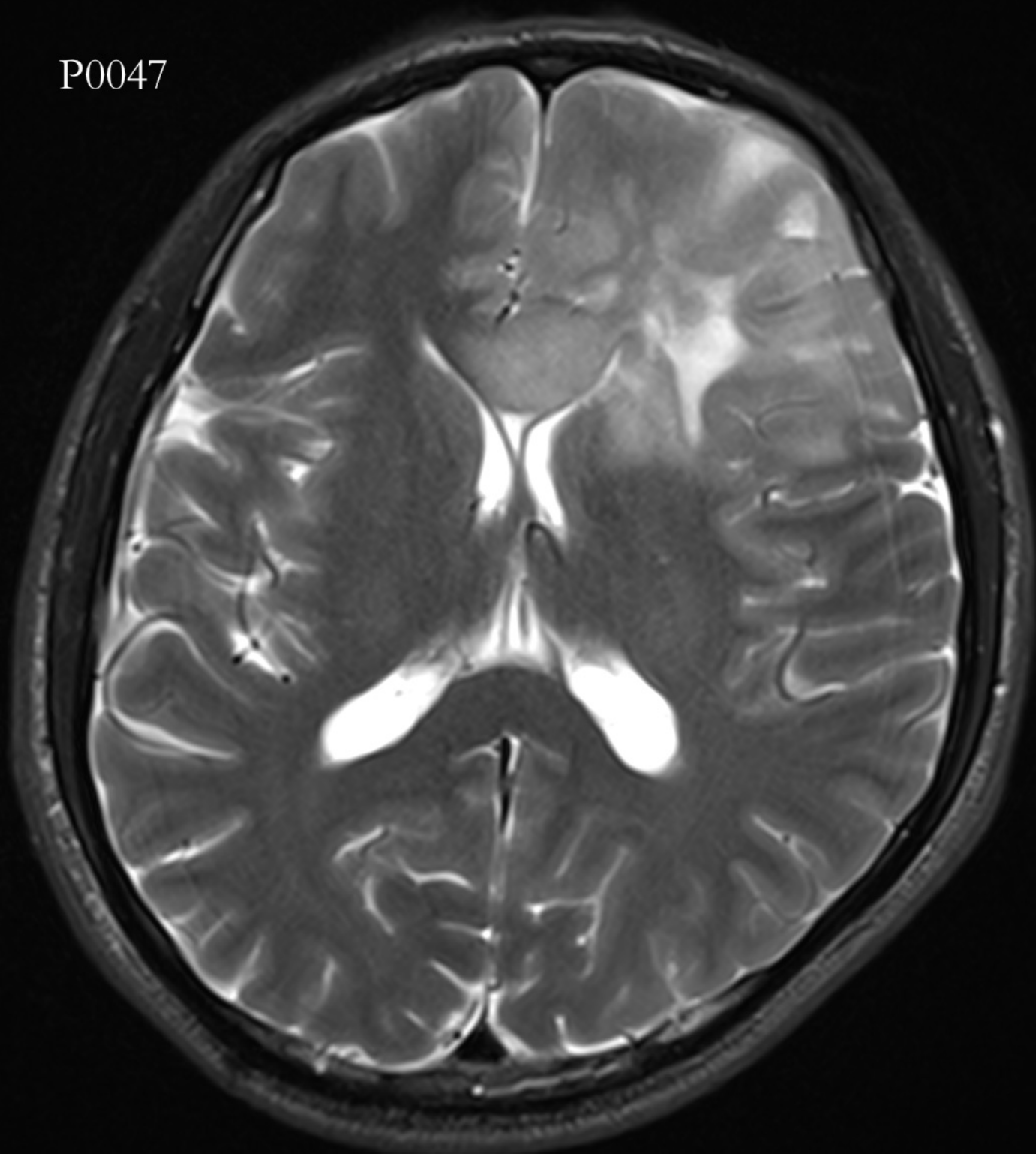

P0049

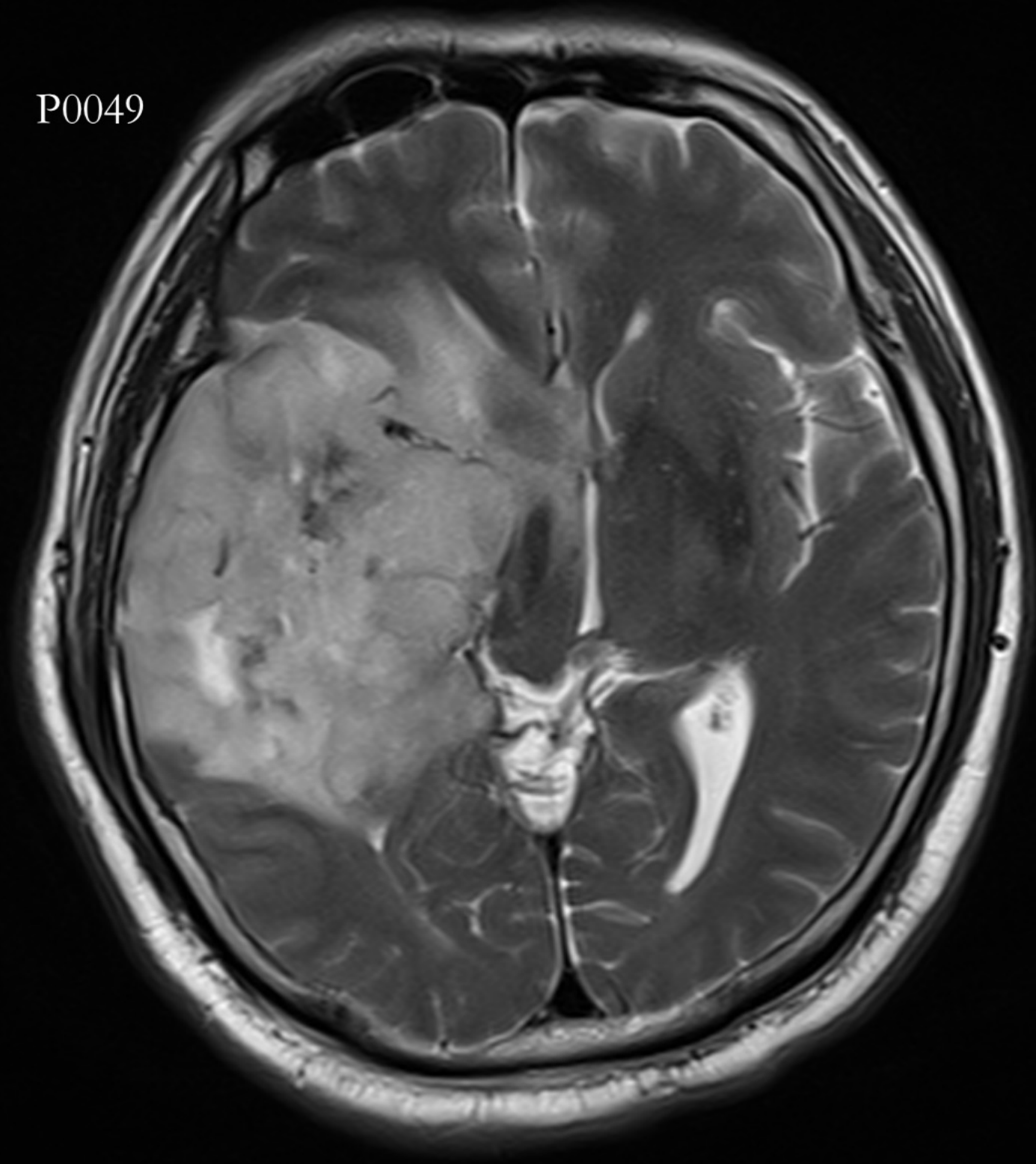

P0056

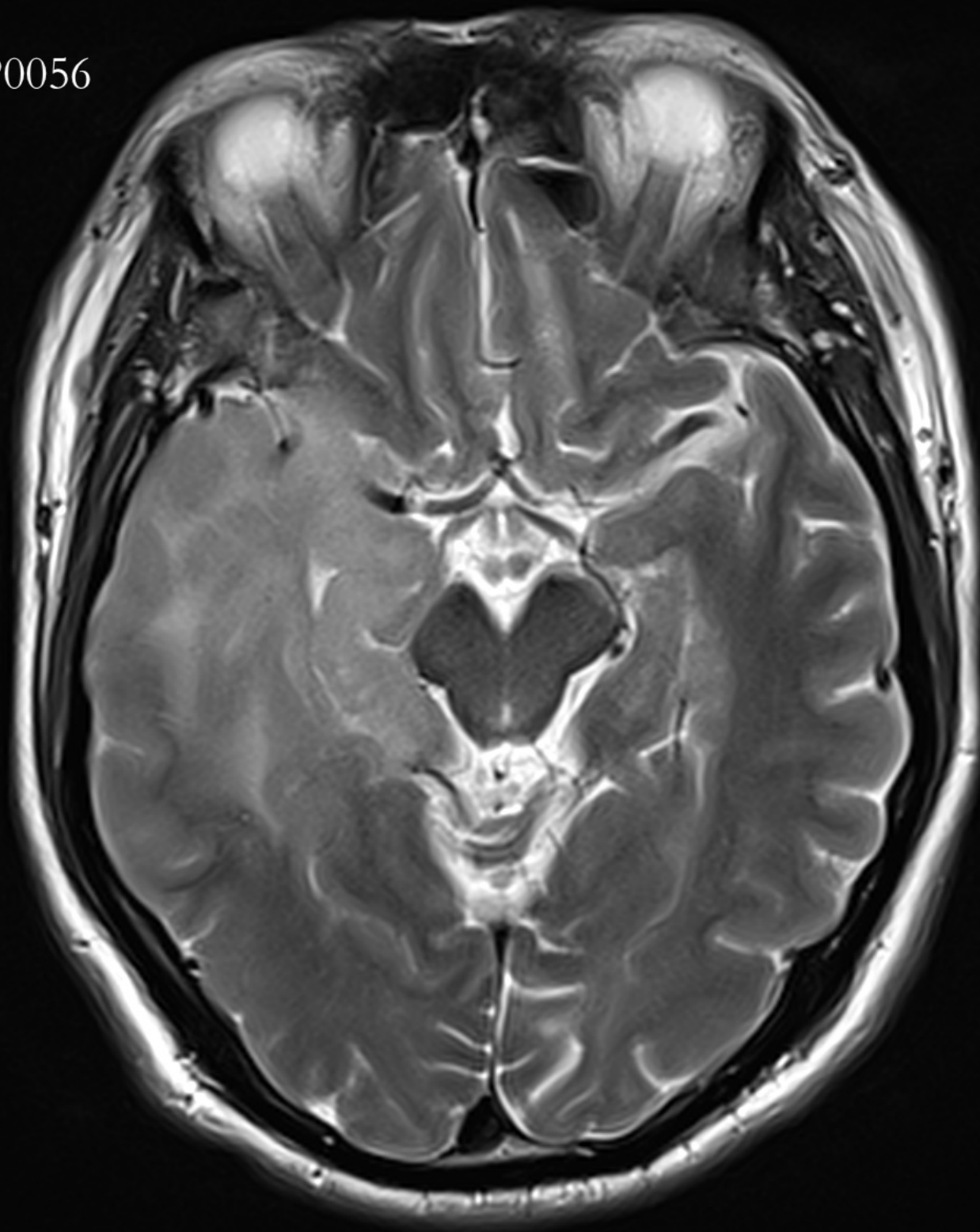

P0068

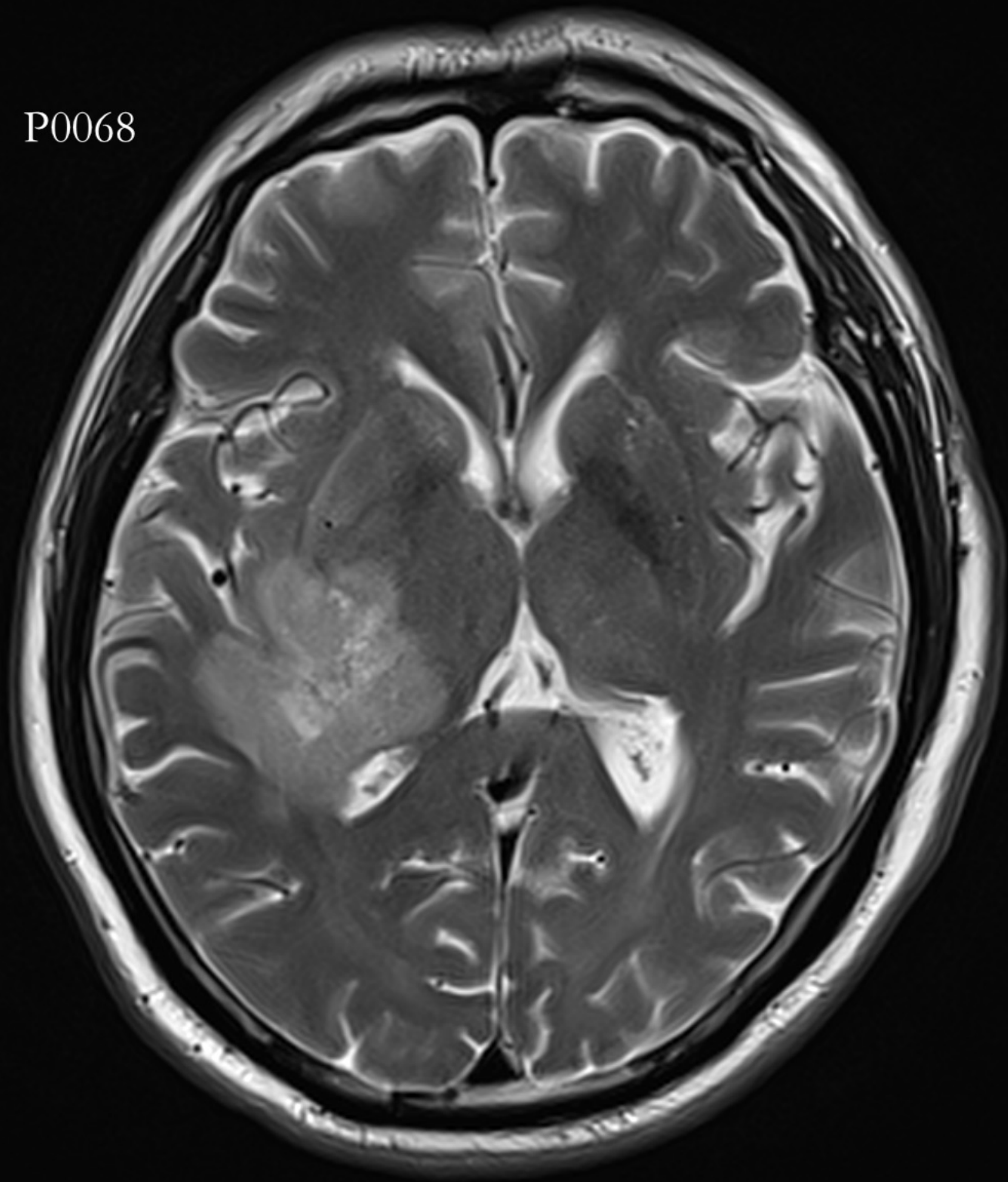

P0088

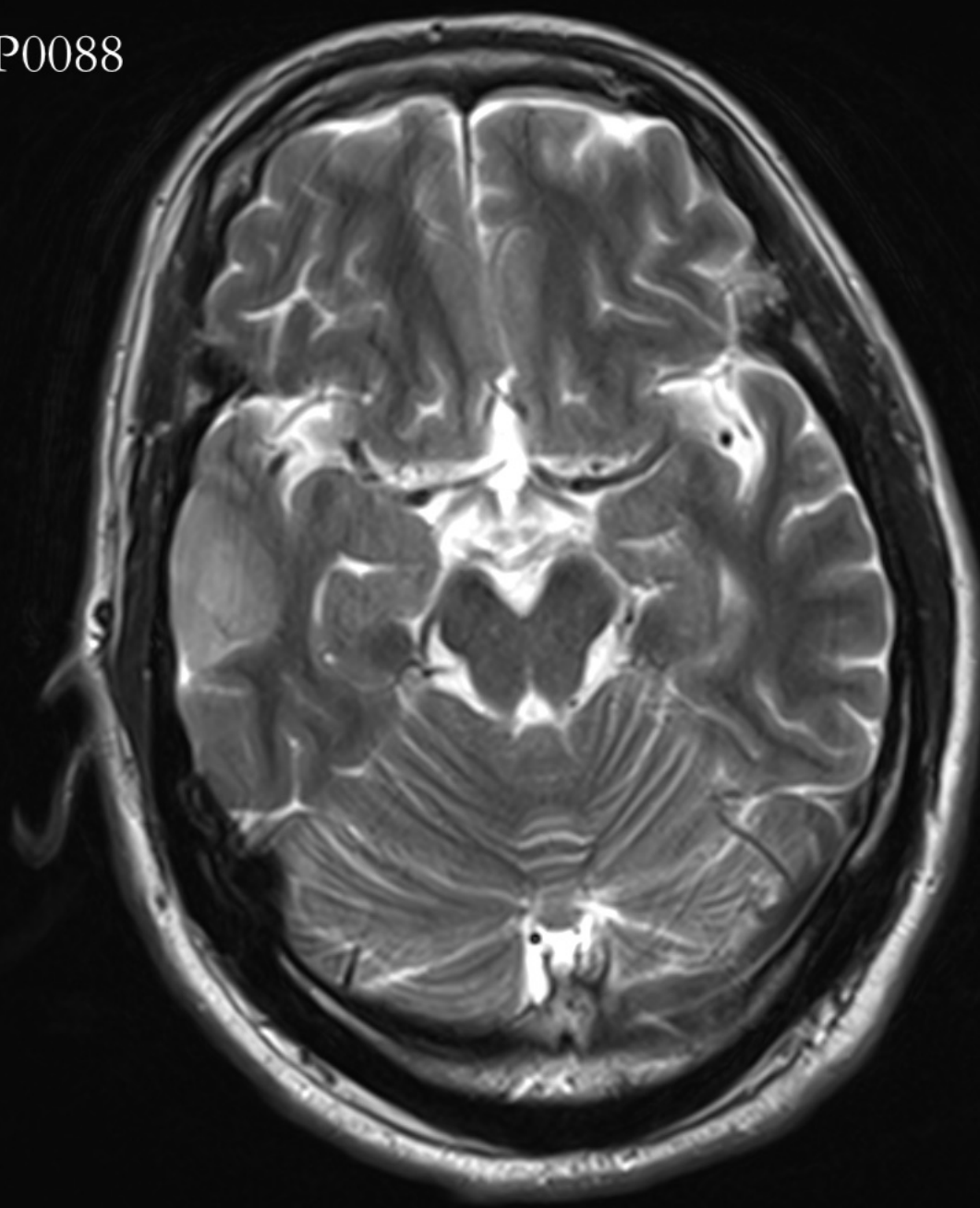

P0106

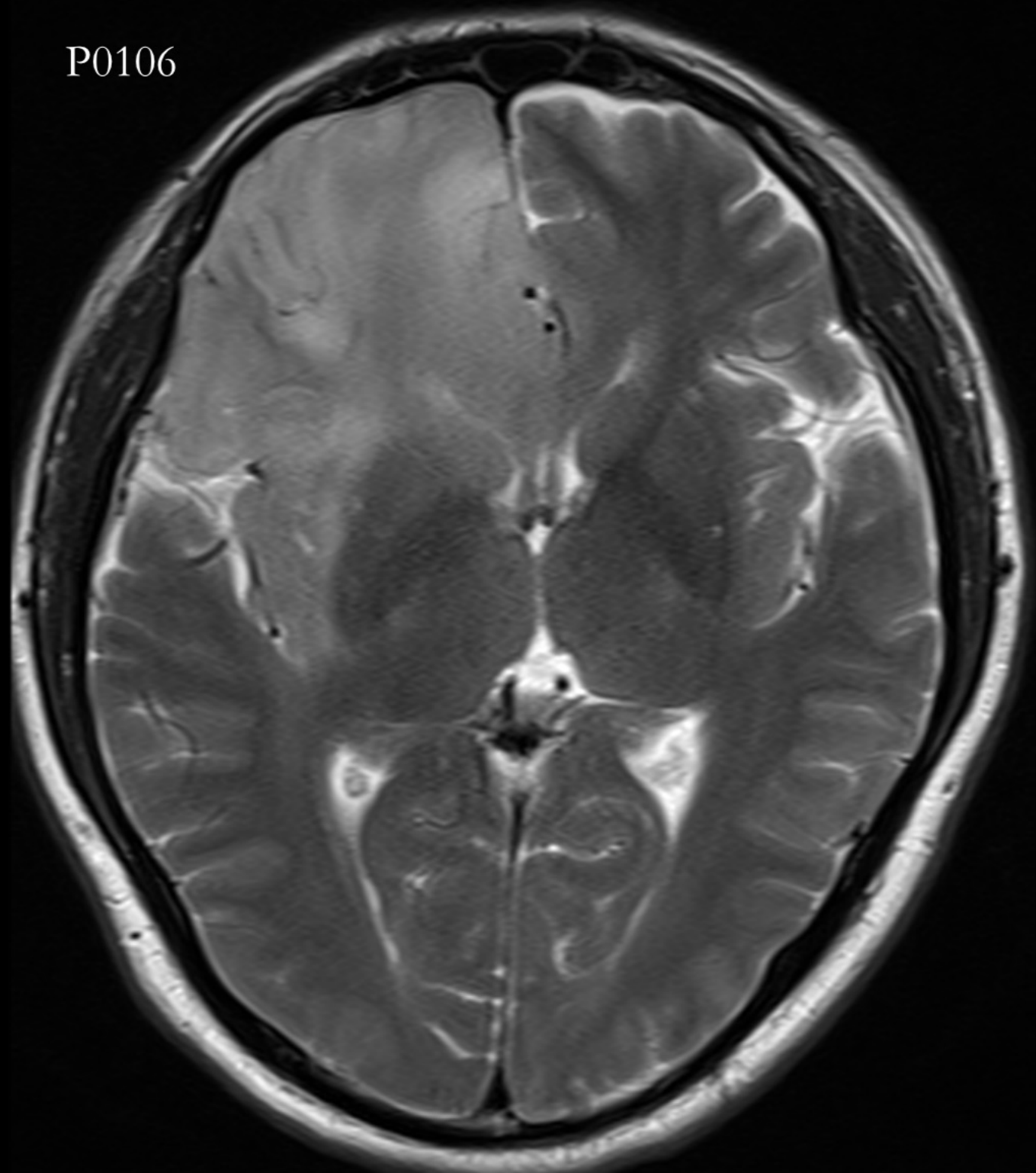

P0109

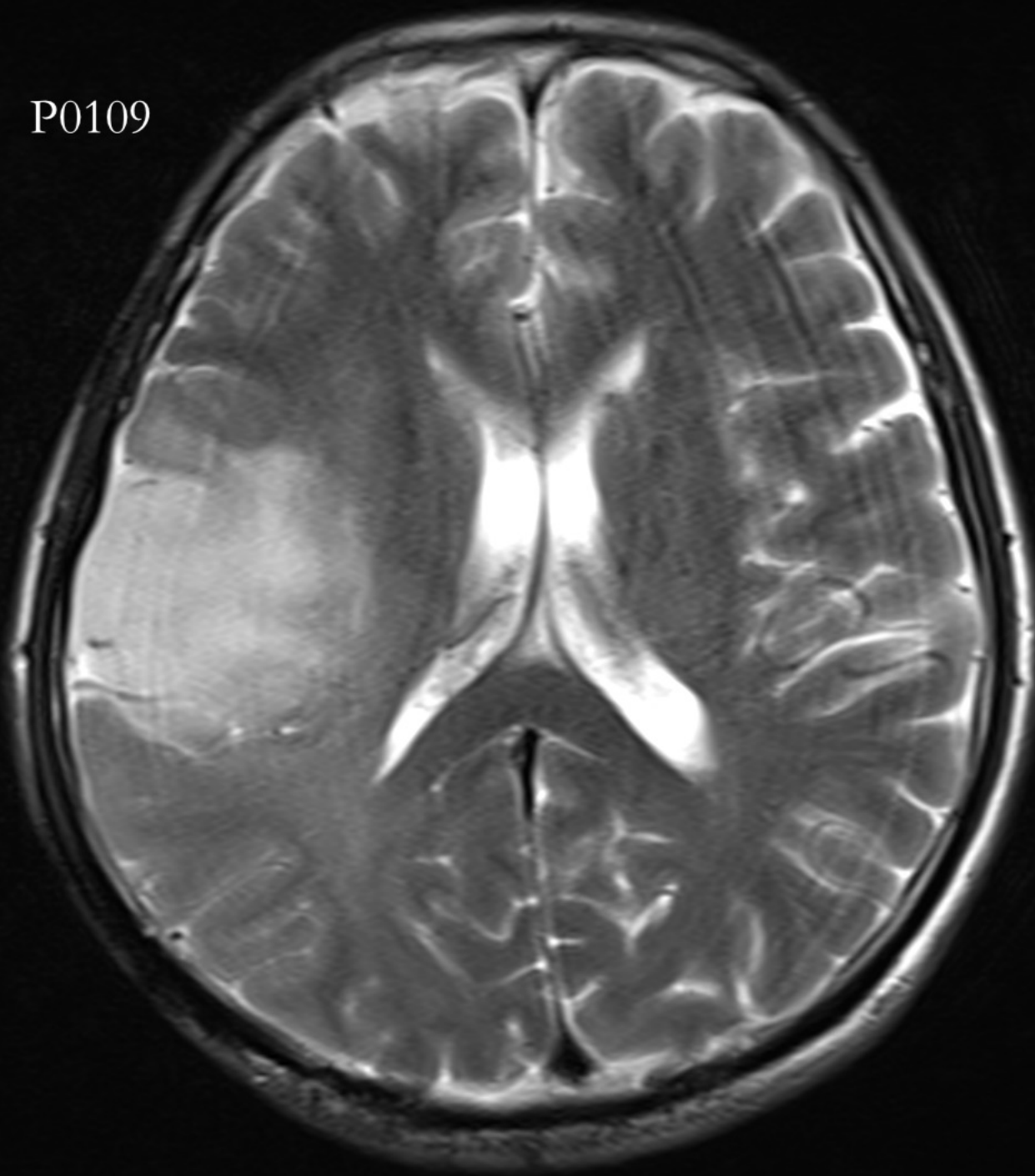

P0115

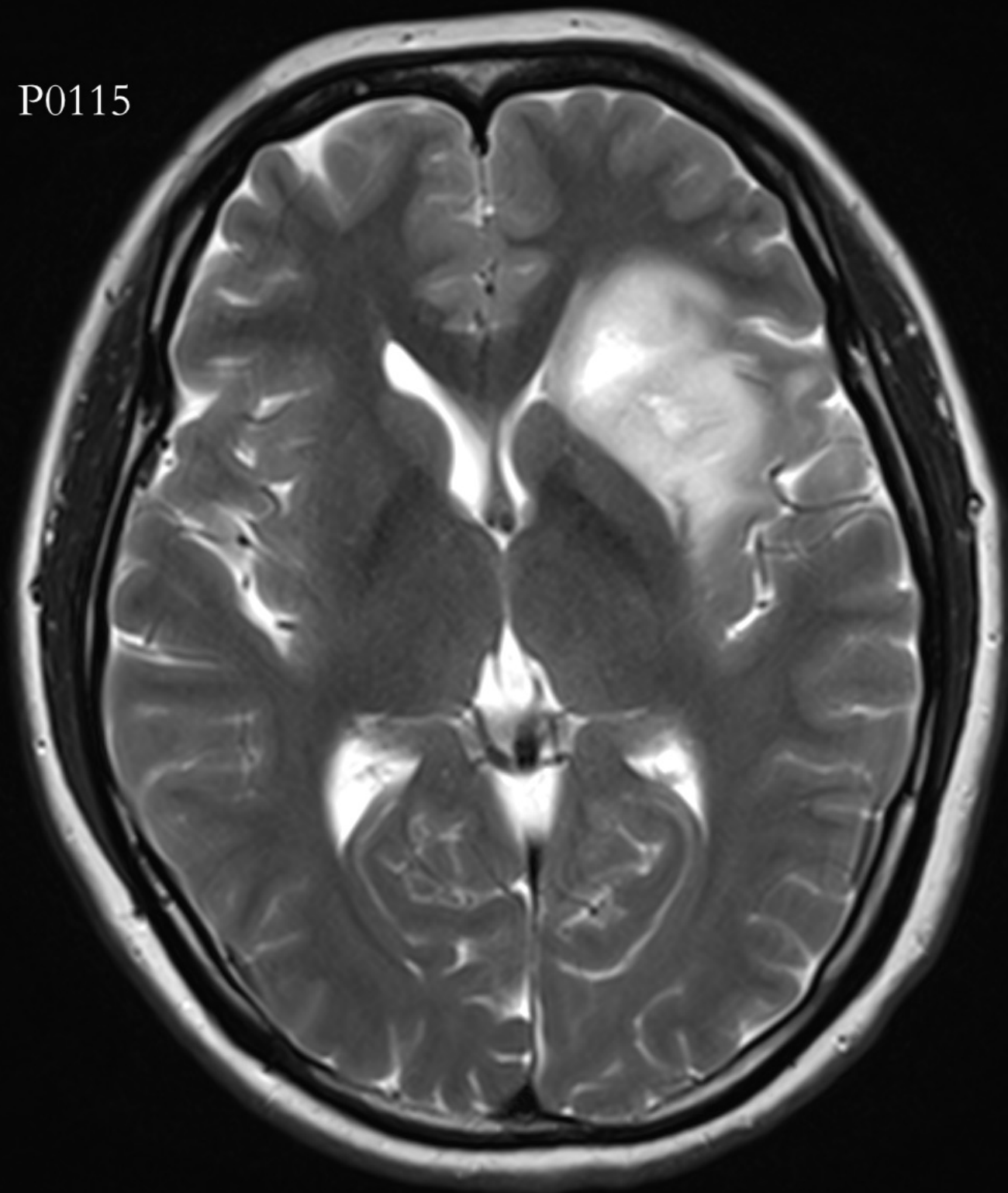

P0116

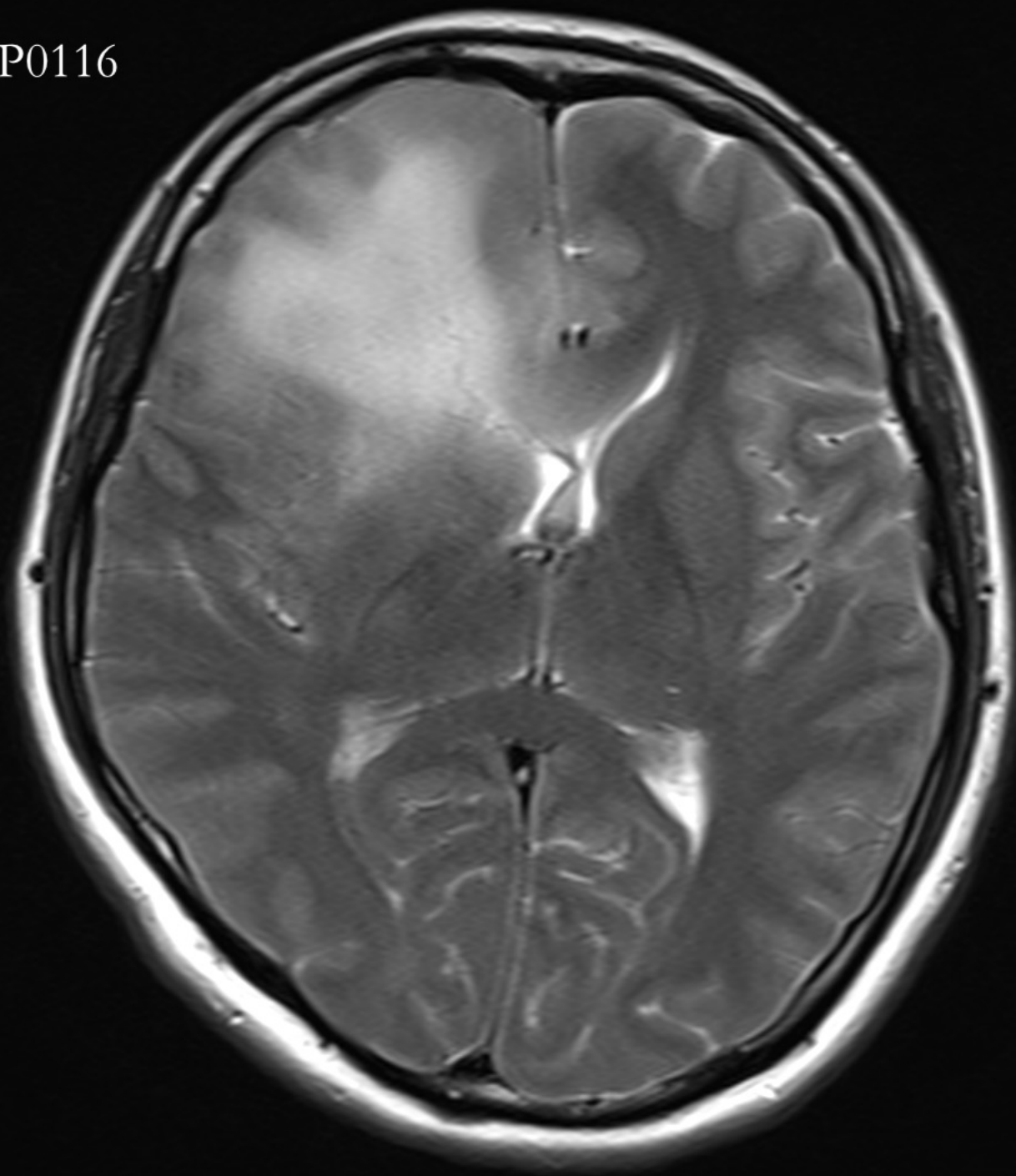

P0120

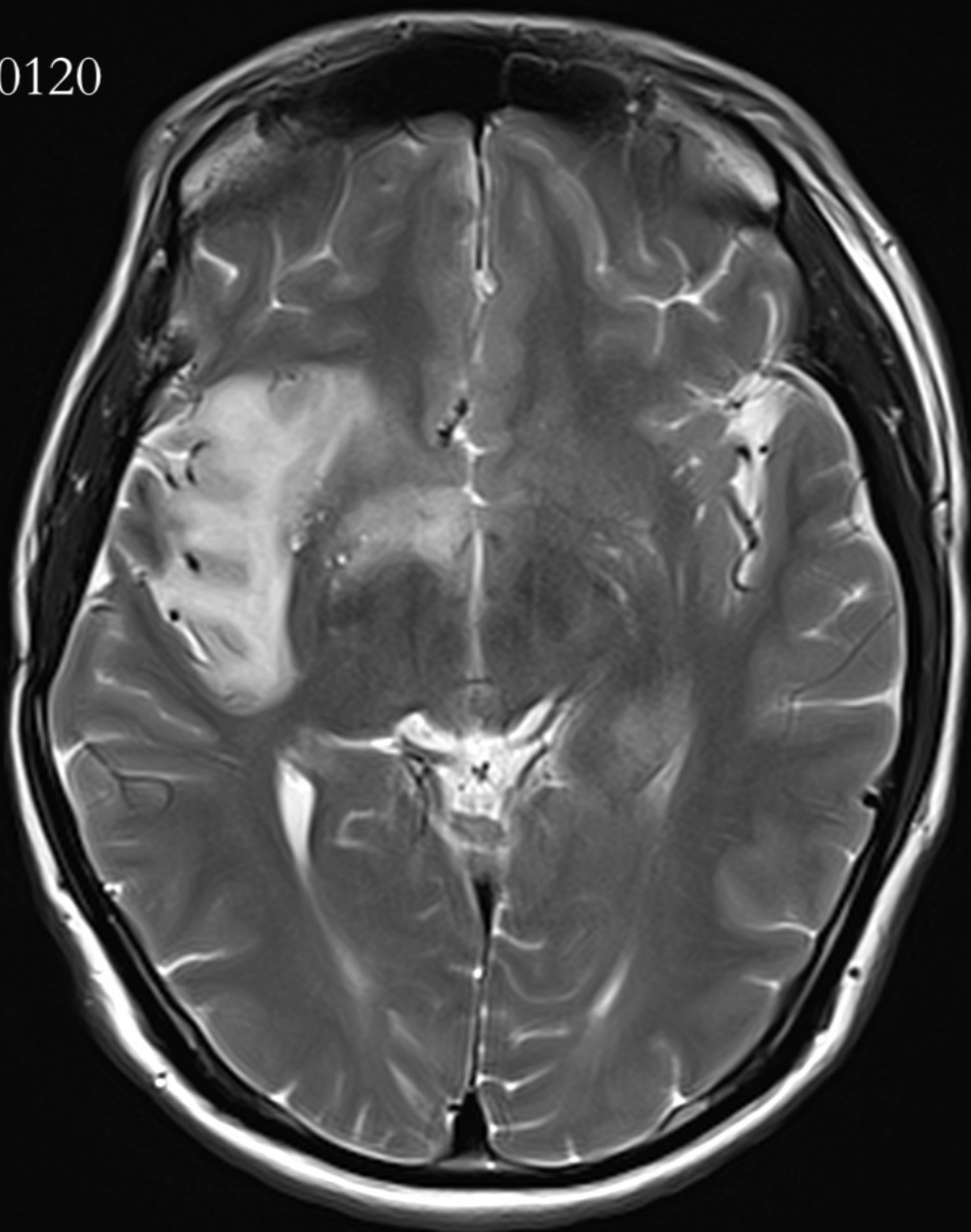

P0121

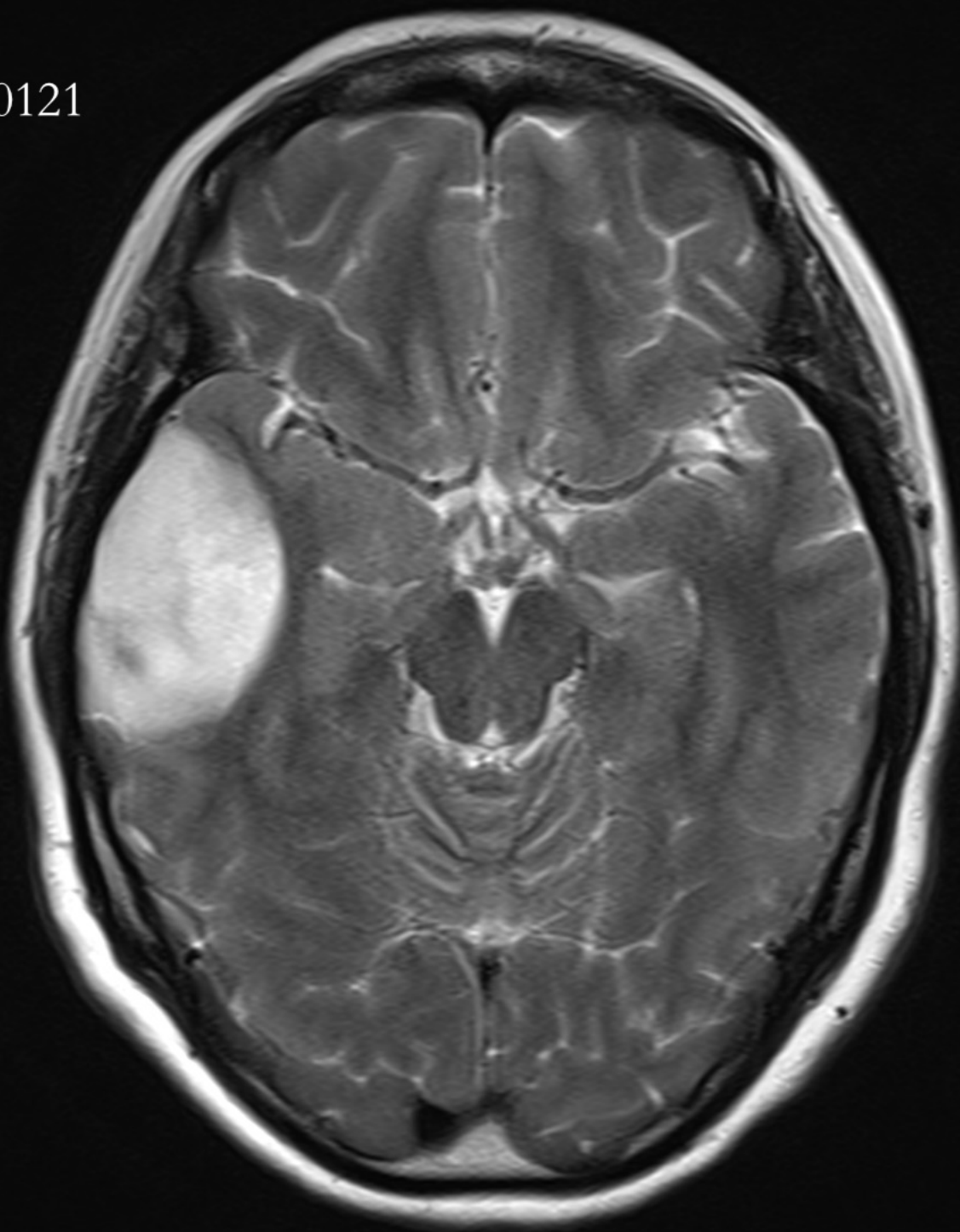

P0122

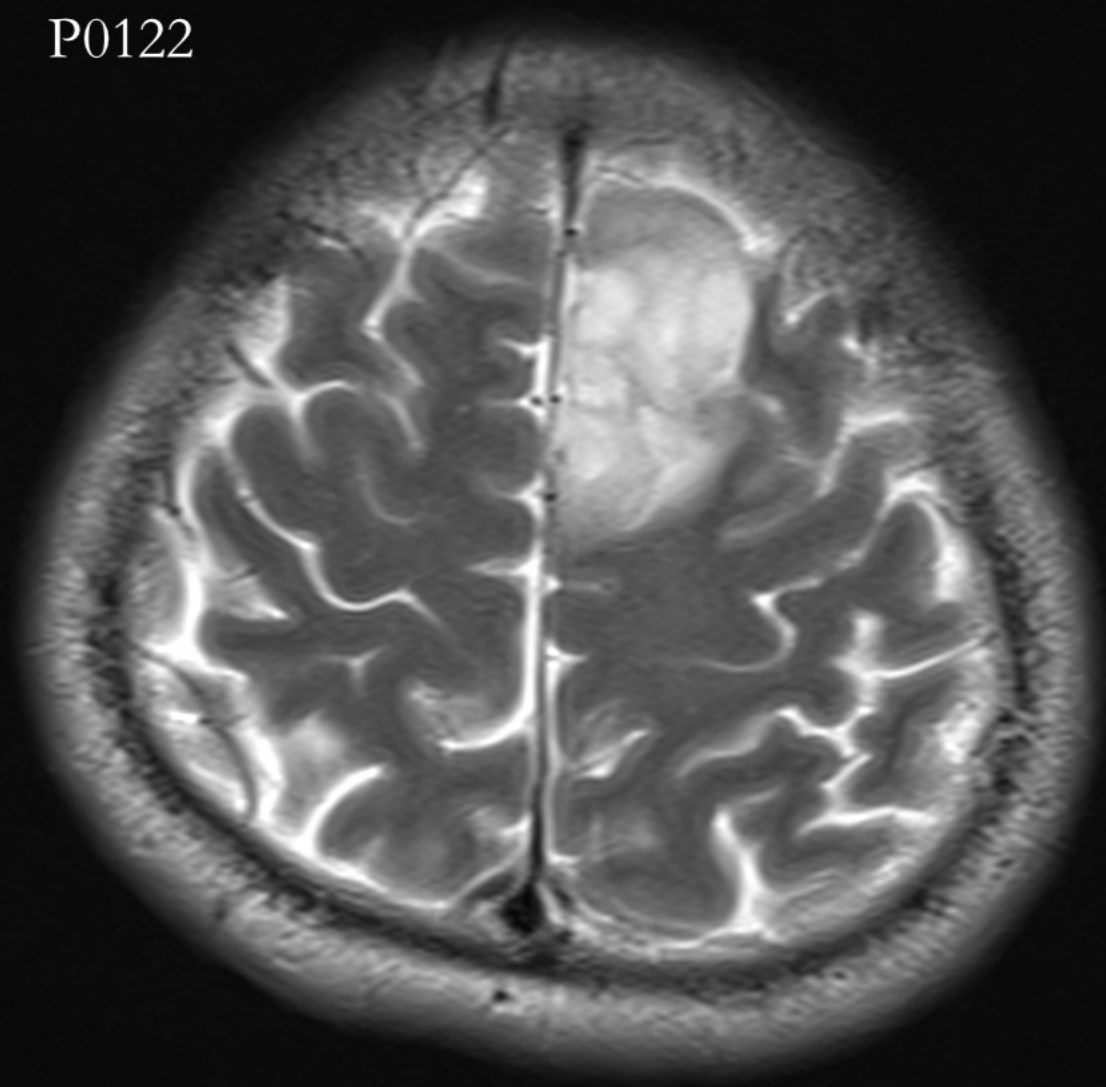

P0123

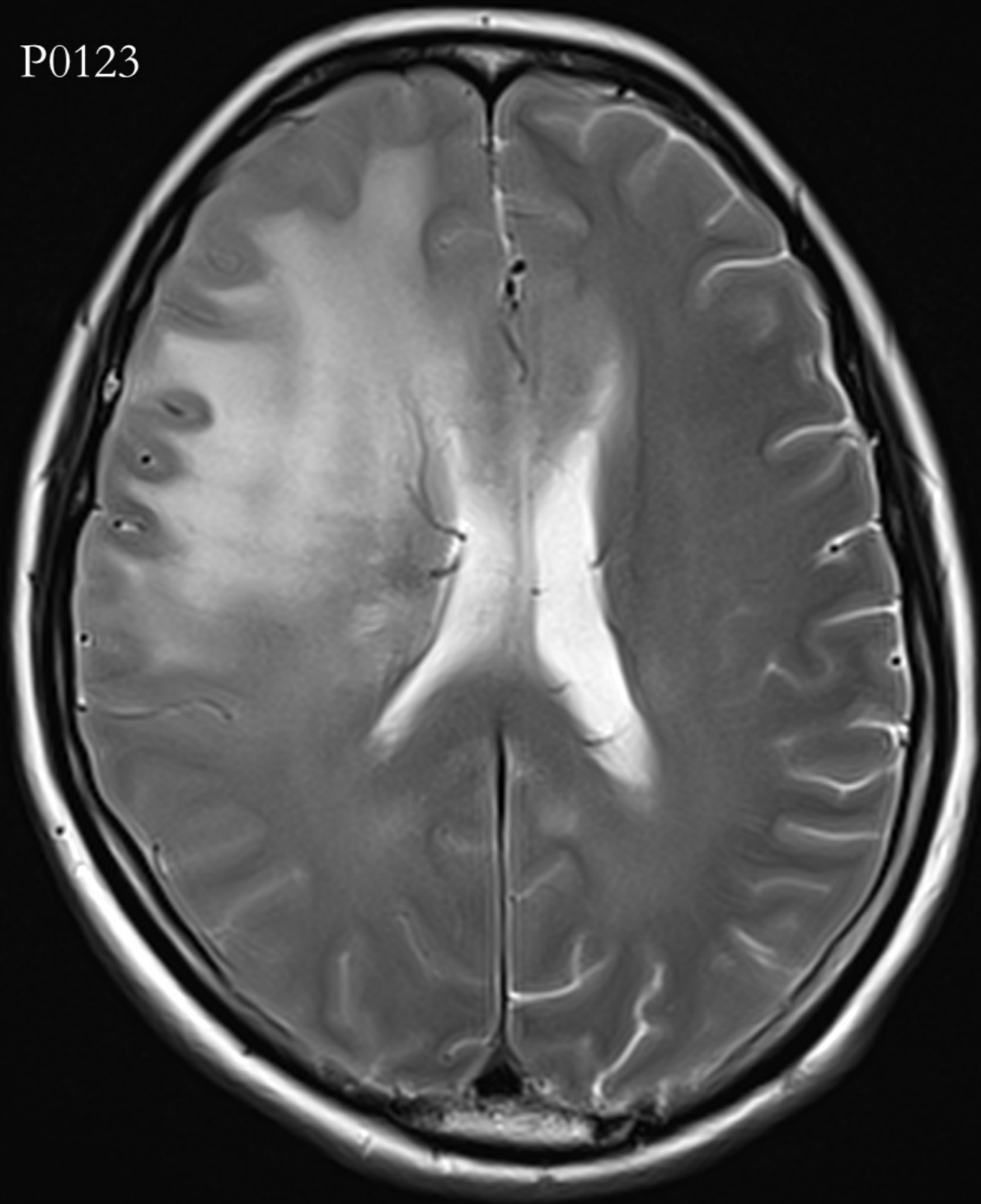

P0124

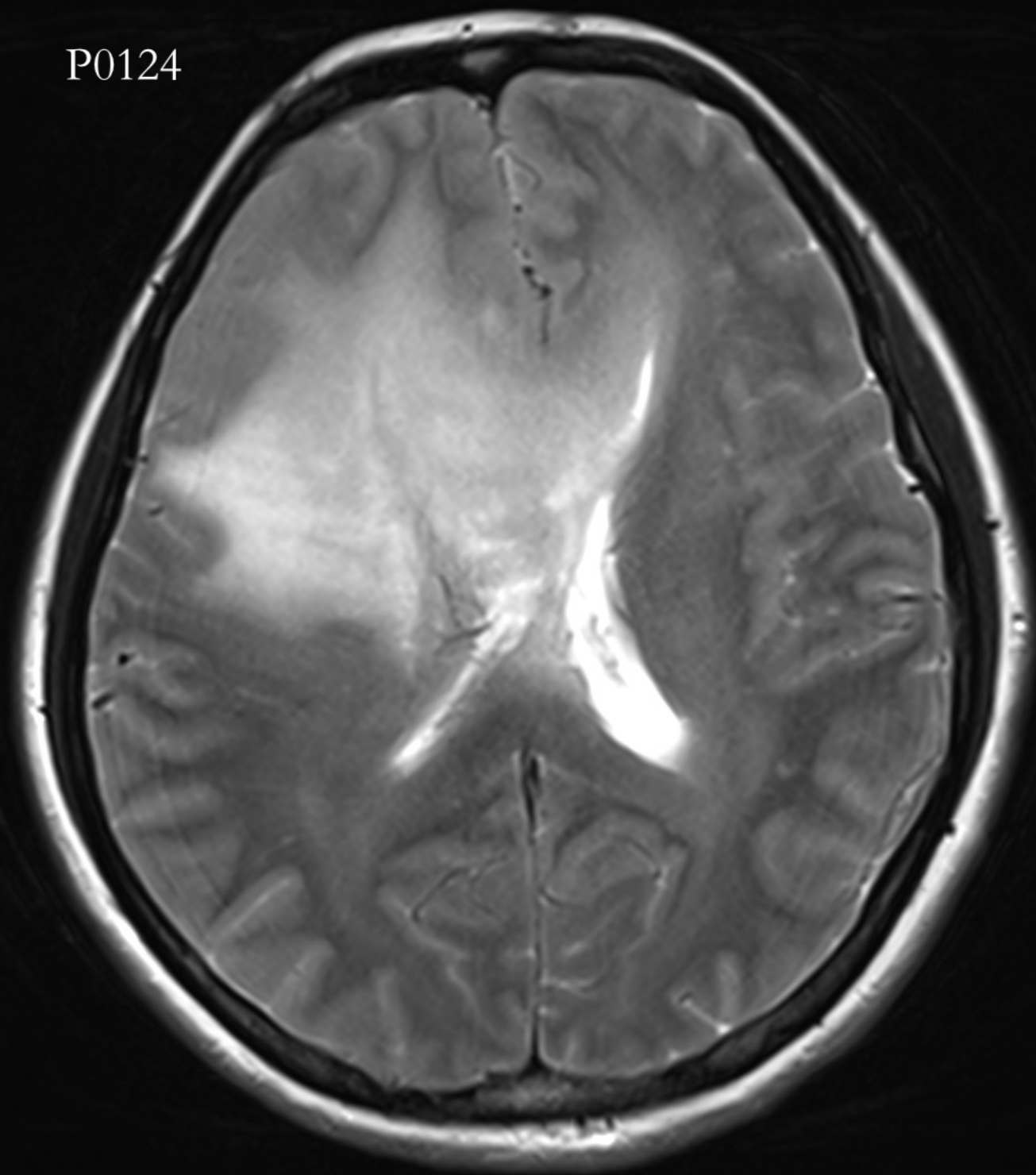

P0125

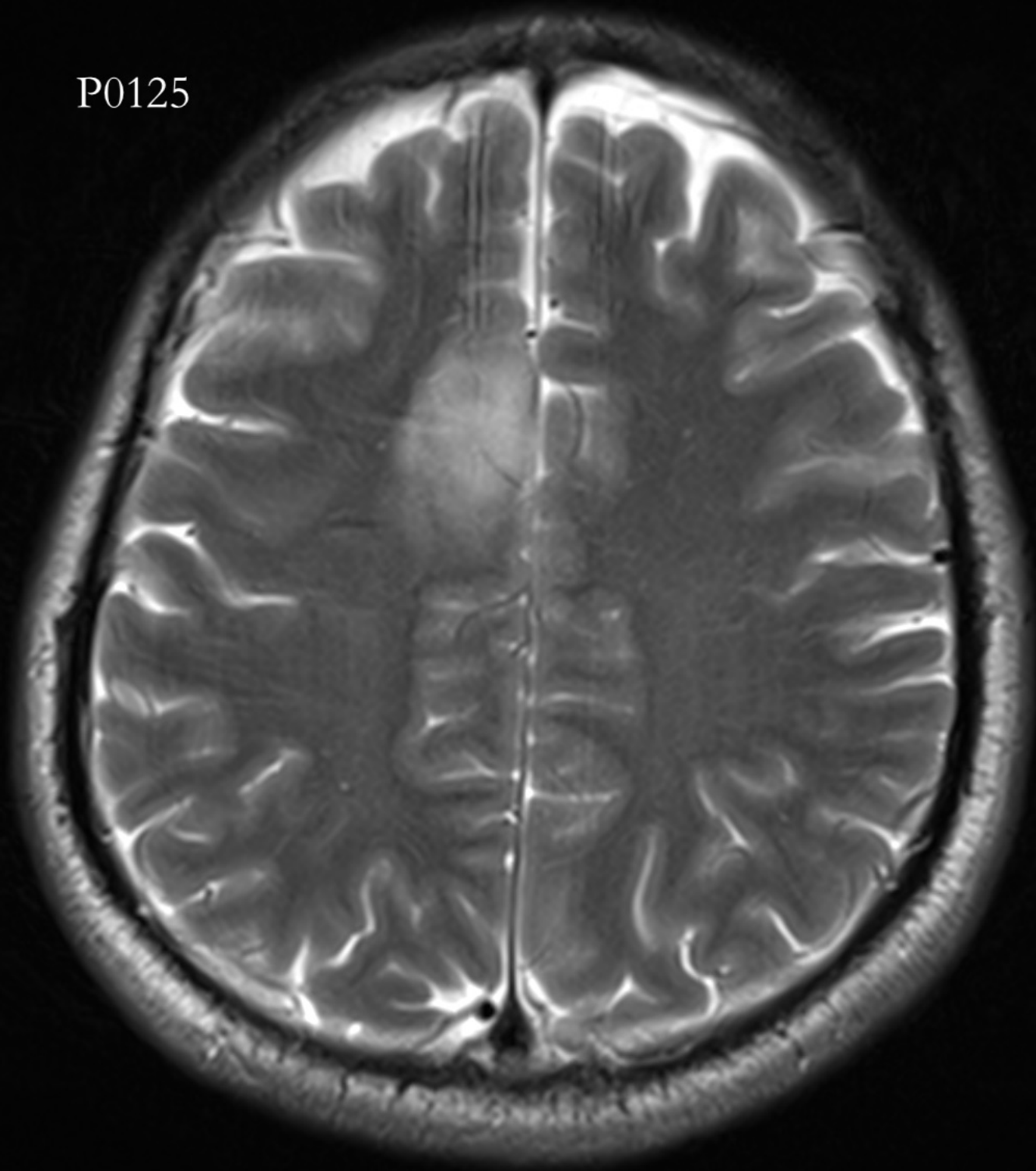

P0126

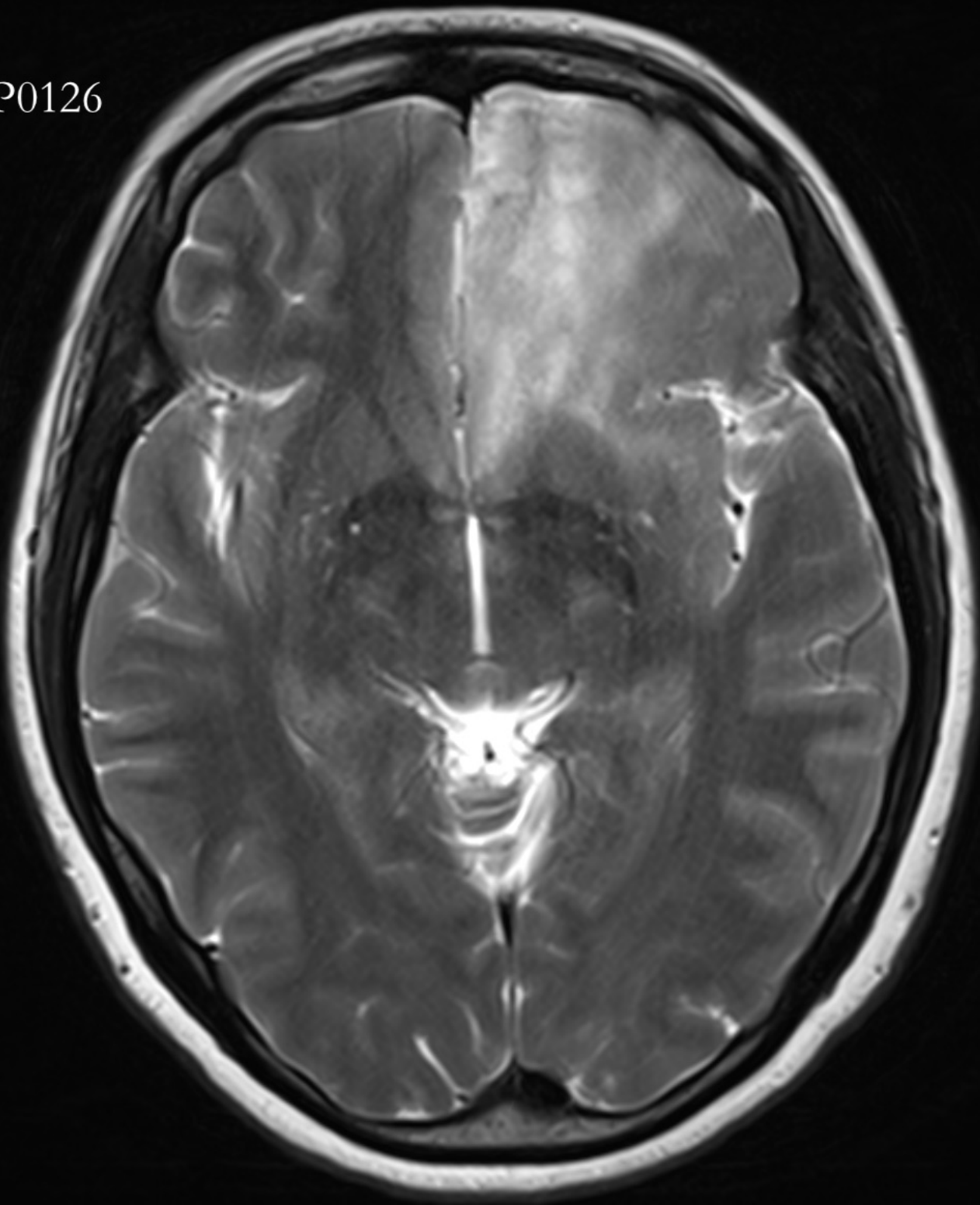

P0127

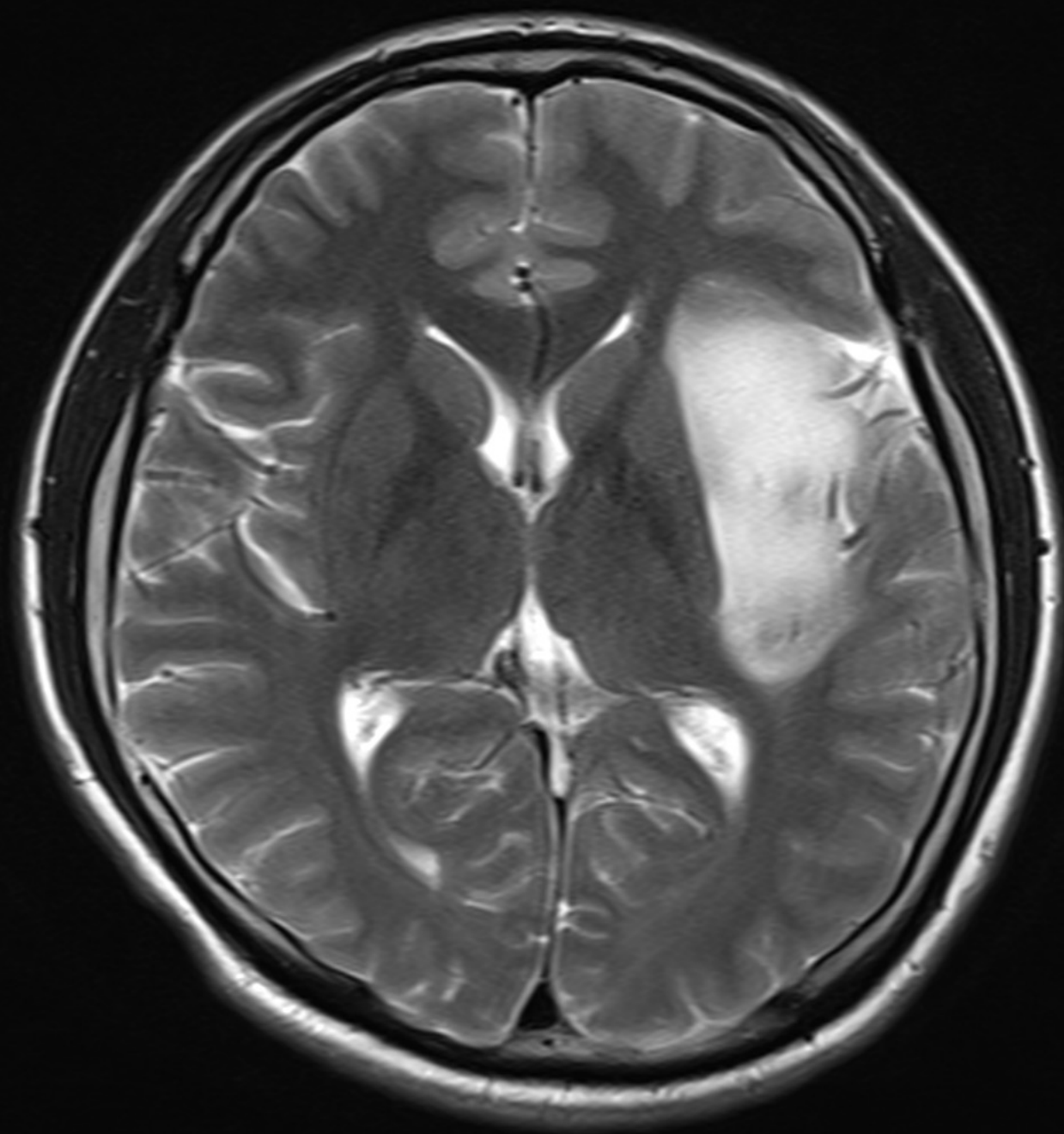

P0128

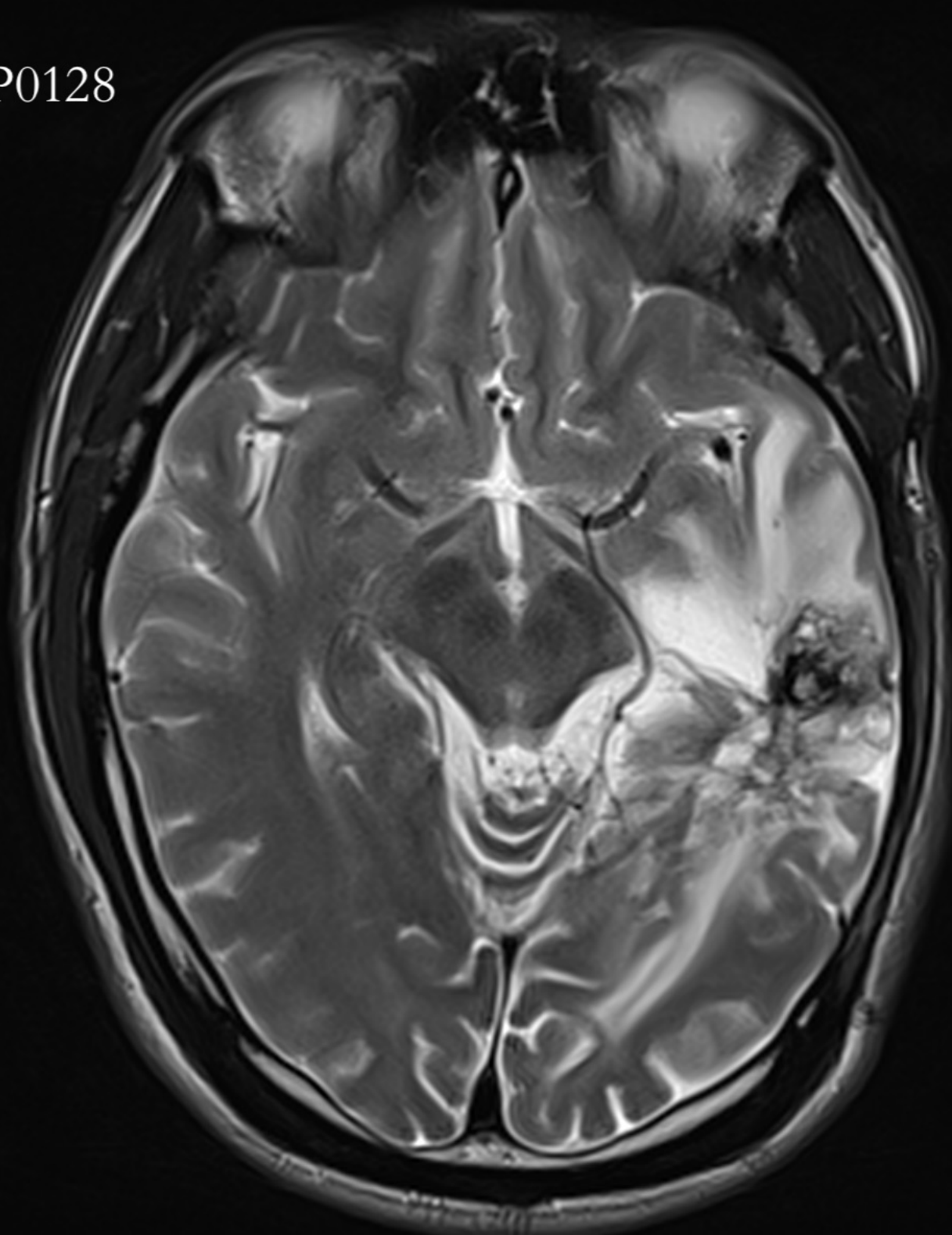

P0129

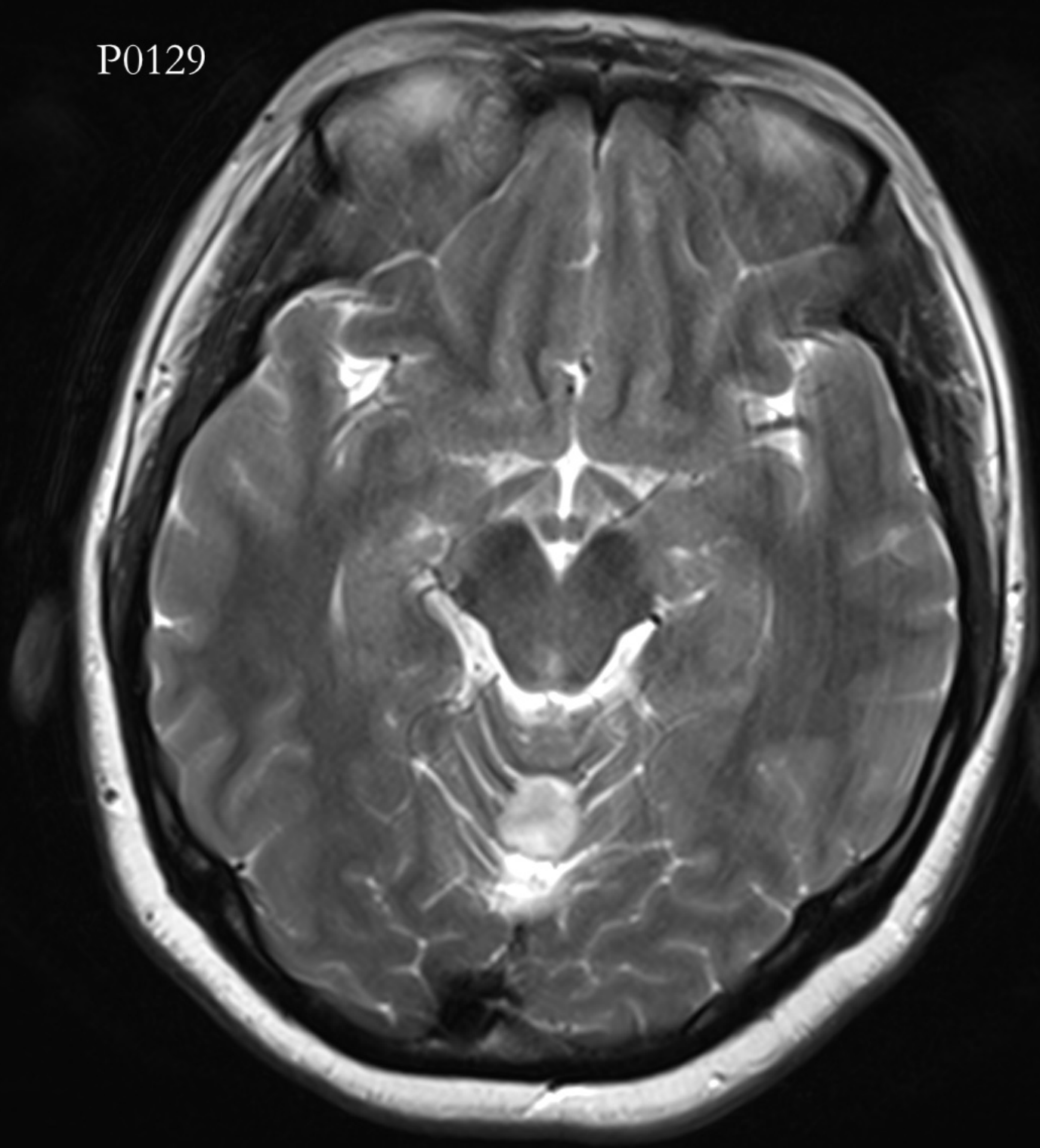

P0130

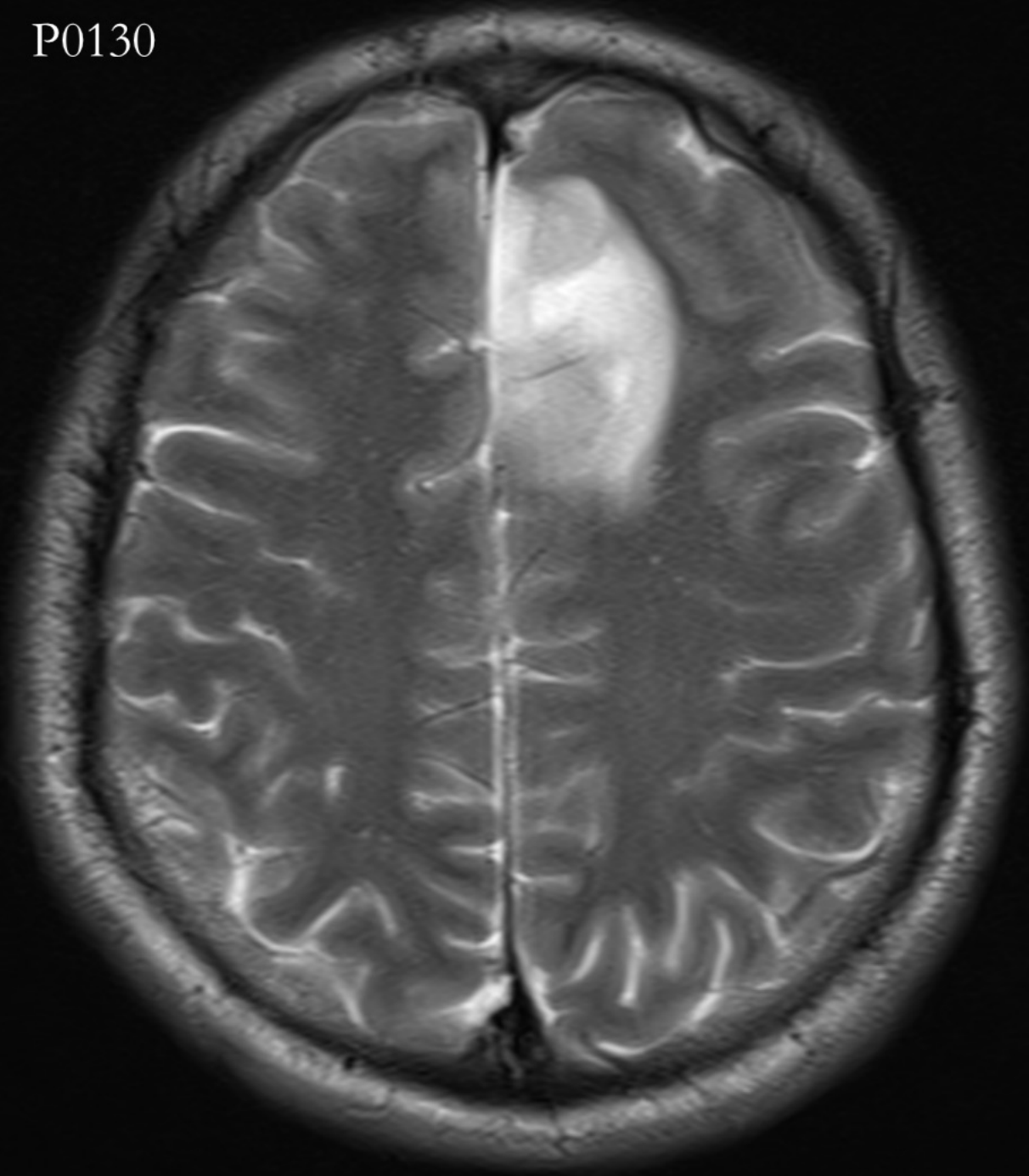

P0132

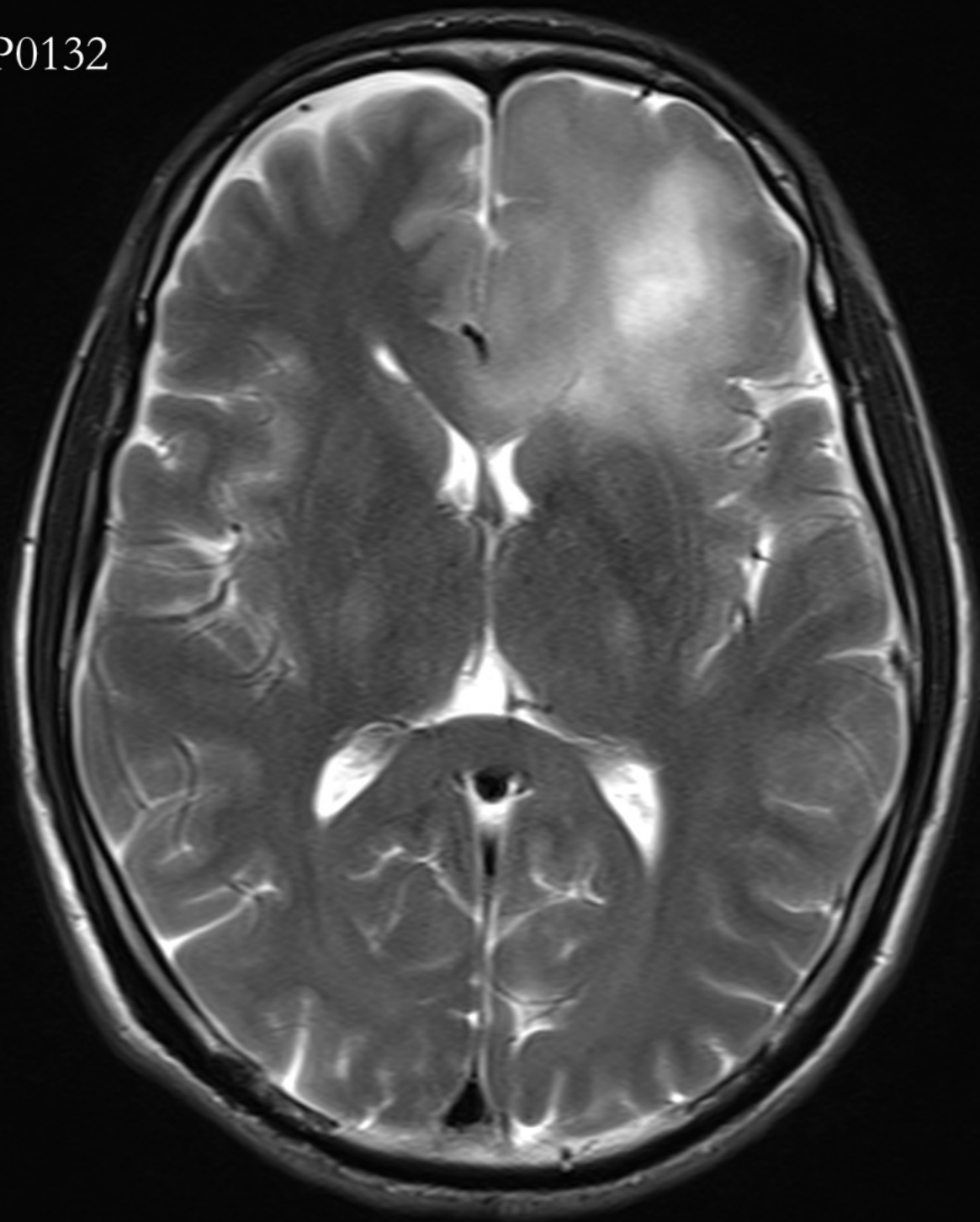

P0133

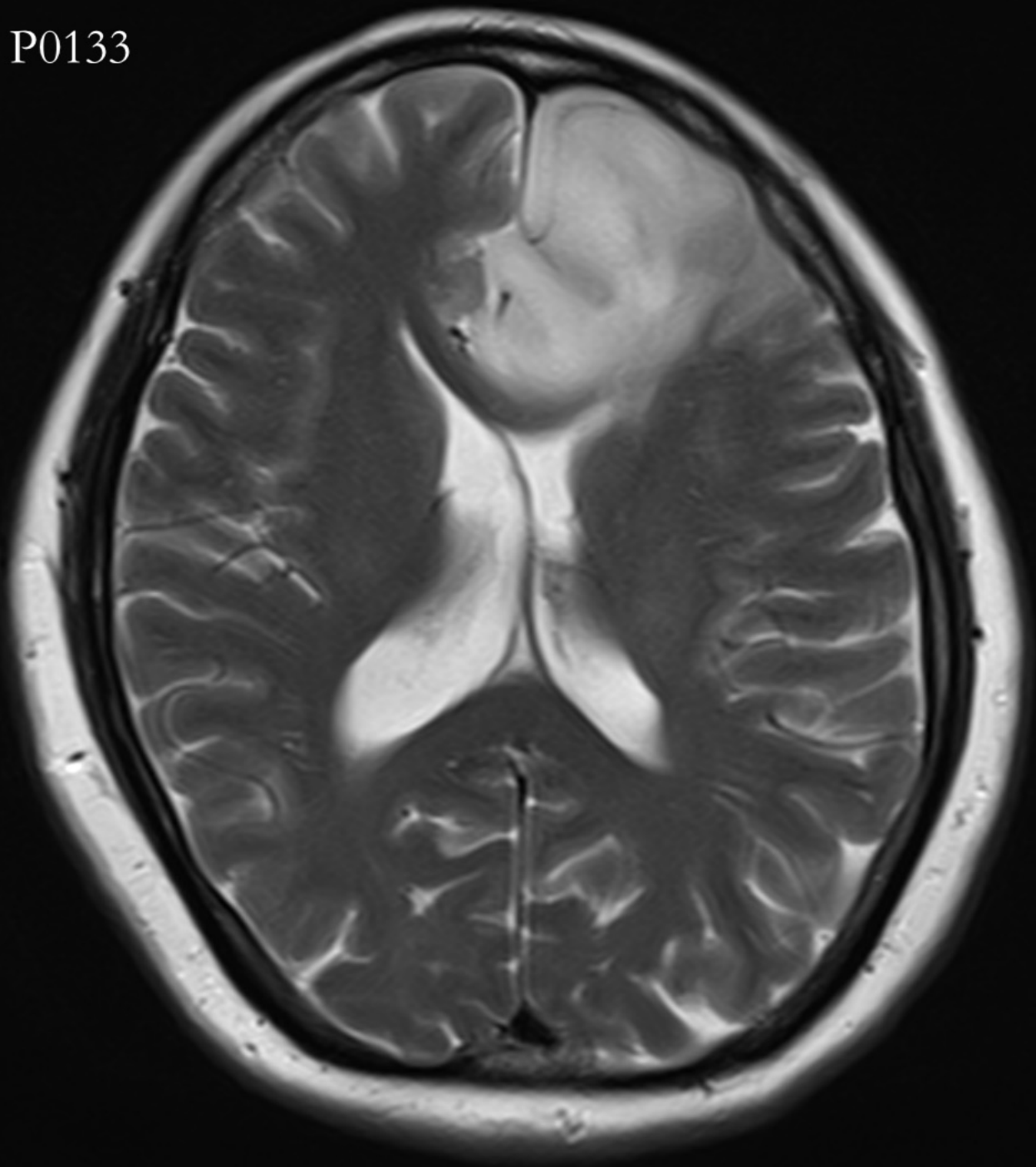

P0134

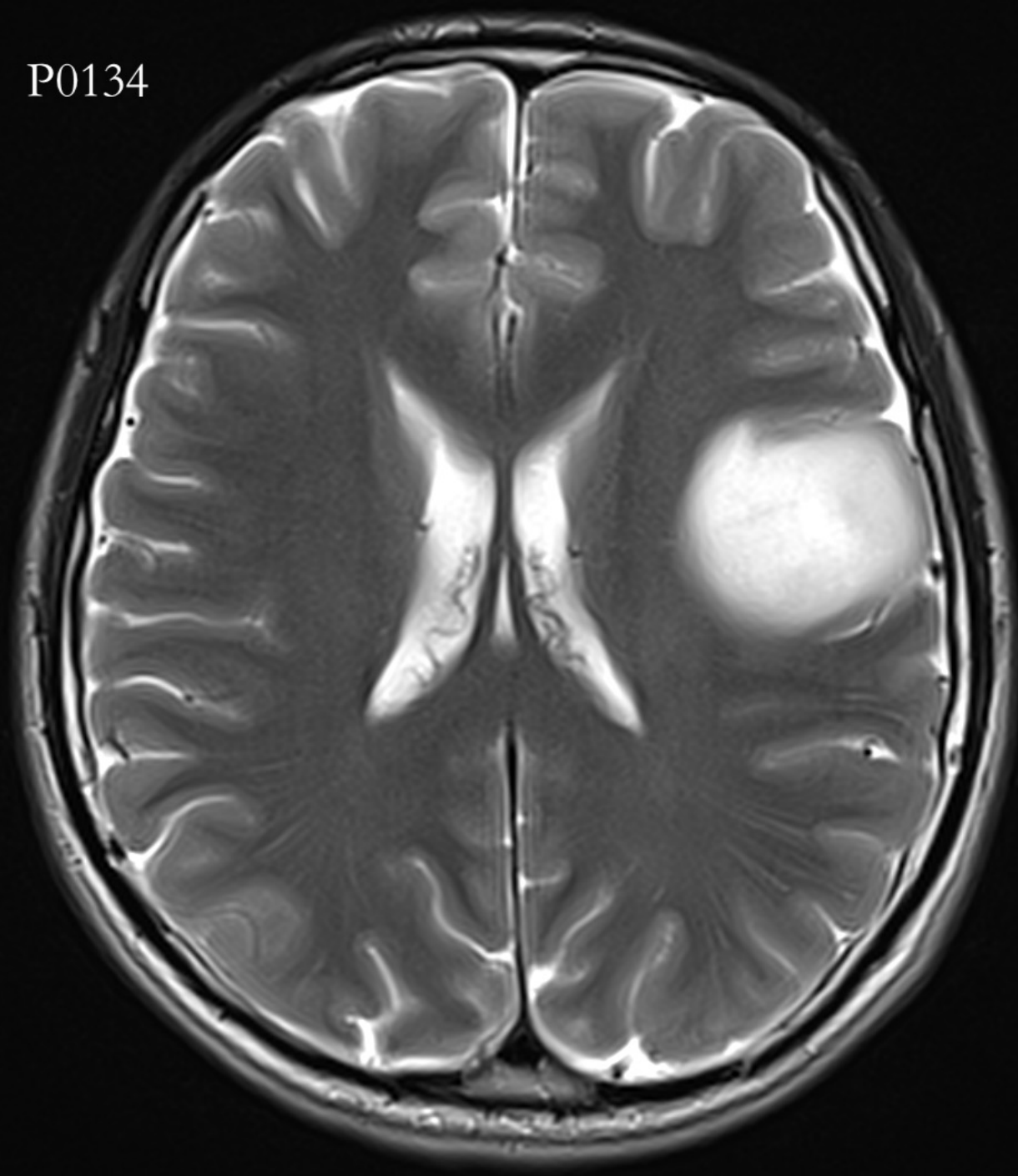

P0135

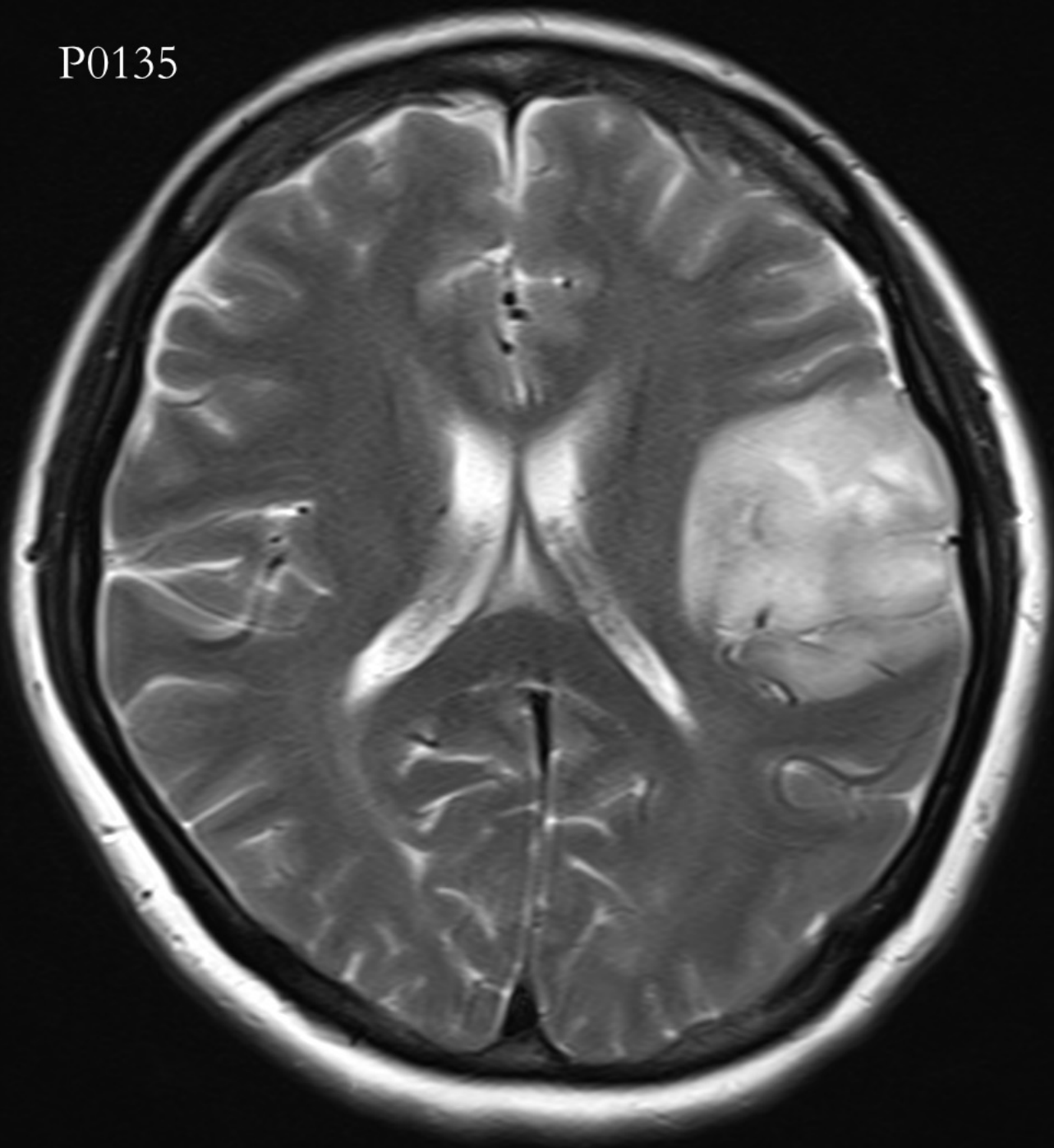

P0137

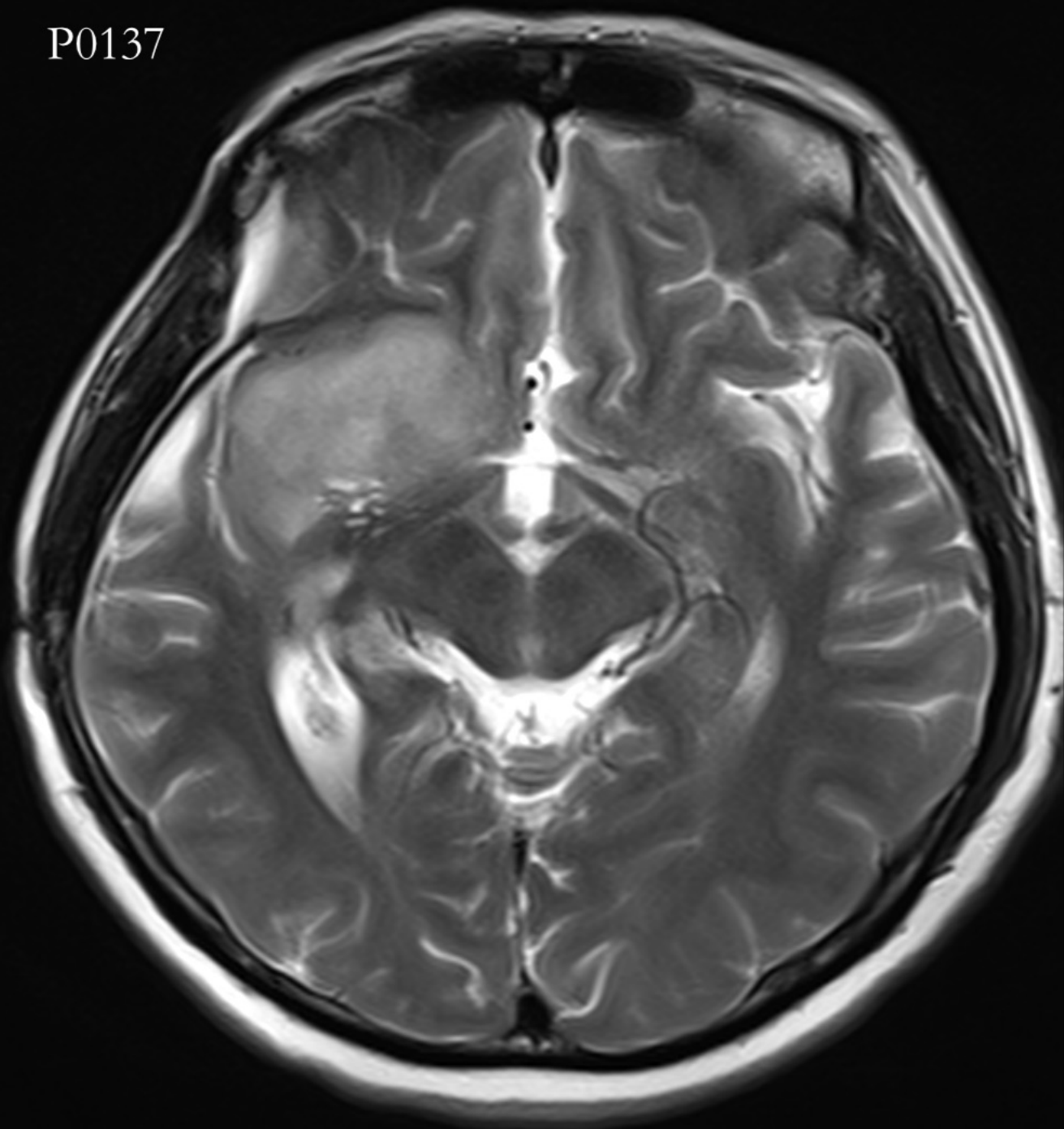

P0139

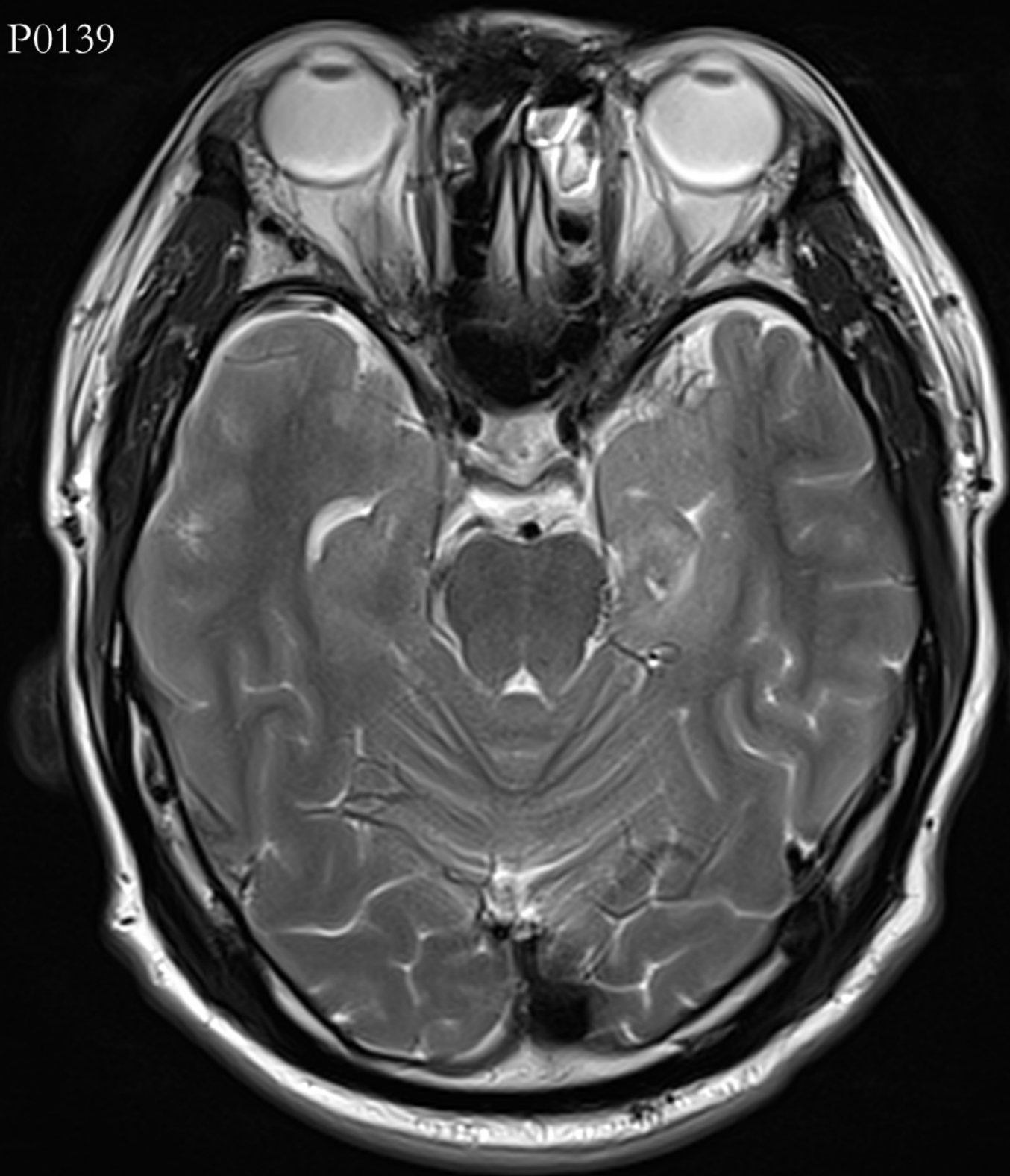

P0142

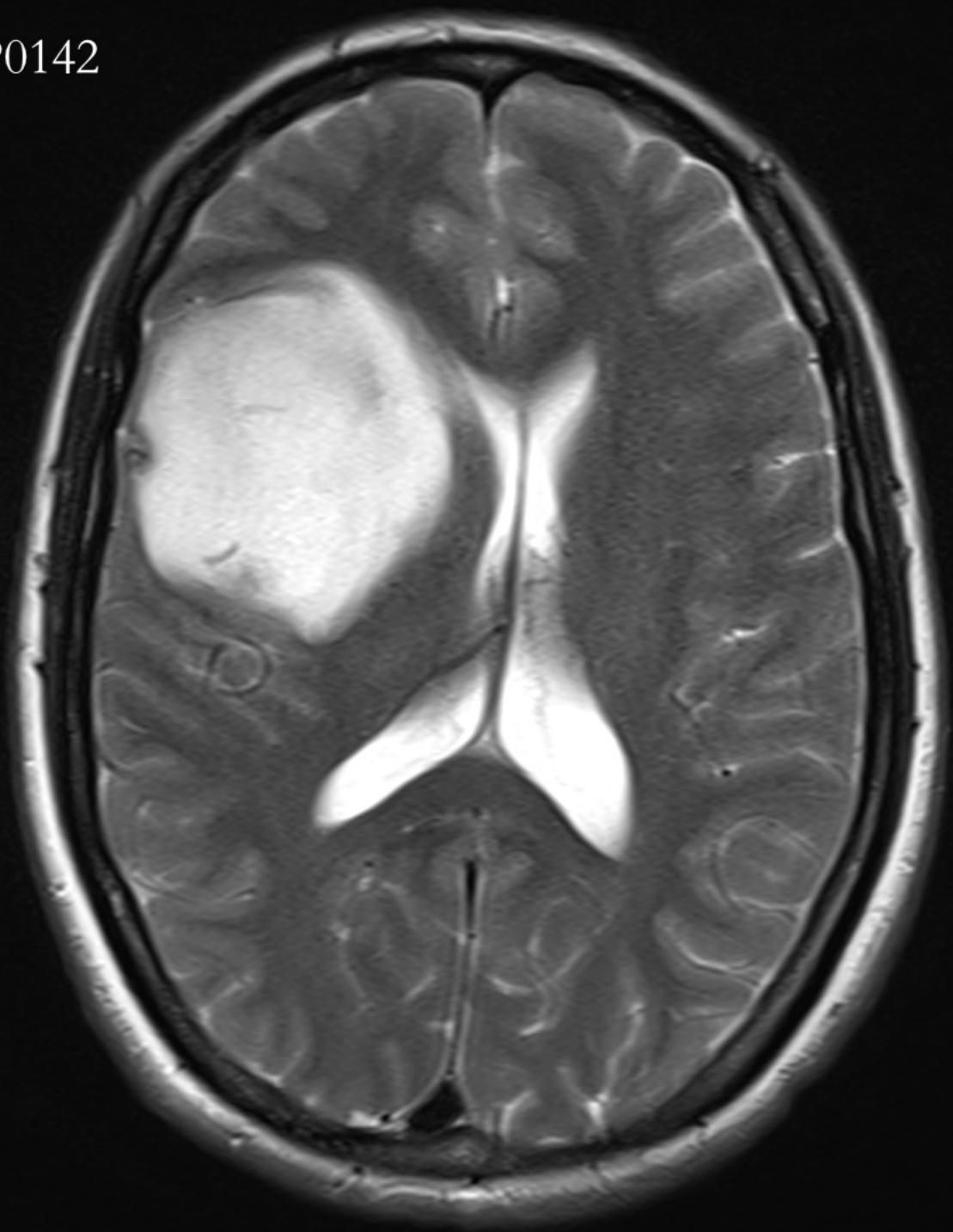

P0148

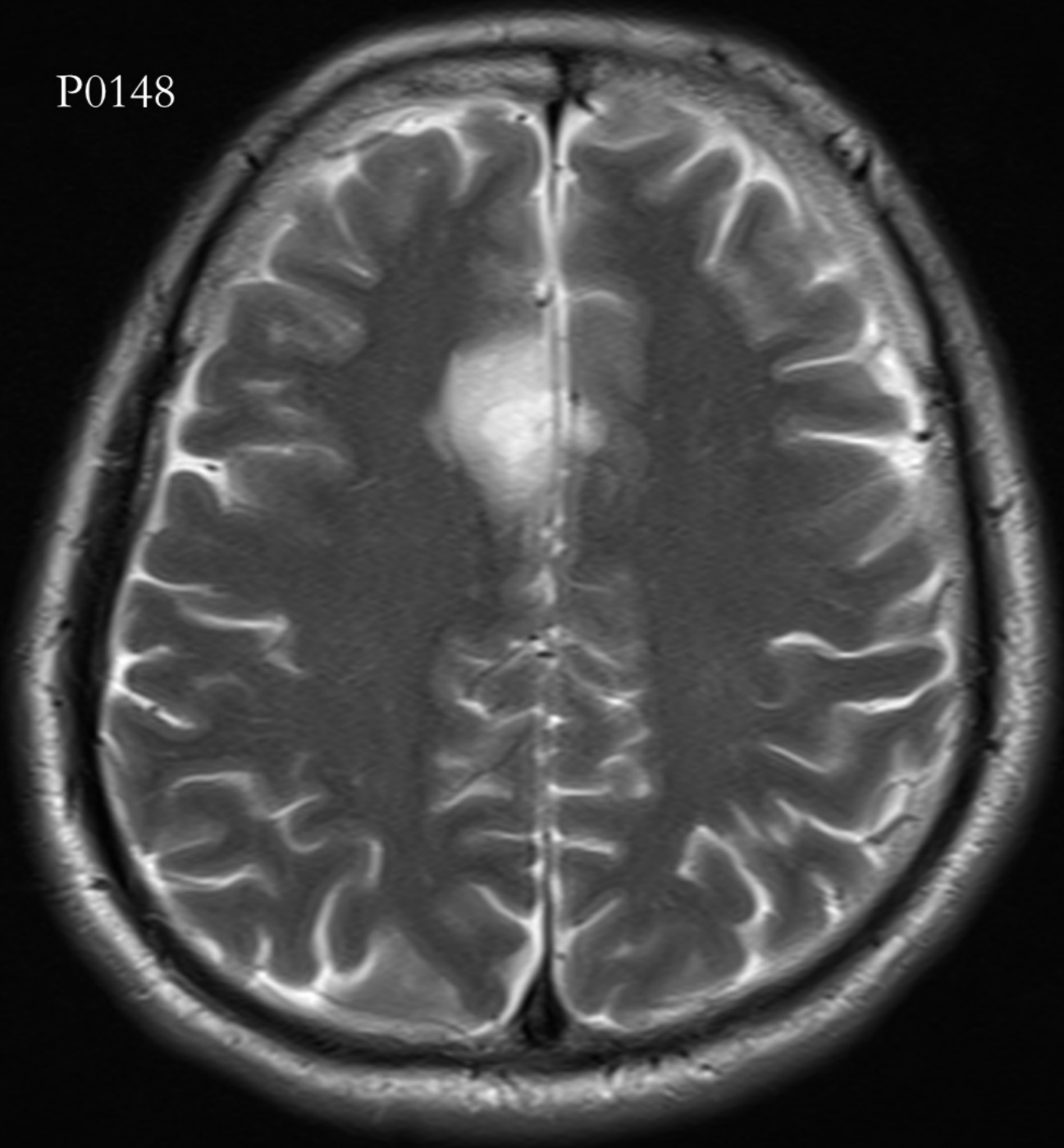

P0149

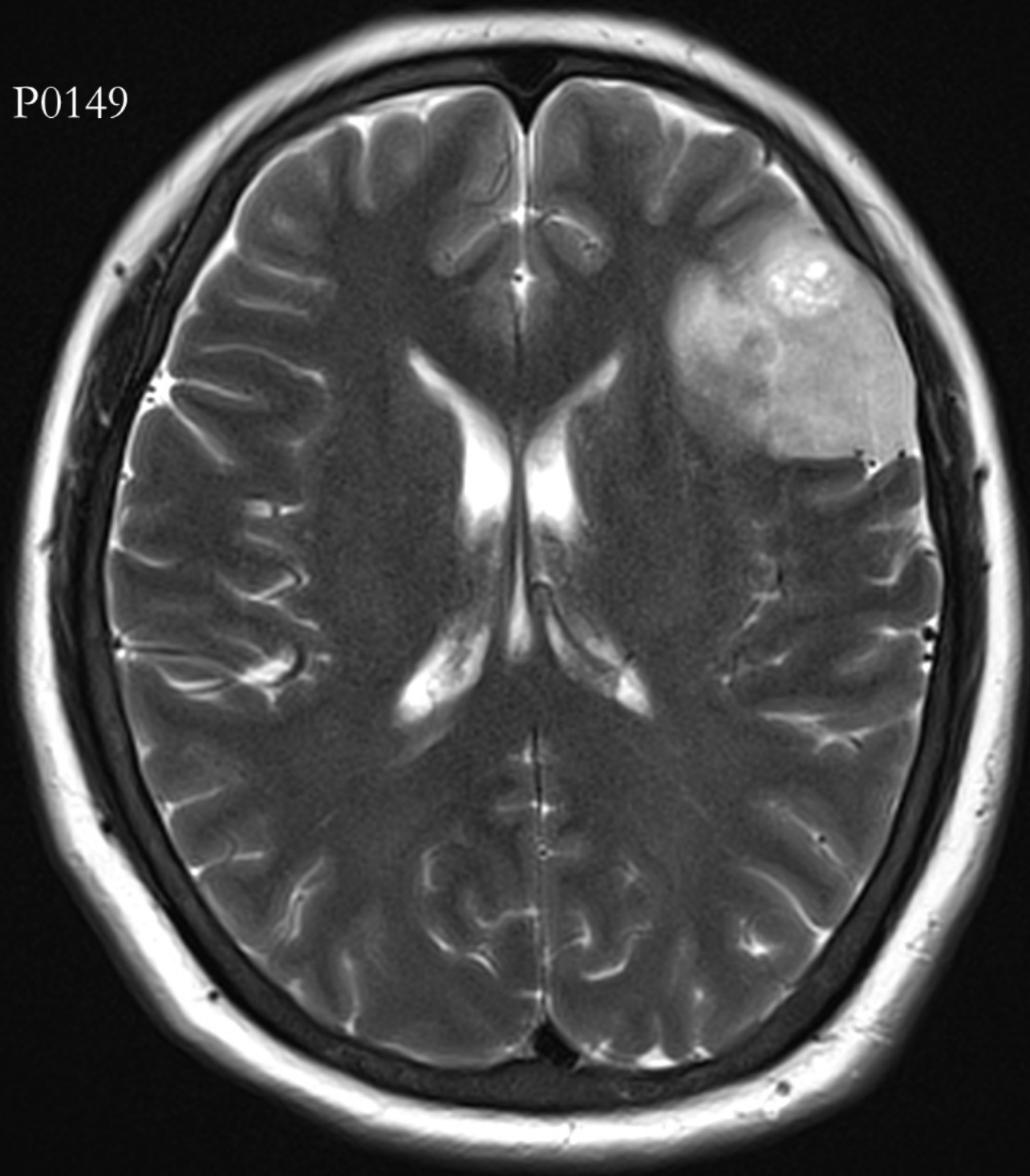

P0152

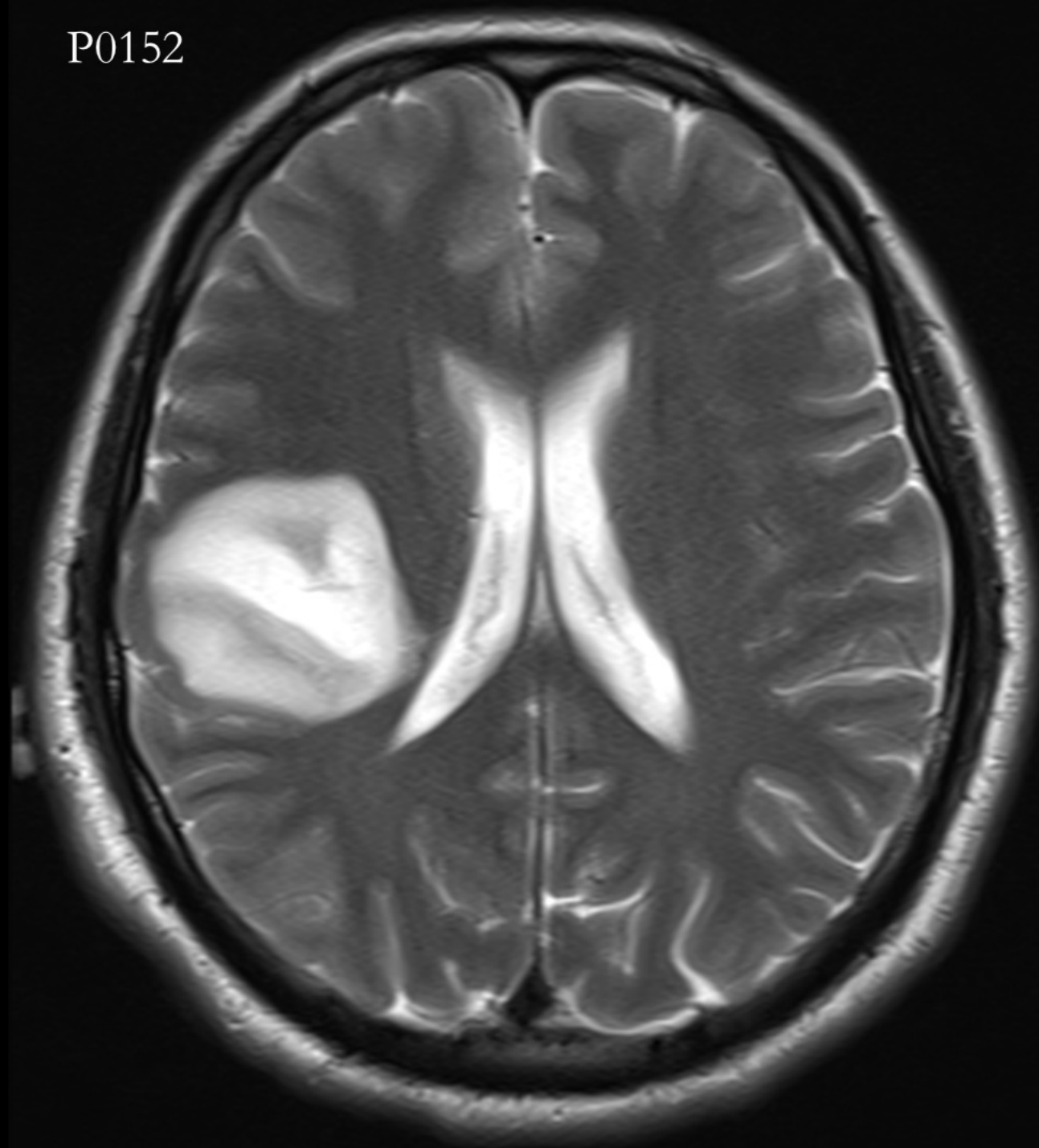

P0153

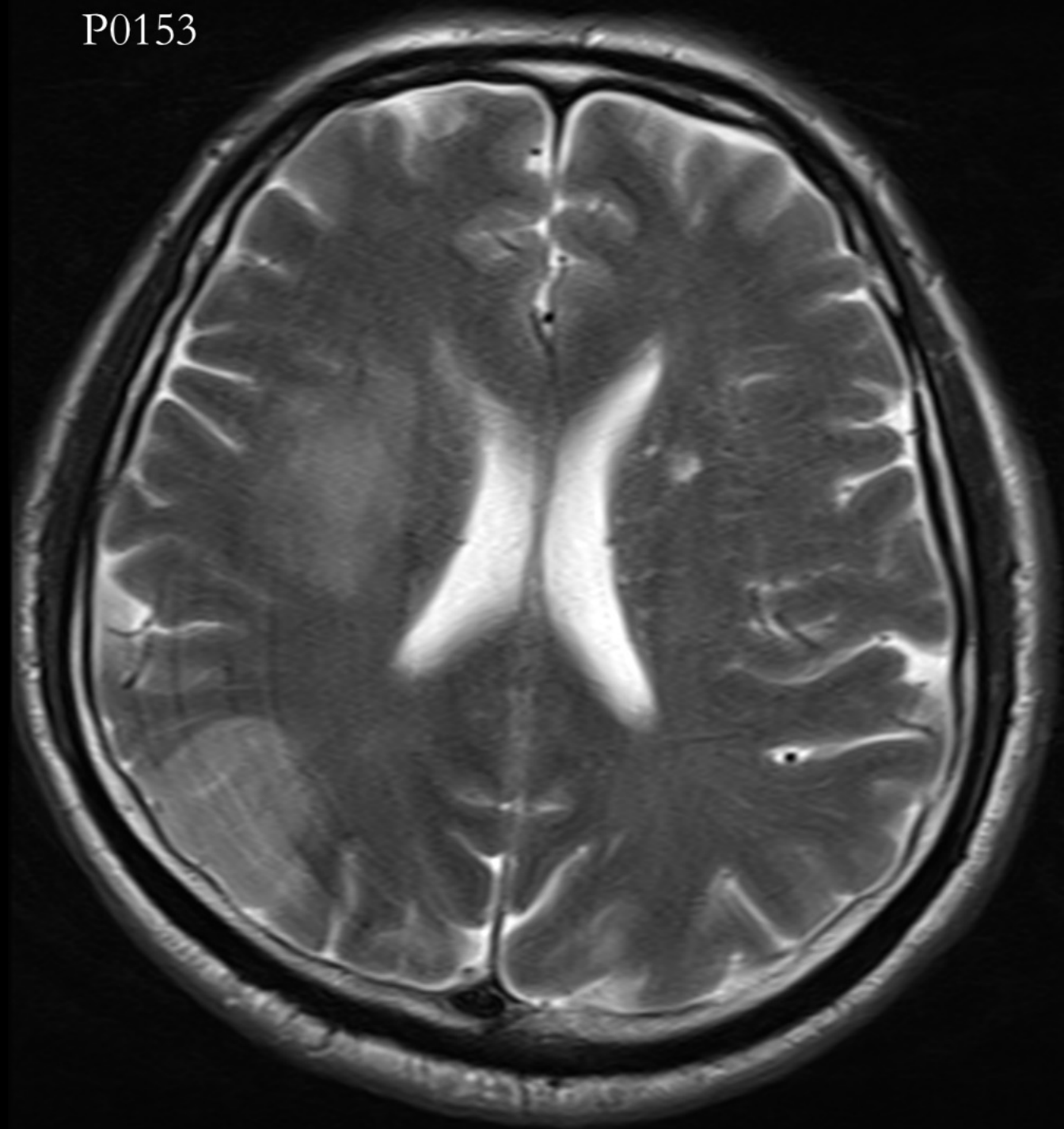

P0156

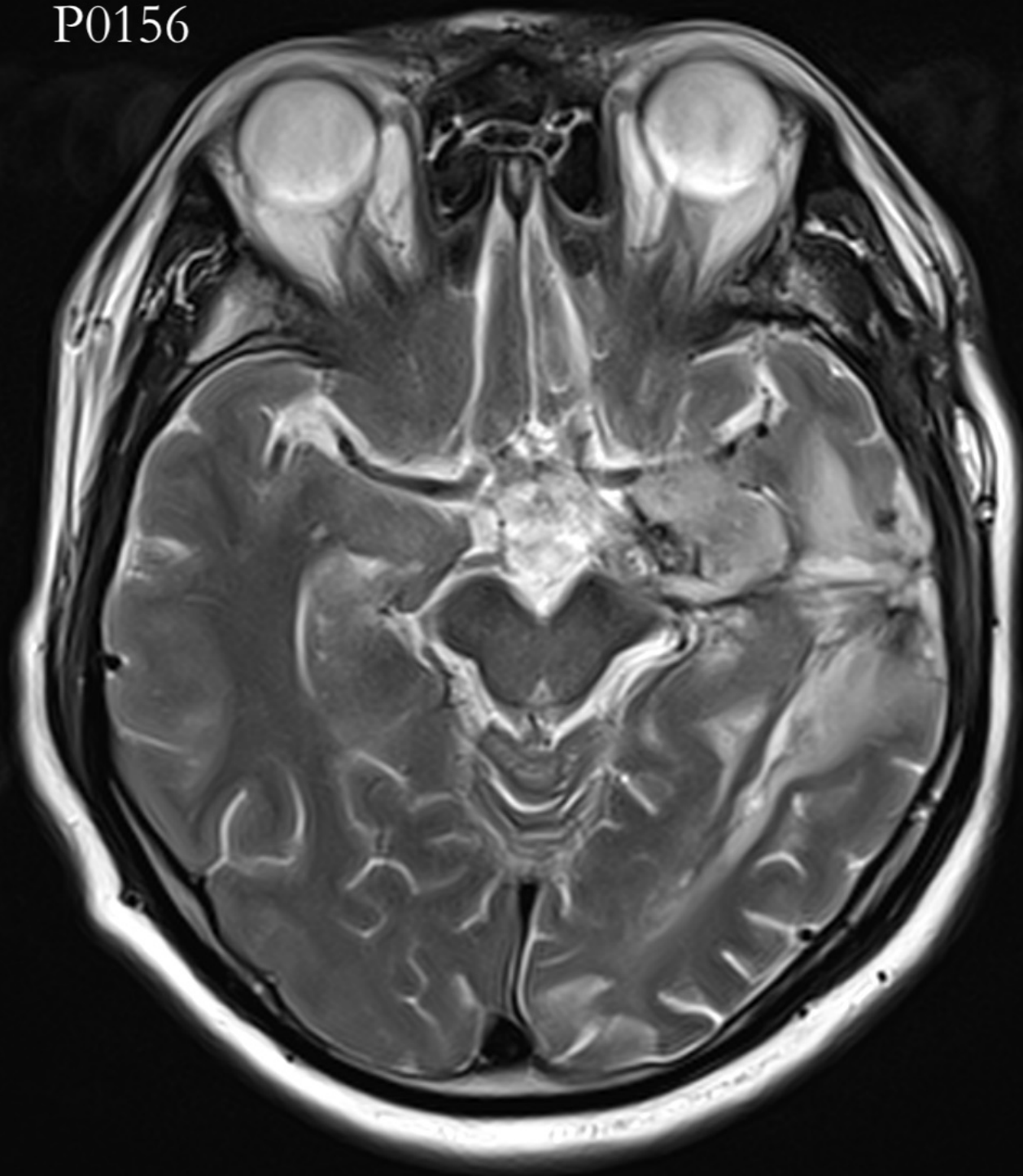

P0163

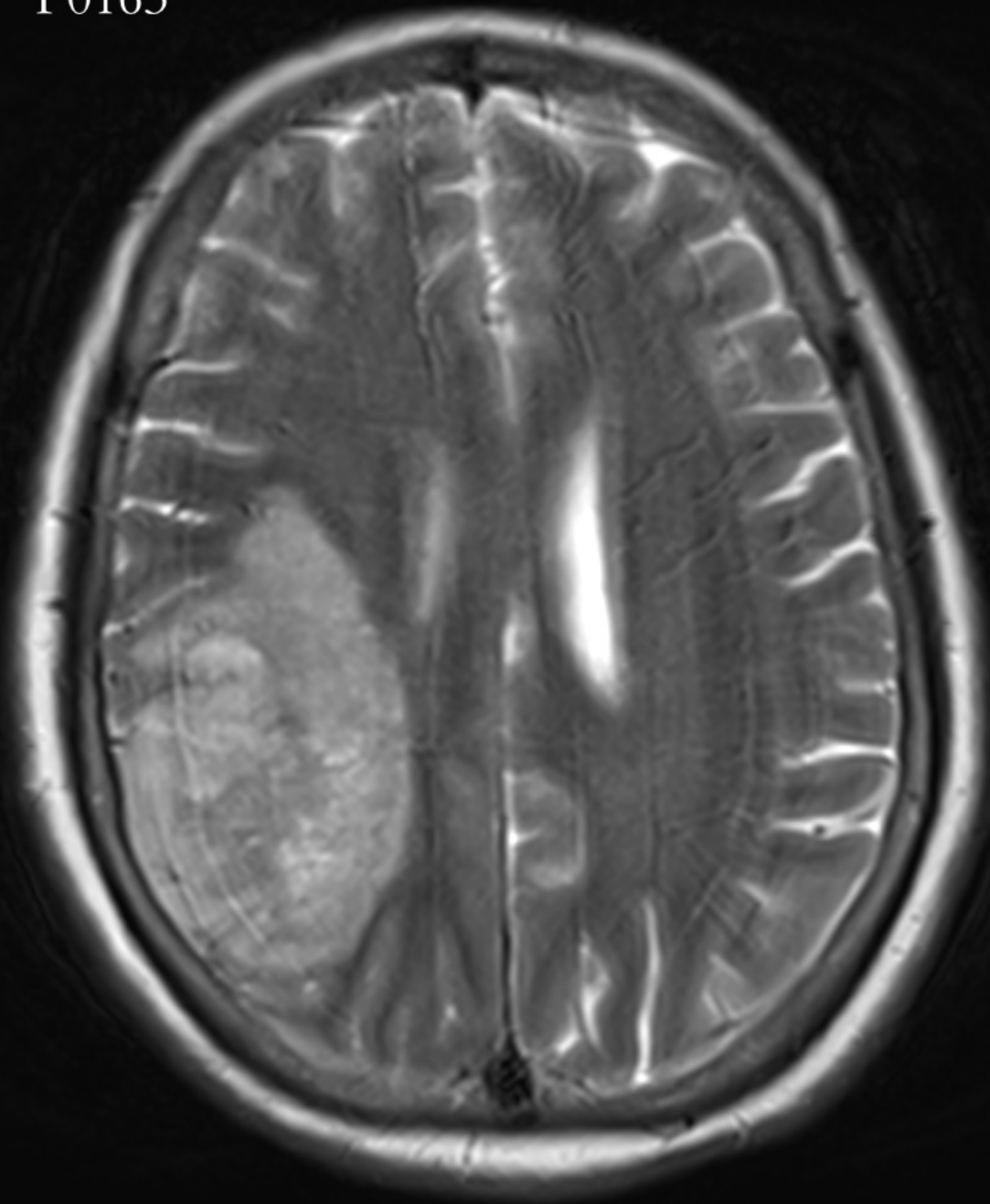

P0167

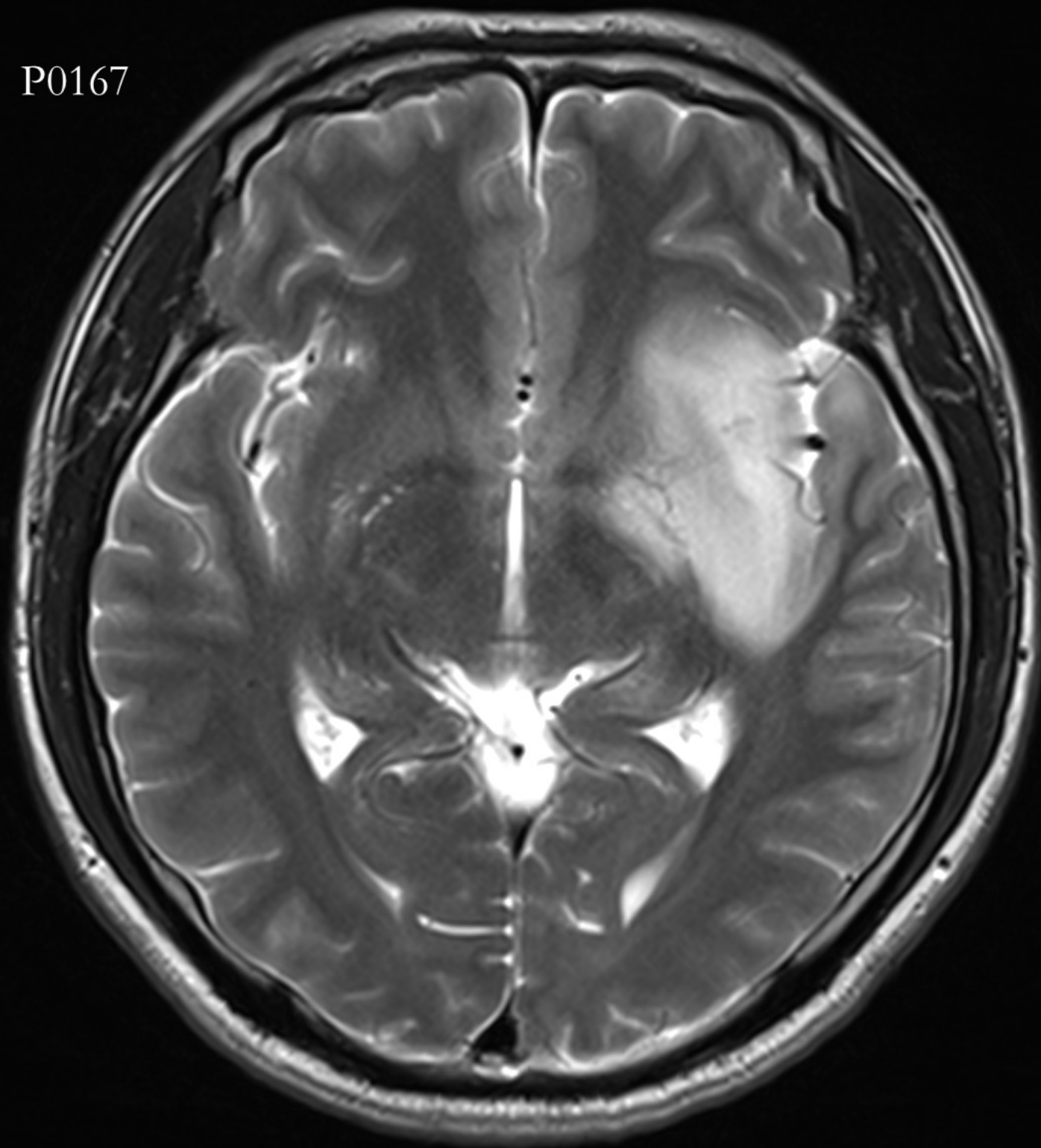

P0169

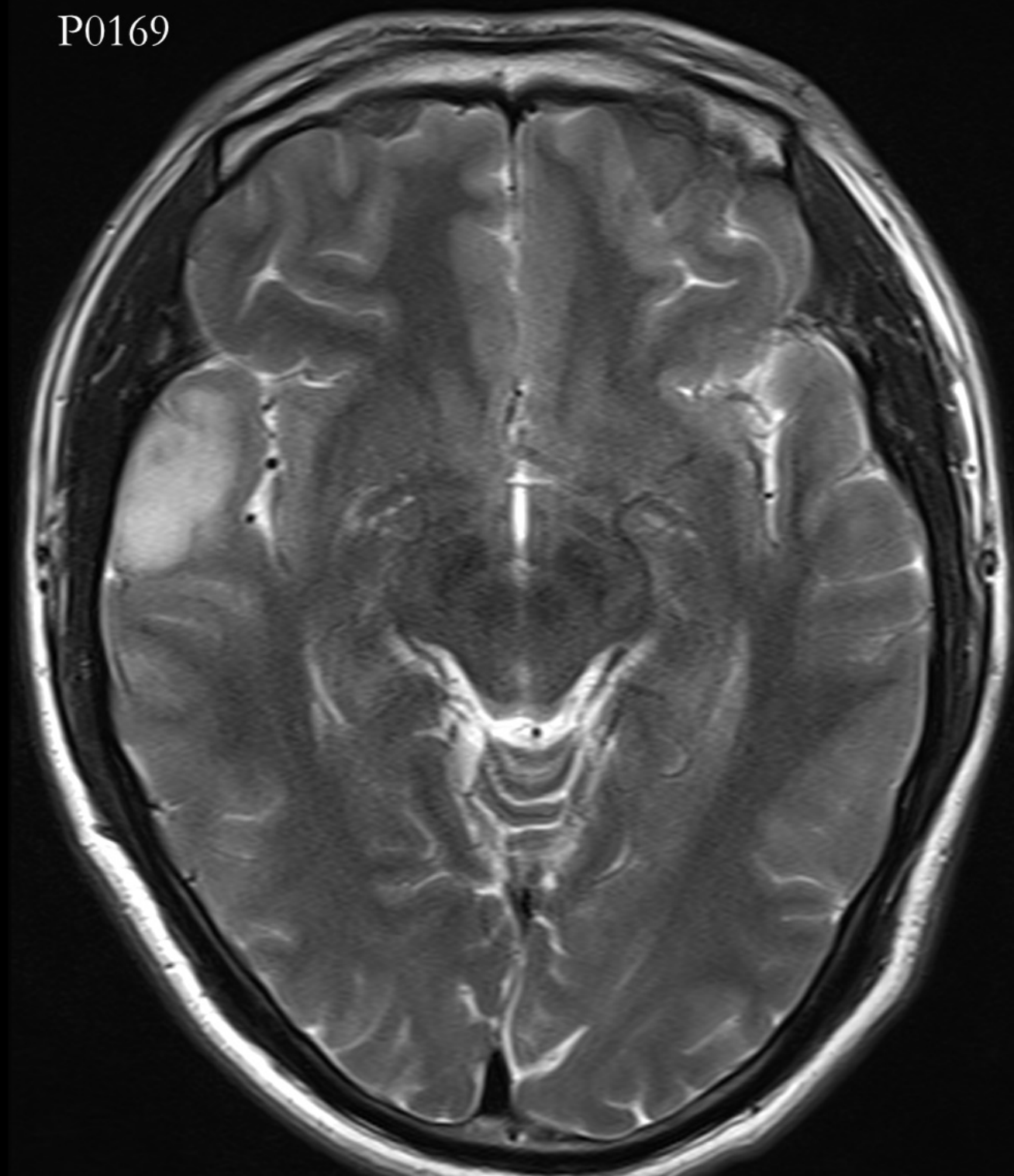

P0170

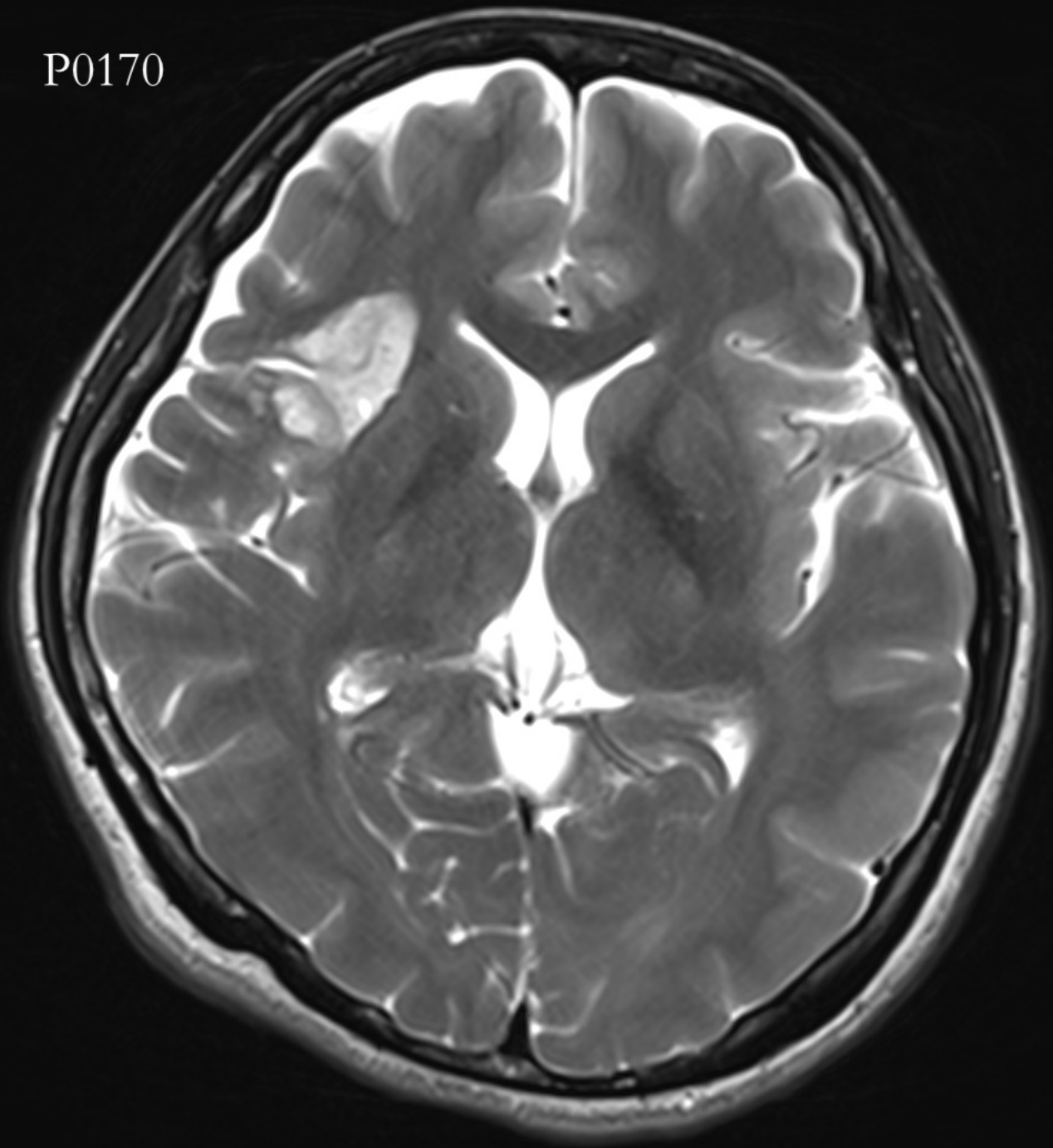

P0171

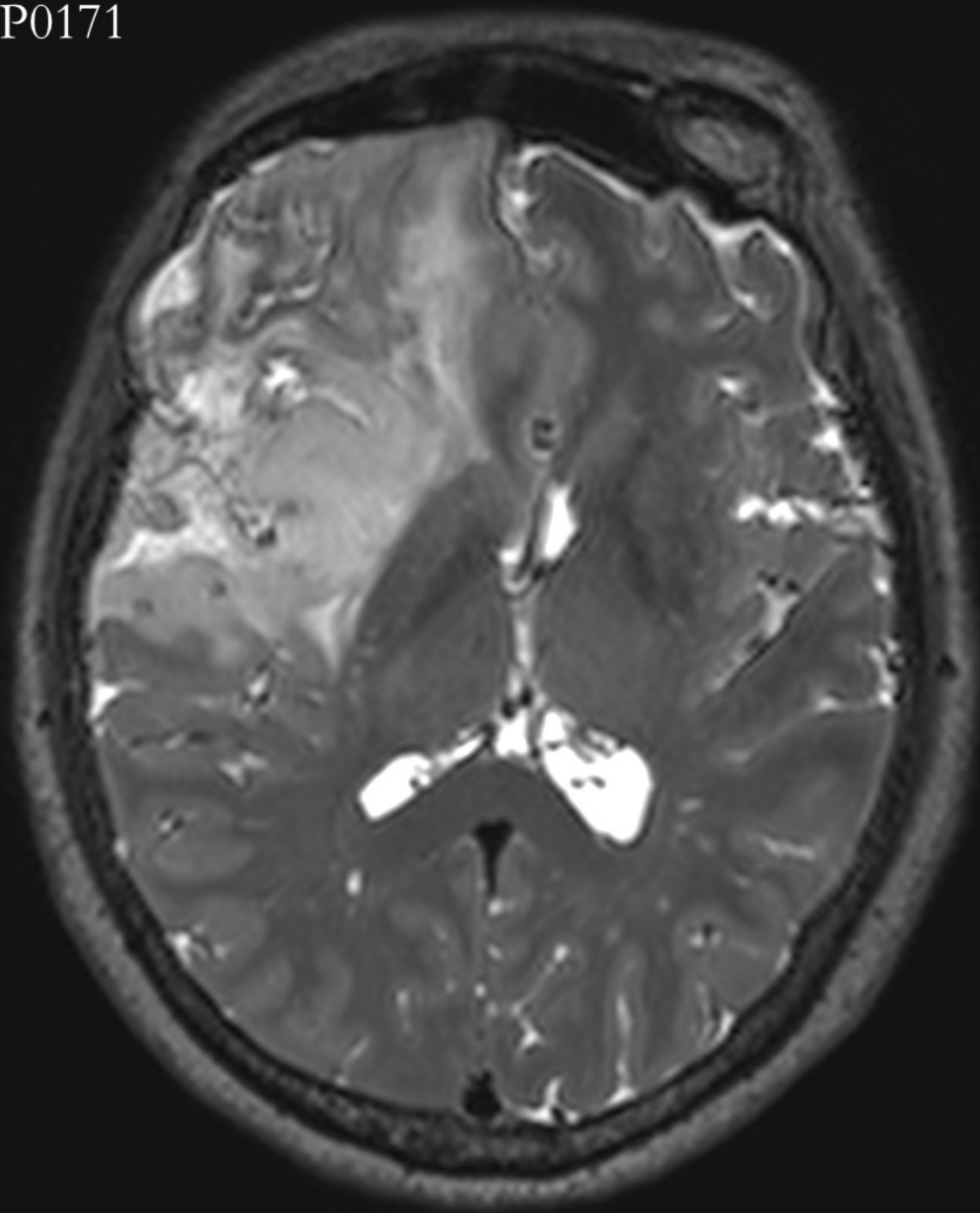

P0174

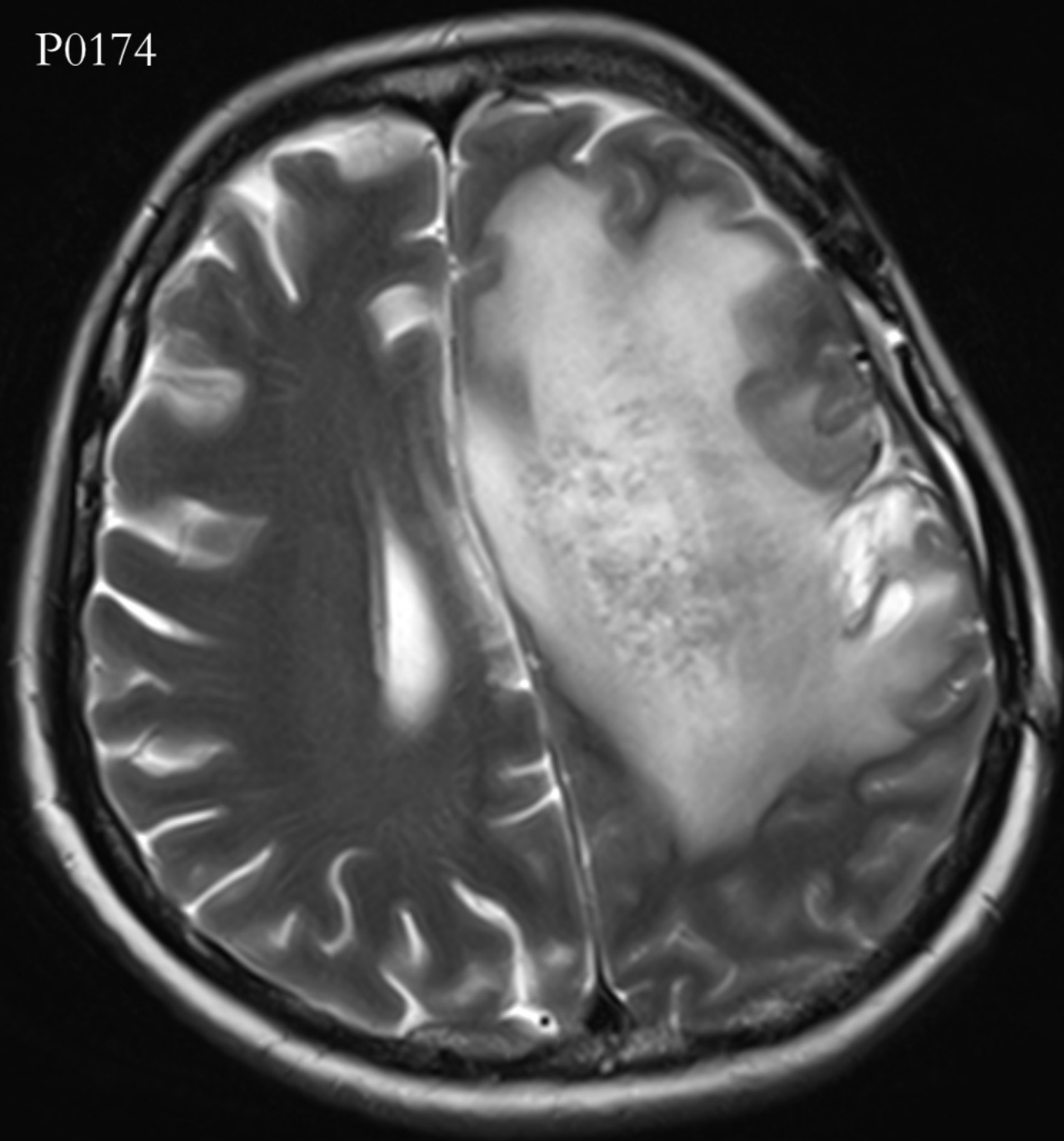

P0175

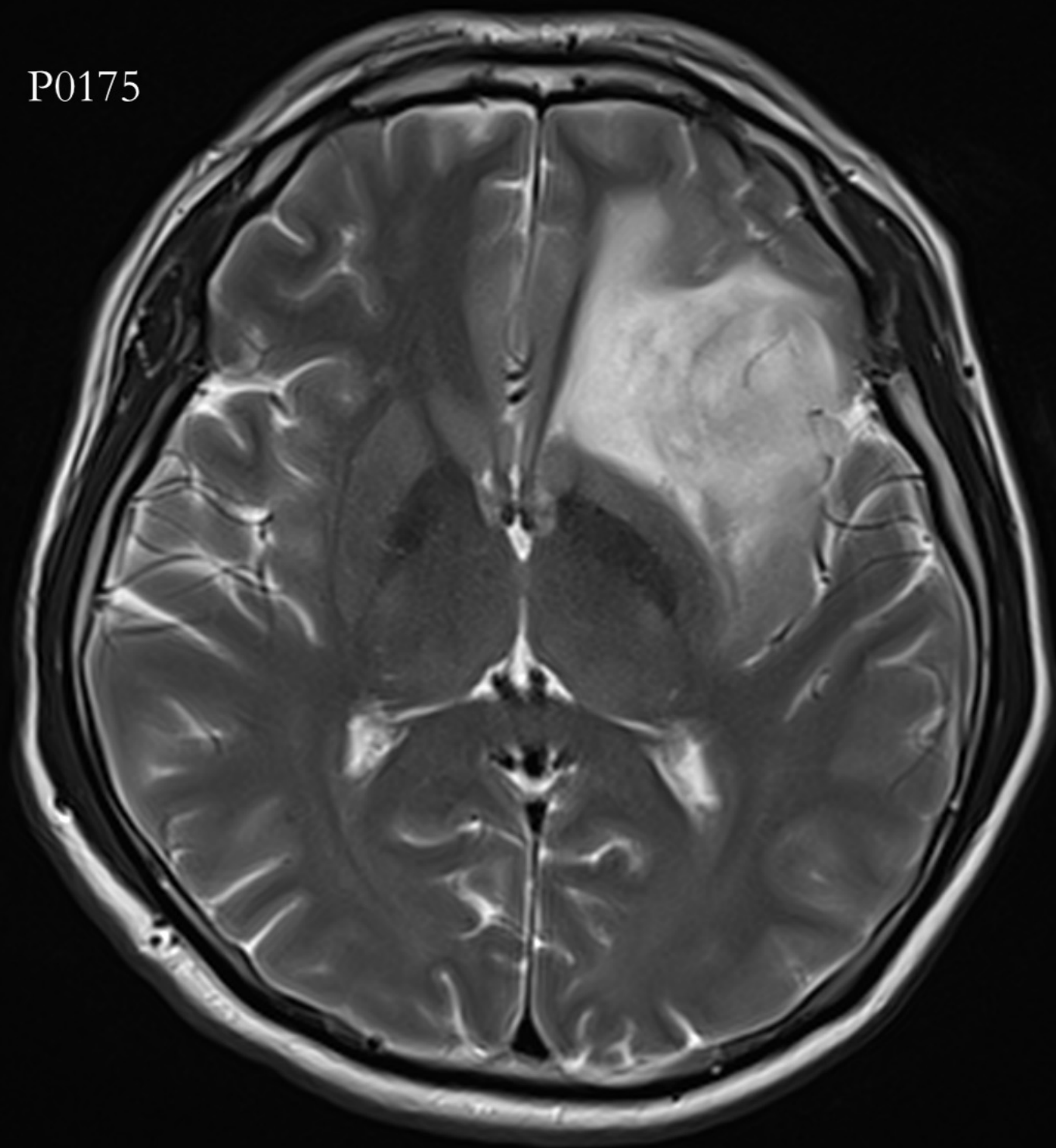

P0179

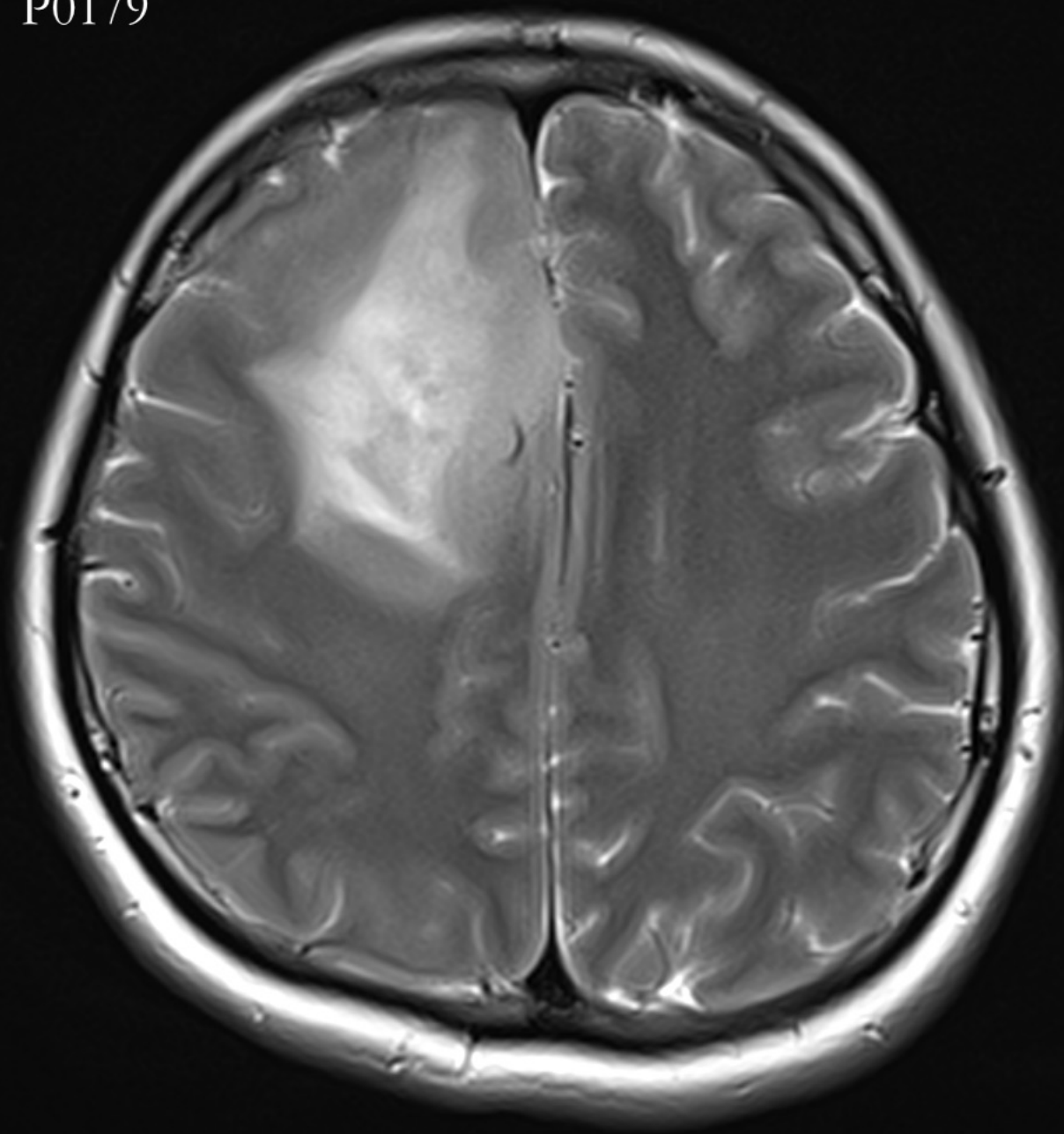

P0189

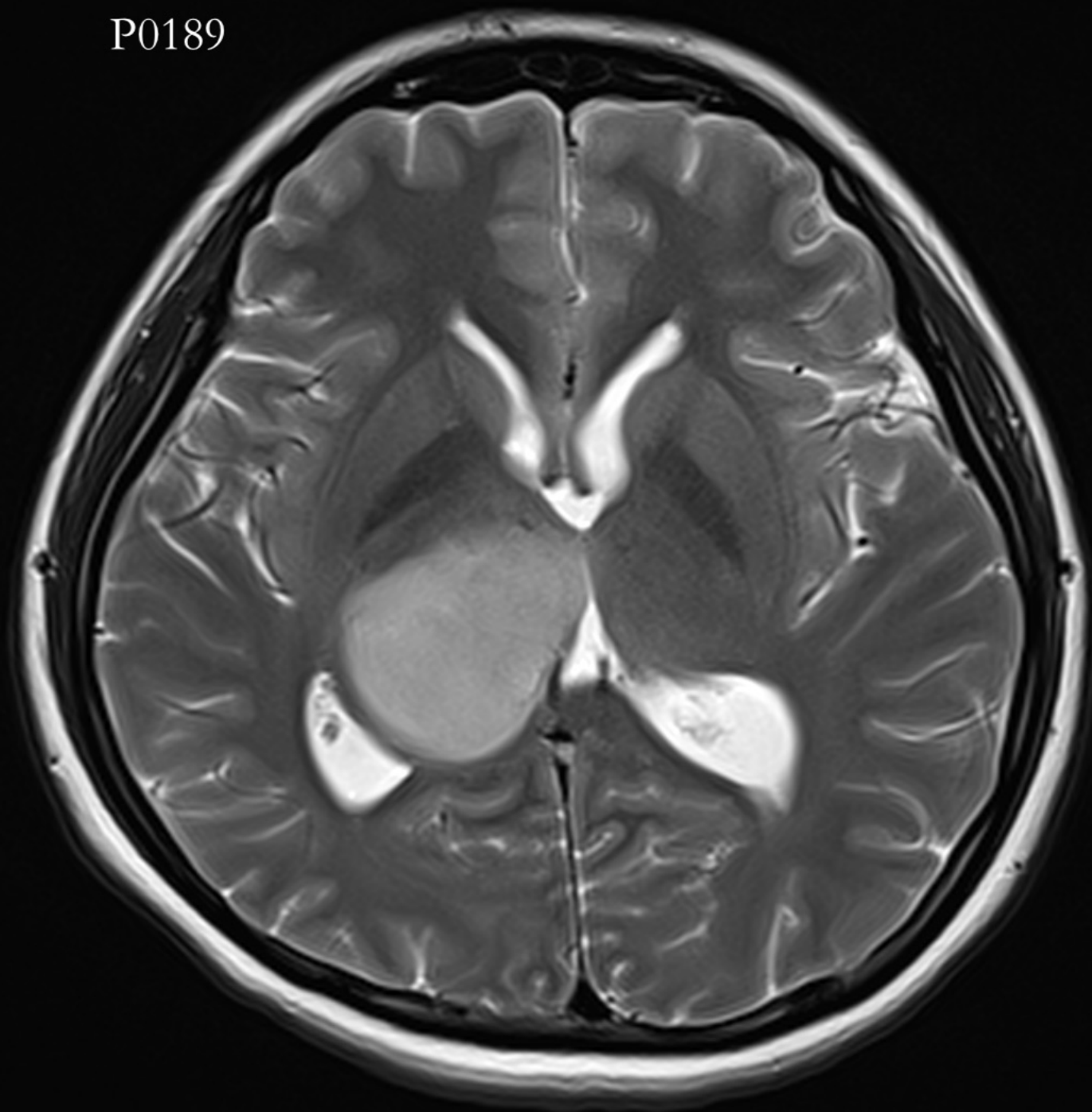

P0191

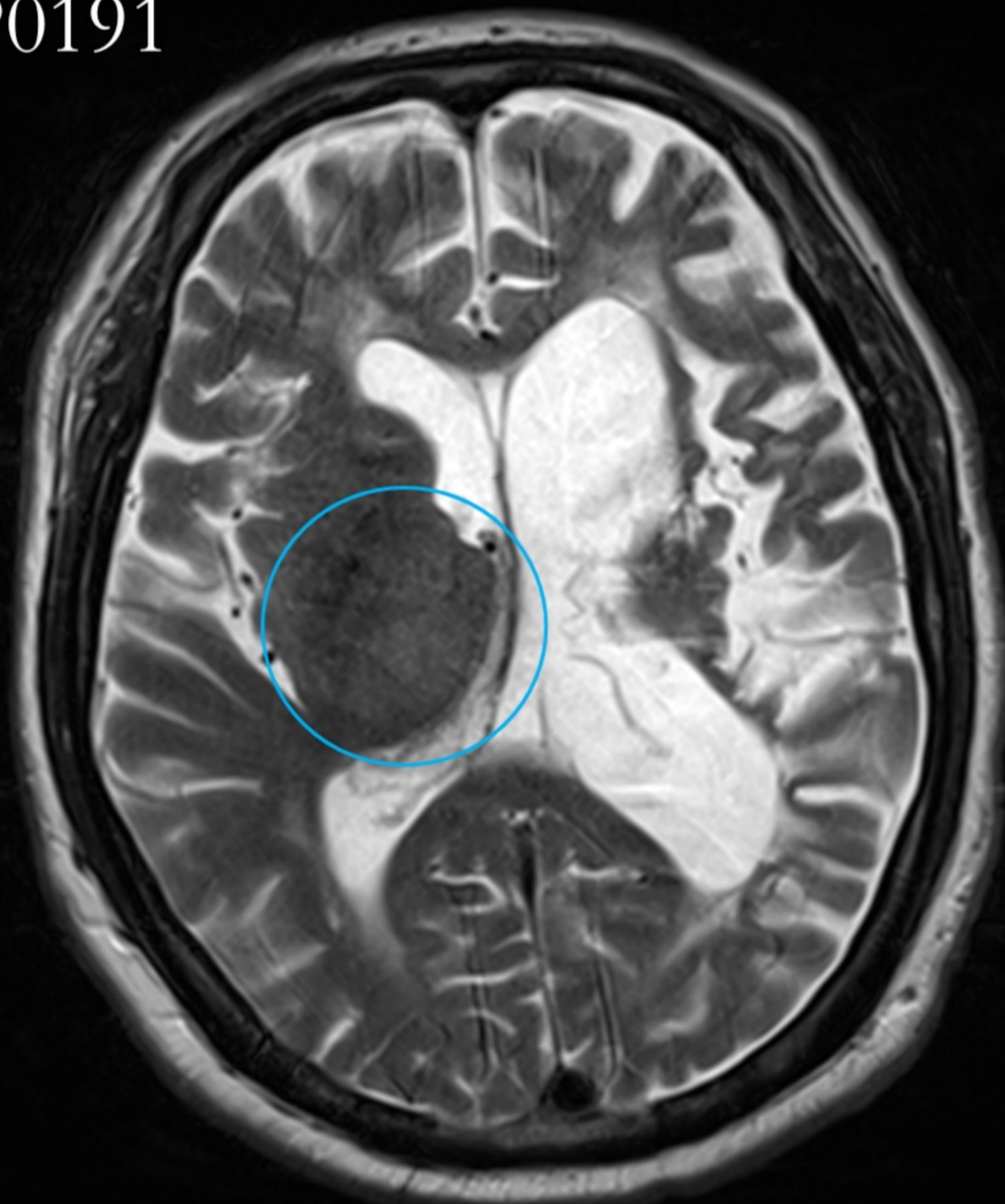

P0194

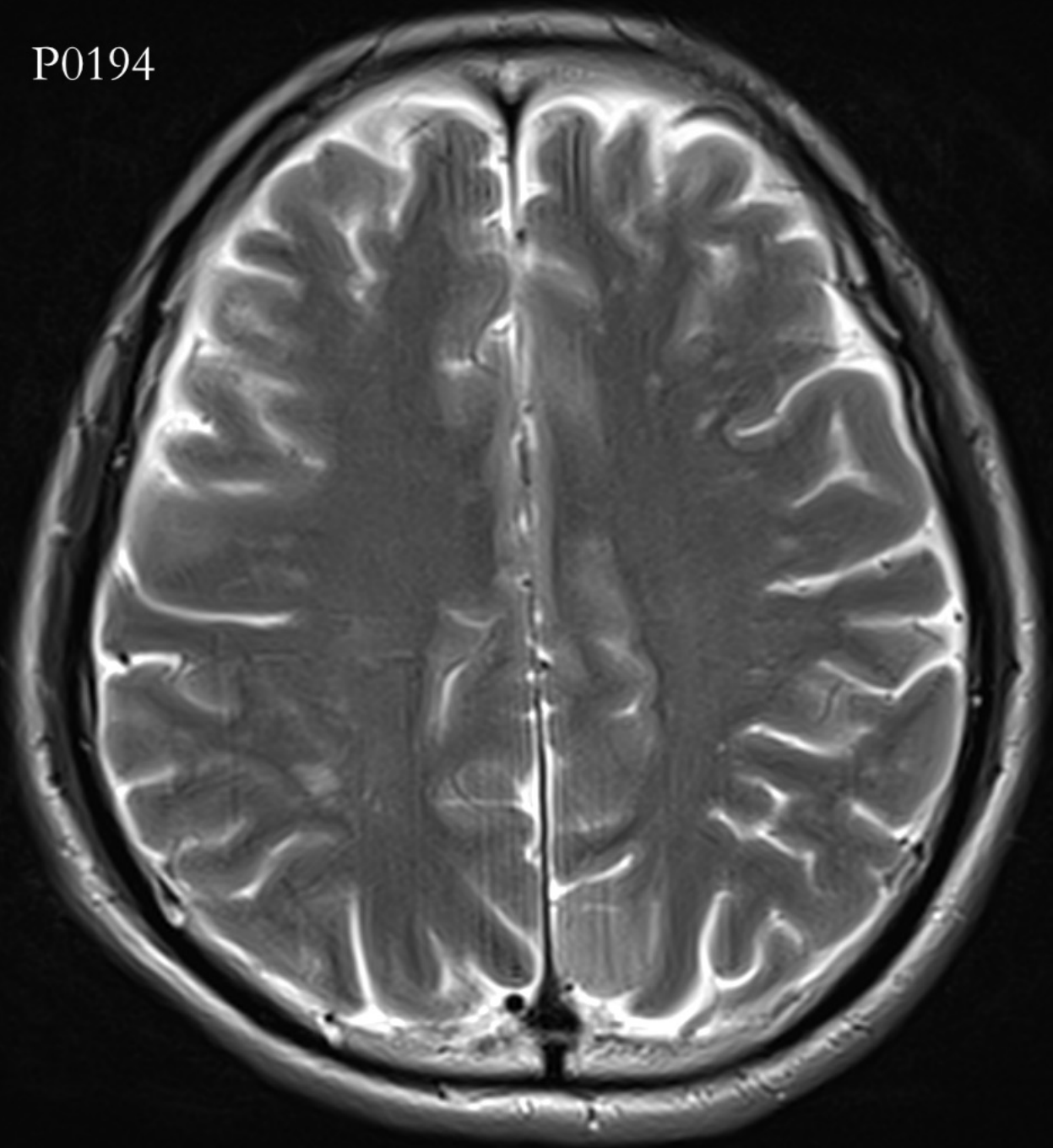

P0195

[RPF]

[LAH]

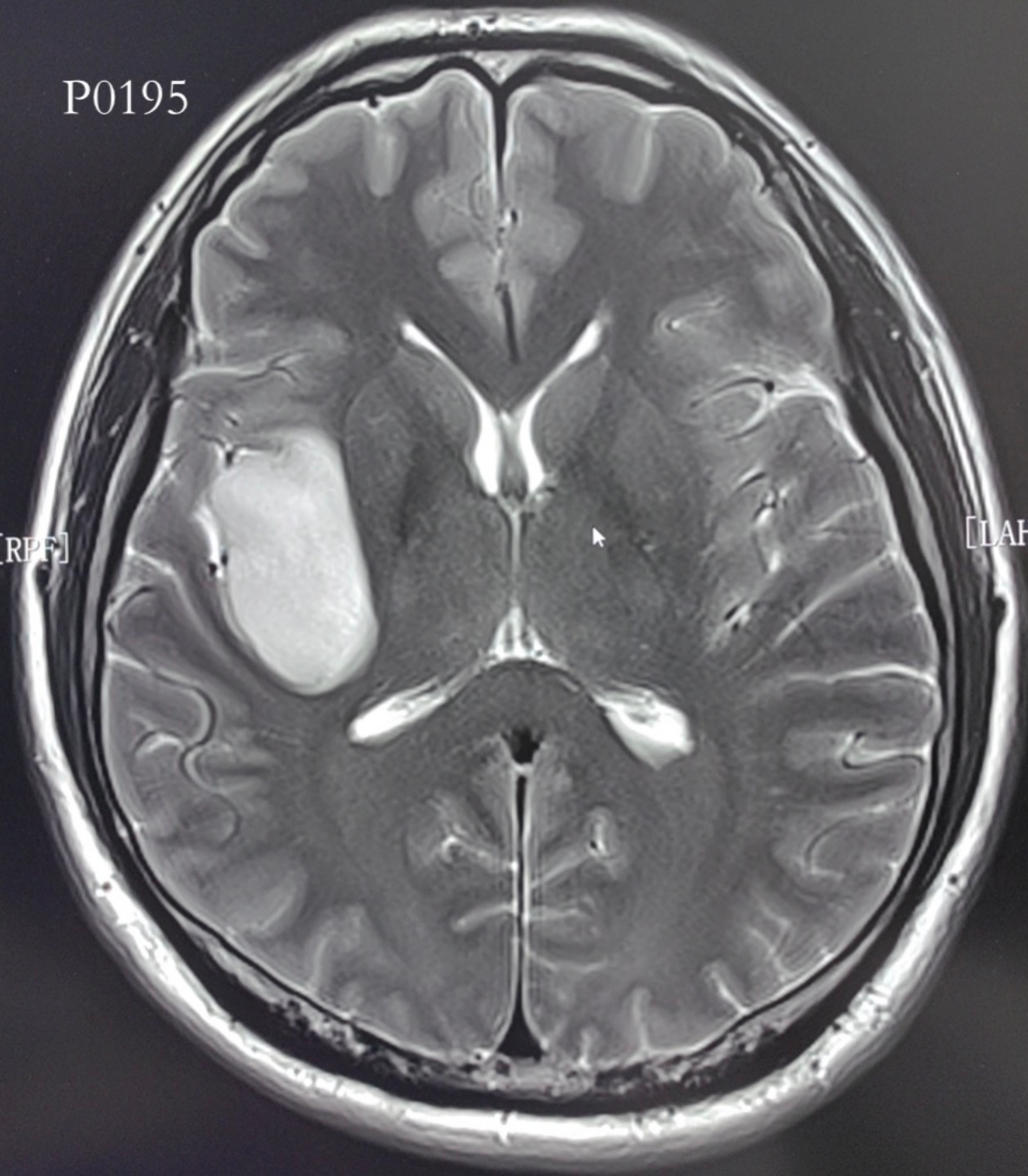

P0199

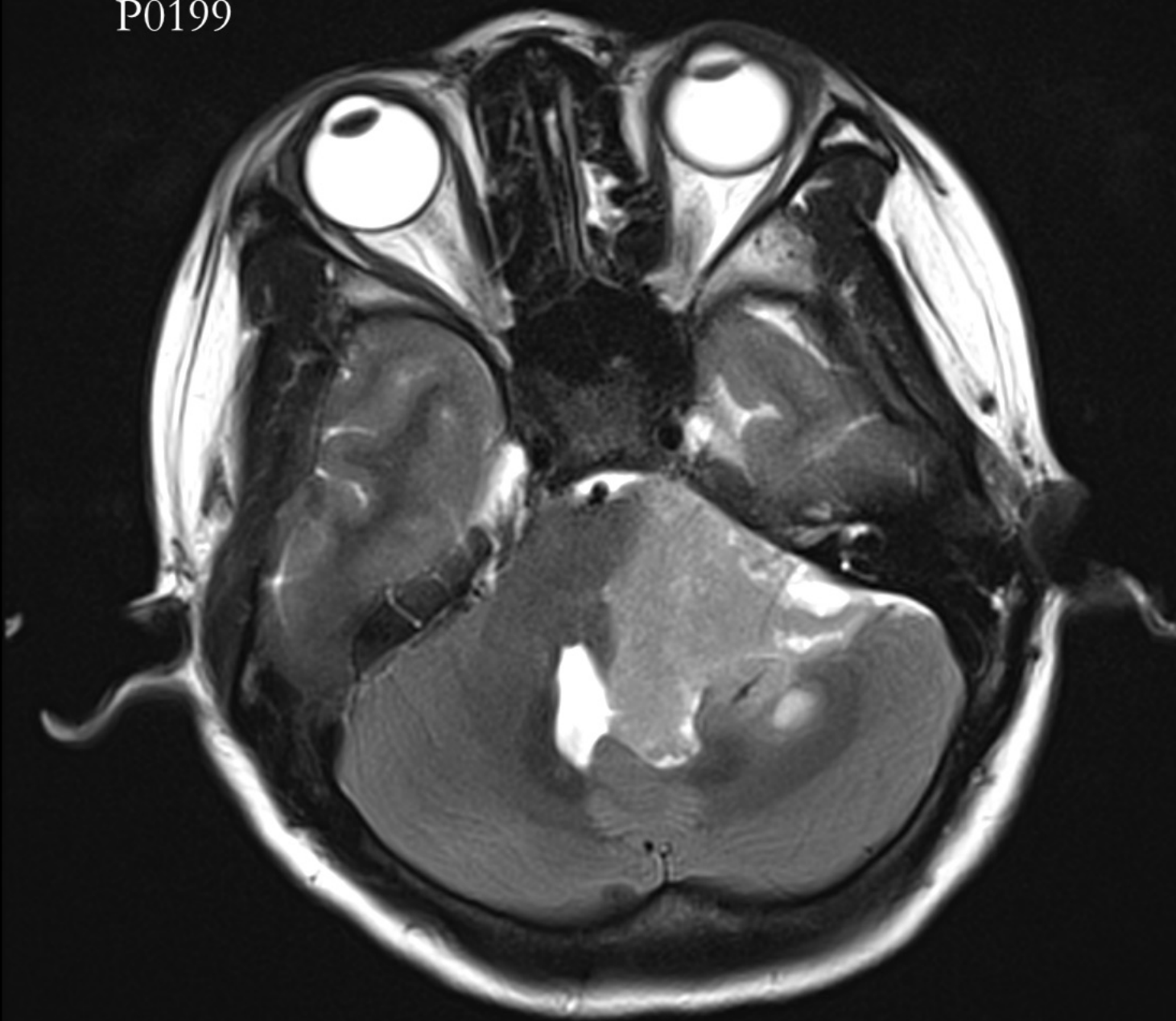

P0200

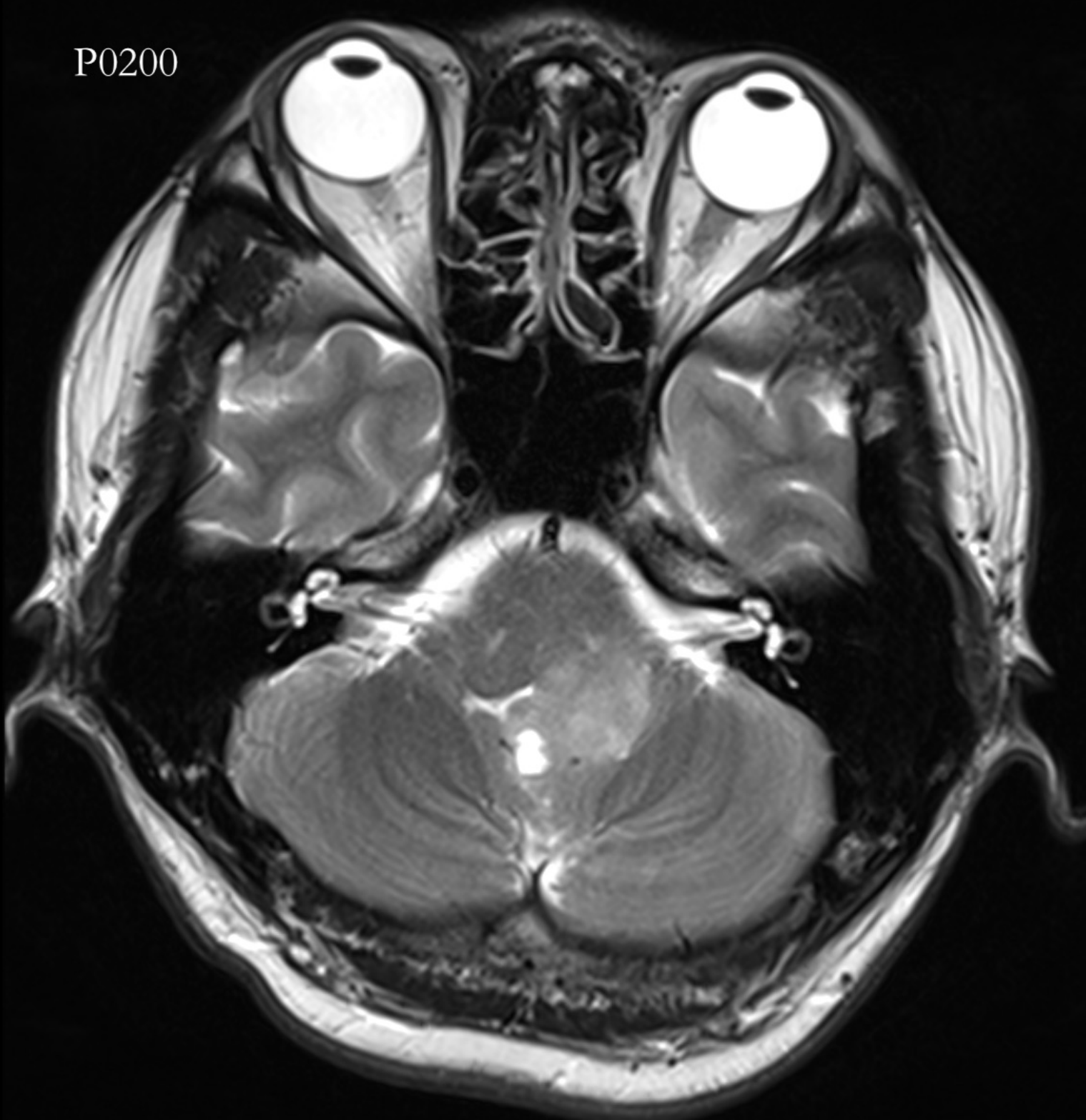

P0204

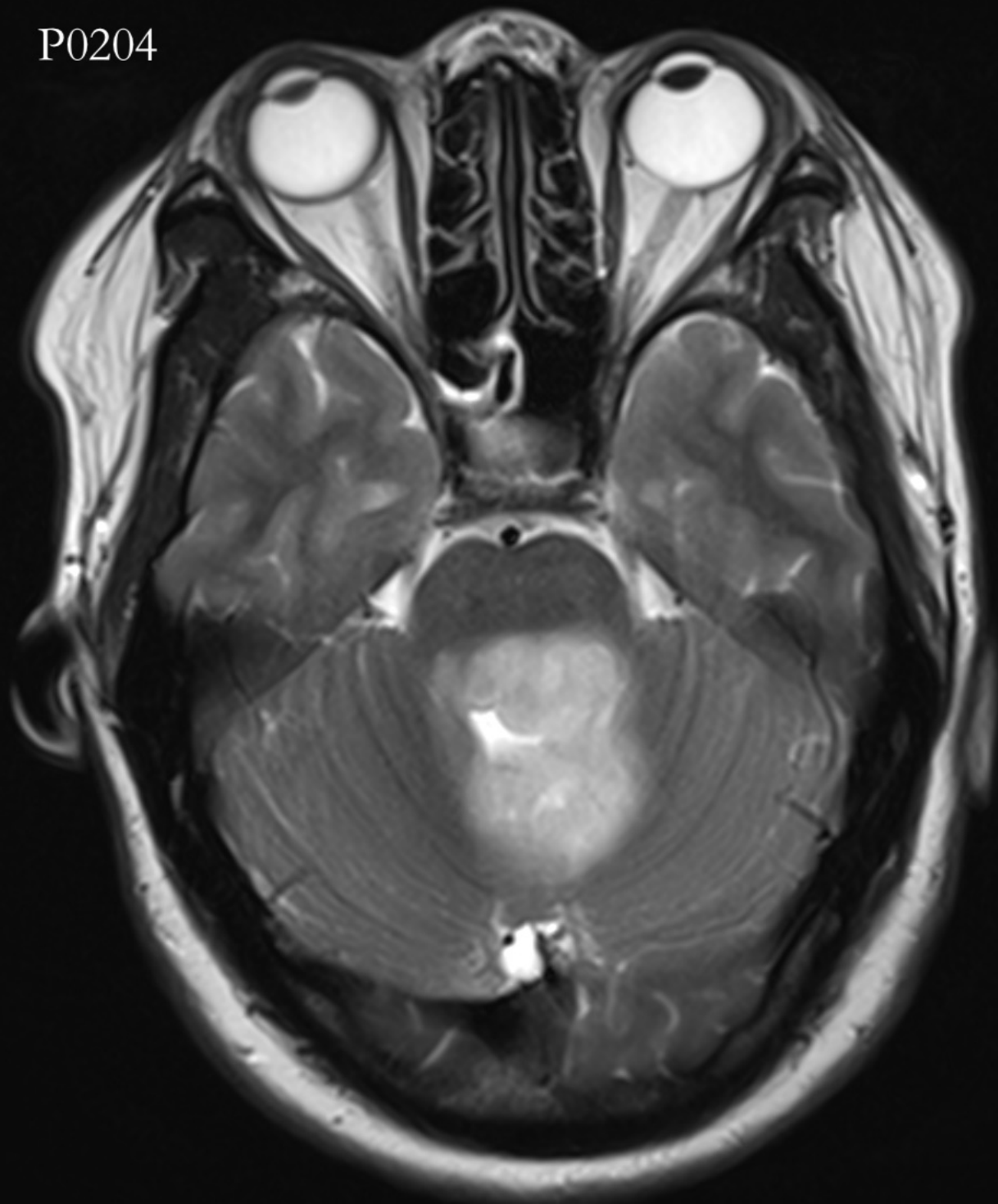

Supplement: Supplementary file 3 — Supplementary Material 3 [file 40644_2024_726_MOESM3_ESM.pdf]
