# Supplementary material for: Identification of T2W hypointense ring as a novel noninvasive indicator for glioma grade and IDH genotype
Source: Cancer Imaging. 2024 Jun 28;24:80. doi: 10.1186/s40644-024-00726-3 (PMC11212435; doi:10.1186/s40644-024-00726-3)
Supplement: Supplementary file 4 — Supplementary Material 4 [file 40644_2024_726_MOESM4_ESM.docx]

| Protocols* | Axial T2WI | Sagittal T2WI | Axial FLAIR | DWI (diffusion mode-3-Scan Trace) | Axial contrast T1WI |
| --- | --- | --- | --- | --- | --- |
| TR (ms) | 4300 | 3740 | 7000 | 1300 | 220 |
| TE (ms) | 98 | 94 | 83 | 62 | 2.46 |
| TI (ms) | / | / | 2217 | / | / |
| B values (s/mm2) | / | / | / | 0/1000 | / |
| Slices | 20-22 | 20 | 20-22 | 20-22 | 20-22 |
| Dist. Factor (%) | 30 | 30 | 30 | 30 | 30 |
| Phase oversampling (%) | 10 | 10 | 0 | 0 | 0 |
| FOV read (mm) | 230 | 230 | 230 | 240 | 230 |
| FOV phase (%) | 81.3 | 100 | 81.3 | 100 | 81.3 |
| Slice thickness (mm) | 5 | 5 | 5 | 5 | 5 |
| Averages | 1 | 1 | 1 | 1 | 1 |
| Voxel size (mm3) | 0.6×0.6×5.0 | 0.7×0.7×5.0 | 0.7×0.7×5.0 | 0.6×0.6×5.0 | 0.7×0.7×5.0 |

*Some of the parameters may be slightly different because of different MRI scanners (MAGNETOM Trio, Vero, Skyra, Prisma, Vida; Siemens Healthcare in Erlangen, Germany).

Abbreviations: FLAIR = fluid-attenuated inversion recovery； DWI = Diffusion-weighted imaging; TR = Repetition time; TE = Echo time; TI = inversion time; FOV = Field of view.

**Secondary Analysis of T2W hypointense ring and T2-FLAIR mismatch sign**

29 cases (15.8%) demonstrated the T2-FLAIR mismatch sign, with 8 cases (27.6%) exhibiting T2W hypointense ring and 21 (72.4%) lacking it. Among the remaining 155 gliomas without the T2-FLAIR mismatch sign, 120 (77.4%) showed T2W hypointense ring and 35 (22.6%) absence.

**Table** Secondary Analysis of T2W hypointense ring and T2-FLAIR mismatch sign

|  | T2-FLAIR mismatch (n=29) | No-T2-FLAIR mismatch (n=155) |
| --- | --- | --- |
| T2W hypointense ring(n=128) | 8 (27.6%) | 120 (77.4%) |
| No T2W hypointense ring (n=56) | 21 (72.4%) | 35 (22.6%) |

T2W: T2-weighted; FLAIR: Fluid-attenuated inversion recovery;
