# Supplementary material for: Identification of T2W hypointense ring as a novel noninvasive indicator for glioma grade and IDH genotype
Source: Cancer Imaging. 2024 Jun 28;24:80. doi: 10.1186/s40644-024-00726-3 (PMC11212435; doi:10.1186/s40644-024-00726-3)
Supplement: Supplementary file 5 — Supplementary Material 5 [file 40644_2024_726_MOESM5_ESM.docx]

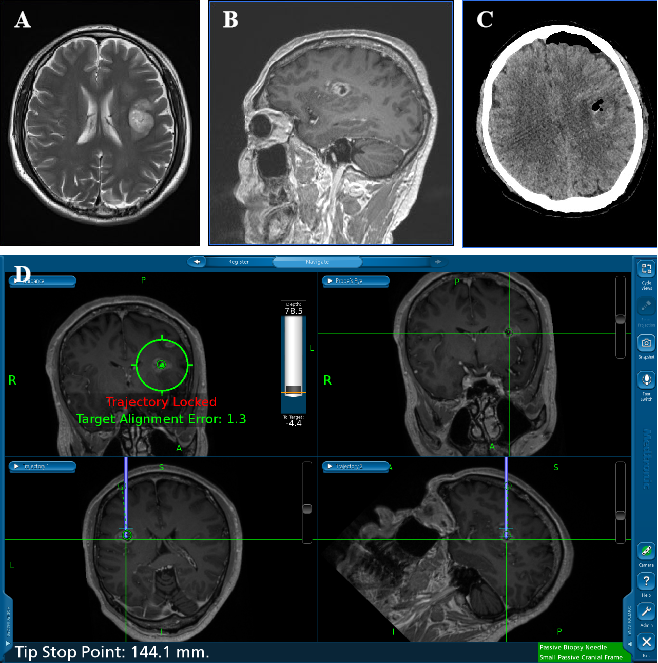


Figure Legend: 53y, M, Amnesia for 0.5y, aphasia for 2ws. A, T2W hypointense ring sign; B, 3D-T1MPRAGE showing lesion enhancement (3D-T2W space_sag_iso sequence could be used instead in subsequent studies); C, CT images reviewed after the puncture procedure; D, Medtronic localized puncture navigation live view allows precise access to the pathologic tissue of interest.
