# Supplementary material for: Identification of T2W hypointense ring as a novel noninvasive indicator for glioma grade and IDH genotype
Source: Cancer Imaging. 2024 Jun 28;24:80. doi: 10.1186/s40644-024-00726-3 (PMC11212435; doi:10.1186/s40644-024-00726-3)
Supplement: Supplementary file 6 — Supplementary Material 6 [file 40644_2024_726_MOESM6_ESM.docx]

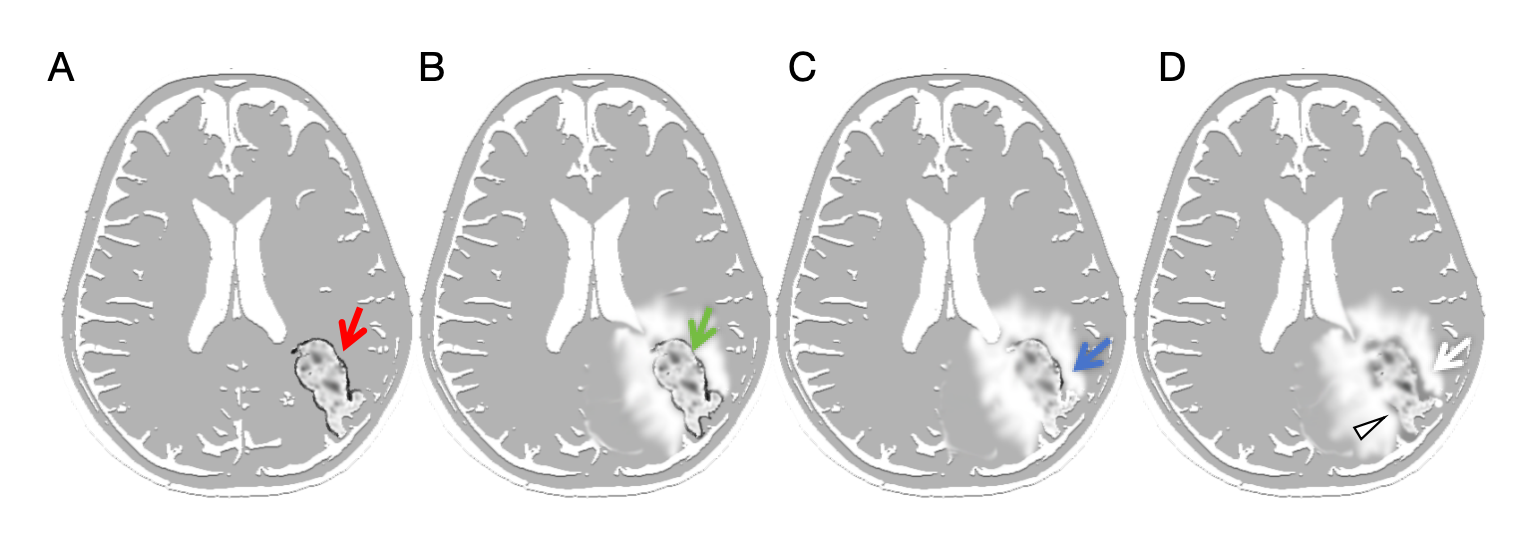


Figure Legend: MRI-T2W schematic of glioma hypointense ring sign. A, a clear T2W hypointense ring (red arrow) surrounded by the tumor margin and normal brain tissue, with no obvious edema signal between the ring and normal brain tissue; B, a slightly hypointense signal surrounded by the tumor margin around the entire circumference of the tumor body (green arrow), with a more obvious edema between the ring and the normal brain tissue; C, a slightly hypointense ring (blue arrow) on the left side of the tumor body; C, a slightly hypointense rim (blue arrow) on the left side of the tumor body, which surrounds the tumor for less than semiring, is more faint compared to A and B, but still shows its presence; D, a discontinuous hypointense ring (white arrows) at the margin of the tumor, which is broken off as shown by the white arrows, which are thicker compared to the hypointense rings in A, B, and C.
